# Assisting Australians with mental health problems and financial difficulties: a Delphi study to develop guidelines for financial counsellors, financial institution staff, mental health professionals and carers

**DOI:** 10.1186/s12913-015-0868-2

**Published:** 2015-06-03

**Authors:** Kathy S. Bond, Kathryn J. Chalmers, Anthony F. Jorm, Betty A. Kitchener, Nicola J. Reavley

**Affiliations:** Mental Health First Aid Australia, Level 6/369 Royal Parade, Parkville, VIC 3052 Australia; Centre for Mental Health, Melbourne School of Population and Global Health, The University of Melbourne, Level 4/207 Bouverie St, Parkville, VIC 3010 Australia; School of Psychology, Deakin University, Melbourne, Australia

**Keywords:** Financial counsellors, Financial and banking staff, Consumers, Carers, Financial difficulties, Debt, Mental illness, Delphi

## Abstract

**Background:**

There is a strong association between mental health problems and financial difficulties. Therefore, people who work with those who have financial difficulties (financial counsellors and financial institution staff) need to have knowledge and helping skills relevant to mental health problems. Conversely, people who support those with mental health problems (mental health professionals and carers) may need to have knowledge and helping skills relevant to financial difficulties. The Delphi expert consensus method was used to develop guidelines for people who work with or support those with mental health problems and financial difficulties.

**Methods:**

A systematic review of websites, books and journal articles was conducted to develop a questionnaire containing items about the knowledge, skills and actions relevant to working with or supporting someone with mental health problems and financial difficulties. These items were rated over three rounds by five Australian expert panels comprising of financial counsellors (n = 33), financial institution staff (n = 54), mental health professionals (n = 31), consumers (n = 20) and carers (n = 24).

**Results:**

A total of 897 items were rated, with 462 items endorsed by at least 80 % of members of each of the expert panels. These endorsed statements were used to develop a set of guidelines for financial counsellors, financial institution staff, mental health professionals and carers about how to assist someone with mental health problems and financial difficulties.

**Conclusions:**

A diverse group of expert panel members were able to reach substantial consensus on the knowledge, skills and actions needed to work with and support people with mental health problems and financial difficulties. These guidelines can be used to inform policy and practice in the financial and mental health sectors.

**Electronic supplementary material:**

The online version of this article (doi:10.1186/s12913-015-0868-2) contains supplementary material, which is available to authorized users.

## Background

There is an association between mental health problems and financial difficulties [1–3]. A national survey in the UK found that people in debt were three times more likely to have a depressive or anxiety disorder than those without debt [4]. Furthermore, Australian research has found that people who report financial hardships (e.g. being unable to heat their home, having to sell possessions, going without meals) are more likely to have mental health problems [1]. For example, the 12-month prevalence rates of depression were 7.5 % in people who report one financial hardship and 13 % in people with multiple financial hardships, compared to 3 % of people reporting no hardships. Similarly, people on income support have a higher rate of mood, anxiety and substance use disorders [5]. A 2012 Australian study of people with psychotic disorders found that 43 % of participants cited financial problems as a major challenge in the coming year [6]. Moreover, an analysis of Australian coroner reports on deaths by suicide found that financial problems were a factor in 9 % of these deaths [7].

Research indicates that the relationship between financial difficulties and mental health problems is complex [8]. Mental health problems can lead to financial difficulties, for example, lack of motivation to pay bills due to depression, shopping and overspending in order to feel better, poor financial decisions and overspending during a manic or psychotic episode [9]. On the other hand, increased number of debts has been shown to increase the likelihood of mental health problems [10]. Furthermore, there are common risk factors for both financial difficulties and mental health problems, such as a lower level of education [11, 12], lower rates of employment [13] and higher rates of underemployment [14, 15].

Therefore, people who work with those who have financial difficulties (financial counsellors and financial institution staff) need to have knowledge and helping skills relevant to people with mental health problems. Conversely, people who support those with mental health problems (mental health professionals and carers) may need to have knowledge and helping skills relevant to people with financial difficulties.

There is limited guidance on best practice for people who work with or care for those with both mental health problems and financial difficulties. A major exception is a series of projects carried out in the UK, which involved the development of guidelines and information for creditors and money advisors (who have a similar role to financial counsellors in Australia). (For examples see *Good Practice Awareness Guidelines for consumers with Mental Health Problems and Debt* [16]; *In the Red* [17]; *Debt collection and mental health: Ten steps to improve recovery* [18]; *Final Demand: Debt and Mental Illness* [19].) The guidelines were developed through a process involving narrative and systematic literature reviews [9, 20–23], expert committees, and a survey of 178 creditors, debt collection agencies and debt purchasers [24]. An important part of this work was the development of a standardised form to allow mental health professionals to communicate information to creditors and money advisors about how a person’s mental health problems affect their ability to manage their financial difficulties. This *Debt and Mental Health Evidence Form* was launched in 2008 [25]. A survey of nearly 1300 debt collection staff found that 84 % thought that the medical information in the form influenced their decision making, with 76 % finding the information to be relevant and 24 % agreeing that using the medical evidence had assisted them in recovering debt [24].

Financial difficulties are strongly influenced by the context within which they are experienced. For instance, government financial support and community services (e.g. financial counselling services, mental health services) vary from country to country or even between local areas within a country [26]. Furthermore, national and state laws can impact on the experience and resolution of financial problems. For example the privacy laws that govern the management of personal information vary between countries. The Australian Privacy Act [27] states that financial institutions cannot use or disclose personal information about an individual for any purpose other than what it was collected for. This has several implications. First, this limits the financial institutions ability to communicate with financial counsellors and mental health professionals. Second, if a financial institution collects mental health information about a customer who is experiencing financial difficulties due to their mental health problems, this information cannot be used to assess the customer for a future loan application. This restriction poses a dilemma for financial institutions, because they are also required to act responsibly when assessing customers for loans, and mental health information may or may not be pertinent in this case. Because of these contextual differences, work carried out in the UK cannot necessarily be generalised to other countries, including Australia [27].

While some resources have been developed for Australian financial counsellors and mental health professionals [28–30], these are limited in a number of ways: they were developed for one Australian state and did not cover some important stakeholders, notably financial institution staff and carers.

Therefore, we carried out a Delphi expert consensus study to develop Australian national guidelines that are tailored to the needs of financial counsellors, financial institution staff, mental health professionals and carers. This research was conducted in collaboration with the peak body for financial counsellors (Financial Counselling Australia), a consortium of financial institutions (ANZ, GE Money, NAB, Westpac), and Australia’s national depression and anxiety initiative (*beyondblue*).

## Methods

The Delphi process is an expert consensus method that can be used to develop best practice guidelines using practice-based evidence [31]. The advantage of the Delphi method over other methods used in the projects described above, such as expert working groups and focus groups, is that the expert opinion is gathered anonymously through the use of online (or postal) surveys [32], allowing for all participants on the panel to equally influence the results. One disadvantage to the Delphi method is the lack of discussion that can take place in working or focus groups, which allows for biases and incorrect assumptions to be challenged. Most often, the Delphi method involves the use of one expert panel. However, more recent work in the mental health area has included multiple panels, including consumer and carer experts, giving equal weight to all panels [33–43].

Development of the current guidelines involved four steps: (1) formation of the expert panels, (2) literature search and survey questionnaire development, (3) data collection and analysis, and (4) guidelines development [31].

### Step 1: Panel formation

This study utilised five expert panels: financial counsellors, financial institution staff, mental health professionals, mental health consumer advocates and carer advocates. All panelists had to be 18 years or older, living in Australia, and have either professional or personal experience with mental health problems and financial difficulties. Table 1 presents specific inclusion criteria and recruitment methods for each of the expert panels.

**Table 1:** Expert panel inclusion criteria and recruitment methods

Expert panel	Inclusion criteria	Recruitment method
Financial counsellors	1. A financial counsellor with at least 2-years experience, or a manager of a financial counselling service or a service that includes at least one financial counsellor	Advertisements through Financial Counselling Australia and word of mouth
	2. Experience working with people with mental health problems and/or have personal experience with mental health problems	
Financial institution staff (included banking staff, banking ombudsman staff and Australian Bankers’ Association staff)	1. At least 2-years combined experience in, or as a manager of, a collection, hardship or complaints department	Advertisement sent out by banking experts on the research work group and the financial institution consortium
	2. Have industry insight into hardship management	
Mental health professional	1. At least 2-years relevant experience	Advertisements sent out to mental health services around Australia
	2. Experience working with people with financial difficulties.	
Consumer	1. Experience with mental health problems and financial difficulties	Advertisements through mental health advocacy organisations
	2. The financial difficulties will ideally be resolved and mental health symptoms well managed	
	3. A member of an advocacy organisation	
Carer	1. Currently, or in the past, care for a person who has experienced mental health problems and financial difficulties	Advertisements through mental health advocacy organisations
	2. A member of an advocacy organization	

The aim was to recruit a minimum of 30 people to each of the five panels so that the panel size would be within the typical Delphi panel size of 15–60 experts [32], allowing for reliable consensus to be reached. A panel size of 23 has been found to yield stable results in a simulation study [44].

### Step 2: Literature search and survey questionnaire development

**Fig. 1:**
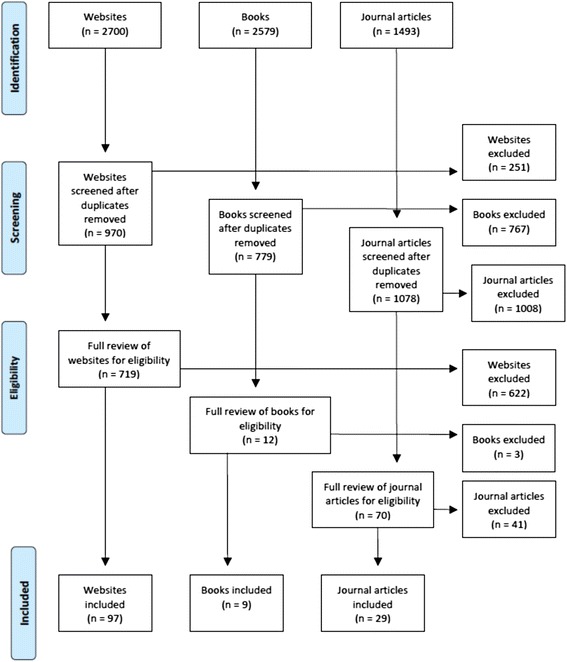
Literature review results

A systematic search of the ‘grey’ and academic literature was conducted in October and November of 2012 to gather information about how to help someone with mental health problems and financial difficulties. The search was conducted using Google Australia, Google UK, Google USA, Google Books and PubMed. The key search terms used were: (debt OR financial strain) AND (mental health OR mental illness OR depression OR anxiety OR suicide OR self-harm OR bipolar OR schizoaffective). The top 50 websites, books and journal articles were retrieved and reviewed for relevant information. Any links appearing on the websites were also reviewed. A total of 97 websites, 9 books and 29 journal articles were included. Figure 1 summarises the literature review results.

A working group, consisting of staff from Mental Health First Aid Australia, the University of Melbourne, financial institutions and financial counselling services, translated the relevant information into statements that were clear, actionable, and contained only one idea. The statements were used to form a questionnaire that was administered to the expert panels via SurveyMonkey. The panel members were asked to rate each of the statements, using a 5-point scale (‘essential’, ‘important’, ‘don’t know/depends’, ‘unimportant’ or ‘should not be included’), according to whether or not they thought the statement should be included in the guidelines. See Additional files 1, 2 and 3 for copies of the Round 1, 2 and 3 surveys.

### Step 3: Data collection and analysis

Data were collected in three survey rounds administered between July and December 2013. In Round 1, panel members had the opportunity to provide comments or suggest new statements. After panel members completed a survey round, the statements were categorised as follows:

1. Endorsed. The item received an ‘essential’ or ‘important’ rating from 80-100 % of members of all five panels.
2. Re-rate. The item was not endorsed, but received an ‘essential’ or ‘important’ rating from 75 % or above of members of all panels.
3. Rejected. The item did not fall into either the endorsed or re-rate categories.

The panel comments were analysed by the Working Group for any new content. This new content was translated into clear and actionable statements for the Round 2 questionnaire. Panel members were given a summary report of Round 1 that included a list of the items that were endorsed and rejected, as well as the items that were to be re-rated. The report included the panel percentages of each rating, as well as their individual scores for each item to be re-rated. The participants were asked to consider whether to maintain or change their rating.

The procedures for Rounds 2 and 3 were the same as described above with two exceptions. There was no opportunity for comments in either of these rounds, and if a re-rated item did not receive an ‘essential’ or ‘important’ rating by 80 % or more of each panel, it was excluded.

### Step 4: Guidelines development

All of the endorsed statements were written into prose to form the guidelines document. This document was given to the expert panel members for comment and final endorsement.

### Ethics

This research was approved by the Australian Government Department of Health and Ageing Ethics Committee. Written informed consent was obtained from all participants by clicking ‘yes’ to a question about informed consent in the SurveyMonkey survey.

## Results

The response rate for completing all three rounds was 47.7 % (see Table 2 for the breakdown of the response rate for each of the panels). Participants who completed all rounds were 74.1 % male, 24.7 % female, and 1.2 % other, and had an average age of 46.2 years (SD 12.9, range 21–75).

**Table 2:** Response rate for each panel

Expert panel	Invited	Completed Round 1	Completed Round 2	Completed Round 3	Response Rate
Financial institution staff	112	81	59	54	48.2 %
Financial counsellors	81	43	35	33	40.7 %
Mental health professionals	72	38	31	31	43.1 %
Consumers	38	25	20	20	52.6 %
Carers	37	27	25	24	64.9 %
Total	340	214	170	162	47.7 %

### Endorsed items

**Fig. 2:**
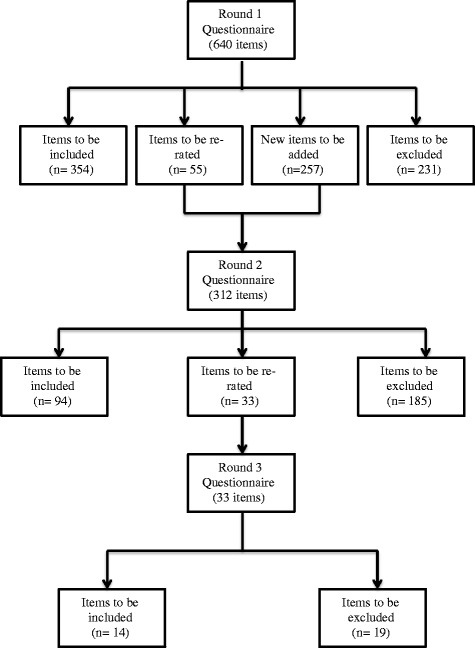
Total number of items rated and results from Rounds 1, 2 and 3

Figure 2 presents the information about the total number of items rated, endorsed and rejected over the three rounds. The three rounds yielded a total of 462 endorsed items (see Additional file 4 for a list of the endorsed items) and 435 rejected items (see Additional file 5 for a list of the rejected items). The endorsed items formed the basis for the guidelines.

There was a strong positive correlation between the panels in the percentage endorsement for whether items should be included in the guidelines. The mental health professional panel and the financial counsellor panel, and the mental health professional panel and the carer panel had the strongest correlations (both r = .89) and the financial institution staff and the carers had the lowest (r = .72).

### Differences between groups

In spite of the strong correlations, there were areas of disagreement, particularly between the financial institution staff panel and the others. Items that were either rejected or endorsed by only one panel and that received notably higher or lower ratings from this panel (±10 %) are noted below.

#### Comparison of ratings by the financial institution staff panel with the other panels

Items that received a lower rating from the financial institution staff panel mainly fell into the following categories:

- Specific procedures the financial institution should have for people with mental health problems, e.g. deferring collection action or suspending interest payments when a person is acutely unwell.
- Passing on information about a customer’s mental health problems to others, e.g. the support person, other financial institutions.
- Whether financial institution staff should refer people with mental health problems to financial counselling services.
- How much knowledge that financial institution staff need about mental health problems.

By contrast, the financial institution staff panel gave a higher rating to the action of financial institution staff and financial counsellors requesting that police check on people when there is a serious concern for safety.

### Comparison of ratings by the financial counsellor panel with the other panels

Items that received lower ratings from the financial counsellor panel included those addressing the support person’s involvement in the management of the person’s financial difficulties. This difference was also reflected in the comments made by participants in Round 1. For example, “Does the support person understand the financial ramifications?” and “Dependent on relationship with support person as to how much information is divulged [to the support person]” and “It is important that the support persons are not exploiting the client and encouraging them to make the wrong decisions.”

One item that received a higher rating from the financial counsellor panel related to getting an assurance from a distressed client with whom they are talking over the phone, that they will not harm themselves.

### Comparison of ratings by the mental health professional panel with the other panels

The one item that received a lower rating from the mental health professional panel related to the mental health professional referring a person with poor money management skills to a financial counsellor.

### Comparison of ratings by the consumer and carer panels with the other panels

Items that received a lower rating from the consumer panel included the support person advising the person to disclose their mental health problems to the financial institution, the financial institution staff and financial counsellor working together to determine the person’s capacity to understand their financial situation, and a number of items about mental health knowledge and skills needed by financial counsellors and financial institution staff.

There were no items that the carer panel rated either higher or lower than the other panels.

### Use of a standardised form

Panel members were asked to rate a number of items relating to the development and use of a standardised form that the mental health professional could complete to communicate how a person’s mental health problems impact on their ability to manage their financial difficulties. Overall, there was support for such a form. Refer to Additional file 1 for the items relating to the standardised form that were endorsed and Additional file 2 for items that were rejected.

### Guidelines development

The first author grouped similar items under specific headings, re-writing them into continuous prose for ease of reading. Original wording of the items was retained, as much as possible. The Working Group reviewed this draft to ensure that the structure and the language were appropriate for the professionals that the guidelines target. The draft guidelines were then given to panel members for final comment, feedback and endorsement. The panel members requested only minor changes.

The final guidelines [45] (see Additional file 6) provide information for professionals and support people on how to assist a person with mental health problems and financial difficulties. The main themes are:

- Knowledge of mental health problems and financial difficulties
- How to support a person with mental health problems and financial difficulties
- Working with other professionals
- Disclosing mental health problems
- Effective communication when working with the person with mental health problems and financial difficulties
- The development and use of a standardised form to enable collaboration between professionals.

## Discussion

This research aimed to develop a consistent approach to working with people with mental health problems and financial difficulties through the expert consensus of financial counsellors, financial institution staff, mental health professionals, consumers and carers. Overall, 462 items were endorsed by all five groups as important or essential to be included in the guidelines. The endorsed items were written into a guidelines document that aims to inform policy and practice. There were a number of findings worthy of further discussion.

### Role delineation

Clear professional role delineation was a consistent theme evident in the data. For instance, while there was endorsement for the financial counsellor to ask about mental health problems, there was limited endorsement for financial counsellors using therapeutic techniques. Conversely, there was endorsement for mental health professionals to ask about financial difficulties, but limited endorsement for them using financial counselling techniques.

Differentiation of roles within the financial institution staff was also evident in the Round 1 survey results. In Round 1, items referred to financial institution staff as a homogenous group, but many did not reach consensus. Comments provided by panel members indicated that some of the statements may be applicable to some roles within the financial institution and not others. For instance:

“I am concerned about the term "financial institution staff" as that covers so many positions…the extent of "how much" would depend on their position and decision making capacity in their organization.”

and

“Asking [about] thoughts of suicide…would not be appropriate for some financial institution staff, e.g. bank teller when the queue is out the door and everyone is listening.”

For this reason a number of the items for Round 2 were re-drafted to differentiate between the roles of hardship, collection, branch and contact centre staff. In Australia, hardship staff are trained to provide financial solutions for people experiencing financial difficulties or hardship, collection staff negotiate payment from people with overdue accounts, branch staff work in the local bank branches and contact centre staff work with customers over the phone. Contact centre staff can be located overseas or in Australia. This redrafting of items to differentiate these roles led to a number of items being endorsed for specific roles.

The guidelines also include a section about what a support person should know and do to help the person with mental health problems and financial difficulties. The financial counsellors, in particular had concerns about the support person’s ability to act in the person’s best interest, either due to ignorance around financial issues or more malicious intent. The consumers also rated an item about the support person providing advice to the person lower than the other panels.

### Specific actions for financial institution staff

A number of items were included in the survey that addressed specific actions that financial institution staff should take to assist the customer with mental health problems and financial difficulties. Some of these items received agreement from all but the financial institution staff. Such differences are likely to stem from the legal and institutional requirements on financial institution staff, which the other panels may not have been aware of. In response to the statements about specific strategies for financial institutions (e.g. suspending interest), one participant said, “System limitations”, while another said, “Financial institutions cannot record or keep sensitive information.” Another commented, “These questions pose a difficulty for me in terms of privacy and yet responsible bank lending for consumers whose finances are not in great shape.” The financial institution staff also commented on the tension created by the need to follow privacy laws and institutional policies, and what they thought would be best for all parties involved:

“I'm not 100 % sure about mental health disclosure due to privacy laws, but knowing a customer’s health issues helps us so much to look at solutions that will assist their financial situation.”

### The use of a standardised form

Panel members were asked to rate a number of items relating to the development and use of a standardised form that the mental health professional could complete to communicate how a person’s mental health problems impact on their ability to manage their financial difficulties. It is envisioned that this form will allow financial counsellors and financial institutions to work collaboratively to find the most appropriate solution to the person’s financial difficulties.

Privacy laws and institutional policies likely influenced the rating of items about the use this form, for instance items relating to recording of information about how the mental health problems affect the person’s ability to manage current financial difficulties received endorsement. However, items asking for detailed information about the diagnosis and treatment of the mental health problem did not.

In addition to the consensus received for the items relating to this form, the researchers also received numerous positive anecdotal comments about the usefulness of such a form. In spite of the consensus and positive feedback, it is expected that the implementation of this form will be complex and difficult given privacy laws and the various institutional policies that govern the management of personal information [46].

### These guidelines compared to other work in Australia and overseas

These guidelines can be compared to other work done in Australia and overseas. In Australia, Good Shepherd Youth and Family Services developed two booklets based on a literature review and interviews with financial counsellors. While the advice in these booklets is generally consistent with the advice in the guidelines, they included a number of pieces of advice that were not endorsed in the current study. Moreover, unlike the current guidelines, these booklets only cover the role of the financial counsellor and mental health professional.

In the UK, the Royal College of Psychiatrists and the Money Advice Trust published a document called *Debt Collection and Mental Health: Ten Steps to Improve Recovery*. The Australian guidelines diverge from this document in several ways. First, the UK document recommends that financial institution staff ask specific questions about how mental health affects the person’s financial situation and their ability to communicate with creditors. Second, they recommend that financial institution staff refer customers to mental health professionals. Items similar to this did not receive endorsement from the panels in this study. This may be because participants thought the actions depended on the specific situation. Furthermore, privacy laws are different in the UK to Australia, which may have implications for the ability to collect and store private information about mental health problems.

Another notable difference from the UK work relates to the type of information to be collected using a standardised form. The UK form requests details about the diagnosis and treatment of the mental health problems. Similar items were included in our Delphi questionnaire, but as stated earlier, were rejected by all panels. The Australian form focuses on the functional impact of the mental health problems on the ability to manage current financial difficulties. Again financial institution staff may have been cautious in wanting to collect detailed information due to the privacy laws in Australia. However, the remaining panels also thought it was not necessary to collect this amount of detail.

### Implementation of the guidelines and potential future work

Until recently, financial counsellors have not had a national framework for training and professional development, which has led to inconsistent training, particularly around working with people with mental health problems [47]. The guidelines for financial counsellors are currently being used to inform mental health training for financial counsellors and financial counselling students. These guidelines are also available as part of a larger suite of mental health first aid guidelines on the Mental Health First Aid Australia website (www.mhfa.com.au). Previous work has shown that people who download these guidelines make practical use of them to help people with mental health problems [48].

Discussions are ongoing with financial institutions and the mental health sector on how best to implement these guidelines, including tailoring of training according to the person’s role. A set of principles for working with and supporting a person who is experiencing mental health problems and financial difficulties is being developed using the guidelines. The principles are broader than the guidelines and will be for the use of financial counsellors, financial institution staff and mental health professionals.

Once the principles are complete and a standardised form is developed, future work will need to be done to better understand and overcome the earlier noted difficulties anticipated in the implementation of these documents. Further research in this area could include an evaluation of the use of the guidelines, principles and standardised form. Future work could also be done to try to get the guidelines and principles incorporated into the existing financial codes of practice. This current project was limited to developing guidelines for people with mental health problems who are already experiencing financial difficulties. Future work is needed to address prevention of financial difficulties by developing guidelines for financial institution staff who make decisions around lending money and providing credit.

### Limitations

Limitations of this study include being able to apply these guidelines consistently within financial institutions, given the legal and systematic constraints highlighted above. Two other limitations are related to the online Delphi process. The first is the possibility that some panel members were asked to advise on statements that were outside their expertise, possibly leading to a lack of inclusion of items related to best-practice evidence. The second limitation is that while participants are able to provide comments in Round 1 of the survey, they are not able to fully discuss their comments and opinions with other experts. Panel members may have made incorrect assumptions and held biases that remained unchallenged because there was no opportunity for discussion. It is possible that key actions were omitted from the guidelines because of this.

## Conclusion

Given the association between mental health problems and financial difficulties, financial counsellors and financial institution staff have an important role to play in the lives of people with both mental health problems and financial difficulties. Financial counsellors, financial institution staff, mental health professionals, consumers and carers were able to reach consensus about a number of strategies for assisting a person with mental health problems and financial difficulties. It is anticipated that these guidelines will be used to inform policy and train financial counsellors and financial institution staff in how to approach and assist their clients and customers in a way that will benefit the person with mental health problems.

## Additional files

Additional file 1:
**Copy of Round 1 survey.**
Additional file 2:
**Copy of Round 2 survey.**
Additional file 3:
**Copy of Round 3 survey.**
Additional file 4:
**Endorsed Items: List of the survey items that were endorsed by all five panels.**
Additional file 5:
**Rejected Items: List of the survey items that were not endorsed by all five panels.**
Additional file 6:
**Guidelines for professionals who work with people with mental health problems and financial difficulties: Final copy (prior to graphic design stage) of the guidelines for financial counsellors, financial institution staff, mental health professionals and carers.**
