# Supplementary material for: Assisting Australians with mental health problems and financial difficulties: a Delphi study to develop guidelines for financial counsellors, financial institution staff, mental health professionals and carers
Source: BMC Health Serv Res. 2015 Jun 3;15:218. doi: 10.1186/s12913-015-0868-2 (PMC4453216; doi:10.1186/s12913-015-0868-2)
Supplement: Additional file 1: — Copy of Round 1 survey. [file 12913_2015_868_MOESM1_ESM.pdf]

Introduction

Purpose of this Research

Studies show a strong association between mental health problems and financial difficulties. In Australia, experiencing financial difficulties is associated with increased risk of depression (Butterworth, Olesen, & Leach, 2012) and is also a risk factor for suicide (Judd, Jackson, Komiti, Bell, & Fraser, 2012). In a survey of people with psychotic disorders, 43% cited financial difficulties as a major challenge in the coming year (Carr, Whiteford, Groves, McGorry, & Shepherd, 2012). Given the strong association between mental health problems and financial difficulties, financial counsellors and financial institution staff need guidance on how best to work with people with mental health problems.

The aim of this project is to develop guidelines to assist financial counsellors and financial institution staff when working with people with mental health problems.

This research project is funded by the Australian Government Department of Health and Ageing, ANZ, GE Capital Finance, National Australia Bank, Westpac and beyondblue.

Information you need to know

Why have I been selected?

You have been selected as a panel member for this study because you have expertise in the field of mental health and financial difficulties (either as a consumer, carer, financial counsellor, financial institution staff or mental health professional).

What do I have to do?

Your task is to rate the statements presented in this questionnaire according to how important you believe they are to working with a person experiencing mental health problems and financial difficulties. When rating the statements, please keep in mind the issues involved in supporting someone with mental health problems and financial difficulties. For example you may be asked to rate the following statement:

1.

The following people should should be aware of how mental health problems may affect financial stability.

	Essential	Important	Don't know/Depends	Unimportant	Should not be included
The financial counsellor	◯	◯	◯	◯	◯
The financial institution staff	◯	◯	◯	◯	◯
The support person	◯	◯	◯	◯	◯

You will then need to decide how important, from 'essential' to 'should not be included', you think it is that this statement be included in the guidelines. You need to answer the question in relation to each of the groups listed.

For an example of previously developed guidelines go to: First aid guidelines for assisting the person who is suicidal.

What if an important statement is missing or if I have something to add?

You will note that there is a place for you to add comments at the end of each section. This is so you can add any missing statements you think are important in working with the person with mental health problems and financial difficulties. These statements will then go into a second round of this survey to be rated by all of the expert panel members.

Where do the statements in the questionnaire come from?

The statements in the following questionnaire were derived from information found online, in books and in academic journal articles. Some of the statements may seem contradictory or controversial; however, we have included them because they reflect the wide range of people's beliefs about policy, services and care. It is important to note that we do not necessarily agree with these statements; we have included them because we do not believe that we should decide what the best practice is in this area. Rather, we want an expert panel to decide this. Therefore, we have invited you to be a member of this expert panel, to help develop guidelines that reflect current expert opinion across the field of mental health problems and financial difficulties.

What is the focus of the questionnaire?

The focus of this research is to increase the financial counsellor's and financial institution staff's knowledge of working with people with mental health problems. Therefore, statements that apply to general financial counselling skills or general financial institution staff duties are not included. Likewise, these guidelines are not intended to help mental health professionals develop skills for working with people with mental health problems. Rather, they are intended to help mental health professionals working with people who also have financial difficulties.

How long will it take to complete the questionnaire?

This questionnaire should take approximately 60 minutes to complete. However, some people may finish it more quickly, while others may take more time, especially if they wish to lodge comments at the end of each section. If necessary, you can complete the survey in two or more sittings. You can save your answers at any time by completing a page and clicking 'Next' at the bottom. This marks your page as complete and you can begin again at a later date on the next page. **Please make sure that you always log back in using the same computer, and that cookies are enabled*, otherwise the software will fail to recognize your code and previously saved responses.**

*see the email you were sent with this link to find out how to enable cookies.

What do I need to do to receive the payment

You must complete all three rounds of the survey.

Consent to Participate in this Research

It is important for you to know that participation in this study is completely voluntary. You are not under any obligation to participate and you can withdraw at any time. Submitting your questionnaire is an indication of your understanding of this and your consent to participate in the study.

We would like to thank you for your time and effort and encourage you to provide us with feedback on this process.

Best Wishes,

The Mental Health First Aid Research Team

Introduction (cont)

Overview of Questionnaire

The questionnaire is divided into the following sections:

Section 1: General awareness about mental health problems and financial difficulties

Section 2: Specific mental health problems

Section 3: Screening and referral

Section 4: Working together

Section 5: The support person

Section 6: Disclosure

Section 7: Documentation

Section 8: Actions to help the person

Section 9: Communication

Section 10: Working with the person over the phone

Section 11: Policy and procedures

Definitions

These terms may have different meaning for the groups represented in this survey (i.e. financial counsellors, financial institution staff, consumers, carers and mental health professionals). When answering these questions, please ensure you use our definitions only.

‘The person’, unless otherwise stated, refers to the person with *mental health problems and financial difficulties*. This person may be called a customer, client, patient, consumer, relative or friend.

'Mental illness' is a diagnosable illness that affects a person's thinking, emotional state and behaviour, and disrupts the person's ability to work or carry out other daily activities and engage in satisfying personal relationships.

‘Mental health problems’ is a broader term including mental illnesses, symptoms of mental illnesses that may not be severe enough to warrant the diagnosis of a mental illness, as well as mental health-related crises such as having thoughts of suicide. For the purposes of this survey, problem gambling has been excluded from mental health problems, as this may be the subject of a future specialised project.

‘Financial difficulties’ are when income is insufficient to cover reasonable living expenses and meet financial commitments as they become due. This definition covers people in financial hardship, as well as those experiencing other financial challenges. This does not include when a payment has simply been overlooked.

‘The support person’ refers to someone who is a significant source of emotional and/or practical support or assistance to 'the person'. It may include a partner, close friend or family member. However, it is not assumed that every person has a support person.

'Authorised third party representative' refers to anyone who has been appointed, in writing or orally, by 'the person' to act on their behalf regarding financial decisions.

‘The financial counsellor’ is a trained professional who assists people who are experiencing financial difficulties. Most financial counsellors work in community organisations, although some work in government agencies and local government. Financial counsellors provide a free, confidential and independent service. The financial counsellor is not a financial planner, financial adviser or legal practitioner and is not a general welfare worker.

'Financial institution' is any institution that is in the position to collect money from individuals, including banks or those who act on their behalf.

'Financial institution staff' are employees of financial institutions that work with the person with mental health problems and financial difficulties.

'Hardship team' is a financial institution's team that is trained to provide financial solutions for people experiencing financial difficulties/hardship.

'Hardship' is defined as when 'the person' is being managed by a financial institution's hardship team.

'Collections staff' are financial institution staff whose primary duty is to negotiate payment from people with overdue accounts.

‘The mental health professional’ refers to a trained health professional who is treating/working with 'the person'. This could be a psychologist, mental health nurse or psychiatrist. It could also be a social worker, occupational therapist or GP who has specialist mental health training.

Information About You

1. Please enter your participant code.

***2. Which best describes your area of expertise?**

- ◯ Financial institution staff
- ◯ Mental health consumer
- ◯ Carer
- ◯ Financial counsellor
- ◯ Mental health professional

Information About You (Consumers)

You must be able to anser 'Yes' to the following quesitions in order to participate in this research.

1. Have you had experience with mental health problems and financial difficulties?

- ◯ Yes
- ◯ No

2. Are both your mental health symptoms and your financial difficulties well managed at the moment?

- ◯ Yes
- ◯ No

3. Are you in a mental health advocacy role?

- ◯ Yes
- ◯ No

Information About You (Carer)

You must be able to anser 'Yes' to the next question in order to participate in this research.

1. Do you currently or have you in the past care/d for a person who experienced mental health problems and financial difficulties?

- ◯ Yes
- ◯ No

Information About You (Financial counsellor)

You must be able to anser 'Yes' to the following questions in order to participate in this research.

1. Do you have at least 2-3 years experience as a financial counsellor or are you a manager of a financial counselling service?

- ◯ Yes
- ◯ No

2. Do you have experience working with people with mental health problems or do you have personal experience with mental health problems (either yourself or someone you know personally)?

- ◯ Yes
- ◯ No

Information About You (Mental health professional)

You must be able to anser 'Yes' to the following questions in order to participate in this research.

1. Do you have at least 2-3 years experience working as a mental health professional?

- ◯ Yes
- ◯ No

2. Have you worked with people with financial difficulties or have you had personal experience with financial difficulties (either yourself or someone you know personally)?

- ◯ Yes
- ◯ No

Information About You (financial institution staff)

You must be able to anser 'Yes' to the following questions in order to participate in this research.

1. Do you have at least 2-3 years experience in or are you a manager of at least one of the following:

collection team

hardship team

complaints team, Or

Work in a job that gives you industry insight into mental health problems and financial difficulties?

- ◯ Yes
- ◯ No

2. Do you have professional or personal experience with mental health problems. (Professional experience is working with customers who have disclosed a mental health problem and personal experience is experiencing mental health problems yourself or someone you know personally.)

◯ Yes ◯ No

Information About You

You must be able to anser 'Yes' to questions 1 and 2 in order to participate in this research.

1. Do you live and, if applicable work in australia?

◯ Yes

◯ No

2. Are you at least 18 years old?

◯ Yes

◯ No

3. What is your gender?

◯ Female

◯ Male

◯ Gender queer

◯ Other

4. What is your age?

5. If applicable, what is your occupation and title?

6. Please name all the relevant organisations you are affiliated with and your role within these organisations, e.g. professional organisations, consumer advocacy groups.

7. Please provide details of your primary place of work, study or residence.

City/Town

State

Section 1: General awareness about mental health problems and financial di...

This section asks you about what information **the financial counsellor** and **the financial institution staff** should know about mental health problems and financial difficulties.

Please rate how important (from 'essential' to 'should not be included') you think it is that each statement be included in the guidelines for each of the groups listed in the left hand column.

Please keep the definitions in mind when answering the questions.

1. The following people should be aware of what mental illness is.

	Essential	Important	Don't know/Depends	Unimportant	Should not be included
Financial counsellor	○	○	○	○	○
Financial institution staff	○	○	○	○	○

2. The following people should be aware of how common mental illnesses are in the community.

	Essential	Important	Don't know/Depends	Unimportant	Should not be included
Financial counsellor	○	○	○	○	○
Financial institution staff	○	○	○	○	○

3. The following people should be aware of the types of mental illnesses.

	Essential	Important	Don't know/Depends	Unimportant	Should not be included
Financial counsellor	○	○	○	○	○
Financial institution staff	○	○	○	○	○

4. The following people should be aware of the signs and symptoms of mental illnesses.

	Essential	Important	Don't know/Depends	Unimportant	Should not be included
Financial counsellor	○	○	○	○	○
Financial institution staff	○	○	○	○	○

5. The following people should be aware of the causes of and risk factors for mental illnesses.

	Essential	Important	Don't know/Depends	Unimportant	Should not be included
Financial counsellor	○	○	○	○	○
Financial institution staff	○	○	○	○	○

6. Do you have any comments on these statements? Is there anything you would like to add to this section? Please write your suggestions in the box provided.

7. The following people should be aware of the range of mental illness treatments and their effects.

	Essential	Important	Don't know/Depends	Unimportant	Should not be included
Financial counsellor	○	○	○	○	○
Financial institution staff	○	○	○	○	○

8. The following people should know the things which might indicate that the person has mental health problems, e.g. not attending appointments, not returning phone calls, not meeting repayment schedules, displaying unusual behaviours, lack of clarity of thought.

	Essential	Important	Don't know/Depends	Unimportant	Should not be included
Financial counsellor	○	○	○	○	○
Financial institution staff	○	○	○	○	○

9. The following people should be aware of the importance of early identification and intervention for preventing or limiting relapse in the person with mental health problems.

	Essential	Important	Don't know/Depends	Unimportant	Should not be included
Financial counsellor	○	○	○	○	○
Financial institution staff	○	○	○	○	○

10. The following people should be aware of how they can support the person with mental health problems in ways that promote recovery.

	Essential	Important	Don't know/Depends	Unimportant	Should not be included
Financial counsellor	○	○	○	○	○
Financial institution staff	○	○	○	○	○

11. Do you have any comments on these statements? Is there anything you would like to add to this section? Please write your suggestions in the box provided.

Section 1 (cont): General awareness about mental health problems and finan...

This section asks you about what information **the financial counsellor** and **the financial institution staff** should know about mental health problems and financial difficulties.

Please rate how important (from 'essential' to 'should not be included') you think it is that each statement be included in the guidelines for each of the groups listed in the left hand column.

Please keep the definitions in mind when answering the questions.

1. The following people should know how they can reduce stressors that may exacerbate the person's mental health problems.

	Essential	Important	Don't know/Depends	Unimportant	Should not be included
Financial counsellor	○	○	○	○	○
Financial institution staff	○	○	○	○	○

2. The following people should be aware of how they can reduce stressors that can contribute to relapse, i.e. a reoccurrence of a mental health problem in a person who is currently well.

	Essential	Important	Don't know/Depends	Unimportant	Should not be included
Financial counsellor	○	○	○	○	○
Financial institution staff	○	○	○	○	○

3. The following people should be aware that the level of support needed by the person with mental health problems will fluctuate, as the symptoms of most mental health problems come and go over time.

	Essential	Important	Don't know/Depends	Unimportant	Should not be included
Financial counsellor	○	○	○	○	○
Financial institution staff	○	○	○	○	○

4. The following people should be aware that the person with mental health problems may not overspend but may have other problems with managing money, e.g. withdrawing all their money from the bank to keep it 'safe' at home.

	Essential	Important	Don't know/Depends	Unimportant	Should not be included
Financial counsellor	○	○	○	○	○
Financial institution staff	○	○	○	○	○

5. The following people should be aware of the impact of the symptoms of mental health problems on the tasks necessary to keep up with financial responsibilities, e.g. problems with memory, decision-making and motivation, or conversely, over-confidence and over-spending in mania.

	Essential	Important	Don't know/Depends	Unimportant	Should not be included
Financial counsellor	◯	◯	◯	◯	◯
Financial institution staff	◯	◯	◯	◯	◯

6. Do you have any comments on these statements? Is there anything you would like to add to this section? Please write your suggestions in the box provided.

7. The following people should be aware that having mental health problems is not necessarily an indicator of an inability to manage money. The effects of mental health problems will differ from one person to another, and may vary over time.

	Essential	Important	Don't know/Depends	Unimportant	Should not be included
Financial counsellor	◯	◯	◯	◯	◯
Financial institution staff	◯	◯	◯	◯	◯

8. The following people should be aware of how they can reduce stressors that can contribute to relapse, i.e. a reoccurrence of a mental health problem in a person who is currently well.

	Essential	Important	Don't know/Depends	Unimportant	Should not be included
Financial counsellor	◯	◯	◯	◯	◯
Financial institution staff	◯	◯	◯	◯	◯

9. The following people should be aware of how the stigmatising attitudes of others can have a negative impact on the person with mental health problems.

	Essential	Important	Don't know/Depends	Unimportant	Should not be included
Financial counsellor	◯	◯	◯	◯	◯
Financial institution staff	◯	◯	◯	◯	◯

10. The following people should be aware of the myths surrounding mental health problems that lead to stigma and limit the potential earning capabilities of the person affected by mental health problems.

	Essential	Important	Don't know/Depends	Unimportant	Should not be included
Financial counsellor	◯	◯	◯	◯	◯
Financial institution staff	◯	◯	◯	◯	◯

11. The following people should be aware of the potential effects of any stigmatising attitudes the financial counsellor or financial institution staff may have on the person with mental health problems.

	Essential	Important	Don't know/Depends	Unimportant	Should not be included
Financial counsellor	◯	◯	◯	◯	◯
Financial institution staff	◯	◯	◯	◯	◯

12. Do you have any comments on these statements? Is there anything you would like to add to this section? Please write your suggestions in the box provided.

Section 1 (cont): General awareness about mental health problems and finan...

This section asks you about what information **the financial counsellor** and **the financial institution staff** should know about mental health problems and financial difficulties.

Please rate how important (from 'essential' to 'should not be included') you think it is that each statement be included in the guidelines for each of the groups listed in the left hand column.

Please keep the definitions in mind when answering the questions.

1. The following people should be aware of the effects of the person's own stigma about their illness on their ability to deal with a financial difficulties.

	Essential	Important	Don't know/Depends	Unimportant	Should not be included
Financial counsellor	○	○	○	○	○
Financial institution staff	○	○	○	○	○

2. The following people should know how to communicate with a person who is very distressed.

	Essential	Important	Don't know/Depends	Unimportant	Should not be included
Financial counsellor	○	○	○	○	○
Financial institution staff	○	○	○	○	○

3. The following people should know how to communicate with a person who appears to be behaving aggressively.

	Essential	Important	Don't know/Depends	Unimportant	Should not be included
Financial counsellor	○	○	○	○	○
Financial institution staff	○	○	○	○	○

4. The following people should be aware of the mental health and disability support services available in the community.

	Essential	Important	Don't know/Depends	Unimportant	Should not be included
Financial counsellor	○	○	○	○	○
Financial institution staff	○	○	○	○	○

5. The following people should be aware of the internal and external resources available to them should they feel distressed by their work with people with mental health problems.

	Essential	Important	Don't know/Depends	Unimportant	Should not be included
Financial counsellor	○	○	○	○	○
Financial institution staff	○	○	○	○	○

6. Do you have any comments on these statements? Is there anything you would like to add to this section? Please write your suggestions in the box provided.

Section 1 (cont): General awareness about mental health problems and finan...

This section asks you about what information **the financial counsellor, the financial institution staff, the mental health professional** and **the suport person** should know about mental health problems and financial difficulties.

Please rate how important (from 'essential' to 'should not be included' or 'this is not my area of expertise') you think it is that each statement be included in the guidelines for each of the groups listed in the left hand column.

Please keep the definitions in mind when answering the questions.

1. The following people should know that the cycle of financial difficulties and mental health problems can be intensified for people from culturally and linguistically diverse communities, due to the difficulty of navigating unfamiliar financial and health systems in Australia.

	Essential	Important	Don't know/Depends	Unimportant	Should not be included
Financial counsellor	○	○	○	○	○
Financial institution staff	○	○	○	○	○
Mental health professional	○	○	○	○	○

2. The following people should be aware of what a financial administration order is and the implications for the client.

	Essential	Important	Don't know/Depends	Unimportant	Should not be included
Financial counsellor	○	○	○	○	○
Financial institution staff	○	○	○	○	○
Mental health professional	○	○	○	○	○

3. Do you have any comments on these statements? Is there anything you would like to add to this section? Please write your suggestions in the box provided.

4. The following people should know the relevant laws that affect interaction between the person with mental health problems and creditors, e.g. Disability Discrimination Act 1992, the Social Security Act, 1991.

	Essential	Important	Don't know/Depends	Unimportant	Should not be included
Financial counsellor	○	○	○	○	○
Mental health professional	○	○	○	○	○

5. The following people should know their state or territory's mental health legislation and the implications for their responsibility in responding to the person's mental health crisis.

	Essential	Important	Don't know/Depends	Unimportant	Should not be included
Financial counsellor	○	○	○	○	○
Mental health professional	○	○	○	○	○

6. Do you have any comments on these statements? Is there anything you would like to add to this section? Please write your suggestions in the box provided.

7. The following people should know of the positive role that financial institutions can play for people with mental health problems and financial difficulties, e.g. promoting financial inclusion.

	Essential	Important	Don't know/Depends	Unimportant	Should not be included
Financial counsellor	○	○	○	○	○
Mental health professional	○	○	○	○	○
Support person	○	○	○	○	○

8. The following people should know that financial difficulties may negatively impact the person's ability to be a part of their community.

	Essential	Important	Don't know/Depends	Unimportant	Should not be included
Financial counsellor	○	○	○	○	○
Mental health professional	○	○	○	○	○
Support person	○	○	○	○	○

9. The following people should know that most financial institutions will have policies about assistance for people who are experiencing financial difficulties.

	Essential	Important	Don't know/Depends	Unimportant	Should not be included
Financial counsellor	○	○	○	○	○
Mental health professional	○	○	○	○	○
Support person	○	○	○	○	○

10. Do you have any comments on these statements? Is there anything you would like to add to this section? Please write your suggestions in the box provided.

11. The following people should know the important role that financial counselling services play for people with mental health problems and financial difficulties.

	Essential	Important	Don't know/Depends	Unimportant	Should not be included
Financial institution staff	○	○	○	○	○
Mental health professional	○	○	○	○	○
Support person	○	○	○	○	○

12. The following people should know that many people have limited financial literacy skills.

	Essential	Important	Don't know/Depends	Unimportant	Should not be included
Financial institution staff	○	○	○	○	○
Mental health professional	○	○	○	○	○
Support person	○	○	○	○	○

13. Do you have any comments on these statements? Is there anything you would like to add to this section? Please write your suggestions in the box provided.

Section 1 (cont): General awareness about mental health problems and finan...

This section asks you about what information **the financial counsellor, the financial institution staff, the mental health professional**, and **the support person** should know about mental health problems and financial difficulties.

Please rate how important (from 'essential' to 'should not be included' or 'this is not my area of expertise') you think it is that each statement be included in the guidelines for each of the groups listed in the left hand column.

Please keep the definitions in mind when answering the questions.

1. The following people should be aware that mental health problems can contribute to financial difficulties, and vice versa, e.g. failing to pay bills because of depression, loss of income increasing anxiety and depression.

	Essential	Important	Don't know/Depends	Unimportant	Should not be included
Financial counsellor	○	○	○	○	○
Financial institution staff	○	○	○	○	○
Mental health professional	○	○	○	○	○
Support person	○	○	○	○	○

2. The following people should know that overspending and financial difficulties can be a sign of a relapse of the person's mental health problems.

	Essential	Important	Don't know/Depends	Unimportant	Should not be included
Financial counsellor	○	○	○	○	○
Financial institution staff	○	○	○	○	○
Mental health professional	○	○	○	○	○
Support person	○	○	○	○	○

3. The following people should know that the person with mental health problems may have increased medical expenses that limit financial resources.

	Essential	Important	Don't know/Depends	Unimportant	Should not be included
Financial counsellor	○	○	○	○	○
Financial institution staff	○	○	○	○	○
Mental health professional	○	○	○	○	○
Support person	○	○	○	○	○

4. The following people should know that the person with mental health problems may incur debt to support a drug or alcohol problem.

	Essential	Important	Don't know/Depends	Unimportant	Should not be included
Financial counsellor	○	○	○	○	○
Financial institution staff	○	○	○	○	○
Mental health professional	○	○	○	○	○
Support person	○	○	○	○	○

5. The following people should know that the person with financial difficulties may find it difficult to pay for treatment for their mental health problems, which may worsen their condition.

	Essential	Important	Don't know/Depends	Unimportant	Should not be included
Financial counsellor	○	○	○	○	○
Financial institution staff	○	○	○	○	○
Mental health professional	○	○	○	○	○
Support person	○	○	○	○	○

6. Do you have any comments on these statements? Is there anything you would like to add to this section? Please write your suggestions in the box provided.

7. The following people should know that financial stability may not be an important goal for some people in the early stages of recovery from mental illness.

	Essential	Important	Don't know/Depends	Unimportant	Should not be included
Financial counsellor	○	○	○	○	○
Financial institution staff	○	○	○	○	○
Mental health professional	○	○	○	○	○
Support person	○	○	○	○	○

8. The following people should know that the person who is making their repayments may still be experiencing financial difficulties and feeling distress.

	Essential	Important	Don't know/Depends	Unimportant	Should not be included
Financial counsellor	◯	◯	◯	◯	◯
Financial institution staff	◯	◯	◯	◯	◯
Mental health professional	◯	◯	◯	◯	◯
Support person	◯	◯	◯	◯	◯

9. The following people should know that the way the person perceives their financial situation is what impacts on their mental health, rather than their actual situation.

	Essential	Important	Don't know/Depends	Unimportant	Should not be included
Financial counsellor	◯	◯	◯	◯	◯
Financial institution staff	◯	◯	◯	◯	◯
Mental health professional	◯	◯	◯	◯	◯
Support person	◯	◯	◯	◯	◯

10. The following people should know that addressing financial difficulties early may reduce the negative impact on mental health.

	Essential	Important	Don't know/Depends	Unimportant	Should not be included
Financial counsellor	◯	◯	◯	◯	◯
Financial institution staff	◯	◯	◯	◯	◯
Mental health professional	◯	◯	◯	◯	◯
Support person	◯	◯	◯	◯	◯

11. The following people should know that the process of debt recovery may have an impact on the person's mental health, e.g. harassment from debt collectors.

	Essential	Important	Don't know/Depends	Unimportant	Should not be included
Financial counsellor	◯	◯	◯	◯	◯
Financial institution staff	◯	◯	◯	◯	◯
Mental health professional	◯	◯	◯	◯	◯
Support person	◯	◯	◯	◯	◯

12. Do you have any comments on these statements? Is there anything you would like to add to this section? Please write your suggestions in the box provided.

13. <u>The financial counsellor</u> should be aware that mental health problems may take time, usually weeks or months, to develop.

Essential	Important	Don't know/Depends	Unimportant	Should not be included
○	○	○	○	○

14. <u>The financial counsellor</u> should be aware that mental health problems can make it difficult for the person to communicate with creditors.

Essential	Important	Don't know/Depends	Unimportant	Should not be included
○	○	○	○	○

15. Do you have any comments on these statements? Is there anything you would like to add to this section? Please write your suggestions in the box provided.

Section 1 (cont): General awareness about mental health problems and finan...

This section asks you about what information **the financial counsellor** and **the financial institution** should know about mental health problems and financial difficulties.

Please rate how important (from 'essential' to 'should not be included' or 'this is not my area of expertise') you think it is that each statement be included in the guidelines for each of the groups listed in the left hand column.

Please keep the definitions in mind when answering the questions.

1. The financial counsellor should be familiar with the following mental health first aid guidelines:

	Essential	Important	Don't know/Depends	Unimportant	Should not be included
Depression	○	○	○	○	○
Suicidal thoughts and behaviours	○	○	○	○	○
Panic attack	○	○	○	○	○
Traumatic events	○	○	○	○	○
Problem drug use	○	○	○	○	○
Problem drinking	○	○	○	○	○
Eating disorders	○	○	○	○	○
Non-suicidal self-injury	○	○	○	○	○
Psychosis	○	○	○	○	○

2. The financial counsellor should be familiar with the cultural considerations of working with the person from a culturally and linguistically diverse background.

Essential	Important	Don't know/Depends	Unimportant	Should not be included
○	○	○	○	○

3. Do you have any comments on these statements? Is there anything you would like to add to this section? Please write your suggestions in the box provided.

4. <u>The financial institution staff who are in customer facing roles</u> should be familiar with the following mental health first aid guidelines:

	Essential	Important	Don't know/Depends	Unimportant	Should not be included
Depression	◯	◯	◯	◯	◯
Suicidal thoughts and behaviours	◯	◯	◯	◯	◯
Panic attack	◯	◯	◯	◯	◯
Traumatic events	◯	◯	◯	◯	◯
Problem drug use	◯	◯	◯	◯	◯
Problem drinking	◯	◯	◯	◯	◯
Eating disorders	◯	◯	◯	◯	◯
Non-suicidal self-injury	◯	◯	◯	◯	◯
Psychosis	◯	◯	◯	◯	◯

5. <u>The financial institution staff who are in customer facing roles</u> should be familiar with the cultural considerations of working with the person from a culturally and linguistically diverse background.

Essential	Important	Don't know/Depends	Unimportant	Should not be included
◯	◯	◯	◯	◯

6. Do you have any comments on these statements? Is there anything you would like to add to this section? Please write your suggestions in the box provided.

Section 2: Specific mental health problems

This section asks you about what information **the financial counsellor, the financial institution, the mental health professional** and **the support person** should know about specific mental health problems and financial difficulties.

Please rate how important (from 'essential' to 'should not be included') you think it is that each statement be included in the guidelines.

Please keep the definitions in mind when answering the questions.

Suicide

1. The following people should be aware of the warning signs of suicide.

	Essential	Important	Don't know/Depends	Unimportant	Should not be included
Financial counsellor	◯	◯	◯	◯	◯
Financial institution staff	◯	◯	◯	◯	◯
Support person	◯	◯	◯	◯	◯

2. The following people should be aware of the risk factors for suicide.

	Essential	Important	Don't know/Depends	Unimportant	Should not be included
Financial counsellor	◯	◯	◯	◯	◯
Financial institution staff	◯	◯	◯	◯	◯
Support person	◯	◯	◯	◯	◯

3. The following people should be aware of the First Aid Guidelines for Assisting the Person Who is Suicidal.

	Essential	Important	Don't know/Depends	Unimportant	Should not be included
Financial counsellor	◯	◯	◯	◯	◯
Financial institution staff	◯	◯	◯	◯	◯
Support person	◯	◯	◯	◯	◯

4. If the person appears to have symptoms of mental illness, the following people should ask them if they are having thoughts of suicide.

	Essential	Important	Don't know/Depends	Unimportant	Should not be included
Financial counsellor	◯	◯	◯	◯	◯
Financial institution staff	◯	◯	◯	◯	◯
Support person	◯	◯	◯	◯	◯

5. The following people should be aware that when a person sees no way back from their financial difficulties they might become suicidal/attempt suicide.

	Essential	Important	Don't know/Depends	Unimportant	Should not be included
Financial counsellor	○	○	○	○	○
Financial institution staff	○	○	○	○	○
Mental health professional	○	○	○	○	○
Support person	○	○	○	○	○

6. Do you have any comments on these statements? Is there anything you would like to add to this section? Please write your suggestions in the box provided.

7. <u>The financial counsellor</u> should routinely ask *<u>new</u>* clients if they are having thoughts of suicide.

Essential	Important	Don't know/Depends	Unimportant	Should not be included	This is not my area of expertise
○	○	○	○	○	○

8. <u>The financial counsellor</u> should regularly ask *<u>all</u>* clients if they are having thoughts of suicide.

Essential	Important	Don't know/Depends	Unimportant	Should not be included
○	○	○	○	○

9. <u>The mental health professional</u> should routinely ask the person with financial difficulties if they are having thoughts of suicide.

Essential	Important	Don't know/Depends	Unimportant	Should not be included
○	○	○	○	○

10. Do you have any comments on these statements? Is there anything you would like to add to this section? Please write your suggestions in the box provided.

Section 2 (cont.): Specific mental health problems

This section asks you about what information **the financial counsellor, the financial institution, the mental health professional** and **the support person** should know about specific mental health problems and financial difficulties.

Please rate how important (from 'essential' to 'should not be included') you think it is that each statement be included in the guidelines.

Please keep the definitions in mind when answering the questions.

Bipolar disorder

1. The following people should encourage the person with bipolar disorder to consider setting up a bank account that requires a second signature for large purchases.

	Essential	Important	Don't know/Depends	Unimportant	Should not be included
Financial counsellor	◯	◯	◯	◯	◯
Financial institution staff	◯	◯	◯	◯	◯
Mental health professional	◯	◯	◯	◯	◯
Support person	◯	◯	◯	◯	◯

2. The following people should be aware that people with bipolar disorder are more vulnerable to problem gambling.

	Essential	Important	Don't know/Depends	Unimportant	Should not be included
Financial counsellor	◯	◯	◯	◯	◯
Financial institution staff	◯	◯	◯	◯	◯
Mental health professional	◯	◯	◯	◯	◯
Support person	◯	◯	◯	◯	◯

3. The following people should be aware of how an episode of mania may affect a person's financial circumstances, e.g. not paying bills, job instability, over-spending.

	Essential	Important	Don't know/Depends	Unimportant	Should not be included
Financial counsellor	◯	◯	◯	◯	◯
Financial institution staff	◯	◯	◯	◯	◯
Mental health professional	◯	◯	◯	◯	◯
Support person	◯	◯	◯	◯	◯

4. Do you have any comments on these statements? Is there anything you would like to add to this section? Please write your suggestions in the box provided.

5. The following people should encourage the person with bipolar disorder to delay making large purchases until they have discussed them with their support person.

	Essential	Important	Don't know/Depends	Unimportant	Should not be included
Financial counsellor	◯	◯	◯	◯	◯
Mental health professional	◯	◯	◯	◯	◯
Support person	◯	◯	◯	◯	◯

6. The following people should advise the person with bipolar disorder to avoid gambling altogether.

	Essential	Important	Don't know/Depends	Unimportant	Should not be included
Financial counsellor	◯	◯	◯	◯	◯
Mental health professional	◯	◯	◯	◯	◯
Support person	◯	◯	◯	◯	◯

7. If <u>the mental health professional</u> has identified overspending as being a feature of the person's bipolar disorder, they should ask about financial difficulties.

Essential	Important	Don't know/Depends	Unimportant	Should not be included
◯	◯	◯	◯	◯

8. Do you have any comments on these statements? Is there anything you would like to add to this section? Please write your suggestions in the box provided.

Depression (includes depressive episode associated with bipolar disorder)

9. The following people should be aware of how depression may affect a person's financial circumstances, e.g. ignoring bills, job loss, over-spending to feel better.

	Essential	Important	Don't know/Depends	Unimportant	Should not be included
Financial counsellor	◯	◯	◯	◯	◯
Financial institution staff	◯	◯	◯	◯	◯
Mental health professional	◯	◯	◯	◯	◯
Support person	◯	◯	◯	◯	◯

10. Do you have any comments on these statements? Is there anything you would like to add to this section? Please write your suggestions in the box provided.

Section 3: Screening and referral

This section asks you about what information **the financial counsellor, financial institution staff,** and **the support person** needs to know in order to refer the person to mental health services, and **the mental health professional, the financial institution staff** and **the support person** needs to know in order to refer the person to financial counselling services.

Please rate how important (from 'essential' to 'should not be included') you think it is that each statement be included in the guidelines.

Please keep the definitions in mind when answering the questions.

Screening

On this page the term 'client' refers to all the people a financial counsellor works with and 'the person' is the client with mental health problems and financial difficulties.

1. The financial counsellor should routinely ask *new* clients if they have *ever* been diagnosed with a mental illness.

Essential	Important	Don't know/Depends	Unimportant	Should not be included
○	○	○	○	○

2. The financial counsellor should routinely ask *new* clients about *current symptoms* of mental health problems.

Essential	Important	Don't know/Depends	Unimportant	Should not be included
○	○	○	○	○

3. The financial counsellor should regularly ask *all* clients about current symptoms of mental health problems.

Essential	Important	Don't know/Depends	Unimportant	Should not be included
○	○	○	○	○

4. The financial counsellor should routinely screen for mental health problems in *new* clients by using a standardised screening questionnaire, e.g. a simple screening tool that is used to measure psychological distress.

Essential	Important	Don't know/Depends	Unimportant	Should not be included
○	○	○	○	○

5. The financial counsellor should regularly screen for mental health problems in *all* clients by using a standardised screening questionnaire, e.g. a simple screening tool that is used to measure psychological distress.

Essential	Important	Don't know/Depends	Unimportant	Should not be included
○	○	○	○	○

6. Do you have any comments on these statements? Is there anything you would like to add to this section? Please write your suggestions in the box provided.

7. If the person appears to have symptoms of mental health problems, <u>the financial counsellor</u> should ask if they have a diagnosis of mental illness.

Essential	Important	Don't know/Depends	Unimportant	Should not be included
○	○	○	○	○

8. If the person appears to have symptoms of mental health problems, <u>the financial counsellor</u> should administer a standardised screening questionnaire, e.g. a simple screening tool that is used to measure psychological distress.

Essential	Important	Don't know/Depends	Unimportant	Should not be included
○	○	○	○	○

9. If the person appears to have symptoms of mental health problems, <u>the financial counsellor</u> should ask about current mental health treatment.

Essential	Important	Don't know/Depends	Unimportant	Should not be included
○	○	○	○	○

10. Do you have any comments on these statements? Is there anything you would like to add to this section? Please write your suggestions in the box provided.

Section 3 (cont): Screening and referral

This section asks you about what information **the financial counsellor, financial institution staff,** and **the support person** needs to know in order to refer the person to mental health services, and **the mental health professional, the financial institution staff** and **the support person** needs to know in order to refer the person to financial counselling services.

Please rate how important (from 'essential' to 'should not be included') you think it is that each statement be included in the guidelines.

Please keep the definitions in mind when answering the questions.

1. The following people should ask the person about mental health problems in any family members who have contributed to financial difficulties, e.g. overspending due to mania or gambling problems.

	Essential	Important	Don't know/Depends	Unimportant	Should not be included
Financial counsellor	○	○	○	○	○
Financial institution staff	○	○	○	○	○

2. The following people should ask direct questions about the person's financial situation, and whether they have any financial difficulties, so they can refer the person for appropriate advice if necessary.

	Essential	Important	Don't know/Depends	Unimportant	Should not be included
Mental health professional	○	○	○	○	○
Support person	○	○	○	○	○

3. The following people should discourage the person from ignoring their financial difficulties.

	Essential	Important	Don't know/Depends	Unimportant	Should not be included
Mental health professional	○	○	○	○	○
Support person	○	○	○	○	○

4. Do you have any comments on these statements? Is there anything you would like to add to this section? Please write your suggestions in the box provided.

Section 3 (cont): Screening and referral

This section asks you about what information **the financial counsellor, financial institution staff,** and **the support person** needs to know in order to refer the person to mental health services, and **the mental health professional, the financial institution staff** and **the support person** needs to know in order to refer the person to financial counselling services.

Please rate how important (from 'essential' to 'should not be included') you think it is that each statement be included in the guidelines.

Please keep the definitions in mind when answering the questions.

Referral

On this page the term 'client' refers to all the people a financial counsellor works with and 'the person' is the client with mental health problems and financial difficulties.

1. If the person appears to have mental health problems for which they are not receiving treatment, the financial counsellor should advise them to seek professional mental health care.

Essential	Important	Don't know/Depends	Unimportant	Should not be included
◯	◯	◯	◯	◯

2. The financial counsellor should *encourage* the person with mental health problems to make their own appointment with a mental health service.

Essential	Important	Don't know/Depends	Unimportant	Should not be included
◯	◯	◯	◯	◯

3. The financial counsellor should *help* the person with mental health problems make an appointment with a mental health service.

Essential	Important	Don't know/Depends	Unimportant	Should not be included
◯	◯	◯	◯	◯

4. The financial counsellor should *make* the appointment with the mental health service for the person with mental health problems.

Essential	Important	Don't know/Depends	Unimportant	Should not be included
◯	◯	◯	◯	◯

5. If the person does not wish to seek the help of a mental health professional, the financial counsellor should explore the pros and cons of seeing a mental health professional with them.

Essential	Important	Don't know/Depends	Unimportant	Should not be included
◯	◯	◯	◯	◯

6. Do you have any comments on these statements? Is there anything you would like to add to this section? Please write your suggestions in the box provided.

7. <u>The financial counsellor</u> should encourage the person to seek professional mental health care for any family member whose illness is contributing to financial difficulties in the family.

Essential	Important	Don't know/Depends	Unimportant	Should not be included
○	○	○	○	○

8. <u>The financial counsellor</u> should know what mental health services are available to their clients.

Essential	Important	Don't know/Depends	Unimportant	Should not be included
○	○	○	○	○

9. <u>The financial counsellor</u> should provide clients with printed material about local mental health services.

Essential	Important	Don't know/Depends	Unimportant	Should not be included
○	○	○	○	○

10. <u>The financial counsellor</u> should display posters or other information about mental health services in their offices.

Essential	Important	Don't know/Depends	Unimportant	Should not be included
○	○	○	○	○

11. Do you have any comments on these statements? Is there anything you would like to add to this section? Please write your suggestions in the box provided.

Section 3 (cont): Screening and referral

This section asks you about what information **the financial counsellor, financial institution staff,** and **the support person** needs to know in order to refer the person to mental health services, and **the mental health professional, the financial institution staff** and **the support person** needs to know in order to refer the person to financial counselling services.

Please rate how important (from 'essential' to 'should not be included') you think it is that each statement be included in the guidelines.

Please keep the definitions in mind when answering the questions.

1. If the financial institution staff suspect that the person in financial difficulties may have mental health problems, they should refer them to financial counselling services, who can advocate on the person's behalf and make an appropriate referral.

Essential	Important	Don't know/Depends	Unimportant	Should not be included
○	○	○	○	○

2. If the financial institution staff are told by the person in financial difficulties that they have mental health problems, they should refer them to financial counselling services, who can advocate on the person's behalf and make an appropriate referral.

Essential	Important	Don't know/Depends	Unimportant	Should not be included
○	○	○	○	○

3. If the financial institution staff suspect that the person in financial hardship may have mental health problems, they should refer them to financial counselling services, who can advocate on the person's behalf and make an appropriate referral. ('Hardship' is defined as when 'the person' is being managed by a financial institution's hardship team.)

Essential	Important	Don't know/Depends	Unimportant	Should not be included
○	○	○	○	○

4. If the financial institution staff are told by the person in financial hardship that they have mental health problems, they should refer them to financial counselling services, who can advocate on the person's behalf and make an appropriate referral.

Essential	Important	Don't know/Depends	Unimportant	Should not be included
○	○	○	○	○

5. If the financial institution staff suspects the person has mental health problems, they should refer the person to the hardship team.

Essential	Important	Don't know/Depends	Unimportant	Should not be included
○	○	○	○	○

6. If the person is being managed by a hardship team, that team should have the discretion to remove the account from any automated collection processes.

Essential	Important	Don't know/Depends	Unimportant	Should not be included
○	○	○	○	○

7. If a hardship team member suspects the person has mental health problems, they should encourage them to seek help from a mental health professional.

Essential	Important	Don't know/Depends	Unimportant	Should not be included
○	○	○	○	○

8. Do you have any comments on these statements? Is there anything you would like to add to this section? Please write your suggestions in the box provided.

Section 3 (cont): Screening and referral

This section asks you about what information **the financial counsellor, financial institution staff,** and **the support person** needs to know in order to refer the person to mental health services, and **the mental health professional, the financial institution staff** and **the support person** needs to know in order to refer the person to financial counselling services.

Please rate how important (from 'essential' to 'should not be included') you think it is that each statement be included in the guidelines.

Please keep the definitions in mind when answering the questions.

1. The mental health professional should know what financial counselling services are available in their area and how to access them.

Essential	Important	Don't know/Depends	Unimportant	Should not be included
○	○	○	○	○

2. The mental health professional should provide the person with printed material about local financial counselling services.

Essential	Important	Don't know/Depends	Unimportant	Should not be included
○	○	○	○	○

3. The mental health professional should display signs about financial counselling services in their offices.

Essential	Important	Don't know/Depends	Unimportant	Should not be included
○	○	○	○	○

4. The mental health professional should provide the person with oral information about financial counselling services in their area.

Essential	Important	Don't know/Depends	Unimportant	Should not be included
○	○	○	○	○

5. The mental health professional should refer the person with poor money management skills to a financial counsellor.

Essential	Important	Don't know/Depends	Unimportant	Should not be included
○	○	○	○	○

6. The mental health professional should explain what financial counsellors do and the potential benefits to the person.

Essential	Important	Don't know/Depends	Unimportant	Should not be included
○	○	○	○	○

7. Do you have any comments on these statements? Is there anything you would like to add to this section? Please write your suggestions in the box provided.

8. <u>The mental health professional</u> should tell the person that dealing with financial difficulties sooner rather than later may make it more manageable.

Essential	Important	Don't know/Depends	Unimportant	Should not be included
○	○	○	○	○

9. If the person appears to have financial difficulties, <u>the mental health professional</u> should <u>*advise them to seek the help*</u> of a financial counsellor.

Essential	Important	Don't know/Depends	Unimportant	Should not be included
○	○	○	○	○

10. If the person appears to have financial difficulties, <u>the mental health professional</u> should <u>*make a referral*</u> to a financial counsellor.

Essential	Important	Don't know/Depends	Unimportant	Should not be included
○	○	○	○	○

11. <u>The mental health professional</u> should help the person prepare for their first appointment with a financial counsellor by helping them gather relevant paperwork or bills.

Essential	Important	Don't know/Depends	Unimportant	Should not be included
○	○	○	○	○

12. <u>The mental health professional</u> and the person should agree on what information will be provided to the financial counsellor concerning how the person's mental health problems affect their ability to manage money or repay their debt.

Essential	Important	Don't know/Depends	Unimportant	Should not be included
○	○	○	○	○

13. If the person is unwilling or unable to talk to a financial counsellor, the mental health professional should:

	Essential	Important	Don't know/Depends	Unimportant	Should not be included
Assist the person to identify priority debts (e.g. debts that can lead to loss of home, imprisonment or disconnection of utilities) so that the person or the mental health professional can contact those creditors immediately.	○	○	○	○	○
Offer to accompany them to appointments with a financial counsellor.	○	○	○	○	○
Seek the advice of a financial counsellor themselves on behalf of the person.	○	○	○	○	○

14. Do you have any comments on these statements? Is there anything you would like to add to this section? Please write your suggestions in the box provided.

Section 4: Working together

This section asks you about what information **the financial counsellor** and **mental health professional** should know about how to work together to get the best outcome for the person with mental health problems and financial difficulties.

Please rate how important (from 'essential' to 'should not be included') you think it is that each statement be included in the guidelines.

Please keep the definitions in mind when answering the questions.

1. The financial counsellor should work with the mental health professional to determine whether the person is able to make specific decisions concerning their current financial situation.

Essential	Important	Don't know/Depends	Unimportant	Should not be included
○	○	○	○	○

2. If the mental health professional has provided advice that the person is unable to make specific decisions concerning their current financial situation, this advice should be reviewed on a regular basis or when the person's mental condition changes significantly.

Essential	Important	Don't know/Depends	Unimportant	Should not be included
○	○	○	○	○

3. The mental health professional should follow-up with the financial counsellor regarding the person's progress, with the person's informed consent.

Essential	Important	Don't know/Depends	Unimportant	Should not be included
○	○	○	○	○

4. The mental health professional should follow-up with the person regarding the progress they are making with the financial counsellor.

Essential	Important	Don't know/Depends	Unimportant	Should not be included	This is not my area of expertise
○	○	○	○	○	○

5. Do you have any comments on these statements? Is there anything you would like to add to this section? Please write your suggestions in the box provided.

6. <u>The financial counsellor and the mental health professional</u> should work together so that the financial plans complement the treatment plan.

Essential	Important	Don't know/Depends	Unimportant	Should not be included
○	○	○	○	○

7. <u>The financial counsellor and the mental health professional</u> should work together and deliver consistent messages to the person.

Essential	Important	Don't know/Depends	Unimportant	Should not be included
○	○	○	○	○

8. <u>The financial counsellor and the mental health professional</u> should be aware of and discuss any conflicting priorities between financial and treatment goals.

Essential	Important	Don't know/Depends	Unimportant	Should not be included	This is not my area of expertise
○	○	○	○	○	○

9. <u>The financial counsellor and the mental health professional</u> should communicate regularly about the person they are both working with, with that person's permission.

Essential	Important	Don't know/Depends	Unimportant	Should not be included
○	○	○	○	○

10. Do you have any comments on these statements? Is there anything you would like to add to this section? Please write your suggestions in the box provided.

11. Where possible, <u>the mental health professional</u> should offer the opportunity for the financial counsellor to meet with the person at the mental health services premises.

Essential	Important	Don't know/Depends	Unimportant	Should not be included
○	○	○	○	○

12. Where possible, <u>the financial counsellor</u> should offer the opportunity for the mental health professional to meet with the person on the financial counselling premises.

Essential	Important	Don't know/Depends	Unimportant	Should not be included
○	○	○	○	○

13. <u>The financial counsellor, mental health professional and the support person</u> should work with the person to determine what specific supports the person needs to enable them to make particular financial decisions.

Essential	Important	Don't know/Depends	Unimportant	Should not be included
○	○	○	○	○

14. Do you have any comments on these statements? Is there anything you would like to add to this section? Please write your suggestions in the box provided.

Section 5: The support person

This section asks you about what information **the financial counsellor** and **the support person** should know about effectively supporting the person with mental health problems and financial difficulties.

Please rate how important (from 'essential' to 'should not be included') you think it is that each statement be included in the guidelines.

When answering the questions please keep the definitions in mind and that 'the support person' may also be 'the authorised third party representative'.

1. The financial counsellor should be aware that the person's financial decisions may be influenced by their support person.

Essential	Important	Don't know/Depends	Unimportant	Should not be included
○	○	○	○	○

2. The financial counsellor should consider working with the person's support person, with the person's permission.

Essential	Important	Don't know/Depends	Unimportant
○	○	○	○

3. The financial counsellor should consider working with the person's support person, with the person present.

Essential	Important	Don't know/Depends	Unimportant
○	○	○	○

4. The financial counsellor should encourage the person to discuss their financial difficulties with their support person.

Essential	Important	Don't know/Depends	Unimportant	Should not be included
○	○	○	○	○

5. The financial counsellor should encourage the person to seek the support of their close family or friends in managing financial difficulties.

Essential	Important	Don't know/Depends	Unimportant	Should not be included
○	○	○	○	○

6. The financial counsellor should advise the person that their family and friends are under no obligation to provide financial support.

Essential	Important	Don't know/Depends	Unimportant	Should not be included
○	○	○	○	○

7. The financial counsellor should encourage the person to form a 'support network' of people who would be helpful in a variety of situations, e.g. when emotional support is needed, when there is an increased risk of overspending or when they are short of money.

Essential	Important	Don't know/Depends	Unimportant	Should not be included
○	○	○	○	○

8. Do you have any comments on these statements? Is there anything you would like to add to this section? Please write your suggestions in the box provided.

9. The support person should *offer* the person practical help with financial matters, e.g. assisting with budgeting, setting up automatic bill payments and attending appointments with the financial institution and the financial counsellor.

Essential	Important	Don't know/Depends	Unimportant	Should not be included
○	○	○	○	○

10. The support person should proactively *provide* the person with practical help with financial matters, e.g. assisting with budgeting, setting up automatic bill payments, attending appointments with banking staff and the financial counsellor.

Essential	Important	Don't know/Depends	Unimportant	Should not be included
○	○	○	○	○

11. The support person should be aware of the financial behaviours that may indicate the person is becoming unwell.

Essential	Important	Don't know/Depends	Unimportant	Should not be included
○	○	○	○	○

12. The support person should reassure the person that there is no such thing as unsolvable financial difficulties.

Essential	Important	Don't know/Depends	Unimportant	Should not be included
○	○	○	○	○

13. Do you have any comments on these statements? Is there anything you would like to add to this section? Please write your suggestions in the box provided.

Section 6: Disclosure

This section asks you about what information **the financial counsellor, mental health professional** and **support person** should know about disclosing mental health problems to a financial institution.

Please rate how important (from 'essential' to 'should not be included') you think it is that each statement be included in the guidelines.

When answering the questions please keep the definitions in mind and that 'the support person' may also be 'the authorised third party representative'.

In this section the term 'disclosure' refers to telling someone else or an institution about the presence or the details of mental health problems.

1. The following people should encourage the person to disclose any mental health problems to the financial institution in order for them to reach the most suitable solution to the financial difficulties.

	Essential	Important	Don't know/Depends	Unimportant	Should not be included
Financial counsellor	◯	◯	◯	◯	◯
Mental health professional	◯	◯	◯	◯	◯
Support person	◯	◯	◯	◯	◯

2. The following people should advise the person that it is sometimes helpful to disclose their mental problems to the financial institution, depending on that financial institution's approach to people with mental health problems.

	Essential	Important	Don't know/Depends	Unimportant	Should not be included
Financial counsellor	◯	◯	◯	◯	◯
Mental health professional	◯	◯	◯	◯	◯
Support person	◯	◯	◯	◯	◯

3. The following people should encourage the person to disclose any mental health problems to the financial institution, in order to reach the most suitable solution to their financial difficulties.

	Essential	Important	Don't know/Depends	Unimportant	Should not be included
Financial counsellor	◯	◯	◯	◯	◯
Mental health professional	◯	◯	◯	◯	◯
Support person	◯	◯	◯	◯	◯

4. Do you have any comments on these statements? Is there anything you would like to add to this section? Please write your suggestions in the box provided.

5. <u>The authorised third party representative</u> should disclose any mental health problems the person has to the financial institution, with the person's permission. This will assist the financial institution staff to reach the most suitable solution to the person's financial difficulties. (Note: the 'authorised third party representative' may also be 'the support person'.)

Essential	Important	Don't know/Depends	Unimportant	Should not be included
○	○	○	○	○

6. Do you have any comments on these statements? Is there anything you would like to add to this section? Please write your suggestions in the box provided.

Section 6 (cont): Disclosure

This section asks you about what information **the financial counsellor** should know about disclosing mental health problems to a financial institution.

Please rate how important (from 'essential' to 'should not be included') you think it is that each statement be included in the guidelines.

Please keep the definitions in mind when answering the questions.

In this section the term 'disclosure' refers to telling someone else or an institution about the presence or the details of mental health problems.

1. The financial counsellor should be aware that people with mental health problems may be reluctant to tell financial institutions about their condition, e.g. because of shame or fear of being refused credit.

Essential	Important	Don't know/Depends	Unimportant	Should not be included
○	○	○	○	○

2. The financial counsellor should be aware that the person may be worried about how information about their mental health problems will be used.

Essential	Important	Don't know/Depends	Unimportant	Should not be included
○	○	○	○	○

3. If the person decides to disclose their mental health problems to a financial institution, the financial counsellor should encourage the person to do the following:

	Essential	Important	Don't know/Depends	Unimportant	Should not be included
Decide what they hope to achieve by disclosure and check if this is realistic.	○	○	○	○	○
Ask to speak to a specialist team member or staff member who works with people who have mental health problems or financial hardship, or a senior customer service team member.	○	○	○	○	○
Keep records of who they have spoken to, what was said and when.	○	○	○	○	○

4. The financial counsellor should inform the person that they may be asked to provide evidence of their mental health problems in order to reach the most suitable solution to the financial difficulties.

Essential	Important	Don't know/Depends	Unimportant	Should not be included
○	○	○	○	○

5. Do you have any comments on these statements? Is there anything you would like to add to this section? Please write your suggestions in the box provided.

6. <u>The financial counsellor</u> should inform the person that although they may have disclosed their mental health problems to a financial institution, the financial institution staff may not necessarily understand the impact of mental health problems on financial difficulties.

Essential	Important	Don't know/Depends	Unimportant	Should not be included
○	○	○	○	○

7. <u>The financial counsellor</u> should consider contacting the financial institution, with the person's consent, to explain how the mental health problems may affect the person's ability to manage their finances.

Essential	Important	Don't know/Depends	Unimportant	Should not be included
○	○	○	○	○

8. Where a person's mental health problems may lead to overspending, <u>the financial counsellor</u> should advise the person that it is possible to ask the financial institution to take a predetermined action if there are any unusual transactions.

Essential	Important	Don't know/Depends	Unimportant	Should not be included
○	○	○	○	○

9. <u>The financial counsellor</u> should advise the person that if they are more likely to overspend or sign up for credit cards when unwell, some financial institutions will add a note to their file, if requested.

Essential	Important	Don't know/Depends	Unimportant	Should not be included
○	○	○	○	○

10. <u>The financial counsellor</u> should ask the mental health professional to write a letter that the person can give to the financial institution stating how their mental health problems impact on their ability to manage their finances.

Essential	Important	Don't know/Depends	Unimportant	Should not be included
○	○	○	○	○

11. Do you have any comments on these statements? Is there anything you would like to add to this section? Please write your suggestions in the box provided.

Section 6 (cont): Disclosure

This section asks you about what information **the financial institution staff** should know about disclosing mental health problems to a financial institution.

Please rate how important (from 'essential' to 'should not be included') you think it is that each statement be included in the guidelines.

Please keep the definitions in mind when answering the questions.

In this section the term 'disclosure' refers to telling someone else or an institution about the presence or the details of mental health problems.

1. The financial institution staff should make the person with financial difficulties aware that if they disclose mental health problems to the financial institution, this will assist the financial institution staff to reach the most suitable solution to their financial difficulties.

Essential	Important	Don't know/Depends	Unimportant	Should not be included
○	○	○	○	○

2. When a person with financial difficulties discloses mental health problems, the financial institution staff should seek permission to record this information for use by other staff members dealing with their case.

Essential	Important	Don't know/Depends	Unimportant	Should not be included
○	○	○	○	○

3. When a person with financial difficulties discloses mental health problems, the financial institution staff should inform the person that they may be asked to provide evidence of their mental health problems.

Essential	Important	Don't know/Depends	Unimportant	Should not be included
○	○	○	○	○

4. When a person with financial difficulties discloses mental health problems, the financial institution staff should tell the person how the information will be used.

Essential	Important	Don't know/Depends	Unimportant	Should not be included
○	○	○	○	○

5. Do you have any comments on these statements? Is there anything you would like to add to this section? Please write your suggestions in the box provided.

6. When a person with financial difficulties has mental health problems recorded, any financial institution staff member who deals with their case should ensure that the recorded information is still current.

Essential	Important	Don't know/Depends	Unimportant	Should not be included
○	○	○	○	○

7. Where a mental health problem has been disclosed, the financial institution staff should review the person's situation prior to the end of any hardship payment arrangements.

Essential	Important	Don't know/Depends	Unimportant	Should not be included
○	○	○	○	○

8. Financial institutions should not sell or refer the debt of people with disclosed mental health problems. (Sale of debt means to transfer the ownership of the debt to another company. Referral of debt means to employ another agency to collect the debt on the financial institution's behalf.)

Essential	Important	Don't know/Depends	Unimportant	Should not be included
○	○	○	○	○

9. If a person has disclosed mental health problems and gives consent, the financial institution staff should ensure that this information is passed on in the event of any sale or referral of debt.

Essential	Important	Don't know/Depends	Unimportant	Should not be included
○	○	○	○	○

10. Do you have any comments on these statements? Is there anything you would like to add to this section? Please write your suggestions in the box provided.

Section 6 (cont): Disclosure

This section asks you about what information **the mental health professional** should know about disclosing mental health problems to a financial institution.

Please rate how important (from 'essential' to 'should not be included') you think it is that each statement be included in the guidelines.

Please keep the definitions in mind when answering the questions.

In this section the term 'disclosure' refers to telling someone else or an institution about the presence or the details of mental health problems.

1. The mental health professional should encourage the person with mental health problems to contact their financial institution as soon as they think they will experience financial difficulties.

Essential	Important	Don't know/Depends	Unimportant	Should not be included
○	○	○	○	○

2. The mental health professional should be aware that tailored payment arrangements with financial institutions are possible for people with mental problems.

Essential	Important	Don't know/Depends	Unimportant	Should not be included
○	○	○	○	○

3. The mental health professional should offer to write a letter that the person can give to the financial insitution stating how their mental health problems have an impact on their ability to manage their finances.

Essential	Important	Don't know/Depends	Unimportant	Should not be included
○	○	○	○	○

4. Do you have any comments on these statements? Is there anything you would like to add to this section? Please write your suggestions in the box provided.

Section 7: Documentation

This section asks you about what information **the financial counsellor, financial institution staff** and **mental health professional** should know about documentation that might benefit the person with mental health problems and financial difficulties and assist in communication between the financial counsellor, the mental health professional and the financial institution.

Please rate how important (from 'essential' to 'should not be included') you think it is that each statement be included in the guidelines.

Please keep the definitions in mind when answering the questions.

Advance Directive (An advance directive is a document describing how the person wants to be treated when they are unable to make their own decisions due to their present state of illness. Advance directives should specify the circumstances under which they will be used and who will take the various actions specified, e.g. when the person is becoming unwell or when they are acutely unwell.)

1. The financial counsellor should *offer to work* with the person and their support person to develop an advance directive about handling financial difficulties arising from future illness-related behaviour.

Essential	Important	Don't know/Depends	Unimportant	Should not be included
○	○	○	○	○

2. The financial counsellor should *work* with the person and their support person to develop an advance directive about handling financial difficulties arising from future illness-related behaviour.

Essential	Important	Don't know/Depends	Unimportant	Should not be included
○	○	○	○	○

3. Do you have any comments on these statements? Is there anything you would like to add to this section? Please write your suggestions in the box provided.

4. The mental health professional should *offer to work* with the person and their support person to develop an advance directive about handling financial difficulties arising from future illness-related behaviour.

Essential	Important	Don't know/Depends	Unimportant	Should not be included
○	○	○	○	○

5. The mental health professional should *work* with the person and their support person to develop an advance directive about handling financial difficulties arising from future illness-related behaviour.

Essential	Important	Don't know/Depends	Unimportant	Should not be included
○	○	○	○	○

6. Do you have any comments on these statements? Is there anything you would like to add to this section? Please write your suggestions in the box provided.

7. The financial counsellor and the mental health professional should should *work together* with the person and their support person to develop an advance directive that would include handling financial difficulties arising from future illness-related behaviour.

Essential	Important	Don't know/Depends	Unimportant	Should not be included
○	○	○	○	○

8. When developing an advance directive, the following topics should be considered, and relevant items included, depending on the individual situation:

	Essential	Important	Don't know/Depends	Unimportant	Should not be included
Allowing the support person to manage the finances.	○	○	○	○	○
Restricting internet access in order to prevent overspending, e.g. online shopping and gambling.	○	○	○	○	○
Restricting or denying access to various sources of funds, e.g. credit cards or ATM cards.	○	○	○	○	○
Restricting access to transport, e.g. taking away car keys so the person can't go shopping.	○	○	○	○	○
Restricting access to phone use, e.g. paid phone services.	○	○	○	○	○
Agreeing on a regular allowance.	○	○	○	○	○
Cancelling credit cards altogether.	○	○	○	○	○
Monitoring mail to ensure offers of credit cards are not taken up.	○	○	○	○	○
Requests for actions to be taken by the financial institutions, e.g. suspension of credit card when patterns of spending are unusual.	○	○	○	○	○

9. Do you have any comments on these statements? Is there anything you would like to add to this section? Please write your suggestions in the box provided.

10. <u>The financial institution staff</u> should know what an advance directive is and how to act upon it.

Essential	Important	Don't know/Depends	Unimportant	Should not be included
○	○	○	○	○

11. <u>Financial institutions</u> should have policies and procedures for dealing with advance directives.

Essential	Important	Don't know/Depends	Unimportant	Should not be included
○	○	○	○	○

12. Do you have any comments on these statements? Is there anything you would like to add to this section? Please write your suggestions in the box provided.

Section 7 (cont): Documentation

This section asks you about what information **the financial counsellor, financial institution staff** and **mental health professional** should know about documentation that might benefit the person with mental health problems and financial difficulties and assist in communication between the financial counsellor, the mental health professional and the financial institution.

Please rate how important (from 'essential' to 'should not be included') you think it is that each statement be included in the guidelines.

Please keep the definitions in mind when answering the questions.

Power of Attorney

1. The following people should encourage the person to arrange for financial power of attorney.

	Essential	Important	Don't know/Depends	Unimportant	Should not be included
Financial counsellor	◯	◯	◯	◯	◯
Financial institution staff	◯	◯	◯	◯	◯
Mental health professional	◯	◯	◯	◯	◯
Support person	◯	◯	◯	◯	◯

2. Where possible, the financial counsellor, mental health professional and support person should work together with the person and other appropriate professionals when making a decision about power of attorney.

Essential	Important	Don't know/Depends	Unimportant	Should not be included
◯	◯	◯	◯	◯

3. Do you have any comments on these statements? Is there anything you would like to add to this section? Please write your suggestions in the box provided.

Financial Administration Order (An Administration Order is a legal order to appoint an administrator to make financial decisions for a person with a mental incapacity.)

4. The following people should be aware of what a financial administration order is and the implications for the person and the financial institution.

	Essential	Important	Don't know/Depends	Unimportant	Should not be included
Financial counsellor	◯	◯	◯	◯	◯
Financial institution staff	◯	◯	◯	◯	◯
Mental health professional	◯	◯	◯	◯	◯
Support person	◯	◯	◯	◯	◯

5. The following people should consider applying for an administration order when they believe the person continues to make decisions causing financial harm, where possible in consultation with the person and other appropriate professionals.

	Essential	Important	Don't know/Depends	Unimportant	Should not be included
Financial counsellor	◯	◯	◯	◯	◯
Mental health professional	◯	◯	◯	◯	◯
Support person	◯	◯	◯	◯	◯

6. Do you have any comments on these statements? Is there anything you would like to add to this section? Please write your suggestions in the box provided.

Forms

7. There should be a standardised form that is filled out by the mental health professional to assist the understanding of the following people:

Essential	Important	Don't know/Depends	Unimportant	Should not be included
◯	◯	◯	◯	◯

8. Financial institutions should have a standard form allowing people with a history of mental health problems and financial difficulties to nominate a third party point of contact who can negotiate on their behalf, should they become acutely unwell.

Essential	Important	Don't know/Depends	Unimportant	Should not be included
◯	◯	◯	◯	◯

9. Do you have any comments on these statements? Is there anything you would like to add to this section? Please write your suggestions in the box provided.

Section 7 (cont): Documentation

This section asks you about what information **the financial counsellor, financial institution staff** and **mental health professional** should know about documentation that might benefit the person with mental health problems and financial difficulties and assist in communication between the financial counsellor, the mental health professional and the financial institution.

Please rate how important (from 'essential' to 'should not be included') you think it is that each statement be included in the guidelines.

Please keep the definitions in mind when answering the questions.

Debt and Mental Illness Evidence Form

The following questions are about whether there should be a standardised Debt and Mental Illness Evidence Form that a mental health professional can use to communicate information about a person's mental health problems to financial counsellors and financial institutions. Such a form may benefit the person with mental health problems and financial difficulties in that it will allow the financial counsellor and the financial institution staff to find the most appropriate solution to the person's financial difficulties. It should be noted that the information on the form may be considered by the financial institution to determine the person's suitability for future borrowing.

1. The following information should be included in a Debt and Mental Illness Evidence Form:

	Essential	Important	Don't know/Depends	Unimportant	Should not be included	This is not my area of expertise
Name, contact details and profession of the person requesting that the form be completed, e.g. financial institution staff, the financial counsellor.	○	○	○	○	○	○
Signature of the mental health professional.	○	○	○	○	○	○
A disclosure statement indicating what this form could be used for in the future.	○	○	○	○	○	○
Details about the mental health problems of the person.	○	○	○	○	○	○
Details of how the mental health problems affect the person's ability to resolve their current financial difficulties.	○	○	○	○	○	○
Details of the treatment/support the person is receiving with regards to their financial difficulties.	○	○	○	○	○	○
Details of the treatment/support the person is receiving with regards to their mental health problems.	○	○	○	○	○	○
Any factors that may be relevant to communicating effectively with the person about their financial difficulties, e.g. preferred mode of communication, comprehension problems.	○	○	○	○	○	○
The person's capacity for and level of understanding.	○	○	○	○	○	○
The person's capacity for decision making.	○	○	○	○	○	○
Details about when the person's mental health problems started.	○	○	○	○	○	○
Details about whether their mental health problems are ongoing.	○	○	○	○	○	○
Details of when treatment started and whether it is ongoing.	○	○	○	○	○	○
Details of how long the condition is likely to last.	○	○	○	○	○	○
An option for the mental health professional to provide any additional relevant information.	○	○	○	○	○	○
A statement about how long the information on the form is valid for.	○	○	○	○	○	○
Option for listing an authorised	○	○	○	○	○	○

third party representative who the financial institution can contact if they are unable to contact the person.						
Option for authorising financial institutions to contact the mental health professional if they are unable to contact the person or their authorised party representative.	○	○	○	○	○	○
The person's signature to indicate the person's informed consent to give this form to the financial institution and the financial counsellor.	○	○	○	○	○	○

Please provide any additional information you believe should be included:

2. The financial counsellor should ask all clients with mental health problems to obtain this completed form from their mental health professional.

Essential	Important	Don't know/Depends	Unimportant	Should not be included
○	○	○	○	○

3. The financial counsellor should ask the person with mental health problems to obtain this completed form from their mental health professional, when proof of condition is required to obtain a particular outcome from the financial institution.

Essential	Important	Don't know/Depends	Unimportant	Should not be included
○	○	○	○	○

4. Do you have any comments on these statements? Is there anything you would like to add to this section? Please write your suggestions in the box provided.

5. The mental health professional should always complete this form when first consulting with a person with financial difficulties.

Essential	Important	Don't know/Depends	Unimportant	Should not be included
○	○	○	○	○

6. The mental health professional should complete this form when the information is requested by a financial counsellor.

Essential	Important	Don't know/Depends	Unimportant	Should not be included
○	○	○	○	○

7. The mental health professional should complete this form when the information is requested by a financial institution.

Essential	Important	Don't know/Depends	Unimportant	Should not be included
○	○	○	○	○

8. The mental health professional should give this completed form to the person to pass on to *the financial counsellor*.

Essential	Important	Don't know/Depends	Unimportant	Should not be included
○	○	○	○	○

9. The mental health professional should give this completed form to the person to pass on to *the financial institution*.

Essential	Important	Don't know/Depends	Unimportant	Should not be included
○	○	○	○	○

10. The mental health professional should give this completed form directly to *the financial counsellor*, with the person's permission.

Essential	Important	Don't know/Depends	Unimportant	Should not be included
○	○	○	○	○

11. The mental health professional should give this completed form directly to *the financial institution*, with the person's permission.

Essential	Important	Don't know/Depends	Unimportant	Should not be included
○	○	○	○	○

12. The mental health professional should review this form annually and resubmit it if needed.

Essential	Important	Don't know/Depends	Unimportant	Should not be included
○	○	○	○	○

13. The mental health professional should review this form every 6 months after initial completion and resubmit it if needed.

Essential	Important	Don't know/Depends	Unimportant	Should not be included
○	○	○	○	○

14. The mental health professional should review this form when the person's mental health problems change and resubmit it if needed.

Essential	Important	Don't know/Depends	Unimportant	Should not be included
○	○	○	○	○

15. Do you have any comments on these statements? Is there anything you would like to add to this section? Please write your suggestions in the box provided.

16. If there is a cost associated with the mental health professional completing the form, this should be paid by:

	Essential	Important	Don't know/Depends	Unimportant	Should not be included
The financial counsellor	○	○	○	○	○
The financial institution	○	○	○	○	○
The mental health professional (i.e. waive cost)	○	○	○	○	○
The person	○	○	○	○	○

17. Financial institutions should develop, in collaboration with mental health professionals and financial counsellors, a policy about which types of health professionals can provide evidence of the impact of mental health problems on financial difficulties.

Essential	Important	Don't know/Depends	Unimportant	Should not be included
○	○	○	○	○

18. Do you have any comments on these statements? Is there anything you would like to add to this section? Please write your suggestions in the box provided.

Section 8: Actions to help the person

This section asks you about what information **the financial counsellor** and **mental health professional** should know about specific actions that can be taken to help the person with mental health problems and financial difficulties.

Please rate how important (from 'essential' to 'should not be included') you think it is that each statement be included in the guidelines.

Please keep the definitions in mind when answering the questions.

1. The financial counsellor should help the person to prevent future financial difficulties by working out how their mental health problems may lead to overspending and other behaviour that results in financial difficulties.

Essential	Important	Don't know/Depends	Unimportant	Should not be included
○	○	○	○	○

2. The financial counsellor should help the person work out their own warning signs that indicate that they may be at risk of relapse of mental health problems.

Essential	Important	Don't know/Depends	Unimportant	Should not be included
○	○	○	○	○

3. The financial counsellor should use appropriate psychological strategies to help the person to deal with any mental health problems associated with financial difficulties.

Essential	Important	Don't know/Depends	Unimportant	Should not be included
○	○	○	○	○

4. The financial counsellor should help the person to understand the interaction of finances, financial distress and mental health problems.

Essential	Important	Don't know/Depends	Unimportant	Should not be included
○	○	○	○	○

5. Do you have any comments on these statements? Is there anything you would like to add to this section? Please write your suggestions in the box provided.

6. <u>The financial counsellor</u> should advise the person to use the following strategies to help cope with strong/unhelpful emotions related to their mental health problems and financial difficulties:

	Essential	Important	Don't know/Depends	Unimportant	Should not be included
Write down their worries and concerns.	○	○	○	○	○
Take a problem solving approach to address their worries and concerns.	○	○	○	○	○
Identify the specific feelings they are experiencing and the concern/worry that may be underlying each of these feelings, e.g. worried about not being able to pay the rent or mortgage, or ashamed about losing their job.	○	○	○	○	○
Identify friends and family members who they can talk to and who will help them to remain positive.	○	○	○	○	○
Maintain a balanced diet and get some regular exercise.	○	○	○	○	○
Avoid drugs and alcohol.	○	○	○	○	○
If they have a pre-existing medical condition that may be aggravated by stress, encourage them to talk to their doctor.	○	○	○	○	○
Be aware that recovery from a significant stress takes time.	○	○	○	○	○
Recall a past difficult situation that they overcame and identify what worked then and apply it to the current situation.	○	○	○	○	○

Please provide any other additional strategies:

7. <u>The financial counsellor</u> should allow extra time when working with the person with mental health problems, e.g. flexible approach to appointments, number of sessions, length of appointments.

Essential	Important	Don't know/Depends	Unimportant	Should not be included
○	○	○	○	○

8. <u>The financial counsellor</u> should work with the mental health professional to determine the person's capacity to understand their financial situation.

Essential	Important	Don't know/Depends	Unimportant	Should not be included
○	○	○	○	○

9. <u>The financial counsellor</u> should consider the following things when working with the person to develop financial strategies:

	Essential	Important	Don't know/Depends	Unimportant	Should not be included
Ability to engage meaningfully with financial institutions.	◯	◯	◯	◯	◯
Ability to implement realistic repayment plans.	◯	◯	◯	◯	◯
Long-term impact of the mental illness.	◯	◯	◯	◯	◯
The person's capacity for and level of understanding.	◯	◯	◯	◯	◯
The person's capacity for decision making.	◯	◯	◯	◯	◯

Please provide any additional considerations:

10. <u>The financial counsellor</u> should be aware that people with mental health problems may have memory and concentration problems.

Essential	Important	Don't know/Depends	Unimportant	Should not be included
◯	◯	◯	◯	◯

11. Do you have any comments on these statements? Is there anything you would like to add to this section? Please write your suggestions in the box provided.

Section 8 (cont): Actions to help the person

This section asks you about what information **financial institutions** and **the financial institution staff** should know about specific actions that can be taken to help the person with mental health problems and financial difficulties.

Please rate how important (from 'essential' to 'should not be included') you think it is that each statement be included in the guidelines.

Please keep the definitions in mind when answering the questions.

1. The financial institution staff should work with the financial counsellor to determine the person's capacity to understand their financial situation.

Essential	Important	Don't know/Depends	Unimportant	Should not be included
○	○	○	○	○

2. The financial institution staff should ask the person how their mental health problems impact on their ability to repay their debt.

Essential	Important	Don't know/Depends	Unimportant	Should not be included
○	○	○	○	○

3. If an authorised third party (e.g. a financial counsellor) is not acting for the person, the financial institution staff should work with the mental health professional to determine the person's capacity to understand their financial situation.

Essential	Important	Don't know/Depends	Unimportant	Should not be included
○	○	○	○	○

4. When the financial institution staff is working with the mental health professional to determine the person's capacity, the following issues should be addressed:

	Essential	Important	Don't know/Depends	Unimportant	Should not be included
Ability to engage meaningfully with financial institutions.	○	○	○	○	○
Implement repayment plans.	○	○	○	○	○
Long-term impact of the mental illness.	○	○	○	○	○
The person's capacity for and level of understanding.	○	○	○	○	○
The person's capacity for decision making.	○	○	○	○	○

5. Do you have any comments on these statements? Is there anything you would like to add to this section? Please write your suggestions in the box provided.

6. <u>The financial institution</u> should follow the hardship provisions in the most recent voluntary <u>Code of Banking Practice</u>.

Essential	Important	Don't know/Depends	Unimportant	Should not be included
○	○	○	○	○

7. <u>The financial institution staff</u> should adopt a respectful and positive approach when working with the person with mental health problems.

Essential	Important	Don't know/Depends	Unimportant	Should not be included
○	○	○	○	○

8. <u>The financial institution staff</u> should be proactive in assisting people with mental health problems before they experience financial difficulties, e.g. providing tips and tools for financial management, and financial counselling contact information.

Essential	Important	Don't know/Depends	Unimportant	Should not be included
○	○	○	○	○

9. Where appropriate, <u>the financial institution staff</u> should encourage the person to find a support person.

Essential	Important	Don't know/Depends	Unimportant	Should not be included
○	○	○	○	○

10. <u>The financial institution staff</u> should suspend collection activity to give the person sufficient time to consult a financial counsellor or mental health professional.

Essential	Important	Don't know/Depends	Unimportant	Should not be included
○	○	○	○	○

11. <u>The financial institution staff</u> and the person with mental health problems should come to an agreement about what is sufficient time to consult a financial counsellor or mental health professional.

Essential	Important	Don't know/Depends	Unimportant	Should not be included
○	○	○	○	○

12. If the person with disclosed mental health problems is not adhering to arrangements tailored to their situation, then <u>the financial institution staff</u> can implement normal debt recovery procedures.

Essential	Important	Don't know/Depends	Unimportant	Should not be included
◯	◯	◯	◯	◯

13. Do you have any comments on these statements? Is there anything you would like to add to this section? Please write your suggestions in the box provided.

14. If the person with disclosed mental health problems is not adhering to arrangements tailored to their situation, <u>the financial institution staff</u> should:

	Essential	Important	Don't know/Depends	Unimportant	Should not be included
Not sell debt to third parties.	◯	◯	◯	◯	◯
Encourage authorised third party representation, e.g. a professional, such as a community lawyer or financial counsellor, advocating on their behalf.	◯	◯	◯	◯	◯
Make a site visit to assess the situation before proceeding with any sale of home.	◯	◯	◯	◯	◯
Defer action when the person is acutely unwell or in a mental health crisis.	◯	◯	◯	◯	◯
Suspend interest payments when the person is acutely unwell or in a mental health crisis.	◯	◯	◯	◯	◯
Ensure any authorised third party representatives are contacted.	◯	◯	◯	◯	◯

Please write any other suggestions

15. <u>The financial institution staff</u> should only initiate court action to pursue the person's debt as a last resort.

Essential	Important	Don't know/Depends	Unimportant	Should not be included
◯	◯	◯	◯	◯

16. Do you have any comments on these statements? Is there anything you would like to add to this section? Please write your suggestions in the box provided.

Section 8 (cont): Actions to help the person

This section asks you about what information **the financial counsellor, the financial institution staff** and **the mental health professional** should know about specific actions that can be taken to help the person with mental health problems and financial difficulties.

Please rate how important (from 'essential' to 'should not be included') you think it is that each statement be included in the guidelines.

Please keep the definitions in mind when answering the questions.

1. The mental health professional should be trained in the following basic skills to assist the person to build financial stability:

	Essential	Important	Don't know/Depends	Unimportant	Should not be included
Assessing the pattern or history of the person's financial concerns.	○	○	○	○	○
Assisting the person to identify financial practices that benefit or add to financial stability, e.g. saving, budgeting, comparison shopping.	○	○	○	○	○
Assisting the person to identify financial practices that have led to the problem debt, e.g. impulsive spending, unstable work history.	○	○	○	○	○
Identifying triggers and consequences of financial successes and failures.	○	○	○	○	○
Assisting the person to create a basic budget.	○	○	○	○	○
Educating the person on the impact that their symptoms may have on their financial stability/financial difficulties.	○	○	○	○	○
Assisting the person to identify how their symptoms impact on their financial difficulties.	○	○	○	○	○
Meeting regularly to review person's financial plans/goals/status.	○	○	○	○	○
Educating the person on typical banking procedures.	○	○	○	○	○

Other (please specify)

2. The mental health professional should be aware that there are a range of concessions and financial assistance available to the person and encourage them to apply for these, e.g. reduced car registration, council rates and energy costs to health care card holders.

Essential	Important	Don't know/Depends	Unimportant	Should not be included
◯	◯	◯	◯	◯

3. The mental health professional should be aware that every state and territory has an ombudsman to deal with difficulties with energy providers.

Essential	Important	Don't know/Depends	Unimportant	Should not be included
◯	◯	◯	◯	◯

4. The mental health professional should be aware that utility providers (gas, electricity, water) have, by law, provisions for people in financial hardship and should encourage the person to make and maintain contact with the utility provider and request 'hardship' assistance, once financial difficulties are evident.

Essential	Important	Don't know/Depends	Unimportant	Should not be included
◯	◯	◯	◯	◯

5. The mental health professional should be aware that water providers cannot disconnect mains water supplies because it is considered a health risk.

Essential	Important	Don't know/Depends	Unimportant	Should not be included
◯	◯	◯	◯	◯

6. Do you have any comments on these statements? Is there anything you would like to add to this section? Please write your suggestions in the box provided.

Centrelink

7. The following people should be aware of the various Centrelink benefits that are available to the person with mental health problems and financial difficulties:

	Essential	Important	Don't know/Depends	Unimportant	Should not be included
Financial counsellor	◯	◯	◯	◯	◯
Financial institution staff	◯	◯	◯	◯	◯
Mental health professional	◯	◯	◯	◯	◯
Support person	◯	◯	◯	◯	◯

8. The following people should advise the person with mental health problems and financial difficulties to discuss with Centrelink their eligibility for benefits:

	Essential	Important	Don't know/Depends	Unimportant	Should not be included
Financial counsellor	◯	◯	◯	◯	◯
Financial institution staff	◯	◯	◯	◯	◯
Mental health professional	◯	◯	◯	◯	◯
Support person	◯	◯	◯	◯	◯

9. Do you have any comments on these statements? Is there anything you would like to add to this section? Please write your suggestions in the box provided.

Section 9: Communication

This section asks you about what information **the financial counsellor** should know about how to effectively communicate with the person with mental health problems and financial difficulties.

Please rate how important (from 'essential' to 'should not be included') you think it is that each statement be included in the guidelines.

Please keep the definitions in mind when answering the questions.

1. When meeting the person face to face and the person is unable to communicate effectively (e.g. with mania), the financial counsellor should:

	Essential	Important	Don't know/Depends	Unimportant	Should not be included
Encourage them to write down their responses to questions.	◯	◯	◯	◯	◯
Consider asking structured rather than open-ended questions.	◯	◯	◯	◯	◯

Other (please specify)

2. When meeting the person face to face and the person is unable to communicate effectively (e.g. with mania), the financial institution staff should:

	Essential	Important	Don't know/Depends	Unimportant	Should not be included
Encourage them to write down their responses to questions.	◯	◯	◯	◯	◯
Consider asking structured rather than open-ended questions.	◯	◯	◯	◯	◯

Other (please specify)

3. When meeting the person face to face and the person is presenting aggressively, the financial counsellor should:

	Essential	Important	Don't know/Depends	Unimportant	Should not be included
Speak to the person slowly and confidently with a gentle, caring tone of voice.					
Not respond in a hostile, disciplinary or challenging manner.					
Not argue with the person.					
Not threaten them as this may increase fear or prompt aggressive behaviour.					
Avoid raising your voice or talking too fast.					
Be aware that the person may overreact to negative words; therefore, use positive words (such as 'stay calm') instead of negative words (such as 'don't fight').					
Stay calm and avoid nervous behaviour (e.g. shuffling your feet, fidgeting, making abrupt movements).					
Do not restrict the person's movement (e.g. if he or she wants to pace up and down the room).					
B aware that the person's symptoms or fear causing their aggression might be exacerbated if you take certain steps (e.g. involve the police).					
Consider taking a break from the conversation to allow the person a chance to calm down.					
Consider inviting the person to sit down if they are standing.					

Other (please specify)

4. When meeting the person face to face and the person is <u>presenting aggressively</u>, <u>the financial institution staff</u> should:

	Essential	Important	Don't know/Depends	Unimportant	Should not be included
Speak to the person slowly and confidently with a gentle, caring tone of voice.	◯	◯	◯	◯	◯
Not respond in a hostile, disciplinary or challenging manner.	◯	◯	◯	◯	◯
Not argue with the person.	◯	◯	◯	◯	◯
Not threaten them as this may increase fear or prompt aggressive behaviour.	◯	◯	◯	◯	◯
Avoid raising your voice or talking too fast.	◯	◯	◯	◯	◯
Be aware that the person may overreact to negative words; therefore, use positive words (such as ‘stay calm’) instead of negative words (such as ‘don’t fight’).	◯	◯	◯	◯	◯
Stay calm and avoid nervous behaviour (e.g. shuffling your feet, fidgeting, making abrupt movements).	◯	◯	◯	◯	◯
Do not restrict the person’s movement (e.g. if he or she wants to pace up and down the room).	◯	◯	◯	◯	◯
B aware that the person’s symptoms or fear causing their aggression might be exacerbated if you take certain steps (e.g. involve the police).	◯	◯	◯	◯	◯
Consider taking a break from the conversation to allow the person a chance to calm down.	◯	◯	◯	◯	◯
Consider inviting the person to sit down if they are standing.	◯	◯	◯	◯	◯

Other (please specify)

5. The following people should be aware that people with mental health problems may have memory or concentration problems that require simple and concise information about their situation and that this information may need to be repeated.

	Essential	Important	Don't know/Depends	Unimportant	Should not be included
Financial counsellor	◯	◯	◯	◯	◯
Financial institution staff	◯	◯	◯	◯	◯
Support person	◯	◯	◯	◯	◯

6. If the person has memory and concentration problems, <u>the financial counsellor</u> should give the person a plain language summary of information and options discussed during their appointment.

Essential	Important	Don't know/Depends	Unimportant	Should not be included
○	○	○	○	○

7. If the person has disclosed mental health problems, <u>the financial institution staff</u> should offer to give the person a plain language summary of information and options discussed during their phone conversation.

Essential	Important	Don't know/Depends	Unimportant	Should not be included
○	○	○	○	○

8. Do you have any comments on these statements? Is there anything you would like to add to this section? Please write your suggestions in the box provided.

9. <u>The financial institution staff</u> should allow the person to choose their preferred method of contact.

Essential	Important	Don't know/Depends	Unimportant	Should not be included
○	○	○	○	○

10. <u>The financial institution staff</u> should provide the following options for people with mental health problems to contact them about financial difficulties:

	Essential	Important	Don't know/Depends	Unimportant	Should not be included
Face to face meeting	○	○	○	○	○
Letters	○	○	○	○	○
Interactive electronic communication, e.g. internet chat, 2-way SMS	○	○	○	○	○
Non-interactive electronic communication, e.g. email, web form	○	○	○	○	○
Voice, e.g. telephone, Skype	○	○	○	○	○

11. The financial institution staff should choose their preferred method of contact when communicating with the person.

Essential	Important	Don't know/Depends	Unimportant	Should not be included
○	○	○	○	○

12. Do you have any comments on these statements? Is there anything you would like to add to this section? Please write your suggestions in the box provided.

Section 10: Working with the person over the phone

This section contains statements that apply to when the **financial counsellor** or the **financial institution staff** are talking over the phone to a person who appears to be in one of the following negative emotional states:

• Confused or out of touch with reality

• Aggressive

• Highly distressed or anxious

• Depressed

Please rate how important (from 'essential' to 'should not be included') you think it is that each statement be included in the guidelines.

Please keep the definitions in mind when answering the questions. On this page the term 'the person' refers to a person who appears to be in one of the four negative emotional states listed above.

1. The financial counsellor should do the following when talking over the phone with the person:

	Essential	Important	Don't know/Depends	Unimportant	Should not be included
Take some time to listen.	◯	◯	◯	◯	◯
Allow the person enough time to think and talk.	◯	◯	◯	◯	◯
As far as possible, let the person set the pace and style of the conversation.	◯	◯	◯	◯	◯
Listen non-judgmentally so that the person can feel like they have been heard and understood.	◯	◯	◯	◯	◯
Show empathy, e.g. "this must be very difficult for you".	◯	◯	◯	◯	◯
Convey acceptance to the person, i.e. respect the person's feelings and opinions even if you do not agree with them, not criticising or trivialising what the person is saying, avoiding judging what the person is saying.	◯	◯	◯	◯	◯
Convey genuineness to the person, i.e. your non-verbal cues match what you are saying.	◯	◯	◯	◯	◯
Don't label the person's feelings for them.	◯	◯	◯	◯	◯
Don't pressure the person to talk about their feelings.	◯	◯	◯	◯	◯
Be on the lookout for any indications that the person may be in crisis, e.g. suicidal intentions, non-suicidal self-injury.	◯	◯	◯	◯	◯
Offer to call the person back at a better time.	◯	◯	◯	◯	◯
Offer relevant resources, e.g. contact details for Lifeline, local mental health service.	◯	◯	◯	◯	◯

Please add any other actions:

2. The financial institution staff should do the following when talking over the phone with the person:

	Essential	Important	Don't know/Depends	Unimportant	Should not be included
Take some time to listen.	○	○	○	○	○
Allow the person enough time to think and talk.	○	○	○	○	○
As far as possible, let the person set the pace and style of the conversation.	○	○	○	○	○
Listen non-judgmentally so that the person can feel like they have been heard and understood.	○	○	○	○	○
Show empathy, e.g. “this must be very difficult for you”.	○	○	○	○	○
Convey acceptance to the person, i.e. respect the person’s feelings and opinions even if you do not agree with them, not criticising or trivialising what the person is saying, avoiding judging what the person is saying.	○	○	○	○	○
Convey genuineness to the person, i.e. your non-verbal cues match what you are saying.	○	○	○	○	○
Don’t label the person’s feelings for them.	○	○	○	○	○
Don’t pressure the person to talk about their feelings.	○	○	○	○	○
Be on the lookout for any indications that the person may be in crisis, e.g. suicidal intentions, non-suicidal self-injury.	○	○	○	○	○
Offer to call the person back at a better time.	○	○	○	○	○
Offer relevant resources, e.g. contact details for Lifeline, local mental health service.	○	○	○	○	○

Please add any other actions:

3. The following people should be aware of their own attitudes about mental health problems and how these might affect their ability to respond to the person non-judgmentally.

	Essential	Important	Don't know/Depends	Unimportant	Should not be included
Financial counsellor	○	○	○	○	○
Financial institution staff	○	○	○	○	○

4. The following people should set aside any negative beliefs and reactions in order to focus on the needs of the person they are assisting.

	Essential	Important	Don't know/Depends	Unimportant	Should not be included
Financial counsellor	○	○	○	○	○
Financial institution staff	○	○	○	○	○

5. The following people should not express negative judgments to the person as this can get in the way of assisting them.

	Essential	Important	Don't know/Depends	Unimportant	Should not be included
Financial counsellor	○	○	○	○	○
Financial institution staff	○	○	○	○	○

6. When there is serious concern about the person's safety, or in situations where the relevant actions do not seem to be working, the following people should contact their immediate manager.

	Essential	Important	Don't know/Depends	Unimportant	Should not be included
Financial counsellor	○	○	○	○	○
Financial institution staff	○	○	○	○	○

7. When there is serious concern about the person's safety, or in situations where the relevant actions do not seem to be working, the following people should contact their local mental health service.

	Essential	Important	Don't know/Depends	Unimportant	Should not be included
Financial counsellor	○	○	○	○	○
Financial institution staff	○	○	○	○	○

8. Do you have any comments on these statements? Is there anything you would like to add to this section? Please write your suggestions in the box provided.

Section 10 (cont): Working with the person over the phone

This section contains statements that apply to when the **financial counsellor** or the **financial institution staff** are talking over the phone to a person who appears to be in one of the following negative emotional states:

- Confused or out of touch with reality
- Aggressive
- Highly distressed or anxious
- Depressed

Please rate how important (from 'essential' to 'should not be included') you think it is that each statement be included in the guidelines.

Please keep the definitions in mind when answering the questions. On this page the term 'the person' refers to a person who appears to be in one of the four negative emotional states listed above.

1. If the financial counsellor has listened non-judgmentally and the person remains in their negative emotional state they should offer the following:

	Essential	Important	Don't know/Depends	Unimportant	Should not be included
Referral to a telephone counselling service, e.g. Lifeline.	○	○	○	○	○
Information about local mental health services (verbally or in an email, or via the post).	○	○	○	○	○
Relevant websites, e.g. beyondblue.	○	○	○	○	○

Please add any other actions:

2. If the financial institution staff has listened non-judgmentally and the person remains in their negative emotional state they should offer the following:

	Essential	Important	Don't know/Depends	Unimportant	Should not be included
Referral to a telephone counselling service, e.g. Lifeline.	○	○	○	○	○
Information about local mental health services (verbally or in an email, or via the post).	○	○	○	○	○
Relevant websites, e.g. beyondblue.	○	○	○	○	○

Please add any other actions:

3. The <u>financial counsellor</u> should use the following verbal skills to show the person that they are listening:

	Essential	Important	Don't know/Depends	Unimportant	Should not be included
Ask questions which show that they genuinely care, e.g. clarifying questions to show that they understand what the person is saying.	◯	◯	◯	◯	◯
Check their understanding by re-stating what the person has said.	◯	◯	◯	◯	◯
Summarise facts and feelings.	◯	◯	◯	◯	◯
Listen not only to what the person says, but how they say it, i.e. their tone of voice.	◯	◯	◯	◯	◯
Use minimal prompts, such as “I see” and “Ah”, when necessary, to keep the conversation going.	◯	◯	◯	◯	◯
Be patient, even when the person may not be communicating well, may be repetitive or may be speaking slowly and unclearly.	◯	◯	◯	◯	◯
Allow pauses and silences to give the person time to think or gather their thoughts.	◯	◯	◯	◯	◯

Please add any other actions:

4. The <u>financial institution staff</u> should use the following verbal skills to show the person that they are listening:

	Essential	Important	Don't know/Depends	Unimportant	Should not be included
Ask questions which show that they genuinely care, e.g. clarifying questions to show that they understand what the person is saying.	◯	◯	◯	◯	◯
Check their understanding by re-stating what the person has said.	◯	◯	◯	◯	◯
Summarise facts and feelings.	◯	◯	◯	◯	◯
Listen not only to what the person says, but how they say it, i.e. their tone of voice.	◯	◯	◯	◯	◯
Use minimal prompts, such as "I see" and "Ah", when necessary, to keep the conversation going.	◯	◯	◯	◯	◯
Be patient, even when the person may not be communicating well, may be repetitive or may be speaking slowly and unclearly.	◯	◯	◯	◯	◯
Allow pauses and silences to give the person time to think or gather their thoughts.	◯	◯	◯	◯	◯

Please add any other actions:

5. The financial counsellor should avoid:

	Essential	Important	Don't know/Depends	Unimportant	Should not be included
Using a hostile or sarcastic tone when the person’s responses are not what they expect.	◯	◯	◯	◯	◯
Trivialising the person’s experiences, e.g. telling them to “put a smile on their face,” to “get their act together,” or to “lighten up”.	◯	◯	◯	◯	◯
Belittling or dismissing the person's feelings, e.g. attempting to say something positive like, "You don't seem that bad to me."	◯	◯	◯	◯	◯
Speaking with a patronising tone of voice.	◯	◯	◯	◯	◯
Trying to come up with answers to their problems.	◯	◯	◯	◯	◯
Being critical or expressing frustration at the person for being in a negative emotional state.	◯	◯	◯	◯	◯
Interrupting the person when they are speaking, especially to give your opinion.	◯	◯	◯	◯	◯
Confronting the person, unless it is necessary to prevent harmful or dangerous acts.	◯	◯	◯	◯	◯

Please add any other actions:

6. The financial institution staff should avoid:

	Essential	Important	Don't know/Depends	Unimportant	Should not be included
Using a hostile or sarcastic tone when the person's responses are not what they expect.	○	○	○	○	○
Trivialising the person's experiences, e.g. telling them to "put a smile on their face," to "get their act together," or to "lighten up".	○	○	○	○	○
Belittling or dismissing the person's feelings, e.g. attempting to say something positive like, "You don't seem that bad to me."	○	○	○	○	○
Speaking with a patronising tone of voice.	○	○	○	○	○
Trying to come up with answers to their problems.	○	○	○	○	○
Being critical or expressing frustration at the person for being in a negative emotional state.	○	○	○	○	○
Interrupting the person when they are speaking, especially to give your opinion.	○	○	○	○	○
Confronting the person, unless it is necessary to prevent harmful or dangerous acts.	○	○	○	○	○

Please add any other actions:

7. The financial counselling service should have a list of statements that their staff can paraphrase, using their own words, to help guide the conversation when talking to the person who appears to be in a negative emotional state, e.g. "It can be important to talk to someone confidentially who is not a friend or family member about what has been happening. Are you currently speaking to someone who can give you that support?" or "It is understandable that you are anxious at a time like this given everything that is happening for you at the moment....Lots of people say that going through financial difficulties can be very stressful..."

Essential	Important	Don't know/Depends	Unimportant	Should not be included
○	○	○	○	○

8. The financial institution should have a list of statements that their staff can paraphrase, using their own words, to help guide the conversation when talking to the person who appears to be in a negative emotional state, e.g. "It can be important to talk to someone confidentially who is not a friend or family member about what has been happening. Are you currently speaking to someone who can give you that support?" or "It is understandable that you are anxious at a time like this given everything that is happening for you at the moment....Lots of people say that going through financial difficulties can be very stressful..."

Essential	Important	Don't know/Depends	Unimportant	Should not be included
○	○	○	○	○

9. Do you have any comments on these statements? Is there anything you would like to add to this section? Please write your suggestions in the box provided.

Section 10 (cont): Working with the person over the phone

This section contains statements that apply to when the **financial counsellor** or the **financial institution staff** are talking over the phone to a person who is in one of the following specific negative emotional states, i.e. depressed, distressed, confused or out of touch with reality, or aggressive.

Please rate how important (from 'essential' to 'should not be included') you think it is that each statement be included in the guidelines.

Please keep the definitions in mind when answering the questions. On this page the term 'the person' refers to a person who appears to be in one of the four specified negative emotional states.

Sounds Depressed

1. The following people should be familiar with and listen for signs that may indicate that the person is feeling depressed, i.e. sadness, hopelessness, worthlessness, difficulty concentrating, difficulty making decisions, agitation, slow monotonous speech, etc.

	Essential	Important	Don't know/Depends	Unimportant	Should not be included
Financial counsellor	◯	◯	◯	◯	◯
Financial institution staff	◯	◯	◯	◯	◯

2. Do you have any comments on these statements? Is there anything you would like to add to this section? Please write your suggestions in the box provided.

Sounds Highly Distressed

3. The following people should be familiar with and listen for signs that may indicate that the person is distressed, i.e. worry, irritability, impatience, pressured speech, crying, etc.

	Essential	Important	Don't know/Depends	Unimportant	Should not be included
Financial counsellor	◯	◯	◯	◯	◯
Financial institution staff	◯	◯	◯	◯	◯

4. Do you have any comments on these statements? Is there anything you would like to add to this section? Please write your suggestions in the box provided.

Agressive

5. If the person is becoming aggressive, the financial counsellor should:

	Essential	Important	Don't know/Depends	Unimportant	Should not be included
Remain as calm as possible.					
Speak to the person slowly and confidently with a gentle, caring tone of voice.					
Not respond in a hostile, disciplinary or challenging manner.					
Not argue with the person.					
Not threaten them as this may increase fear or prompt aggressive behaviour.					
Avoid raising their voice or talking too fast.					
Be aware that the person may overreact to negative words; therefore, use positive words (such as 'Let's stay calm') instead of negative or patronising words (such as 'don't fight').					
Be aware that the person's symptoms or fear causing their aggression might be made worse if you take certain steps (e.g. involve the police).					
Consider taking a break from the conversation to allow the person a chance to calm down.					
Take any threats or warnings seriously, particularly if the person believes they are being persecuted.					

Other (please specify)

6. If the person is becoming aggressive, the financial institution staff should:

	Essential	Important	Don't know/Depends	Unimportant	Should not be included
Remain as calm as possible.	○	○	○	○	○
Speak to the person slowly and confidently with a gentle, caring tone of voice.	○	○	○	○	○
Not respond in a hostile, disciplinary or challenging manner.	○	○	○	○	○
Not argue with the person.	○	○	○	○	○
Not threaten them as this may increase fear or prompt aggressive behaviour.	○	○	○	○	○
Avoid raising their voice or talking too fast.	○	○	○	○	○
Be aware that the person may overreact to negative words; therefore, use positive words (such as 'Let's stay calm') instead of negative or patronising words (such as 'don't fight').	○	○	○	○	○
Be aware that the person's symptoms or fear causing their aggression might be made worse if you take certain steps (e.g. involve the police).	○	○	○	○	○
Consider taking a break from the conversation to allow the person a chance to calm down.	○	○	○	○	○
Take any threats or warnings seriously, particularly if the person believes they are being persecuted.	○	○	○	○	○

Other (please specify)

7. Do you have any comments on these statements? Is there anything you would like to add to this section? Please write your suggestions in the box provided.

Section 10 (cont): Working with the person over the phone

This section contains statements that apply to when the **financial counsellor** or the **financial institution staff** are talking over the phone to a person who is in one of the following specific negative emotional states, i.e. depressed, distressed, confused or out of touch with reality, or aggressive.

Please rate how important (from 'essential' to 'should not be included') you think it is that each statement be included in the guidelines.

Please keep the definitions in mind when answering the questions. On this page the term 'the person' refers to a person who appears to be in one of the four specified negative emotional states.

Sounds Confused or Out of Touch with Reality

1. The following people should be familiar with and listen for signs that may indicate that the person may be confused or out of touch with reality, e.g. bizarre beliefs, paranoia, illogical thinking, etc.

	Essential	Important	Don't know/Depends	Unimportant	Should not be included
Financial counsellor	○	○	○	○	○
Financial institution staff	○	○	○	○	○

2. If possible, the following people should request that the person who may be confused or out of touch with reality finds a spot to talk that is quiet and free of distractions.

	Essential	Important	Don't know/Depends	Unimportant	Should not be included
Financial counsellor	○	○	○	○	○
Financial institution staff	○	○	○	○	○

3. If possible, the following people should finds a spot to talk that is quiet and free of background noise when they are talking to the person that may be confused or out of touch with reality.

	Essential	Important	Don't know/Depends	Unimportant	Should not be included
Financial counsellor	○	○	○	○	○
Financial institution staff	○	○	○	○	○

4. The following people should be aware that the person who is confused or out of touch with reality may be frightened by their thoughts and feelings.

	Essential	Important	Don't know/Depends	Unimportant	Should not be included
Financial counsellor	○	○	○	○	○
Financial institution staff	○	○	○	○	○

5. The following people should tailor their approach and interaction to the way the person who is confused or out of touch with reality is behaving, e.g. if the person is suspicious, be sensitive to this and reassure them you are there to assist them.

	Essential	Important	Don't know/Depends	Unimportant	Should not be included
Financial counsellor	○	○	○	○	○
Financial institution staff	○	○	○	○	○

6. Do you have any comments on these statements? Is there anything you would like to add to this section? Please write your suggestions in the box provided.

7. The financial counsellor should be aware that delusions and hallucinations are very real to the person. They should not:

	Essential	Important	Don't know/Depends	Unimportant	Should not be included
Dismiss, minimise or argue with the person about their confusion, delusions or hallucinations.	○	○	○	○	○
Act alarmed, horrified or embarrassed by the person's confusion, delusions or hallucinations.	○	○	○	○	○
Laugh at the person's confusion, delusions or hallucinations.	○	○	○	○	○
Encourage or inflame the person's paranoia, if the person exhibits paranoid behaviour.	○	○	○	○	○

Other (please specify)

8. The financial institution staff should be aware that delusions and hallucinations are very real to the person. They should not:

	Essential	Important	Don't know/Depends	Unimportant	Should not be included
Dismiss, minimise or argue with the person about their confusion, delusions or hallucinations.					
Act alarmed, horrified or embarrassed by the person’s confusion, delusions or hallucinations.					
Laugh at the person’s confusion, delusions or hallucinations.					
Encourage or inflame the person’s paranoia, if the person exhibits paranoid behaviour.					

Other (please specify)

9. When talking to the person who is confused or out of touch with reality, the financial counsellor should:

	Essential	Important	Don't know/Depends	Unimportant	Should not be included
Respond to the person’s delusions without agreeing with them, by saying something like “that must be horrible for you” or “I can see that you are upset”.					
Respond to disorganised speech by communicating in an uncomplicated and succinct manner.					
Repeat things if necessary.					
Be patient and allow plenty of time for the person to process the information and respond to what has been said.					
Not assume the person cannot understand what is being said, even if their response is limited.					

Other (please specify)

10. When talking to the person who is confused or out of touch with reality, the financial institution staff should:

	Essential	Important	Don't know/Depends	Unimportant	Should not be included
Respond to the person's delusions without agreeing with them, by saying something like "that must be horrible for you" or "I can see that you are upset".	○	○	○	○	○
Respond to disorganised speech by communicating in an uncomplicated and succinct manner.	○	○	○	○	○
Repeat things if necessary.	○	○	○	○	○
Be patient and allow plenty of time for the person to process the information and respond to what has been said.	○	○	○	○	○
Not assume the person cannot understand what is being said, even if their response is limited.	○	○	○	○	○

Other (please specify)

11. The following people should be aware that the person's confusion and fear about what is happening to them may lead them to deny that anything is wrong with them.

	Essential	Important	Don't know/Depends	Unimportant	Should not be included
Financial counsellor	○	○	○	○	○
Financial institution staff	○	○	○	○	○

12. When the person is denying that anything is wrong, the following people should encourage them to talk to someone they trust.

	Essential	Important	Don't know/Depends	Unimportant	Should not be included
Financial counsellor	○	○	○	○	○
Financial institution staff	○	○	○	○	○

13. Do you have any comments on these statements? Is there anything you would like to add to this section? Please write your suggestions in the box provided.

Section 11: Policy and procedures

This section contains statements about policies and procedures relevant to working with people with mental health problems and financial difficulties which may be adopted by **financial counselling and mental health services**.

Please rate how important (from 'essential' to 'should not be included') you think it is that each statement be included in the guidelines.

Please keep the definitions in mind when answering the questions.

1. Financial counsellors should receive basic mental health training, e.g. mental health first aid. (Mental health first aid is the help offered a person developing to a mental health problem or experiencing a mental health crisis. The first aid is given until appropriate professional help is received or until the crisis resolves.)

Essential	Important	Don't know/Depends	Unimportant	Should not be included
○	○	○	○	○

2. Financial counsellors should be required to have continuing professional development that includes mental health training.

Essential	Important	Don't know/Depends	Unimportant	Should not be included
○	○	○	○	○

3. Do you have any comments on these statements? Is there anything you would like to add to this section? Please write your suggestions in the box provided.

4. Mental health professionals should have procedures for prompt referral to financial counselling services of people with financial difficulties.

Essential	Important	Don't know/Depends	Unimportant	Should not be included
○	○	○	○	○

5. Mental health professionals should be trained in basic financial literacy skills.

Essential	Important	Don't know/Depends	Unimportant	Should not be included
○	○	○	○	○

6. <u>Mental health professionals</u> should be required to have financial literacy skills training as part of their continuing professional development.

Essential	Important	Don't know/Depends	Unimportant	Should not be included
○	○	○	○	○

7. Do you have any comments on these statements? Is there anything you would like to add to this section? Please write your suggestions in the box provided.

Section 11 (cont): Policy and procedures

This section contains statements about policies and procedures relevant to working with people with mental health problems and financial difficulties which may be adopted by **financial institutions**.

Please rate how important (from 'essential' to 'should not be included') you think it is that each statement be included in the guidelines.

Please keep the definitions in mind when answering the questions.

On this page the term 'customer' refers to all the people a financial institution serves and 'the person' is the customer with mental health problems and financial difficulties.

1. The financial institution should have specific policies around credit listing for people with mental health problems. ('Credit list' means to report the debt to a credit listing agency who would record a default against the person. These records are routinely checked by lenders prior to approving credit.)

Essential	Important	Don't know/Depends	Unimportant	Should not be included
○	○	○	○	○

2. Financial institutions should have a policy for working with people with mental health problems and financial difficulties.

Essential	Important	Don't know/Depends	Unimportant	Should not be included
○	○	○	○	○

3. Financial institutions should make their policy for working with people with mental health problems and financial difficulties available to financial counsellors.

Essential	Important	Don't know/Depends	Unimportant	Should not be included
○	○	○	○	○

4. Financial institutions should make their policy on working with people with mental health problems and financial difficulties publically available.

Essential	Important	Don't know/Depends	Unimportant	Should not be included
○	○	○	○	○

5. Financial institutions should monitor the implementation of mental health problem policies to ensure they are meeting their aims, e.g. number of people who disclose, types of conditions disclosed, broken arrangements involving people who disclose, use of specialist team.

Essential	Important	Don't know/Depends	Unimportant	Should not be included
○	○	○	○	○

6. Do you have any comments on these statements? Is there anything you would like to add to this section? Please write your suggestions in the box provided.

7. Financial institutions should have specific procedures for working with the person with mental health problems in complex or sensitive situations, e.g. suicidal thoughts and behaviours, threats to harm others, sale of a family home.

Essential	Important	Don't know/Depends	Unimportant	Should not be included
○	○	○	○	○

8. These procedures should include:

	Essential	Important	Don't know/Depends	Unimportant	Should not be included
Having a process to redirect the person away from the usual collections process to an appropriately skilled team/staff member.	○	○	○	○	○
Having a process for handing on calls when the staff member requires support to manage the situation.	○	○	○	○	○
Having a review panel with a range of expertise to ensure all appropriate options are considered.	○	○	○	○	○
Having a process for working with crisis situations, e.g. threat of harm to self or others.	○	○	○	○	○
Having processes for referring the person to mental health services.	○	○	○	○	○
Having processes for referring the person to financial counselling services.	○	○	○	○	○

Other (please specify)

9. <u>Financial institutions</u> should be flexible in their arrangements with people experiencing mental health problems and financial difficulties, e.g. changing payment arrangements as required, accepting oral rather than written confirmation.

Essential	Important	Don't know/Depends	Unimportant	Should not be included
○	○	○	○	○

10. For people with mental health problems, <u>financial institutions</u> should have a tailored procedure for reviewing and taking action at key points in the collection process, e.g. sale of assets or sale of debt.

Essential	Important	Don't know/Depends	Unimportant	Should not be included
○	○	○	○	○

11. The tailored procedure should include the following options:

	Essential	Important	Don't know/Depends	Unimportant	Should not be included
Longer time lines for repayment or settlement of debt.	○	○	○	○	○
Longer time line to vacate property.	○	○	○	○	○
Not selling debt to third parties.	○	○	○	○	○
Encouraging third party representation (e.g. a professional, such as a community lawyer or financial counsellor) advocating on their behalf.	○	○	○	○	○
Making a site visit to assess the situation before proceeding with sale of property.	○	○	○	○	○
Referring action when the person is acutely unwell or in a mental health crisis.	○	○	○	○	○
Suspending interest payments when the person is acutely unwell or in a mental health crisis.	○	○	○	○	○

12. Do you have any comments on these statements? Is there anything you would like to add to this section? Please write your suggestions in the box provided.

Section 11 (cont): Policy and procedures

This section contains statements about policies and procedures relevant to working with people with mental health problems and financial difficulties which may be adopted by **financial institutions**.

Please rate how important (from 'essential' to 'should not be included') you think it is that each statement be included in the guidelines.

Please keep the definitions in mind when answering the questions.

On this page the term 'customer' refers to all the people a financial institution serves and 'the person' is the customer with mental health problems and financial difficulties.

1. Financial institutions should provide a single point of contact for the person with mental health problems for the duration of the financial difficulties.

Essential	Important	Don't know/Depends	Unimportant	Should not be included
○	○	○	○	○

2. Financial institutions should provide a single point of contact for the person with severe mental illness for the duration of the financial difficulties. (A severe mental illness is defined as a mental illness that has a significant disabling affect on the person's ability to work, look after themselves, and carry on relationships with family and friends. It is considered to be a long-term illness.)

Essential	Important	Don't know/Depends	Unimportant	Should not be included
○	○	○	○	○

3. Financial institutions should provide a dedicated phone number for the person with mental health problems and financial difficulties and their authorised representatives.

Essential	Important	Don't know/Depends	Unimportant	Should not be included
○	○	○	○	○

4. Financial institutions should provide a specialised dedicated team for working with the person with mental health problems and financial difficulties.

Essential	Important	Don't know/Depends	Unimportant	Should not be included
○	○	○	○	○

5. Financial institutions should allow for extra time to work with people with mental health problems, e.g. longer conversations, additional time to provide written confirmation of communication, more frequent calls.

Essential	Important	Don't know/Depends	Unimportant	Should not be included
○	○	○	○	○

6. Do you have any comments on these statements? Is there anything you would like to add to this section? Please write your suggestions in the box provided.

7. The hardship team should have access to a mental health professional who they can consult with to give better customer care.

Essential	Important	Don't know/Depends	Unimportant	Should not be included
○	○	○	○	○

8. Financial institutions that use specialist teams to work with people with mental health problems should also consider how the expertise that members of these teams possess can be shared with other colleagues via informal/semi-formal, in-house training courses.

Essential	Important	Don't know/Depends	Unimportant	Should not be included
○	○	○	○	○

9. Do you have any comments on these statements? Is there anything you would like to add to this section? Please write your suggestions in the box provided.

10. Financial institutions should have a process for training frontline/branch staff to identify people with mental health problems and financial difficulties and refer them to a specialised team within the bank, e.g. hardship team.

Essential	Important	Don't know/Depends	Unimportant	Should not be included
○	○	○	○	○

11. Financial institutions should provide their staff with mental health awareness training that is tailored to their role and the amount of contact they are likely to have with people with mental health problems.

Essential	Important	Don't know/Depends	Unimportant	Should not be included
○	○	○	○	○

12. Financial institutions should provide tailored mental health training for collections call centre staff.

Essential	Important	Don't know/Depends	Unimportant	Should not be included
○	○	○	○	○

13. Financial institutions should provide tailored mental health training for hardship staff.

Essential	Important	Don't know/Depends	Unimportant	Should not be included
○	○	○	○	○

14. Financial institutions should provide training for their staff on how to assist someone over the phone who is having a mental health crisis.

Essential	Important	Don't know/Depends	Unimportant	Should not be included
○	○	○	○	○

15. Financial institutions should ensure that any mental health training received by financial institution staff is revisited periodically.

Essential	Important	Don't know/Depends	Unimportant	Should not be included
○	○	○	○	○

16. Do you have any comments on these statements? Is there anything you would like to add to this section? Please write your suggestions in the box provided.

17. In providing relevant information about financial hardship to their customers, financial institutions should include mental illness as a possible contributor to financial difficulties.

Essential	Important	Don't know/Depends	Unimportant	Should not be included
○	○	○	○	○

18. Financial institutions should have a mental health advisory committee to review relevant information for customers (e.g. financial education, hardship literature) to ensure it is appropriate and non-stigmatising.

Essential	Important	Don't know/Depends	Unimportant	Should not be included
○	○	○	○	○

19. Financial institutions should publicise and share examples of good practice from within their own organisation with colleagues across the industry.

Essential	Important	Don't know/Depends	Unimportant	Should not be included
○	○	○	○	○

20. Financial institutions should work with state and national financial counselling organisations in order to develop policies to better assist people with mental health problems and financial difficulties.

Essential	Important	Don't know/Depends	Unimportant	Should not be included
○	○	○	○	○

21. Financial institutions should work with state and national mental health organisations in order to develop policies that better assist people with mental health problems and financial difficulties.

Essential	Important	Don't know/Depends	Unimportant	Should not be included
○	○	○	○	○

22. Financial institutions should work with community services organisations in order to develop policies that better assist people with mental health problems and financial difficulties.

Essential	Important	Don't know/Depends	Unimportant	Should not be included
◯	◯	◯	◯	◯

23. Do you have any comments on these statements? Is there anything you would like to add to this section? Please write your suggestions in the box provided.

One final item

If participating in this survey has caused you distress and you wish to talk to someone about this you can contact Kathy Bond, MHFA Researcher on 03 9079 0200. Kathy can provide you with a list of people or places that can help you with your distress. Alternatively, you can contact Lifeline on 13 11 14.

That is the end of the first round survey!

Thank you very much for your contribution.

By pressing the "next" button your final responses will be registered with our survey software. Once all panel members have lodged their responses, we will collate the data and send you a report on the findings and the second survey.

We are extremely grateful for your contribution.

The Mental Health First Aid Research Team
