# Supplementary material for: Assisting Australians with mental health problems and financial difficulties: a Delphi study to develop guidelines for financial counsellors, financial institution staff, mental health professionals and carers
Source: BMC Health Serv Res. 2015 Jun 3;15:218. doi: 10.1186/s12913-015-0868-2 (PMC4453216; doi:10.1186/s12913-015-0868-2)
Supplement: Additional file 2: — Copy of Round 2 survey. [file 12913_2015_868_MOESM2_ESM.pdf]

Helping the person with mental health problems and financial difficulties -

Introduction and Instructions

1. Purpose of the research

Mental Health First Aid (MHFA) is the help provided to a person developing a mental health problem or in a mental health crisis. The first aid is given until appropriate professional treatment is received or until the crisis resolves. The purpose of this project is to develop guidelines for offering appropriate mental health first aid to the person who is experiencing mental health problems and financial difficulties.

This research project is funded by the Australian Government Department of Health and Ageing.

2. Your role in Round Two

Your task is to rate the statements presented in this questionnaire according to how important you believe they are for providing guidance to financial counsellors, financial institution staff, mental health professionals and the support person who is assisting the person experiencing mental health problems and financial difficulties.

3. How this Round Two questionnaire was developed

Statements in this questionnaire were developed in the following ways:

Re-rate items: These are items from Round 1 that did not receive a level of consensus that was either high enough to be clearly endorsed or low enough to be clearly rejected. These items are marked as a rerate item.

New items: These items appear for the first time in Round 2 and have been developed from comments made by participants in Round 1.

Altered items: These items were not endorsed, but were altered according to panel comments about aspects of the wording of the original items.

It is important to note that we do not necessarily agree with these statements; we have included them because we do not believe that we should decide what the best practice is in this area. Rather, we have invited you, a member of the expert panel, to help develop a set of guidelines that reflect current expert opinion.

4. Instructions

Please complete the questionnaire by rating each statement according to how important you believe it is for inclusion in guidelines for offering mental health first aid to the person experiencing mental health problems and financial difficulties. This questionnaire should take approximately 45 to 60 minutes to complete. If necessary, you can complete the survey in two or more sittings. Your answers are saved when you click 'Next' at the bottom of a page. This marks your page and you can begin again at a later date on the next page. Please be aware that once you have logged on and started responding you must complete the questionnaire on this same computer.

5. Consent to participate

It is important for you to know that participation in this study is completely voluntary. You are not under any obligation to participate and you can withdraw at any time. Submitting your questionnaire is an indication of your understanding of this and your consent to participate in the study.

We would like to thank you for your time and effort and encourage you to provide us with feedback on this process.

Best Wishes,

The Mental Health First Aid Research Team

Helping the person with mental health problems and financial difficulties -

Introduction and Instructions

Definitions

These terms may have different meaning for the groups represented in this survey (i.e. financial counsellors, financial institution staff, consumers, carers and mental health professionals). When answering these questions, please ensure you use our definitions only.

'The person', unless otherwise stated, refers to the person with *mental health problems and financial difficulties*. This person may be called a customer, client, patient, consumer, relative or friend.

'Mental illness' is a diagnosable illness that affects a person's thinking, emotional state and behaviour, and disrupts the person's ability to work or carry out other daily activities and engage in satisfying personal relationships.

'Mental health problems' is a broader term including mental illnesses, symptoms of mental illnesses that may not be severe enough to warrant the diagnosis of a mental illness, as well as mental health-related crises such as having thoughts of suicide. For the purposes of this survey, problem gambling has been excluded from mental health problems, as this may be the subject of a future specialised project.

'Financial difficulties' are when income is insufficient to cover reasonable living expenses and meet financial commitments as they become due. This definition covers people in financial hardship, as well as those experiencing other financial challenges. This does not include when a payment has simply been overlooked.

'The support person' refers to someone who is a significant source of emotional and/or practical support or assistance to 'the person'. It may include a partner, close friend or family member. However, it is not assumed that every person has a support person.

'Authorised third party representative' refers to anyone who has been appointed, in writing or orally, by 'the person' to act on their behalf regarding financial decisions.

'The financial counsellor' is a trained professional who assists people who are experiencing financial difficulties. Most financial counsellors work in community organisations, although some work in government agencies and local government. Financial counsellors provide a free, confidential and independent service. The financial counsellor is not a financial planner, financial adviser or legal practitioner and is not a general welfare worker.

'Financial institution' is any institution that is in the position to collect money from individuals, including banks or those who act on their behalf.

'Financial institution staff' are employees of financial institutions that work with the person with mental health problems and financial difficulties.

'Hardship team' is a financial institution's team that is trained to provide financial solutions for people experiencing financial difficulties/hardship.

'Hardship' is defined as when 'the person' is being managed by a financial institution's hardship team.

'Collections staff' are financial institution staff whose primary duty is to negotiate payment from people with overdue accounts.

'Branch staff' are financial institution staff who work in the local bank branches.

'Contact/call centre staff' are financial institution staff who work with customers over the phone. They can be located overseas or in Australian.

Helping the person with mental health problems and financial difficulties -

‘The mental health professional’ refers to a trained health professional who is treating/working with 'the person'. This could be a psychologist, mental health nurse or psychiatrist. It could also be a social worker, occupational therapist or GP who has specialist mental health training.

Information about you

*1. Please enter you participant code.

Helping the person with mental health problems and financial difficulties -

Section 1: General awareness about mental health problems and financial di...

This section asks you about what information **the financial counsellor** and **the financial institution staff** should know about mental health problems and financial difficulties.

Please rate how important (from 'essential' to 'should not be included') you think it is that each statement be included in the guidelines for each of the groups listed in the left hand column.

***2. The following people should be aware that people with mental health problems can be intelligent, capable people going through a difficult time, like many other people in the community.**

(New item)

	Essential	Important	Don't know/Depends	Unimportant	Should not be included
Financial counsellor	◯	◯	◯	◯	◯
Financial institution hardship staff	◯	◯	◯	◯	◯
Financial institution collection staff	◯	◯	◯	◯	◯
Financial institution branch staff	◯	◯	◯	◯	◯
Financial institution contact/call centre staff	◯	◯	◯	◯	◯

***3. The following financial institution staff should be aware of the signs and symptoms of mental illnesses. (*Please note that this does not mean knowing how to diagnose a mental illness.*)**

(Altered item)

	Essential	Important	Don't know/Depends	Unimportant	Should not be included
Hardship staff	◯	◯	◯	◯	◯
Collection staff	◯	◯	◯	◯	◯
Branch staff	◯	◯	◯	◯	◯
Contact/call centre staff	◯	◯	◯	◯	◯

***4. The following financial institution staff should be aware of the types of mental illnesses. (*<u>Please note that this does not mean knowing how to diagnose a mental illness.</u>*)**

(Altered item)

	Essential	Important	Don't know/Depends	Unimportant	Should not be included
Hardship staff	○	○	○	○	○
Collection staff	○	○	○	○	○
Branch staff	○	○	○	○	○
Contact/call centre staff	○	○	○	○	○

***5. The following financial institution staff should be aware of the risk factors for suicide.**

(Altered item)

	Essential	Important	Don't know/Depends	Unimportant	Should not be included
Hardship staff	○	○	○	○	○
Collection staff	○	○	○	○	○
Branch staff	○	○	○	○	○
Contact/call centre staff	○	○	○	○	○

***6. The following financial institution staff should be aware of the range of mental illness treatments and their effects. (*<u>Please note that this does not mean knowing how to diagnose a mental illness.</u>*)**

(Altered item)

	Essential	Important	Don't know/Depends	Unimportant	Should not be included
Hardship staff	○	○	○	○	○
Collection staff	○	○	○	○	○
Branch staff	○	○	○	○	○
Contact/call centre staff	○	○	○	○	○

***7. The following financial institution staff should be aware of the things which might indicate that the person has mental health problems, e.g. not attending appointments, not returning phone calls, not meeting repayment schedules, displaying unusual behaviours, lack of clarity of thought. (*Please note that this does not mean knowing how to diagnose a mental illness.*)**

(Altered item)

	Essential	Important	Don't know/Depends	Unimportant	Should not be included
Hardship staff	○	○	○	○	○
Collection staff	○	○	○	○	○
Branch staff	○	○	○	○	○
Contact/call centre staff	○	○	○	○	○

***8. The following people should be aware of the importance of early identification and intervention for preventing or limiting relapse in the person with mental health problems. (*Please note that this does not mean knowing how to diagnose a mental illness.*)**

(Altered item)

	Essential	Important	Don't know/Depends	Unimportant	Should not be included
Financial counsellor	○	○	○	○	○
Financial institution hardship staff	○	○	○	○	○
Financial institution collection staff	○	○	○	○	○
Financial institution branch staff	○	○	○	○	○
Financial institution contact/call centre staff	○	○	○	○	○

***9. The following financial institution staff should be aware that the level of support needed by the person with mental health problems will fluctuate, as the symptoms of most mental health problems come and go over time.**

(Altered item)

	Essential	Important	Don't know/Depends	Unimportant	Should not be included
Hardship staff	○	○	○	○	○
Collection staff	○	○	○	○	○
Branch staff	○	○	○	○	○
Contact/call centre staff	○	○	○	○	○

***10. The following people should be aware of how they can reduce stressors that can contribute to relapse, i.e. a reoccurrence of a mental health problem in a person who is currently well.**

(Altered item)

	Essential	Important	Don't know/Depends	Unimportant	Should not be included
Financial counsellor	◯	◯	◯	◯	◯
Financial institution hardship staff	◯	◯	◯	◯	◯
Financial institution collection staff	◯	◯	◯	◯	◯
Financial institution branch staff	◯	◯	◯	◯	◯
Financial institution contact/call centre staff	◯	◯	◯	◯	◯

***11. The following financial institution staff should know how they can reduce stressors that may exacerbate the person's mental health problems.**

(Altered item)

	Essential	Important	Don't know/Depends	Unimportant	Should not be included
Hardship staff	◯	◯	◯	◯	◯
Collection staff	◯	◯	◯	◯	◯
Branch staff	◯	◯	◯	◯	◯
Contact/call centre staff	◯	◯	◯	◯	◯

Section 1 (cont): General awareness about mental health problems and finan...

This section asks you about what information **the financial counsellor** and **the financial institution staff** should know about mental health problems and financial difficulties.

Please rate how important (from 'essential' to 'should not be included') you think it is that each statement be included in the guidelines for each of the groups listed in the left hand column.

***12. The following financial institution staff should be aware that mental health problems may take time, usually weeks or months, to develop.**

(New item)

	Essential	Important	Don't know/Depends	Unimportant	Should not be included
Hardship staff	◯	◯	◯	◯	◯
Collection staff	◯	◯	◯	◯	◯
Branch staff	◯	◯	◯	◯	◯
Contact/call centre staff	◯	◯	◯	◯	◯

***13. The following people should be aware that mental health problems can develop or worsen rapidly.**

(New item)

	Essential	Important	Don't know/Depends	Unimportant	Should not be included
Financial counsellor	◯	◯	◯	◯	◯
Financial institution hardship staff	◯	◯	◯	◯	◯
Financial institution collection staff	◯	◯	◯	◯	◯
Financial institution branch staff	◯	◯	◯	◯	◯
Financial institution contact/call centre staff	◯	◯	◯	◯	◯

***14. The financial counsellor should be familiar with the following mental health first aid guidelines:**

	Essential	Important	Don't know/Depends	Unimportant	Should not be included
How to help a person having a panic attack<br><br>**(Re-rate item)**	○	○	○	○	○
How to help a person with problem drug use<br><br>**(Re-rate item)**	○	○	○	○	○
How to help a person with problem drinking<br><br>**(Re-rate item)**	○	○	○	○	○

***15. The following financial institution staff should be familiar with the mental health first aid guidelines for psychosis.**

(Altered item)

	Essential	Important	Don't know/Depends	Unimportant	Should not be included
Hardship staff	○	○	○	○	○
Collection staff	○	○	○	○	○
Branch staff	○	○	○	○	○
Contact/call centre staff	○	○	○	○	○

***16. The following financial institution staff should be familiar with the mental health first aid guidelines for panic attack.**

(Altered item)

	Essential	Important	Don't know/Depends	Unimportant	Should not be included
Hardship staff	○	○	○	○	○
Collection staff	○	○	○	○	○
Branch staff	○	○	○	○	○
Contact/call centre staff	○	○	○	○	○

Section 1 (cont): General awareness about mental health problems and finan...

This section asks you about what information **the financial institution staff** should know about mental health problems and financial difficulties.

Please rate how important (from 'essential' to 'should not be included') you think it is that each statement be included in the guidelines for each of the groups listed in the left hand column.

***17. The following financial institution staff should be aware of how the stigmatising attitudes of others can have a negative impact on the person with mental health problems.**

(Altered item)

	Essential	Important	Don't know/Depends	Unimportant	Should not be included
Hardship staff	○	○	○	○	○
Collection staff	○	○	○	○	○
Branch staff	○	○	○	○	○
Contact/call centre staff	○	○	○	○	○

***18. The following financial institution staff should be aware of the potential effects of any stigmatising attitudes the financial institution staff may have on the person with mental health problems.**

(Altered item)

	Essential	Important	Don't know/Depends	Unimportant	Should not be included
Hardship staff	○	○	○	○	○
Collection staff	○	○	○	○	○
Branch staff	○	○	○	○	○
Contact/call centre staff	○	○	○	○	○

***19. The following financial institution staff should be aware of how the person's own stigmatising attitudes about their illness may impact on their ability to deal with financial difficulties.**

(Altered item)

	Essential	Important	Don't know/Depends	Unimportant	Should not be included
Hardship staff	○	○	○	○	○
Collection staff	○	○	○	○	○
Branch staff	○	○	○	○	○
Contact/call centre staff	○	○	○	○	○

***20. The following financial institution staff should be aware of the myths surrounding mental health problems that lead to stigma and limit the potential earning capabilities of the person affected by mental health problems.**

(Altered item)

	Essential	Important	Don't know/Depends	Unimportant	Should not be included
Hardship staff	○	○	○	○	○
Collection staff	○	○	○	○	○
Branch staff	○	○	○	○	○
Contact/call centre staff	○	○	○	○	○

Section 1 (cont): General awareness about mental health problems and finan...

This section asks you about what information **the financial counsellor** and **the financial institution staff** should know about mental health problems and financial difficulties.

Please rate how important (from 'essential' to 'should not be included') you think it is that each statement be included in the guidelines for each of the groups listed in the left hand column.

***21. The following financial institution staff should know that the cycle of financial difficulties and mental health problems can be intensified for people from culturally and linguistically diverse communities, due to the difficulty of navigating unfamiliar financial and health systems in Australia.**

(Altered item)

	Essential	Important	Don't know/Depends	Unimportant	Should not be included
Hardship staff	○	○	○	○	○
Collection staff	○	○	○	○	○
Branch staff	○	○	○	○	○
Contact/call centre staff	○	○	○	○	○

***22. The following people should be familiar with cultural considerations when working with a person with a mental health problem who is from an Aboriginal or Torres Strait Islander background.**

(New item)

	Essential	Important	Don't know/Depends	Unimportant	Should not be included
Financial counsellor	○	○	○	○	○
Financial institution hardship staff	○	○	○	○	○
Financial institution collection staff	○	○	○	○	○
Financial institution branch staff	○	○	○	○	○
Financial institution contact/call centre staff	○	○	○	○	○

Section 1 (cont): General awareness about mental health problems and finan...

This section asks you about what information **the financial counsellor** and **the financial institution staff** should know about mental health problems and financial difficulties.

Please rate how important (from 'essential' to 'should not be included') you think it is that each statement be included in the guidelines for each of the groups listed in the left hand column.

*23. The following people should be aware that dealing with financial matters (e.g. opening mail from creditors, visiting Centrelink) may be overwhelming for the person.

(New item)

	Essential	Important	Don't know/Depends	Unimportant	Should not be included
Financial counsellor	○	○	○	○	○
Financial institution hardship staff	○	○	○	○	○
Financial institution collection staff	○	○	○	○	○
Financial institution branch staff	○	○	○	○	○
Financial institution contact/call centre staff	○	○	○	○	○

*24. The following financial institution staff should know that the person with financial difficulties may find it difficult to pay for treatment for their mental health problems, which may worsen their condition.

(Altered item)

	Essential	Important	Don't know/Depends	Unimportant	Should not be included
Hardship staff	○	○	○	○	○
Collection staff	○	○	○	○	○
Branch staff	○	○	○	○	○
Contact/call centre staff	○	○	○	○	○

***25. The following financial institution staff should know that the way the person perceives their financial situation is what impacts on their mental health, rather than their actual situation.**

(Altered item)

	Essential	Important	Don't know/Depends	Unimportant	Should not be included
Hardship staff	○	○	○	○	○
Collection staff	○	○	○	○	○
Branch staff	○	○	○	○	○
Contact/call centre staff	○	○	○	○	○

***26. The following financial institution staff should know that the person with mental health problems may incur debt to support a drug or alcohol problem.**

(Altered item)

	Essential	Important	Don't know/Depends	Unimportant	Should not be included
Hardship staff	○	○	○	○	○
Collection staff	○	○	○	○	○
Branch staff	○	○	○	○	○
Contact/call centre staff	○	○	○	○	○

***27. The following financial institution staff should be aware that the person with mental health problems may not overspend but may have other problems with managing money, e.g. withdrawing all their money from the bank to keep it 'safe' at home.**

(Altered item)

	Essential	Important	Don't know/Depends	Unimportant	Should not be included
Hardship staff	○	○	○	○	○
Collection staff	○	○	○	○	○
Branch staff	○	○	○	○	○
Contact/call centre staff	○	○	○	○	○

***28. The following financial institution staff should know that financial stability may not be an important goal for some people in the early stages of recovery from mental illness.**

(Altered item)

	Essential	Important	Don't know/Depends	Unimportant	Should not be included
Hardship staff	○	○	○	○	○
Collection staff	○	○	○	○	○
Branch staff	○	○	○	○	○
Contact/call centre staff	○	○	○	○	○

Section 1 (cont): General awareness about mental health problems and finan...

This section asks you about what information **the mental health professional** and **the support person** should know about mental health problems and financial difficulties.

Please rate how important (from 'essential' to 'should not be included') you think it is that each statement be included in the guidelines.

***29. The mental health professional should be aware that if the person's financial difficulties are addressed then their mental health may improve.**

(New item)

Essential	Important	Don't know/Depends	Unimportant	Should not be included
○	○	○	○	○

***30. The mental health professional should be aware of what a financial administration order is and the implications for the client.**

(Re-rate item)

Essential	Important	Don't know/Depends	Unimportant	Should not be included
○	○	○	○	○

***31. The support person should know that many people have limited financial literacy skills.**

(Re-rate item)

Essential	Important	Don't know/Depends	Unimportant	Should not be included
○	○	○	○	○

Section 1 (cont): General awareness about mental health problems and finan...

This section asks you about what information **the financial counsellor** and **the financial institution staff** should know about mental health problems and financial difficulties.

Please rate how important (from 'essential' to 'should not be included') you think it is that each statement be included in the guidelines for each of the groups listed in the left hand column.

***32. The following people should have a *thorough working knowledge* of their state or territory laws regarding the appointment of a third party to manage the person's finances, e.g. guardianship, administration orders, financial management orders.**

(New item)

	Essential	Important	Don't know/Depends	Unimportant	Should not be included
Financial counsellor	○	○	○	○	○
Mental health professional	○	○	○	○	○
Financial institution hardship staff	○	○	○	○	○
Financial institution collection staff	○	○	○	○	○
Financial institution branch staff	○	○	○	○	○
Financial institution contact/call centre staff	○	○	○	○	○

***33. The following people should have a *general understanding* of their state or territory laws regarding the appointment of a third party to manage the person's finances, e.g. guardianship, administration orders, financial management orders.**

(New item)

	Essential	Important	Don't know/Depends	Unimportant	Should not be included
Financial counsellor	○	○	○	○	○
Mental health professional	○	○	○	○	○
Financial institution hardship staff	○	○	○	○	○
Financial institution collection staff	○	○	○	○	○
Financial institution branch staff	○	○	○	○	○
Financial institution contact/call centre staff	○	○	○	○	○

Section 2: Specific mental health problems

This section asks you about what information **the financial counsellor and the financial institution staff** should know about specific mental health problems and financial difficulties.

Please rate how important (from 'essential' to 'should not be included') you think it is that each statement be included in the guidelines.

***34. The financial counsellor should encourage the person with bipolar disorder to delay making large purchases until they have discussed them with their support person.**

(Re-rate item)

Essential	Important	Don't know/Depends	Unimportant	Should not be included
○	○	○	○	○

***35. The following financial institution staff should be aware of how an episode of mania may affect a person's financial circumstances, e.g. not paying bills, job instability, over-spending.**

(Altered item)

	Essential	Important	Don't know/Depends	Unimportant	Should not be included
Hardship staff	○	○	○	○	○
Collection staff	○	○	○	○	○
Branch staff	○	○	○	○	○
Contact/call centre staff	○	○	○	○	○

Section 3: Screening and referral

This section asks you about what information **the financial counsellor** and **the financial institution staff** need to know in order to refer the person to mental health services, and **the mental health professional** and **the financial institution staff** need to know in order to refer the person to financial counselling services.

Please rate how important (from 'essential' to 'should not be included') you think it is that each statement be included in the guidelines.

***36. The financial counsellor should ask all new clients if they or anyone in their family suffers from any illnesses as a subtle way of asking about mental health problems.**

(New item)

Essential	Important	Don't know/Depends	Unimportant	Should not be included
○	○	○	○	○

***37. When the financial counsellor is completing a *'client service plan'* with the person, they should take note of any medications the person takes as this may indicate if they have mental health problems. (A *'client service plan'* is a document that financial counsellors develop people to identify financial issues and possible pathways for options and solutions to financial difficulties.)**

(New item)

Essential	Important	Don't know/Depends	Unimportant	Should not be included
○	○	○	○	○

***38. If the person discloses mental health problems and *asks for help*, the financial counsellor should**

(Altered item):

	Essential	Important	Don't know/Depends	Unimportant	Should not be included
Refer the client to appropriate professional help.	○	○	○	○	○
Provide written material about appropriate professional help.	○	○	○	○	○

***39. If it is suspected that the person in financial difficulties may have mental health problems, the following financial institution staff should refer them to financial counselling services, who can advocate on the person's behalf and make an appropriate referral.**

(Altered item)

	Essential	Important	Don't know/Depends	Unimportant	Should not be included
Hardship staff	◯	◯	◯	◯	◯
Collection staff	◯	◯	◯	◯	◯
Branch staff	◯	◯	◯	◯	◯
Contact/call centre staff	◯	◯	◯	◯	◯

***40. If it is suspected that the person in *<u>financial difficulties</u>* may have mental health problems, the the following financial institution staff should *<u>offer</u>* to refer them to financial counselling services, who can advocate on the person's behalf and make an appropriate referral.**

(Altered item)

	Essential	Important	Don't know/Depends	Unimportant	Should not be included
Hardship staff	◯	◯	◯	◯	◯
Collection staff	◯	◯	◯	◯	◯
Branch staff	◯	◯	◯	◯	◯
Contact/call centre staff	◯	◯	◯	◯	◯

***41. If the financial institution staff suspect that the person in *<u>financial hardship</u>* may have mental health problems, they should *<u>offer</u>* to refer them to financial counselling services, who can advocate on the person's behalf and make an appropriate referral. ('Hardship' is defined as when 'the person' is being managed by a financial institution's hardship team.)**

(Altered item)

Essential	Important	Don't know/Depends	Unimportant	Should not be included
◯	◯	◯	◯	◯

***42. The mental health professional and the person should agree on what information will be provided to the financial counsellor concerning how the person's mental health problems affect their ability to manage money or repay their debt.**

(Re-rate item)

Essential	Important	Don't know/Depends	Unimportant	Should not be included
○	○	○	○	○

***43. If the person is unable to talk to a financial counsellor (e.g. due to anxiety, speech or language difficulties), the mental health professional should**

(Altered item):

	Essential	Important	Don't know/Depends	Unimportant	Should not be included
Assist the person to identify priority debts (e.g. debts that can lead to loss of home, imprisonment or disconnection of utilities) so that the mental health professional can contact those creditors immediately.	○	○	○	○	○
Offer to accompany them to appointments with a financial counsellor.	○	○	○	○	○
Seek the advice of a financial counsellor themselves on behalf of the person, with the person's consent.	○	○	○	○	○

Section 4: Working together

This section asks you about what information **the financial counsellor, the financial institution staff, the mental health professional** and **the support person** should know about how to work together to get the best outcome for the person with mental health problems and financial difficulties.

Please rate how important (from 'essential' to 'should not be included') you think it is that each statement be included in the guidelines.

***44. The financial counsellor and the mental health professional should inform the person about the content of any discussion between them.**

(New item)

Essential	Important	Don't know/Depends	Unimportant	Should not be included
◯	◯	◯	◯	◯

***45. The person should be present at any meeting between the financial counsellor, the mental health professional and the support person.**

(New item)

Essential	Important	Don't know/Depends	Unimportant	Should not be included
◯	◯	◯	◯	◯

46. The financial counsellor should work with the mental health professional to determine whether the person is able to make specific decisions concerning their current financial situation, *with the person's consent.

(Altered item)

Essential	Important	Don't know/Depends	Unimportant	Should not be included
◯	◯	◯	◯	◯

***47. The financial counsellor should work with the mental health professional to determine whether the person is able to make specific decisions concerning their current financial situation.**

(Altered item)

Essential	Important	Don't know/Depends	Unimportant	Should not be included
◯	◯	◯	◯	◯

***48. The financial counsellor and the mental health professional should work together and deliver consistent messages to the person.**

(Re-rate item)

Essential	Important	Don't know/Depends	Unimportant	Should not be included
○	○	○	○	○

***49. The financial counsellor and the mental health professional should work together and deliver consistent messages to the person, *with the person's consent*.**

(Altered item)

Essential	Important	Don't know/Depends	Unimportant	Should not be included
○	○	○	○	○

***50. The financial counsellor and the mental health professional should work together and deliver consistent messages to the person.**

(Re-rate item)

Essential	Important	Don't know/Depends	Unimportant	Should not be included
○	○	○	○	○

***51. The financial counsellor and the mental health professional should work together so that the client service plan complements the treatment plan, *with the person's consent*. (A client service plan is a document that financial counsellors develop with people to identify financial issues and possible pathways for options and solutions to financial difficulties.)**

(Altered item)

Essential	Important	Don't know/Depends	Unimportant	Should not be included
○	○	○	○	○

***52. The financial counsellor and the mental health professional should be aware of and discuss any conflicting priorities between financial and treatment goals, *with the person's consent*.**

(Altered item)

Essential	Important	Don't know/Depends	Unimportant	Should not be included
○	○	○	○	○

***53. The financial counsellor and the mental health professional should be aware of and discuss any conflicting priorities between financial and treatment goals.**

(Re-rate item)

Essential	Important	Don't know/Depends	Unimportant	Should not be included
◯	◯	◯	◯	◯

***54. The financial counsellor and the mental health professional should communicate regularly about the person they are both working with, with the person's consent and with the person knowing the content of the conversation.**

(Altered item)

Essential	Important	Don't know/Depends	Unimportant	Should not be included
◯	◯	◯	◯	◯

***55. The financial counsellor, mental health professional and the support person should work with the person to determine what specific supports the person needs to enable them to make particular financial decisions.**

(Re-rate item)

Essential	Important	Don't know/Depends	Unimportant	Should not be included
◯	◯	◯	◯	◯

***56. The financial institution staff should work with the financial counsellor to determine whether the person is able to make specific decisions concerning their current financial situation, with the person's consent.**

(Altered item)

	Essential	Important	Don't know/Depends	Unimportant	Should not be included
Hardship staff	◯	◯	◯	◯	◯
Collection staff	◯	◯	◯	◯	◯
Branch staff	◯	◯	◯	◯	◯
Contact/call centre staff	◯	◯	◯	◯	◯

Section 6: Disclosure

This section asks you about what information **the financial counsellor**, and **the mental health professional** should know about disclosing mental health problems to a financial institution.

Please rate how important (from 'essential' to 'should not be included') you think it is that each statement be included in the guidelines.

***57. The financial counsellor should consider contacting the financial institution, with the person's consent, to explain how the mental health problems may affect the person's ability to manage their finances.**

(Re-rate item)

Essential	Important	Don't know/Depends	Unimportant	Should not be included
○	○	○	○	○

***58. The financial counsellor should advise the person that if they are more likely to overspend or sign up for credit cards when unwell, some financial institutions will add a note to their file, if requested.**

(Re-rate item)

Essential	Important	Don't know/Depends	Unimportant	Should not be included
○	○	○	○	○

***59. The financial counsellor should inform the person that although they may have disclosed their mental health problems to a financial institution, the financial institution staff may not necessarily understand the impact of mental health problems on financial difficulties.**

(Re-rate item)

Essential	Important	Don't know/Depends	Unimportant	Should not be included
○	○	○	○	○

***60. If the person decides to disclose their mental health problems to the financial institution, the financial counsellor should discuss with the person what to disclose.**

(New item)

Essential	Important	Don't know/Depends	Unimportant	Should not be included
○	○	○	○	○

***61. If the person decides to disclose their mental health problems to the financial institution, the mental health professional should discuss with the person what to disclose.**

(New item)

Essential	Important	Don't know/Depends	Unimportant	Should not be included
○	○	○	○	○

Section 7: Documentation

This section asks you about what information **the financial counsellor, financial institution staff** and **mental health professional** should know about documentation that might benefit the person with mental health problems and financial difficulties and assist in communication between the financial counsellor, the mental health professional and the financial institution.

Please rate how important (from 'essential' to 'should not be included') you think it is that each statement be included in the guidelines.

Advance Directive (An advance directive is a document describing how the person wants to be treated when they are unable to make their own decisions due to their present state of illness. Advance directives should specify the circumstances under which they will be used and who will take the various actions specified, e.g. when the person is becoming unwell or when they are acutely unwell.)

*62. The financial counsellor and the mental health professional should work together with the person to discuss the option of an advance directive, with the person's consent.

(New item)

Essential	Important	Don't know/Depends	Unimportant	Should not be included
○	○	○	○	○

*63. The following financial institution staff should know what an advance directive is and how to act upon it.

(Altered item)

	Essential	Important	Don't know/Depends	Unimportant	Should not be included
Hardship staff	○	○	○	○	○
Collection staff	○	○	○	○	○
Branch staff	○	○	○	○	○
Contact/call centre staff	○	○	○	○	○

*64. If a person has an advance directive in place, the hardship team should be the person's first point of call.

(New item)

Essential	Important	Don't know/Depends	Unimportant	Should not be included
○	○	○	○	○

Power of Attorney

***65. Where possible, the financial counsellor, mental health professional and support person should work together with the person and other appropriate professionals when making a decision about power of attorney.**

(Re-rate item)

Essential	Important	Don't know/Depends	Unimportant	Should not be included
○	○	○	○	○

Debt and Mental Illness Evidence Form

The following questions are about whether there should be a standardised Debt and Mental Illness Evidence Form that a mental health professional can use to communicate information about a person's mental health problems to financial counsellors and financial institutions. Such a form may benefit the person with mental health problems and financial difficulties in that it will allow the financial counsellor and the financial institution staff to find the most appropriate solution to the person's financial difficulties. It should be noted that the information on the form may be considered by the financial institution to determine the person's suitability for future borrowing.

***66. The following information should be included in a Debt and Mental Illness Evidence Form:**

	Essential	Important	Don't know/Depends	Unimportant	Should not be included
Details of the support the person is receiving with regards to their financial difficulties. **(Altered item)**	○	○	○	○	○
An option for the mental health professional to provide any additional relevant information. **(Re-rate item)**	○	○	○	○	○
Information about how any treatment (e.g. medication, ECT) may affect the person's comprehension. **(New item)**	○	○	○	○	○

***67. The financial counsellor should ask the person with mental health problems to obtain this completed form from their mental health professional, when proof of condition is required to obtain a particular outcome from the financial institution.**

(Re-rate item)

Essential	Important	Don't know/Depends	Unimportant	Should not be included
○	○	○	○	○

***68. A copy of the completed form should be given to the person (and their support person, where appropriate).**

(New item)

Essential	Important	Don't know/Depends	Unimportant	Should not be included
○	○	○	○	○

***69. The mental health professional should review this form when the person's mental health problems change and resubmit it if needed, *with the person's consent*.**

(Altered item)

Essential	Important	Don't know/Depends	Unimportant	Should not be included
○	○	○	○	○

***70. The financial institution should develop, in collaboration with mental health professionals and financial counsellors, a policy about which types of health professionals can provide evidence of the impact of mental health problems on financial difficulties.**

(Re-rate item)

Essential	Important	Don't know/Depends	Unimportant	Should not be included
○	○	○	○	○

***71. The financial institution should have a policy on how long the information on the debt and mental illness evidence form is held by the financial institution and this policy should be available to the person.**

(New item)

Essential	Important	Don't know/Depends	Unimportant	Should not be included
○	○	○	○	○

***72. The financial counsellor should receive training on any documents or forms used when working with people with mental health problems, e.g. debt and mental illness evidence form, advance directive.**

(New item)

Essential	Important	Don't know/Depends	Unimportant	Should not be included
○	○	○	○	○

Section 8: Actions to help the person

This section asks you about what information **the financial counsellor** should know about specific actions that can be taken to help the person with mental health problems and financial difficulties.

Please rate how important (from 'essential' to 'should not be included') you think it is that each statement be included in the guidelines.

***73. The financial counsellor should encourage the person to form a 'support network' of people who would be helpful in a variety of situations, e.g. when emotional support is needed, when there is an increased risk of overspending or when they are short of money.**

(Re-rate item)

Essential	Important	Don't know/Depends	Unimportant	Should not be included
○	○	○	○	○

***74. Where possible, and if memory problems are present, the financial counsellor should encourage the person to record important information in a diary.**

(New item)

Essential	Important	Don't know/Depends	Unimportant	Should not be included
○	○	○	○	○

***75. The financial counsellor should know about the local self-help and support groups for people with mental health problems and recommend them when appropriate.**

(New item)

Essential	Important	Don't know/Depends	Unimportant	Should not be included
○	○	○	○	○

***76. If it is known that a person behaves aggressively, the financial counsellor should ask a support worker or family member to attend the meeting with them.**

(New item)

Essential	Important	Don't know/Depends	Unimportant	Should not be included
○	○	○	○	○

Section 8 (cont): Actions to help the person

This section asks you about what information **the financial institution staff** should know about specific actions that can be taken to help the person with mental health problems and financial difficulties.

Please rate how important (from 'essential' to 'should not be included') you think it is that each statement be included in the guidelines.

***77. The financial institution staff should be proactive in assisting people with mental health problems before they experience financial difficulties, e.g. providing tips and tools for financial management, and financial counselling contact information.**

(Re-rate item)

Essential	Important	Don't know/Depends	Unimportant	Should not be included
○	○	○	○	○

***78. The financial institution staff and the person with mental health problems should come to an agreement about what is sufficient time to consult a financial counsellor or mental health professional.**

(Re-rate item)

Essential	Important	Don't know/Depends	Unimportant	Should not be included
○	○	○	○	○

***79. When the financial institution staff are referring a person who is in arrears to a financial counsellor, they should find out about current waiting list times in order to give the client sufficient time to consult with the financial counsellor.**

(New item)

Essential	Important	Don't know/Depends	Unimportant	Should not be included
○	○	○	○	○

***80. If the person with disclosed mental health problems is not adhering to arrangements tailored to their situation and there is a risk that they may lose their family home, the following financial institution staff should make a referral to an agency who can help them find alternative accommodation before selling the family home.**

(New item)

	Essential	Important	Don't know/Depends	Unimportant	Should not be included
Hardship staff	◯	◯	◯	◯	◯
Collection staff	◯	◯	◯	◯	◯
Branch staff	◯	◯	◯	◯	◯
Contact/call centre staff	◯	◯	◯	◯	◯

***81. If the financial institution staff in a customer facing role is concerned that the person may be experiencing mental health problems, they should talk with the person in a private room rather than at the desk with other customers present.**

(New item)

Essential	Important	Don't know/Depends	Unimportant	Should not be included
◯	◯	◯	◯	◯

***82. If the person appears to be in a negative state (i.e. confused or out of touch with reality, aggressive, highly distressed or anxious, or depressed) the following financial institution staff should avoid trying to come up with answers to the person's problems.**

(Altered item)

	Essential	Important	Don't know/Depends	Unimportant	Should not be included
Hardship staff	◯	◯	◯	◯	◯
Collection staff	◯	◯	◯	◯	◯
Branch staff	◯	◯	◯	◯	◯
Contact/call centre staff	◯	◯	◯	◯	◯

***83. If the person is being managed by a hardship team, that team should have the discretion to move from an automated to a tailored collection process.**

(Altered item)

Essential	Important	Don't know/Depends	Unimportant	Should not be included
◯	◯	◯	◯	◯

Section 8 (cont): Actions to help the person

This section asks you about what information **the mental health professional** should know about specific actions that can be taken to help the person with mental health problems and financial difficulties.

Please rate how important (from 'essential' to 'should not be included') you think it is that each statement be included in the guidelines.

*84. The mental health professional should be trained in the following basic skills:

	Essential	Important	Don't know/Depends	Unimportant	Should not be included
Assessing the pattern or history of the person's financial concerns. **(Re-rate item)**	○	○	○	○	○
Assisting the person to identify behaviours that have led to the problem debt, e.g. impulsive spending, unstable work history. **(Altered item)**	○	○	○	○	○

*85. The mental health professional should be aware that every state and territory has an ombudsman to deal with difficulties with energy providers.

(Re-rate item)

Essential	Important	Don't know/Depends	Unimportant	Should not be included
○	○	○	○	○

*86. The mental health professional should be aware that utility providers (gas, electricity, water) have, by law, provisions for people in financial hardship, and should encourage the person to make and maintain contact with the utility provider and request 'hardship' assistance, once financial difficulties are evident.

(Re-rate item)

Essential	Important	Don't know/Depends	Unimportant	Should not be included
○	○	○	○	○

***87. The mental health professional should not make payment arrangements with creditors regarding the person's debt without consulting with a financial counsellor, as there are specific consumer protection laws that might mean the debt does not need to be paid.**

(New item)

Essential	Important	Don't know/Depends	Unimportant	Should not be included
○	○	○	○	○

Section 9: Communication

This section asks you about what information **the financial counsellor and the financial institution staff** should know about how to effectively communicate with the person with mental health problems and financial difficulties.

Please rate how important (from 'essential' to 'should not be included') you think it is that each statement be included in the guidelines.

*88. When meeting the person face to face and the person is unable to communicate effectively (e.g. with mania), the financial counsellor should:

	Essential	Important	Don't know/Depends	Unimportant	Should not be included
Terminate the interview and reschedule the appointment.<br><br>**(New item)**	◯	◯	◯	◯	◯
Terminate the interview and reschedule the appointment to a time when a support person or mental health worker can attend with the person.<br><br>**(New item)**	◯	◯	◯	◯	◯
Ask the person if they would like to terminate the appointment and reschedule.<br><br>**(New item)**	◯	◯	◯	◯	◯
Discourage the person from making any financial decisions until they are able to communicate effectively.<br><br>**(New item)**	◯	◯	◯	◯	◯

***89. When meeting the person face to face and the person is unable to communicate effectively (e.g. with mania), the financial institution staff should:**

	Essential	Important	Don't know/Depends	Unimportant	Should not be included
Terminate the interview and reschedule the appointment. **(New item)**	○	○	○	○	○
Terminate the interview and reschedule the appointment to a time when a support person or mental health worker can attend with the person. **(New item)**	○	○	○	○	○
Ask the person if they would like to terminate the interview and reschedule. **(New item)**	○	○	○	○	○
Discourage the person from making any financial decisions until they are able to communicate effectively. **(New item)**	○	○	○	○	○

***90. When meeting the person face to face and the person is <u>presenting aggressively</u>, the financial counsellor should:**

	Essential	Important	Don't know/Depends	Unimportant	Should not be included
Be aware that the person's symptoms or fear causing their aggression might be exacerbated if you take certain steps (e.g. involve the police). **(Re-rate item)**	○	○	○	○	○
Terminate the interview and seek assistance from a supervisor or manager, if there is concern about their own or the person's safety. **(New item)**	○	○	○	○	○
Terminate the interview and reschedule the appointment. **(New item)**	○	○	○	○	○
Take a deep breath to calm and ground themselves. **(New item)**	○	○	○	○	○
State that the aggression is unacceptable behaviour. **(New item)**	○	○	○	○	○
Validate the person's feeling of frustration and anger. **(New item)**	○	○	○	○	○
Encourage them to get professional help, if appropriate. **(New item)**	○	○	○	○	○
Exercise good listening skills. **(New item)**	○	○	○	○	○
Try to ensure the safety of those around you. **(New item)**	○	○	○	○	○
Do not take the person's aggressive behaviour personally and understand that they may be feeling confused or frightened. **(New item)**	○	○	○	○	○

	Essential	Important	Don't know/Depends	Unimportant	Should not be included
Take a break after the encounter and talk to a supervisor or manager. **(New item)**	○	○	○	○	○

***91. When meeting the person face to face and the person is presenting aggressively, the financial institution staff should:**

	Essential	Important	Don't know/Depends	Unimportant	Should not be included
Be aware that the person's symptoms or fear causing their aggression might be exacerbated if you take certain steps (e.g. involve the police). **(Re-rate item)**	○	○	○	○	○
Consider inviting the person to sit down if they are standing. **(Rerate item)**	○	○	○	○	○
Terminate the interview and seek assistance from a supervisor or manager, if there is concern about their own or the person's safety. **(New item)**	○	○	○	○	○
Terminate the interview and reschedule the appointment. **(New item)**	○	○	○	○	○
Take a deep breath to calm and ground themselves. **(New item)**	○	○	○	○	○
State that the aggression is unacceptable behaviour. **(New item)**	○	○	○	○	○
Validate the person's feeling of frustration and anger. **New item)**	○	○	○	○	○
Encourage them to get professional help, if appropriate. **(New item)**	○	○	○	○	○
Exercise good listening skills. **(New item)**	○	○	○	○	○
Try to ensure the safety of those around you.	○	○	○	○	○

(New item)

Do not take the person's aggressive behaviour personally and understand that they may be feeling confused or frightened. **(New item)**	◌	◌	◌	◌	◌
Take a break after the encounter and talk to a supervisor or manager. **(New item)**	◌	◌	◌	◌	◌
Offer to see the person privately. **(New item)**	◌	◌	◌	◌	◌

*92. When there is serious concern about the person's safety, or in situations where the relevant actions do not seem to be working, the financial counsellor should:

	Essential	Important	Don't know/Depends	Unimportant	Should not be included
Request the police to check on them. **(New item)**	◌	◌	◌	◌	◌
Contact the authorised third party, if there is one, and discuss their concern. **(New item)**	◌	◌	◌	◌	◌
Terminate the interview and seek assistance from a supervisor or manager. **(New item)**	◌	◌	◌	◌	◌

***93. When there is serious concern about the person's safety, or in situations where the relevant actions do not seem to be working, the financial institution staff should:**

	Essential	Important	Don't know/Depends	Unimportant	Should not be included
Request the police to check on them. **(New item)**	○	○	○	○	○
Contact the authorised third party, if there is one, and discuss their concern. **(New item)**	○	○	○	○	○
Terminate the interview and seek assistance from a supervisor or manager. **(New item)**	○	○	○	○	○

***94. The financial institution staff should provide electronic communication (e.g. email, web form) for people with mental health problems to contact them about financial difficulties.**

(Re-rate item)

Essential	Important	Don't know/Depends	Unimportant	Should not be included
○	○	○	○	○

Section 10: Working with the person over the phone

This section contains statements that apply to when the **financial counsellor** or the **financial institution staff** are **talking over the phone** to a person who appears to be in one of the following negative emotional states:

• Confused or out of touch with reality

• Aggressive

• Depressed

Please rate how important (from 'essential' to 'should not be included') you think it is that each statement be included in the guidelines.

Confused or out of touch with reality

This section is about working with the person *over the phone.*

*95. The following financial institution staff should be aware that the person who is confused or out of touch with reality may be frightened by their thoughts and feelings.

(Altered item)

	Essential	Important	Don't know/Depends	Unimportant	Should not be included
Hardship staff	○	○	○	○	○
Collection staff	○	○	○	○	○
Branch staff	○	○	○	○	○
Contact/call centre staff	○	○	○	○	○

*96. The following financial institution staff should be aware that the person's confusion and fear about what is happening to them may lead them to deny that anything is wrong with them.

(Altered item)

	Essential	Important	Don't know/Depends	Unimportant	Should not be included
Hardship staff	○	○	○	○	○
Collection staff	○	○	○	○	○
Branch staff	○	○	○	○	○
Contact/call centre staff	○	○	○	○	○

***97. When talking over the phone to the person who is <u>confused or out of touch with reality</u>, the financial counsellor should:**

	Essential	Important	Don't know/Depends	Unimportant	Should not be included
Paraphrase what the person has said. **(New item)**	○	○	○	○	○
Terminate the conversation and attempt to organise another time to talk. **(New item)**	○	○	○	○	○
Should not raise their voice as if the person is deaf. **(New item)**	○	○	○	○	○

***98. When talking over the phone to the person who is <u>confused or out of touch with reality</u>, the financial institution staff should:**

	Essential	Important	Don't know/Depends	Unimportant	Should not be included
Paraphrase what the person has said. **(New item)**	○	○	○	○	○
Terminate the conversation and attempt to organise another time to talk. **(New item)**	○	○	○	○	○
Should not raise their voice as if the person is deaf. **(New item)**	○	○	○	○	○

Section 10 (cont): Working with the person over the phone

This section contains statements that apply to when the **financial counsellor** or the **financial institution staff** are **talking over the phone** to a person who appears to be in one of the following negative emotional states:

• Confused or out of touch with reality

• Aggressive

• Depressed

Please rate how important (from 'essential' to 'should not be included') you think it is that each statement be included in the guidelines.

Aggression

This section is about working with the person *over the phone.*

***99. If the person is becoming aggressive while talking over the phone, the financial counsellor should:**

	Essential	Important	Don't know/Depends	Unimportant	Should not be included
Offer for the client to talk with another staff member. **(New item)**	○	○	○	○	○
Involve a more senior staff member who is trained in mental health issues. **(New item)**	○	○	○	○	○
Contact the support person, with the person's consent. **(New item)**	○	○	○	○	○
Contact the person's mental health professional, with the person's consent. **(New item)**	○	○	○	○	○
Explain to the person that although you would like to help them, it is not possible when they are (state the unacceptable behaviour, e.g. yelling). **(New item)**	○	○	○	○	○
If the person is threatening harm to themselves or others, explain that this cannot be kept confidential and try to ascertain the person's whereabouts. **(New item)**	○	○	○	○	○
If the person is threatening harm to themselves or others, take any necessary protective action, e.g. contact the police or mental health crisis team. **(New item)**	○	○	○	○	○

***100. If the person is becoming aggressive over the phone, the financial institution staff should:**

	Essential	Important	Don't know/Depends	Unimportant	Should not be included
Offer for the client to talk with another staff member.<br><br>**(New item)**	◯	◯	◯	◯	◯
Involve a more senior staff member who is trained in mental health issues.<br><br>**(New item)**	◯	◯	◯	◯	◯
Contact the support person, with the person's consent.<br><br>**(New item)**	◯	◯	◯	◯	◯
Contact the person's mental health professional, with the person's consent.<br><br>**(New item)**	◯	◯	◯	◯	◯
Explain to the person that although you would like to help them, it is not possible when they are (state the unacceptable behaviour, e.g. yelling).<br><br>**(New item)**	◯	◯	◯	◯	◯
If the person is threatening harm to themselves or others, explain that this cannot be kept confidential and try to ascertain the person's whereabouts.<br><br>**(New item)**	◯	◯	◯	◯	◯
If the person is threatening harm to themselves or others, take any necessary protective action, e.g. contact the police or mental health crisis team.<br><br>**(New item)**	◯	◯	◯	◯	◯

Section 10 (cont): Working with the person over the phone

This section contains statements that apply to when the **financial counsellor** or the **financial institution staff** are **talking over the phone** to a person who appears to be in one of the following negative emotional states:

• Confused or out of touch with reality

• Aggressive

• Depressed

Please rate how important (from 'essential' to 'should not be included') you think it is that each statement be included in the guidelines.

Depressed

This section is about working with the person *over the phone.*

***101. When talking over the phone with the person the financial institution staff should be familiar with and listen for signs that may indicate that the person is feeling depressed, i.e. sadness, hopelessness, worthlessness, difficulty concentrating, difficulty making decisions, agitation, slow monotonous speech, etc.**

(Re-rate item)

Essential	Important	Don't know/Depends	Unimportant	Should not be included
○	○	○	○	○

***102. If the financial counsellor has listened non-judgmentally while talking with the person over the phone and the person remains in their negative emotional state they should offer the following:**

	Essential	Important	Don't know/Depends	Unimportant	Should not be included
Offer to contact the support person. **(New item)**	◯	◯	◯	◯	◯
Offer to contact the person's mental health worker. **(New item)**	◯	◯	◯	◯	◯
Before breaking off contact, get assurance from the person that they will not harm themselves. **(New item)**	◯	◯	◯	◯	◯
Offer to text the person the phone numbers for the crisis line. **(New item)**	◯	◯	◯	◯	◯

***103. When talking over the phone with the person and they are talking about suicide, the following people should offer to call a suicide call-back service on behalf of the person.**

(New item)

	Essential	Important	Don't know/Depends	Unimportant	Should not be included
Financial counsellor	◯	◯	◯	◯	◯
Financial institution staff	◯	◯	◯	◯	◯

***104. If the financial institution staff have listened non-judgmentally while talking to the person over the phone, and the person remains in their negative emotional state they should offer the following:**

	Essential	Important	Don't know/Depends	Unimportant	Should not be included
Offer to contact the support person. **(New item)**	◯	◯	◯	◯	◯
Offer to contact the person's mental health worker. **(New item)**	◯	◯	◯	◯	◯
Before breaking off contact, get assurance from the person that they will not harm themselves. **(New item)**	◯	◯	◯	◯	◯
Offer to text the person the phone numbers for the crisis line. **(New item)**	◯	◯	◯	◯	◯

Section 11: Policy and Procedures

This section contains statements about policies and procedures relevant to working with people with mental health problems and financial difficulties which may be adopted by **financial counselling services and financial institutions**.

Please rate how important (from 'essential' to 'should not be included') you think it is that each statement be included in the guidelines.

***105. Financial counsellors should receive basic counselling skills training.**

(New item)

Essential	Important	Don't know/Depends	Unimportant	Should not be included
○	○	○	○	○

***106. Any mental health training of financial counsellors should include:**

	Essential	Important	Don't know/Depends	Unimportant	Should not be included
Hearing about the lived experience of people who have had mental health problems and financial difficulties. **(New item)**	○	○	○	○	○
Learning about the impact of mental health problems and financial difficulties on the family of the person. **(New item)**	○	○	○	○	○

***107. Any mental health training of financial counsellors should be contemporary values-based, recovery-oriented, person-centred, trauma-informed thinking, and not a 'chemical imbalance'/defective brain disease model.**

(New item)

Essential	Important	Don't know/Depends	Unimportant	Should not be included
○	○	○	○	○

Helping the person with mental health problems and financial difficulties -

***108. Any mental health training of financial institution staff should include:**

	Essential	Important	Don't know/Depends	Unimportant	Should not be included
Hearing about the lived experience of people who have had mental health problems and financial difficulties. **(New item)**	○	○	○	○	○
Learning about the impact of mental health problems and financial difficulties on the family of the person. **(New item)**	○	○	○	○	○

***109. Any mental health training of financial institution staff should be contemporary-values based, recovery-oriented, person-centred, trauma-informed thinking, and not a 'chemical imbalance'/defective brain disease model.**

(New item)

Essential	Important	Don't know/Depends	Unimportant	Should not be included
○	○	○	○	○

***110. For people with mental health problems, financial institutions should have a tailored procedure for reviewing and taking action at key points in the collection process, e.g. sale of assets or sale of debt.**

(Re-rate item)

Essential	Important	Don't know/Depends	Unimportant	Should not be included
○	○	○	○	○

***111. The tailored procedure should include:**

	Essential	Important	Don't know/Depends	Unimportant	Should not be included
Deferring action when the person is acutely unwell or in a mental health crisis. **(Altered item)**	○	○	○	○	○
Engaging a specialist service to visit the property and the person to assess the situation before proceeding with sale of property. **(New item)**	○	○	○	○	○

***112. If sale of debt is deemed necessary, the financial institution should contact either the person or their third party representative to determine what, if any, information should be passed on to to the next financial institution.**

(New item)

Essential	Important	Don't know/Depends	Unimportant	Should not be included
○	○	○	○	○

***113. Financial institutions should provide a single point of contact for the person with severe mental illness for the duration of their financial difficulties. (A severe mental illness is defined as a mental illness that has a significant disabling affect on the person's ability to work, look after themselves, and carry on relationships with family and friends. It is considered to be a long-term illness.)**

(Re-rate item)

Essential	Important	Don't know/Depends	Unimportant	Should not be included
○	○	○	○	○

***114. Financial institutions should provide a dedicated phone number for people with mental health problems and financial difficulties and their authorised representatives.**

(Re-rate item)

Essential	Important	Don't know/Depends	Unimportant	Should not be included
○	○	○	○	○

***115. Financial institutions should provide a specialised dedicated team for working with the person with mental health problems and financial difficulties.**

(Re-rate item)

Essential	Important	Don't know/Depends	Unimportant	Should not be included
○	○	○	○	○

116. The hardship team should have access to a mental health professional who they can consult with to give better customer care.

(Re-rate item)

Essential	Important	Don't know/Depends	Unimportant	Should not be included
○	○	○	○	○

***117. Financial institution staff should know a <u>few key</u> mental health and disability support services available in their community.**

(New item)

	Essential	Important	Don't know/Depends	Unimportant	Should not be included
Hardship staff	○	○	○	○	○
Collection staff	○	○	○	○	○
Branch staff	○	○	○	○	○
Contact/call centre staff	○	○	○	○	○

***118. Financial institutions should update their information on external resources/community services regularly to ensure they are not out of date.**

(New item)

Essential	Important	Don't know/Depends	Unimportant	Should not be included
○	○	○	○	○

***119. Financial counselling services should update their information on external resources/community services regularly to ensure they are not out of date.**

(New item)

Essential	Important	Don't know/Depends	Unimportant	Should not be included
○	○	○	○	○

***120. Any helpline for people with financial difficulties should have a menu option to assist people in a mental health crisis.**

(New item)

Essential	Important	Don't know/Depends	Unimportant	Should not be included
○	○	○	○	○

***121. Any website for people with financial difficulties should have a link to information which assists people in a mental health crisis.**

(New item)

Essential	Important	Don't know/Depends	Unimportant	Should not be included
○	○	○	○	○

***122. The financial institution's hardship webpage should have a link to information which assists people in a mental health crisis.**

(New item)

Essential	Important	Don't know/Depends	Unimportant	Should not be included
○	○	○	○	○

***123. Financial institutions should monitor the implementation of mental health problem policies to ensure they are meeting their aims, e.g. number of people who disclose, types of conditions disclosed, broken arrangements involving people who disclose, use of specialist team.**

(Re-rate item)

Essential	Important	Don't know/Depends	Unimportant	Should not be included
○	○	○	○	○

That is the end of the second round survey!

Thank you very much for your contribution.

By pressing the "next" button your final responses will be registered with our survey software. Once all panel members have lodged their responses, we will collate the data and send you a report on the findings and the third and final survey.

We are extremely grateful for your contribution.

The Mental Health First Aid Research Team
