# Supplementary material for: Assisting Australians with mental health problems and financial difficulties: a Delphi study to develop guidelines for financial counsellors, financial institution staff, mental health professionals and carers
Source: BMC Health Serv Res. 2015 Jun 3;15:218. doi: 10.1186/s12913-015-0868-2 (PMC4453216; doi:10.1186/s12913-015-0868-2)
Supplement: Additional file 3: — Copy of Round 3 survey. [file 12913_2015_868_MOESM3_ESM.pdf]

Helping the person with mental health problems and financial difficulties -

Information about this research

1. Purpose of the research

Mental Health First Aid (MHFA) is the help provided to a person developing a mental health problem or in a mental health crisis. The first aid is given until appropriate professional treatment is received or until the crisis resolves. The purpose of this project is to develop guidelines for offering appropriate mental health first aid to the person who is experiencing mental health problems and financial difficulties.

This research project is funded by the Australian Government Department of Health and Ageing, and ANZ, GE Money, NAB, Westpac and *beyondblue*.

2. Your role in Round Three

Your task is to rate the statements presented in this questionnaire according to how important you believe they are for providing guidance to financial counsellors, financial institution staff, mental health professionals and the support person who is assisting the person experiencing mental health problems and financial difficulties.

3. How this Round Three questionnaire was developed

Statements in this questionnaire are the new or altered items from Round 2 that did not receive a level of consensus that was either high enough to be clearly endorsed or low enough to be clearly rejected.

It is important to note that we do not necessarily agree with these statements; we have included them because we do not believe that we should decide what the best practice is in this area. Rather, we have invited you, a member of the expert panel, to help develop a set of guidelines that reflect current expert opinion.

4. Instructions

Please complete the questionnaire by rating each statement according to how important you believe it is for inclusion in guidelines for offering mental health first aid to the person experiencing mental health problems and financial difficulties. This questionnaire should take approximately 20 minutes to complete. If necessary, you can complete the survey in two or more sittings. Your answers are saved when you click 'Next' at the bottom of a page. This marks your page and you can begin again at a later date on the next page. <u>Please be aware that once you have logged on and started responding you must complete the questionnaire on this same computer.</u>

5. Consent to participate

It is important for you to know that participation in this study is completely voluntary. You are not under any obligation to participate and you can withdraw at any time. Submitting your questionnaire is an indication of your understanding of this and your consent to participate in the study.

We would like to thank you for your time and effort and encourage you to provide us with feedback on this process.

Best Wishes,

The Mental Health First Aid Research Team

Definitions

Definitions

These terms may have different meaning for the groups represented in this survey (i.e. financial counsellors, financial institution staff, consumers, carers and mental health professionals). When answering these questions, please ensure you use our definitions only.

‘The person’, unless otherwise stated, refers to the person with *mental health problems and financial difficulties*. This person may be called a customer, client, patient, consumer, relative or friend.

'Mental illness' is a diagnosable illness that affects a person's thinking, emotional state and behaviour, and disrupts the person's ability to work or carry out other daily activities and engage in satisfying personal relationships.

‘Mental health problems’ is a broader term including mental illnesses, symptoms of mental illnesses that may not be severe enough to warrant the diagnosis of a mental illness, as well as mental health-related crises such as having thoughts of suicide. For the purposes of this survey, problem gambling has been excluded from mental health problems, as this may be the subject of a future specialised project.

‘Financial difficulties’ are when income is insufficient to cover reasonable living expenses and meet financial commitments as they become due. This definition covers people in financial hardship, as well as those experiencing other financial challenges. This does not include when a payment has simply been overlooked.

‘The support person’ refers to someone who is a significant source of emotional and/or practical support or assistance to 'the person'. It may include a partner, close friend or family member. However, it is not assumed that every person has a support person.

'Authorised third party representative' refers to anyone who has been appointed, in writing or orally, by 'the person' to act on their behalf regarding financial decisions.

‘The financial counsellor’ is a trained professional who assists people who are experiencing financial difficulties. Most financial counsellors work in community organisations, although some work in government agencies and local government. Financial counsellors provide a free, confidential and independent service. The financial counsellor is not a financial planner, financial adviser or legal practitioner and is not a general welfare worker.

'Financial institution' is any institution that is in the position to collect money from individuals, including banks or those who act on their behalf.

'Financial institution staff' are employees of financial institutions that work with the person with mental health problems and financial difficulties.

'Hardship team' is a financial institution's team that is trained to provide financial solutions for people experiencing financial difficulties/hardship.

'Hardship' is defined as when 'the person' is being managed by a financial institution's hardship team.

'Collections staff' are financial institution staff whose primary duty is to negotiate payment from people with overdue accounts.

'Branch staff' are financial institution staff who work in the local bank branches.

'Contact/call centre staff' are financial institution staff who work with customers over the phone. They can be located overseas or in Australian.

‘The mental health professional’ refers to a trained health professional who is treating/working with 'the person'. This could be a psychologist, mental health nurse or psychiatrist. It could also be a social worker, occupational therapist or GP who has specialist mental health training.

Information about you

*1. Please enter your participant code or your name.

Section 1: General awareness about mental health problems and financial di...

This section asks you about what information **the financial institution staff** should know about mental health problems and financial difficulties.

Please rate how important (from 'essential' to 'should not be included') you think it is that each statement be included in the guidelines for each of the groups listed in the left hand column.

***2. The financial institution *branch* staff should be aware of the signs and symptoms of mental illnesses. (*Please note that this does not mean knowing how to diagnose a mental illness.*)**

Round 2 results (percentage of the panel that endorsed the item)

Consumer - 95.0

Carer - 95.8

MHP - 87.1

FC - 97.1

FIS - 78.3

Essential	Important	Don't know/Depends	Unimportant	Should not be included
○	○	○	○	○

***3. The financial institution contact/call centre staff should be aware of the risk factors for suicide.**

Round 2 results (percentage of the panel that endorsed the item)

Consumer - 90.0

Carer - 92.0

MHP - 80.6

FC - 91.2

FIS - 76.7

Essential	Important	Don't know/Depends	Unimportant	Should not be included
○	○	○	○	○

***4. The financial institution *hardship* staff should be aware of how they can reduce stressors that can contribute to relapse, i.e. a reoccurrence of a mental health problem in a person who is currently well.**

Round 2 results (percentage of the panel that endorsed the item)
Consumer - 95.0
Carer - 100.0
MHP - 96.8
FC - 100.0
FIS - 76.7

Essential	Important	Don't know/Depends	Unimportant	Should not be included
○	○	○	○	○

***5. The financial institution collection staff should be aware of the potential effects of any stigmatising attitudes the financial institution staff may have on the person with mental health problems.**

Round 2 results (percentage of the panel that endorsed the item)
Consumer - 100.0
Carer - 100.0
MHP - 100.0
FC - 100.0
FIS - 78.3

Essential	Important	Don't know/Depends	Unimportant	Should not be included
○	○	○	○	○

***6. The financial institution *hardship* staff should be aware of the myths surrounding mental health problems that lead to stigma and limit the potential earning capabilities of the person affected by mental health problems.**

Round 2 results (percentage of the panel that endorsed the item)
Consumer - 100.0
Carer - 100.0
MHP - 100.0
FC - 94.1
FIS - 78.3

Essential	Important	Don't know/Depends	Unimportant	Should not be included
○	○	○	○	○

***7. The financial institution *collection* staff should be aware that dealing with financial matters (e.g. opening mail from creditors, visiting Centrelink) may be overwhelming for the person.**

Round 2 results (percentage of the panel that endorsed the item)

Consumer - 95.0

Carer - 100.0

MHP - 96.8

FC - 100.0

FIS - 78.0

Essential	Important	Don't know/Depends	Unimportant	Should not be included
○	○	○	○	○

***8. The financial institution *hardship* staff should know that the way the person perceives their financial situation is what impacts on their mental health, rather than their actual situation.**

Round 2 results (percentage of the panel that endorsed the item)

Consumer - 95.0

Carer - 100.0

MHP - 93.5

FC - 100.0

FIS - 78.3

Essential	Important	Don't know/Depends	Unimportant	Should not be included
○	○	○	○	○

***9. The financial institution *hardship* staff should know that the person with mental health problems may incur debt to support a drug or alcohol problem.**

Round 2 results (percentage of the panel that endorsed the item)

Consumer - 80.0

Carer - 88.0

MHP - 87.1

FC - 94.1

FIS - 75.0

Essential	Important	Don't know/Depends	Unimportant	Should not be included
○	○	○	○	○

***10. The financial institution *branch* staff should have a *general understanding* of their state or territory laws regarding the appointment of a third party to manage the person's finances, e.g. guardianship, administration orders, financial management orders.**

Round 2 results (percentage of the panel that endorsed the item)

Consumer - 90.0

Carer - 92.0

MHP - 87.1

FC - 91.2

FIS - 76.7

Essential	Important	Don't know/Depends	Unimportant	Should not be included
○	○	○	○	○

Section 2: Specific mental health problems

This section asks you about what information **the financial institution staff** should know about specific mental health problems and financial difficulties.

Please rate how important (from ‘essential’ to ‘should not be included’) you think it is that each statement be included in the guidelines.

***11. The financial institution *collection* staff should be aware of how an episode of mania may affect a person's financial circumstances, e.g. not paying bills, job instability, over-spending.**

Round 2 results (percentage of the panel that endorsed the item)

Consumer - 95.0

Carer - 100.0

MHP - 96.8

FC - 97.1

FIS - 77.6

Essential	Important	Don't know/Depends	Unimportant	Should not be included
○	○	○	○	○

Section 3: Screening and referral

This section asks you about what information **the financial counsellor** and **the financial institution staff** need to know in order to refer the person to mental health services.

Please rate how important (from 'essential' to 'should not be included') you think it is that each statement be included in the guidelines.

***12. If the person discloses mental health problems and *asks for help*, the financial counsellor should provide written material about appropriate professional help.**

Round 2 results (percentage of the panel that endorsed the item)
Consumer - 75.0
Carer - 92.0
MHP - 96.7
FC - 82.4
FIS - 88.3

Essential	Important	Don't know/Depends	Unimportant	Should not be included
○	○	○	○	○

***13. If it is suspected that the person in *financial difficulties* may have mental health problems, the the financial institution *collection* staff should *offer* to refer them to financial counselling services, who can advocate on the person's behalf and make an appropriate referral.**

Round 2 results (percentage of the panel that endorsed the item)
Consumer - 90.0
Carer - 88.0
MHP - 100.0
FC - 94.1
FIS - 76.7

Essential	Important	Don't know/Depends	Unimportant	Should not be included
○	○	○	○	○

Section 4: Working together

This section asks you about what information **the financial counsellor** and **the mental health professional** should know about how to work together to get the best outcome for the person with mental health problems and financial difficulties.

Please rate how important (from 'essential' to 'should not be included') you think it is that each statement be included in the guidelines.

***14. The financial counsellor and the mental health professional should inform the person about the content of any discussion between them.**

Round 2 results (percentage of the panel that endorsed the item)

Consumer - 95.0

Carer - 92.0

MHP - 90.3

FC - 76.5

FIS - 76.7

Essential	Important	Don't know/Depends	Unimportant	Should not be included
○	○	○	○	○

15. The financial counsellor should work with the mental health professional to determine whether the person is able to make specific decisions concerning their current financial situation, *with the person's consent.

Round 2 results (percentage of the panel that endorsed the item)

Consumer - 90.0

Carer - 88.0

MHP - 77.4

FC - 79.4

FIS - 91.7

Essential	Important	Don't know/Depends	Unimportant	Should not be included
○	○	○	○	○

Helping the person with mental health problems and financial difficulties -

***16. The financial counsellor and the mental health professional should work together and deliver consistent messages to the person, *with the person's consent*.**

Round 2 results (percentage of the panel that endorsed the item)

Consumer - 100.0

Carer - 96.0

MHP - 90.3

FC - 76.5

FIS - 88.3

Essential	Important	Don't know/Depends	Unimportant	Should not be included
○	○	○	○	○

***17. The financial counsellor and the mental health professional should be aware of and discuss any conflicting priorities between financial and treatment goals, *with the person's consent*.**

Round 2 results (percentage of the panel that endorsed the item)

Consumer - 100.0

Carer - 96.0

MHP - 83.9

FC - 79.4

FIS - 88.3

Essential	Important	Don't know/Depends	Unimportant	Should not be included
○	○	○	○	○

Section 6: Disclosure

This section asks you about what information **the mental health professional** should know about disclosing mental health problems to a financial institution.

Please rate how important (from 'essential' to 'should not be included') you think it is that each statement be included in the guidelines.

***18. If the person decides to disclose their mental health problems to the financial institution, the mental health professional should discuss with the person what to disclose.**

Round 2 results (percentage of the panel that endorsed the item)

Consumer - 85.0

Carer - 84.0

MHP - 83.9

FC - 76.5

FIS - 80.0

Essential	Important	Don't know/Depends	Unimportant	Should not be included
○	○	○	○	○

Section 7: Documentation

This section asks you about what information **financial institution staff** should know about documentation that might benefit the person with mental health problems and financial difficulties and assist in communication between the financial counsellor, the mental health professional and the financial institution.

Please rate how important (from ‘essential’ to ‘should not be included’) you think it is that each statement be included in the guidelines.

***19. The financial institution *hardship* staff should know what an advance directive is and how to act upon it.**

Round 2 results (percentage of the panel that endorsed the item)

Consumer - 100.0

Carer - 100.0

MHP - 100.0

FC - 88.2

FIS - 78.3

Essential	Important	Don't know/Depends	Unimportant	Should not be included
○	○	○	○	○

Section 8: Actions to help the person

This section asks you about what information **the financial institution staff** and **the mental health professional** should know about specific actions that can be taken to help the person with mental health problems and financial difficulties.

Please rate how important (from 'essential' to 'should not be included') you think it is that each statement be included in the guidelines.

***20. If the person is being managed by a hardship team, that team should have the discretion to move from an automated to a tailored collection process.**

Round 2 results (percentage of the panel that endorsed the item)

Consumer - 100.0

Carer - 92.0

MHP - 77.4

FC - 100.0

FIS - 83.3

Essential	Important	Don't know/Depends	Unimportant	Should not be included
○	○	○	○	○

***21. The mental health professional should not make payment arrangements with creditors regarding the person's debt without consulting with a financial counsellor, as there are specific consumer protection laws that might mean the debt does not need to be paid.**

Round 2 results (percentage of the panel that endorsed the item)

Consumer - 95.0

Carer - 88.0

MHP - 87.1

FC - 91.2

FIS - 78.3

Essential	Important	Don't know/Depends	Unimportant	Should not be included
○	○	○	○	○

Section 9: Communication

This section asks you about what information **the financial institution staff** should know about how to effectively communicate with the person with mental health problems and financial difficulties.

Please rate how important (from 'essential' to 'should not be included') you think it is that each statement be included in the guidelines.

***22. When there is serious concern about the person's safety, or in situations where the relevant actions do not seem to be working, the financial institution staff should contact the authorised third party, if there is one, and discuss their concern.**

Round 2 results (percentage of the panel that endorsed the item)

Consumer - 90.0

Carer - 100.0

MHP - 100.0

FC - 88.2

FIS - 78.3

Essential	Important	Don't know/Depends	Unimportant	Should not be included
○	○	○	○	○

***23. The financial institution *collection* staff should be aware that the person who is <u>confused or out of touch with reality</u> may be frightened by their thoughts and feelings.**

Round 2 results (percentage of the panel that endorsed the item)

Consumer - 95.0

Carer - 92.0

MHP - 100.0

FC - 100.0

FIS - 76.7

Essential	Important	Don't know/Depends	Unimportant	Should not be included
○	○	○	○	○

***24. The financial institution *branch* staff should be aware that the person who is <u>confused or out of touch with reality</u> may be frightened by their thoughts and feelings.**

Round 2 results (percentage of the panel that endorsed the item)

Consumer - 100.0

Carer - 88.0

MHP - 96.8

FC - 93.9

FIS - 75.0

Essential	Important	Don't know/Depends	Unimportant	Should not be included
○	○	○	○	○

***25. The financial institution *collection* staff should be aware that the person's confusion and fear about what is happening to them may lead them to deny that anything is wrong with them.**

Round 2 results (percentage of the panel that endorsed the item)

Consumer - 95.0

Carer - 96.0

MHP - 96.8

FC - 100.0

FIS - 78.3

Essential	Important	Don't know/Depends	Unimportant	Should not be included
○	○	○	○	○

Section 10: Working with the person over the phone

This section contains statements that apply to when the **financial counsellor** or the **financial institution staff** are **talking over the phone** to a person who appears to be in one of the following negative emotional states:

• Confused or out of touch with reality

• Aggressive

• Depressed

Please rate how important (from 'essential' to 'should not be included') you think it is that each statement be included in the guidelines.

***26. When talking over the phone to the person who is confused or out of touch with reality, the financial counsellor should paraphrase what the person has said.**

Round 2 results (percentage of the panel that endorsed the item)
Consumer - 75.0
Carer - 76.0
MHP - 77.4
FC - 85.3
FIS - 83.3

Essential	Important	Don't know/Depends	Unimportant	Should not be included
○	○	○	○	○

***27. If the person is becoming aggressive while talking over the phone, the financial counsellor should explain to the person that although you would like to help them, it is not possible when they are (state the unacceptable behaviour, e.g. yelling).**

Round 2 results (percentage of the panel that endorsed the item)
Consumer - 80.0
Carer - 80.0
MHP - 80.6
FC - 88.2
FIS - 78.3

Essential	Important	Don't know/Depends	Unimportant	Should not be included
○	○	○	○	○

***28. If the person is becoming aggressive over the phone, the financial institution staff should involve a more senior staff member who is trained in mental health issues.**

Round 2 results (percentage of the panel that endorsed the item)

Consumer - 90.0

Carer - 91.7

MHP - 80.6

FC - 79.4

FIS - 78.3

Essential	Important	Don't know/Depends	Unimportant	Should not be included
○	○	○	○	○

***29. If the person is becoming aggressive over the phone, the financial institution staff should explain to the person that although you would like to help them, it is not possible when they are (state the unacceptable behaviour, e.g. yelling).**

Round 2 results (percentage of the panel that endorsed the item)

Consumer - 75.0

Carer - 84.0

MHP - 90.3

FC - 88.2

FIS - 83.1

Essential	Important	Don't know/Depends	Unimportant	Should not be included
○	○	○	○	○

***30. When talking over the phone with the person and they are talking about suicide, the financial counsellor should offer to call a suicide call-back service on behalf of the person.**

Round 2 results (percentage of the panel that endorsed the item)

Consumer - 75.0

Carer - 92.0

MHP - 80.6

FC - 88.2

FIS - 93.3

Essential	Important	Don't know/Depends	Unimportant	Should not be included
○	○	○	○	○

Section 11: Policy and Procedures

This section contains statements about policies and procedures relevant to working with people with mental health problems and financial difficulties which may be adopted by **financial counselling services and financial institutions**.

Please rate how important (from 'essential' to 'should not be included') you think it is that each statement be included in the guidelines.

31.** ***The following item was endorsed:

For people with mental health problems, financial institutions should have a tailored procedure for reviewing and taking action at key points in the collection process, e.g. sale of assets or sale of debt.

Please rate the following statement:

The tailored procedure should include deferring action when the person is acutely unwell or in a mental health crisis.

Round 2 results (percentage of the panel that endorsed the item)
Consumer - 100.0
Carer - 100.0
MHP - 93.5
FC - 100.0
FIS - 76.3

Essential	Important	Don't know/Depends	Unimportant	Should not be included
○	○	○	○	○

***32. The financial institution *collection* staff should know a <u>few key</u> mental health and disability support services available in their community.**

Round 2 results (percentage of the panel that endorsed the item)
Consumer - 95.0
Carer - 100.0
MHP - 87.1
FC - 93.9
FIS - 76.3

Essential	Important	Don't know/Depends	Unimportant	Should not be included
○	○	○	○	○

***33. The financial institution *branch* staff should know a <u>few key</u> mental health and disability support services available in their community.**

Round 2 results (percentage of the panel that endorsed the item)

Consumer - 95.0

Carer - 100.0

MHP - 76.7

FC - 93.9

FIS - 86.4

Essential	Important	Don't know/Depends	Unimportant	Should not be included
○	○	○	○	○

***34. Any website for people with financial difficulties should have a link to information which assists people in a mental health crisis, e.g. Lifeline, *beyondblue*.**

Round 2 results (percentage of the panel that endorsed the item)

Consumer - 95.0

Carer - 96.0

MHP - 77.4

FC - 100.0

FIS - 88.1

Essential	Important	Don't know/Depends	Unimportant	Should not be included
○	○	○	○	○

That is the end of the round 3 survey! This is also the last survey you will need to complete for this project.

<u>**Thank you for your time!**</u>

If you are interested in being involved in any further mental health first aid research please check the MHFA website (www.mhfa.com.au) for any future opportunities.

All the best from,
The MHFA Research Team
