# Supplementary material for: Assisting Australians with mental health problems and financial difficulties: a Delphi study to develop guidelines for financial counsellors, financial institution staff, mental health professionals and carers
Source: BMC Health Serv Res. 2015 Jun 3;15:218. doi: 10.1186/s12913-015-0868-2 (PMC4453216; doi:10.1186/s12913-015-0868-2)
Supplement: Additional file 4: — Endorsed Items: List of the survey items that were endorsed by all five panels. [file 12913_2015_868_MOESM4_ESM.pdf]

Additional file 1: Endorsed items

Financial Counsellors: General awareness about mental health problems

Financial counsellors should be aware of:

- What mental illness is.*
- How common mental illnesses are in the community.*
- The types of mental illnesses.*
- The signs and symptoms of mental illnesses.*
- The causes of and risk factors for mental illnesses.*
- The things which might indicate that the person has mental health problems, e.g. not attending a[illegible] not meeting repayment schedules, displaying unusual behaviours, lack of clarity of thought.*
- How they can support the person with mental health problems in ways that promote recovery.*
- How they can reduce stressors that may exacerbate the person's mental health problems.*
- That mental health problems may take time, usually weeks or months, to develop.*
- The warning signs of suicide.*
- The risk factors for suicide.*

Financial counsellors should be familiar with the following mental health first aid guidelines:

- Depression*
- Suicidal thoughts and behaviours*
- Psychosis*
- Panic attack^

*Endorsed in Round 1; ^Endorsed in Round 2

All Financial Institution Staff: General awareness about mental health problems

Financial institution staff should be aware of what mental illness is.*

Financial institution staff should be aware of how common mental illnesses are in the community.*

*Endorsed in Round 1

Hardship Staff: General awareness about mental health problems

Hardship staff should be aware of the signs and symptoms of mental illnesses. Please note that this does not mean knowing how to diagnose a mental illness.^

Hardship staff should be aware of the things which might indicate that the person has mental health problems, e.g. not attending appointments, not returning phone calls, not meeting repayment schedules, displaying unusual behaviours, lack of clarity of thought. Please note that this does not mean knowing how to diagnose a mental illness.^

^Endorsed in Round 2

Hardship Staff: General awareness about mental health problems (cont.)

Hardship staff should be aware of the risk factors for suicide.^

Hardship staff should know how they can reduce stressors that may exacerbate the person's mental health problems.^

Hardship staff should be aware that mental health problems can develop or worsen rapidly.^

^Endorsed in Round 2

Collection Staff: General awareness about mental health problems

Collection staff should be aware of the risk factors for suicide.^

Collection staff should be aware that mental health problems can develop or worsen rapidly.^

^Endorsed in Round 2

Branch Staff: General awareness about mental health problems

Financial institution staff who are in customer facing roles should be familiar with the following mental health first aid guidelines:

- Depression*
- Suicidal thoughts and behaviours*

The following financial institution staff should be aware of the risk factors for suicide.^

*Endorsed in Round 1; ^Endorsed in Round 2

Contact Centre Staff: General awareness about mental health problems

Contact centre staff should be aware of the risk factors for suicide.*

*Endorsed in Round 1

The Support Person: General awareness about mental health problems

The support person should be aware of the warning signs of suicide.*

The support person should be aware of the risk factors for suicide.*

The support person should be aware of the First Aid Guidelines for Assisting the Person Who is Suicidal. *

*Endorsed in Round 1; ^Endorsed in Round 2; #Endorsed in Round 3

Financial Counsellors: Awareness about mental health problems and financial difficulties

Financial counsellors should be aware:

- Of the level of support needed by the person with mental health problems will fluctuate, as the symptoms of most mental health problems come and go over time.*
- Of the person with mental health problems may not overspend but may have other problems with managing money, e.g. withdrawing all their money from the bank to keep it 'safe' at home.*
- Of the impact of the symptoms of mental health problems on the tasks necessary to keep up with financial responsibilities, e.g. problems with memory, decision-making and motivation, or conversely, over-confidence and over-spending in mania.*
- That having mental health problems is not necessarily an indicator of an inability to manage money. The effects of mental health problems will differ from one person to another, and may vary over time.*
- That the cycle of financial difficulties and mental health problems can be intensified for people from culturally and linguistically diverse communities, due to the difficulty of navigating unfamiliar financial and health systems in Australia.*
- The following people should be familiar with cultural considerations when working with a person with a mental health problem who is from an Aboriginal or Torres Strait Islander background.^
- Financial counsellors should be familiar with the cultural considerations of working with the person from a culturally and linguistically diverse background.*
- That financial difficulties may negatively impact the person's ability to be a part of their community.*
- That mental health problems can contribute to financial difficulties, and vice versa, e.g. failing to pay bills because of depression, loss of income increasing anxiety and depression.*
- That overspending and financial difficulties can be a sign of a relapse of the person's mental health problems.*
- That the person with mental health problems may have increased medical expenses that limit financial resources.*
- That the person with mental health problems may incur debt to support a drug or alcohol problem.*
- That the person with financial difficulties may find it difficult to pay for treatment for their mental health problems, which may worsen their condition.*
- That financial stability may not be an important goal for some people in the early stages of recovery from mental illness.*
- That addressing financial difficulties early may reduce the negative impact on mental health.*
- That the process of debt recovery may have an impact on the person's mental health, e.g. harassment from debt collectors.*

*Endorsed in Round 1;^Endorsed in Round 2

**Financial Counsellors: Awareness about mental health problems and financial difficulties (cont.)**
That mental health problems can make it difficult for the person to communicate with creditors.*
That when a person sees no way back from their financial difficulties they might become suicidal/attempt suicide.*
That people with bipolar disorder are more vulnerable to problem gambling.*
Of how an episode of mania may affect a person's financial circumstances, e.g. not paying bills, job instability, over-spending.*
Of how depression may affect a person's financial circumstances, e.g. ignoring bills, job loss, over-spending to feel better.*
That the person who is making their repayments may still be experiencing financial difficulties and feeling distress.*
That the way the person perceives their financial situation is what impacts on their mental health, rather than their actual situation.*
That the person's financial decisions may be influenced by their support person. *
Financial counsellors should be aware of the importance of early identification and intervention for preventing or limiting relapse in the person with mental health problems. Please note that this does not mean knowing how to diagnose a mental illness.^
Financial counsellors should be aware of how they can reduce stressors that can contribute to relapse, i.e. a reoccurrence of a mental health problem in a person who is currently well.^
The following people should be aware that mental health problems can develop or worsen rapidly.^
The following people should be aware that dealing with financial matters (e.g. opening mail from creditors, visiting Centrelink) may be overwhelming for the person.^

*Endorsed in Round 1; ^Endorsed in Round 2; #Endorsed in Round 3

**All Financial Institution Staff: Awareness about mental health problems and financial difficulties**
Financial institution staff should be aware of the impact of the symptoms of mental health problems on the tasks necessary to keep up with financial responsibilities, e.g. problems with memory, decision-making and motivation, or conversely, over-confidence and over-spending in mania.*
Financial institution staff should be aware that having mental health problems is not necessarily an indicator of an inability to manage money. The effects of mental health problems will differ from one person to another, and may vary over time.*

*Endorsed in Round 1

All Financial Institution Staff: Awareness about mental health problems and financial difficulties (cont.)
Financial institution staff should be aware that mental health problems can contribute to financial difficulties, and vice versa, e.g. failing to pay bills because of depression, loss of income increasing anxiety and depression.*
Financial institution staff should know that overspending and financial difficulties can be a sign of a relapse of the person's mental health problems.*
Financial institution staff should know that the person with mental health problems may have increased medical expenses that limit financial resources.*
Financial institution staff should know that addressing financial difficulties early may reduce the negative impact on mental health.*
Financial institution staff should be aware of how depression may affect a person's financial circumstances, e.g. ignoring bills, job loss, over-spending to feel better.*
Financial institution staff should be aware that when a person sees no way back from their financial difficulties they might become suicidal/attempt suicide.*
Financial institution staff should know that the person who is making their repayments may still be experiencing financial difficulties and feeling distress.*
Financial institution staff should know that the process of debt recovery may have an impact on the person's mental health, e.g. harassment from debt collectors.*

*Endorsed in Round 1

Hardship staff: Awareness about mental health problems and financial difficulties
Hardship staff should know that the cycle of financial difficulties and mental health problems can be intensified for people from culturally and linguistically diverse communities, due to the difficulty of navigating unfamiliar financial and health systems in Australia.^
Hardship staff should be aware that dealing with financial matters (e.g. opening mail from creditors, visiting Centrelink) may be overwhelming for the person.^
Hardship staff should know that the person with financial difficulties may find it difficult to pay for treatment for their mental health problems, which may worsen their condition.^
Hardship staff should be aware that the person with mental health problems may not overspend but may have other problems with managing money, e.g. withdrawing all their money from the bank to keep it 'safe' at home.^
Hardship staff should know that financial stability may not be an important goal for some people in the early stages of recovery from mental illness.^
Hardship staff should be aware of how an episode of mania may affect a person's financial circumstances, e.g. not paying bills, job instability, over-spending.^

^Endorsed in Round 2

Hardship staff: Awareness about mental health problems and financial difficulties (cont.)
Hardship staff should know that the way the person perceives their financial situation is what impacts on their mental health, rather than their actual situation.#

#Endorsed in Round 3

Collection staff: Awareness about mental health problems and financial difficulties
Collection staff should know that financial stability may not be an important goal for some people in the early stages of recovery from mental illness.^

^Endorsed in Round 2

Branch Staff: Awareness about mental health problems and financial difficulties
Financial institution staff who are in customer facing roles should be familiar with the cultural considerations of working with the person from a culturally and linguistically diverse background.*

*Endorsed in Round 1

Mental Health Professional: Awareness about mental health problems and financial difficulties
Mental health professionals should know that the cycle of financial difficulties and mental health problems can be intensified for people from culturally and linguistically diverse communities, due to the difficulty of navigating unfamiliar financial and health systems in Australia.*
Mental health professionals should know that financial difficulties may negatively impact the person's ability to be a part of their community.*
Mental health professionals should be aware that mental health problems can contribute to financial difficulties, and vice versa, e.g. failing to pay bills because of depression, loss of income increasing anxiety and depression.*
Mental health professionals should know that overspending and financial difficulties can be a sign of a relapse of the person's mental health problems.*
Mental health professionals should know that the person with mental health problems may have increased medical expenses that limit financial resources.*

*Endorsed in Round 1

**Mental Health Professional: Awareness about mental health problems and financial difficulties (cont.)**
Mental health professionals should know that the person with mental health problems may incur debt to support a drug or alcohol problem.*
Mental health professionals should know that the person with financial difficulties may find it difficult to pay for treatment for their mental health problems, which may worsen their condition.*
Mental health professionals should know that financial stability may not be an important goal for some people in the early stages of recovery from mental illness.*
Mental health professionals should know that the person who is making their repayments may still be experiencing financial difficulties and feeling distress.*
Mental health professionals should know that the way the person perceives their financial situation is what impacts on their mental health, rather than their actual situation.*
Mental health professionals should know that addressing financial difficulties early may reduce the negative impact on mental health.*
Mental health professionals should know that the process of debt recovery may have an impact on the person's mental health, e.g. harassment from debt collectors.*
Mental health professionals should be aware that when a person sees no way back from their financial difficulties they might become suicidal/attempt suicide.*
Mental health professionals should be aware that people with bipolar disorder are more vulnerable to problem gambling.*
Mental health professionals should be aware of how an episode of mania may affect a person's financial circumstances, e.g. not paying bills, job instability, over-spending.*
Mental health professionals should encourage the person with bipolar disorder to delay making large purchases until they have discussed them with their support person.*
Mental health professionals should be aware of how depression may affect a person's financial circumstances, e.g. ignoring bills, job loss, over-spending to feel better.*
Mental health professionals should be aware that if the person's financial difficulties are addressed then their mental health may improve.^

*Endorsed in Round 1; ^Endorsed in Round 2

**The Support Person: Awareness about mental health problems and financial difficulties**
The support person should be aware that mental health problems can contribute to financial difficulties, and vice versa, e.g. failing to pay bills because of depression, loss of income increasing anxiety and depression.*

*Endorsed in Round 1

The Support Person: Awareness about mental health problems and financial difficulties (cont.)

The support person should know that financial difficulties may negatively impact the person's ability to be a part of their community.*

The support person should know that overspending and financial difficulties can be a sign of a relapse of the person's mental health problems.*

The support person should know that the person with mental health problems may have increased medical expenses that limit financial resources.*

The support person should know that the person with mental health problems may incur debt to support a drug or alcohol problem.*

The support person should know that the person with financial difficulties may find it difficult to pay for treatment for their mental health problems, which may worsen their condition.*

The support person should know that financial stability may not be an important goal for some people in the early stages of recovery from mental illness.*

The support person should know that the person who is making their repayments may still be experiencing financial difficulties and feeling distress.*

The support person should know that the way the person perceives their financial situation is what impacts on their mental health, rather than their actual situation.*

The support person should know that addressing financial difficulties early may reduce the negative impact on mental health.*

The support person should know that the process of debt recovery may have an impact on the person's mental health, e.g. harassment from debt collectors.*The following people should be aware that when a person sees no way back from their financial difficulties they might become suicidal/attempt suicide.*

The support person should be aware that people with bipolar disorder are more vulnerable to problem gambling.*

The support person should be aware of how an episode of mania may affect a person's financial circumstances, e.g. not paying bills, job instability, over-spending.*

The support person should encourage the person with bipolar disorder to delay making large purchases until they have discussed them with their support person.*

The support person should be aware of how depression may affect a person's financial circumstances, e.g. ignoring bills, job loss, over-spending to feel better.*

The support person should be aware of the financial behaviours that may indicate the person is becoming unwell.*

The support person should be aware of the various Centrelink benefits that are available to the person with mental health problems and financial difficulties.*

The support person should advise the person with mental health problems and financial difficulties to discuss with Centrelink their eligibility for benefits.*

*Endorsed in Round 1

Financial Counsellors: Stigma

Financial counsellors should know:

- How the stigmatising attitudes of others can have a negative impact on the person with mental health problems.*
- The myths surrounding mental health problems that lead to stigma and limit the potential earning capabilities of the person affected by mental health problems.*
- The potential effects of any stigmatising attitudes the financial counsellor or financial institution staff may have on the person with mental health problems.*
- The effects of the person's own stigma about their illness on their ability to deal with a financial difficulties.*

Financial counsellors should be aware that people with mental health problems can be intelligent, capable people going through a difficult time, like many other people in the community.^

*Endorsed in Round 1; ^Endorsed in Round 2

All Financial Institution Staff: Stigma

Financial institution staff should know that many people have limited financial literacy skills.*

Financial institution staff should be aware that people with mental health problems can be intelligent, capable people going through a difficult time, like many other people in the community.^

*Endorsed in Round 1; ^Endorsed in Round 2

Hardship Staff: Stigma

Hardship staff should be aware of how the stigmatising attitudes of others can have a negative impact on the person with mental health problems.^

Hardship staff should be aware of the potential effects of any stigmatising attitudes Financial institution staff may have on the person with mental health problems.^

Hardship staff should be aware of how the person's own stigmatising attitudes about their illness may impact on their ability to deal with financial difficulties.^

^Endorsed in Round 2

Collection staff: stigma

Collection staff should be aware of how the stigmatising attitudes of others can have a negative impact on the person with mental health problems.^

^Endorsed in Round 2

Branch staff: Stigma

Branch staff should be aware of how the stigmatising attitudes of others can have a negative impact on the person with mental health problems.^

Branch staff should be aware of the potential effects of any stigmatising attitudes Financial institution staff may have on the person with mental health problems.^

^Endorsed in Round 2

Mental Health Professional: Stigma

Mental health professionals should know that many people have limited financial literacy skills.*

*Endorsed in Round 1

The Support Person: Stigma

The support person should know that many people have limited financial literacy skills.^

^Endorsed in Round 2

Financial Counsellors: Support of clients with mental health problems and financial difficulties

Financial counsellors should allow extra time when working with the person with mental health problems, e.g. flexible approach to appointments, number of sessions, length of appointments.*

Financial counsellors should consider the following things when working with the person to develop financial strategies:

- Ability to engage meaningfully with financial institutions.*
- Ability to implement realistic repayment plans.*
- Long-term impact of the mental illness.*
- The person's capacity for and level of understanding.*
- The person's capacity for decision-making.*

*Endorsed in Round 1

Financial Counsellors: Support of clients with mental health problems and financial difficulties (cont.)

Financial counsellors should be aware of the various Centrelink benefits that are available to the person with mental health problems and financial difficulties.*

The financial counsellor should advise the person with mental health problems and financial difficulties to discuss with Centrelink their eligibility for benefits.*

If the person discloses mental health problems and asks for help, the financial counsellor should provide written material about appropriate professional help.#

Financial counsellors should be aware of:

- The mental health and disability support services available in the community. *
- The internal and external resources available to them should they feel distressed by their work with people with mental health problems.*
- What mental health services are available to their clients.*

Financial counselling services should update their information on external resources/community services regularly to ensure they are not out of date.^

Financial counsellors should be aware of what a financial administration order is and the implications for the client.*

Financial counsellors should know the relevant laws that affect interaction between the person with mental health problems and creditors, e.g. Disability Discrimination Act 1992, the Social Security Act, 1991.*

Financial counsellors should know their state or territory's mental health legislation and the implications for their responsibility in responding to the person's mental health crisis.*

Financial counsellors should be aware of what a financial administration order is and the implications for the person and the financial institution.*

Financial counsellors should have a general understanding of their state or territory laws regarding the appointment of a third party to manage the person's finances, e.g. guardianship, administration orders, financial management orders.^

Financial counsellors should have a thorough working knowledge of their state or territory laws regarding the appointment of a third party to manage the person's finances, e.g. guardianship, administration orders, financial management orders.^

*Endorsed in Round 1; ^Endorsed in Round 2

All Financial Institution Staff: Support of clients with mental health problems and financial difficulties

Financial institution staff should adopt a respectful and positive approach when working with the person with mental health problems.*

Financial institution staff should suspend collection activity to give the person sufficient time to consult a financial counsellor or mental health professional.

If the person with disclosed mental health problems is not adhering to arrangements tailored to their situation, Financial institution staff should:

- Encourage authorised third party representation, e.g. a professional, such as a community lawyer or financial counsellor, advocating on their behalf.*
- Ensure any authorised third party representatives are contacted.*

Financial institution staff should only initiate court action to pursue the person's debt as a last resort.*

If the person has disclosed mental health problems, Financial institution staff should offer to give the person a plain language summary of information and options discussed during their phone conversation.*

Financial institution staff should allow the person to choose their preferred method of contact. *

Financial institution staff should provide the following options for people with mental health problems to contact them about financial difficulties:

- Letters*
- Voice, e.g. telephone, Skype*

Financial institution staff should be aware of the internal and external resources available to them should they feel distressed by their work with people with mental health problems.*

The following people should be aware of what a financial administration order is and the implications for the client.*

The following people should be aware of what a financial administration order is and the implications for the person and the financial institution.*

*Endorsed in Round 1

Hardship Staff: Support of clients with mental health problems and financial difficulties

If it is suspected that the person in financial difficulties may have mental health problems, hardship staff should offer to refer them to financial counselling services, who can advocate on the person's behalf and make an appropriate referral.^

If the hardship staff suspects that the person in financial hardship may have mental health problems, they should offer to refer them to financial counselling services, who can advocate on the person's behalf and make an appropriate referral. ('Hardship' is defined as when 'the person' is being managed by a financial institution's hardship team.)^

If the person is being managed by a hardship team, that team should have the discretion to move from an automated to a tailored collection process.#

Hardship staff should know a few key mental health and disability support services available in their community.^

Hardship staff should have a general understanding of their state or territory laws regarding the appointment of a third party to manage the person's finances, e.g. guardianship, administration orders, financial management orders.^

^Endorsed in Round 2; #Endorsed in Round 3

Collection Staff: Support of clients with mental health problems and financial difficulties

Collection staff should have a general understanding of their state or territory laws regarding the appointment of a third party to manage the person's finances, e.g. guardianship, administration orders, financial management orders.^

^Endorsed in Round 2

Branch Staff: Support of clients with mental health problems and financial difficulties

Branch staff should have a general understanding of their state or territory laws regarding the appointment of a third party to manage the person's finances, e.g. guardianship, administration orders, financial management orders.#

#Endorsed in Round 3

Mental Health professional: Support of clients with mental health problems and financial difficulties

Mental health professionals should know the relevant laws that affect interaction between the person with mental health problems and creditors, e.g. Disability Discrimination Act 1992, the Social Security Act, 1991.*

Mental health professionals should know their state or territory's mental health legislation and the implications for their responsibility in responding to the person's mental health crisis.*

Mental health professionals should ask direct questions about the person's financial situation, and whether they have any financial difficulties, so they can refer the person for appropriate advice if necessary.*

Mental health professionals should discourage the person from ignoring their financial difficulties.*

Mental health professionals should tell the person that dealing with financial difficulties sooner rather than later may make it more manageable.*

Mental health professionals should encourage the person with mental health problems to contact their financial institution as soon as they think they will experience financial difficulties.*

Mental health professionals should be aware of what a financial administration order is and the implications for the person and the financial institution.*

Mental health professionals should be aware that there are a range of concessions and financial assistance available to the person and encourage them to apply for these, e.g. reduced car registration, council rates and energy costs to health care card holders.*

Mental health professionals should be aware of the various Centrelink benefits that are available to the person with mental health problems and financial difficulties.*

Mental health professionals advise the person with mental health problems and financial difficulties to discuss with Centrelink their eligibility for benefit.*

Mental health professionals should be aware of what a financial administration order is and the implications for the client.^

Mental health professionals should have a thorough working knowledge of their state or territory laws regarding the appointment of a third party to manage the person's finances, e.g. guardianship, administration orders, financial management orders.^

Mental health professionals should have a general understanding of their state or territory laws regarding the appointment of a third party to manage the person's finances, e.g. guardianship, administration orders, financial management orders.^

Mental health professionals should be aware that every state and territory has an ombudsman to deal with difficulties with energy providers.^

*Endorsed in Round 1; ^Endorsed in Round 2

**Mental Health professional: Support of clients with mental health problems and financial difficulties (cont.)**
Mental health professionals should be aware that utility providers (gas, electricity, water) have, by law, provisions for people in financial hardship, and should encourage the person to make and maintain contact with the utility provider and request 'hardship' assistance, once financial difficulties are evident.^
Mental health professionals should not make payment arrangements with creditors regarding the person's debt without consulting with a financial counsellor, as there are specific consumer protection laws that might mean the debt does not need to be paid.#
If the mental health professional has identified overspending as being a feature of the person's bipolar disorder, they should ask about financial difficulties.*

*Endorsed in Round 1; ^Endorsed in Round 2; #Endorsed in Round 3

**The Support Person: Support of clients with mental health problems and financial difficulties**
The following people should ask direct questions about the person's financial situation, and whether they have any financial difficulties, so they can refer the person for appropriate advice if necessary. - Support person*
The following people should discourage the person from ignoring their financial difficulties. - Support person*
The following people should be aware of what a financial administration order is and the implications for the person and the financial institution.*
The following people should be aware that people with mental health problems may have memory or concentration problems that require simple and concise information about their situation and that this information may need to be repeated. - Support person*
If the person appears to have symptoms of mental illness, the following people should ask them if they are having thoughts of suicide. - Support person*

*Endorsed in Round 1

The Financial Counsellor, Financial Institution Staff, Mental Health Professional and Support Person: Working together
Financial counsellors and Mental health professionals should work together so that the client service plan complements the treatment plan, with the person's consent. (A client service plan is a document that financial counsellors develop with people to identify financial issues and possible pathways for options and solutions to financial difficulties.)^
The financial counsellor, mental health professional and the support person should work with the person to determine what specific supports the person needs to enable them to make particular financial decisions.^
The financial counsellor should work with the mental health professional to determine whether the person is able to make specific decisions concerning their current financial situation, with the person's consent.#
The financial counsellor and the mental health professional should work together and deliver consistent messages to the person, with the person's consent.#
The financial counsellor and the mental health professional should be aware of and discuss any conflicting priorities between financial and treatment goals, with the person's consent.#
The financial counsellor should know of the positive role that financial institutions can play for people with mental health problems and financial difficulties, e.g. promoting financial inclusion.*
The financial counsellor should know that that most financial institutions will have policies about assistance for people who are experiencing financial difficulties.*
Mental health professionals should know the important role that financial counselling services play for people with mental health problems and financial difficulties. - Mental health professional*
Mental health professionals should explain what financial counsellors do and the potential benefits to the person.*
Mental health professionals should know what financial counselling services are available in their area and how to access them.*
Mental health professionals should provide the person with printed material about local financial counselling services.*
Mental health professionals should display signs about financial counselling services in their offices.*

^Endorsed in Round 2; #Endorsed in Round 3

The Financial Counsellor, Financial Institution Staff, Mental Health Professional and Support Person: Working together (cont.)

Mental health professionals should provide the person with oral information about financial counselling services in their area.*

Financial institutions should work with state and national financial counselling organisations in order to develop policies to better assist people with mental health problems and financial difficulties.*

Financial institution staff should know the important role that financial counselling services play for people with mental health problems and financial difficulties.*

If Financial institution staff are told by the person in financial difficulties that they have mental health problems, they should refer them to financial counselling services, who can advocate on the person's behalf and make an appropriate referral.*

If Financial institution staff are told by the person in financial hardship that they have mental health problems, they should refer them to financial counselling services, who can advocate on the person's behalf and make an appropriate referral.*

When Financial institution staff is working with the mental health professional to determine the person’s capacity, the following issues should be addressed:

- Ability to engage meaningfully with financial institutions.*
- The person’s capacity for and level of understanding.*
- The person’s capacity for decision making.*

Hardship staff should work with the financial counsellor to determine whether the person is able to make specific decisions concerning their current financial situation, with the person's consent.^

The following people should know the important role that financial counselling services play for people with mental health problems and financial difficulties. - Support person*

The following people should know of the positive role that financial institutions can play for people with mental health problems and financial difficulties, e.g. promoting financial inclusion. - Mental health professional*

If the person appears to have financial difficulties, the mental health professional should advise them to seek the help of a financial counsellor.*

The following people should know that most financial institutions will have policies about assistance for people who are experiencing financial difficulties. - Mental health professional*

Mental health professionals should be aware that tailored payment arrangements with financial institutions are possible for people with mental problems.*

The following people should know of the positive role that financial institutions can play for people with mental health problems and financial difficulties, e.g. promoting financial inclusion. - Support person*

The following people should know that most financial institutions will have policies about assistance for people who are experiencing financial difficulties. - Support person*

*Endorsed in Round 1

Financial Counsellors: Disclosing mental health problems

Financial counsellors should be aware that people with mental health problems may be reluctant to tell financial institutions about their condition, e.g. because of shame or fear of being refused credit.*

Financial counsellors should be aware that the person may be worried about how information about their mental health problems will be used.*

If the person decides to disclose their mental health problems to a financial institution, the financial counsellor should encourage the person to do the following:

- Decide what they hope to achieve by disclosure and check if this is realistic.*
- Ask to speak to a specialist team member or staff member who works with people who have mental health problems or financial hardship, or a senior customer service team member.*
- Keep records of who they have spoken to, what was said and when.*

Financial counsellors should inform the person that they may be asked to provide evidence of their mental health problems in order to reach the most suitable solution to the financial difficulties.*

If the person discloses mental health problems and asks for help, the financial counsellor should refer the client to appropriate professional help.^

Financial counsellors should inform the person that although they may have disclosed their mental health problems to a financial institution, Financial institution staff may not necessarily understand the impact of mental health problems on financial difficulties.^

*Endorsed in Round 1; ^Endorsed in Round 2; #Endorsed in Round 3

Financial Institution Staff: Disclosing mental health problems

When a person with financial difficulties discloses mental health problems, Financial institution staff should tell the person how the information will be used.*

*Endorsed in Round 1; ^Endorsed in Round 2; #Endorsed in Round 3

Financial Counsellors: Communication guidelines

Financial counsellors should be aware of their own attitudes about mental health problems and how these might affect their ability to respond to the person non-judgmentally.*

Financial counsellors should set aside any negative beliefs and reactions in order to focus on the needs of the person they are assisting.*

Financial counsellors should not express negative judgments to the person as this can get in the way of assisting them.*

Financial counsellors should use the following verbal skills to show the person that they are listening:

- Ask questions which show that they genuinely care, e.g. clarifying questions to show that they understand what the person is saying.*
- Check their understanding by re-stating what the person has said.*
- Summarise facts and feelings.*
- Listen not only to what the person says, but how they say it, i.e. their tone of voice.*
- Use minimal prompts, such as “I see” and “Ah”, when necessary, to keep the conversation going.*
- Be patient, even when the person may not be communicating well, may be repetitive or may be speaking slowly and unclearly.*
- Allow pauses and silences to give the person time to think or gather their thoughts.*
- Offer to contact the support person.^
- Offer to contact the person's mental health worker.^

Financial counsellors should avoid:

- Using a hostile or sarcastic tone when the person’s responses are not what they expect.*
- Trivialising the person’s experiences, e.g. telling them to “put a smile on their face,” to “get their act together,” or to “lighten up”.*
- Belittling or dismissing the person's feelings, e.g. attempting to say something positive like, "You don't seem that bad to me."*
- Speaking with a patronising tone of voice.*
- Being critical or expressing frustration at the person for being in a negative emotional state.*
- Interrupting the person when they are speaking, especially to give your opinion.*
- Confronting the person, unless it is necessary to prevent harmful or dangerous acts.*

When meeting the person face to face and the person is unable to communicate effectively (e.g. with mania), the financial counsellor should discourage the person from making any financial decisions until they are able to communicate effectively.^

*Endorsed in Round 1; ^Endorsed in Round 2

Financial Counsellors: Communication guidelines (cont.)

Financial counsellors should do the following when talking over the phone with the person:

- Take some time to listen.*
- Allow the person enough time to think and talk.*
- As far as possible, let the person set the pace and style of the conversation.*
- Listen non-judgmentally so that the person can feel like they have been heard and understood.*
- Show empathy, e.g. "this must be very difficult for you".*
- Convey acceptance to the person, i.e. respect the person's feelings and opinions even if you do not agree with them, not criticising or trivialising what the person is saying, avoiding judging what the person is saying.*
- Convey genuineness to the person, i.e. your non-verbal cues match what you are saying.*
- Don't label the person's feelings for them.*
- Don't pressure the person to talk about their feelings.*
- Be on the lookout for any indications that the person may be in crisis, e.g. suicidal intentions, non-suicidal self-injury.*
- Offer to call the person back at a better time.*
- Offer relevant resources, e.g. contact details for Lifeline, local mental health service.*

Financial counsellors should be familiar with and listen for signs that may indicate that the person may be:

- Feeling depressed, i.e. sadness, hopelessness, worthlessness, difficulty concentrating, difficulty making decisions, agitation, slow monotonous speech, etc.*
- Distressed, i.e. worry, irritability, impatience, pressured speech, crying, etc.*
- Confused or out of touch with reality, e.g. bizarre beliefs, paranoia, illogical thinking, etc.*

Financial counsellors should know how to communicate with a person who:

- Is very distressed.*
- Who appears to be behaving aggressively.*

*Endorsed in Round 1

Financial Counsellors: Communication guidelines (cont.)

If the person is becoming aggressive (face to face or one the phone), the financial counsellor should:

- Be aware that the person may overreact to negative words; therefore, use positive words (such as ‘Let’s stay calm’) instead of negative or patronising words (such as ‘don’t fight’).*
- Be aware that the person’s symptoms or fear causing their aggression might be made worse if you take certain steps (e.g. involve the police).*
- Remain as calm as possible.*
- Speak to the person slowly and confidently with a gentle, caring tone of voice.*
- Not respond in a hostile, disciplinary or challenging manner.*
- Not argue with the person.*
- Not threaten them as this may increase fear or prompt aggressive behaviour.*
- Avoid raising their voice or talking too fast.*
- Consider taking a break from the conversation to allow the person a chance to calm down.*
- Take any threats or warnings seriously, particularly if the person believes they are being persecuted.*
- If the person is threatening harm to themselves or others, explain that this cannot be kept confidential and try to ascertain the person's whereabouts. (over the phone)^
- If the person is threatening harm to themselves or others, take any necessary protective action, e.g. contact the police or mental health crisis team.^
- explain to the person that although you would like to help them, it is not possible when they are (state the unacceptable behaviour, e.g. yelling).#
- Stay calm and avoid nervous behaviour (e.g. shuffling your feet, fidgeting, making abrupt movements).*
- Do not restrict the person’s movement (e.g. if he or she wants to pace up and down the room).*
- Terminate the interview and seek assistance from a supervisor or manager, if there is concern about their own or the person’s safety.^
- Exercise good listening skills.^
- Try to ensure the safety of those around you.^
- not take the person's aggressive behaviour personally and understand that they may be feeling confused or frightened.^
- Take a break after the encounter and talk to a supervisor or manager.^

Financial counsellors should be aware that people with mental health problems may have memory and concentration problems.*

*Endorsed in Round 1; ^Endorsed in Round 2; #Endorsed in Round 3

Financial Counsellors: Communication guidelines (cont.)

Financial counsellors should tailor their approach and interaction to the way the person who is confused or out of touch with reality is behaving, e.g. if the person is suspicious, be sensitive to this and reassure them you are there to assist them. *

Financial counsellors should be aware that people with mental health problems may have memory or concentration problems that require simple and concise information about their situation and that this information may need to be repeated.*

If the person has memory and concentration problems, the financial counsellor should give the person a plain language summary of information and options discussed during their appointment.*

Where possible, and if memory problems are present, Financial counsellors should encourage the person to record important information in a diary.^

If possible, Financial counsellors should request that the person who may be confused or out of touch with reality finds a spot to talk that is quiet and free of distractions.*

If possible, Financial counsellors should finds a spot to talk that is quiet and free of background noise when they are talking to the person that may be confused or out of touch with reality.*

Financial counsellors should be aware that the person who is confused or out of touch with reality may be frightened by their thoughts and feelings.*

Financial counsellors should be aware that the person's confusion and fear about what is happening to them may lead them to deny that anything is wrong with them.*

When the person is denying that anything is wrong, the financial counsellor should encourage them to talk to someone they trust.*

When talking to the person who is confused or out of touch with reality, the financial counsellor should:

- Respond to the person's delusions without agreeing with them, by saying something like "that must be horrible for you" or "I can see that you are upset".*
- Respond to disorganised speech by communicating in an uncomplicated and succinct manner.*
- Repeat things if necessary.*
- Not assume the person cannot understand what is being said, even if their response is limited.*
- Be patient and allow plenty of time for the person to process the information and respond to what has been said.*

*Endorsed in Round 1; ^Endorsed in Round 2;

Financial Counsellors: Communication guidelines (cont.)

Financial counsellors should be aware that delusions and hallucinations are very real to the person. They should not:

- Dismiss, minimise or argue with the person about their confusion, delusions or hallucinations.*
- Act alarmed, horrified or embarrassed by the person's confusion, delusions or hallucinations.*
- Laugh at the person's confusion, delusions or hallucinations.*
- Encourage or inflame the person's paranoia, if the person exhibits paranoid behaviour.*
- not raise their voice as if the person is deaf.^

If the financial counsellor has listened non-judgmentally and the person remains in their negative emotional state they should offer the following:

- Referral to a telephone counselling service, e.g. Lifeline.*
- Information about local mental health services (verbally or in an email, or via the post).*

When there is serious concern about the person's safety, or in situations where the relevant actions do not seem to be working, the financial counsellor should

- Contact their immediate manager.*
- Contact their local mental health service.*
- Contact the authorised third party, if there is one, and discuss their concern.^

*Endorsed in Round 1

All Financial institution Staff: Communication

Financial institution staff should be aware of their own attitudes about mental health problems and how these might affect their ability to respond to the person non-judgmentally.*

Financial institution staff should set aside any negative beliefs and reactions in order to focus on the needs of the person they are assisting.*

Financial institution staff should not express negative judgments to the person as this can get in the way of assisting them.*

Financial institution staff should use the following verbal skills to show the person that they are listening:

- Ask questions which show that they genuinely care, e.g. clarifying questions to show that they understand what the person is saying.*
- Check their understanding by re-stating what the person has said.*
- Summarise facts and feelings.*

*Endorsed in Round 1

All Financial institution Staff: Communication (cont.)

Financial institution staff should use the following verbal skills to show the person that they are listening:

- Listen not only to what the person says, but how they say it, i.e. their tone of voice.*
- Use minimal prompts, such as "I see" and "Ah", when necessary, to keep the conversation going.*
- Be patient, even when the person may not be communicating well, may be repetitive or may be speaking slowly and unclearly.*
- Allow pauses and silences to give the person time to think or gather their thoughts.*

Financial institution staff should avoid:

- Using a hostile or sarcastic tone when the person's responses are not what they expect.*
- Trivialising the person's experiences, e.g. telling them to "put a smile on their face," to "get their act together," or to "lighten up".*
- Belittling or dismissing the person's feelings, e.g. attempting to say something positive like, "You don't seem that bad to me."*
- Speaking with a patronising tone of voice.*
- Being critical or expressing frustration at the person for being in a negative emotional state.
- Interrupting the person when they are speaking, especially to give your opinion.*
- Confronting the person, unless it is necessary to prevent harmful or dangerous acts.*

Financial institution staff should do the following when talking over the phone with the person:

- Take some time to listen.*
- Allow the person enough time to think and talk.*
- As far as possible, let the person set the pace and style of the conversation.*
- Listen non-judgmentally so that the person can feel like they have been heard and understood.*
- Show empathy, e.g. "this must be very difficult for you".*
- Convey acceptance to the person, i.e. respect the person's feelings and opinions even if you do not agree with them, not criticising or trivialising what the person is saying, avoiding judging what the person is saying.*
- Convey genuineness to the person, i.e. your non-verbal cues match what you are saying.*
- Don't label the person's feelings for them.*
- Don't pressure the person to talk about their feelings.*
- Be on the lookout for any indications that the person may be in crisis, e.g. suicidal intentions, non-suicidal self-injury.*
- Offer to call the person back at a better time.*

*Endorsed in Round 1; ^Endorsed in Round 2; #Endorsed in Round 3

All Financial institution Staff: Communication (cont.)

When meeting the person face to face and the person is unable to communicate effectively (e.g. with mania), Financial institution staff should discourage the person from making any financial decisions until they are able to communicate effectively.^

Financial institution staff should be familiar with and listen for signs that may indicate that the person is distressed, i.e. worry, irritability, impatience, pressured speech, crying, etc.*

Financial institution staff should know how to communicate with a person who is very distressed.*

Financial institution staff should know how to communicate with a person who appears to be behaving aggressively.*

When meeting the person face to face and the person is presenting aggressively, Financial institution staff should:

- Speak to the person slowly and confidently with a gentle, caring tone of voice.*
- Not respond in a hostile, disciplinary or challenging manner.*
- Not argue with the person.*

Not threaten them as this may increase fear or prompt aggressive behaviour.*

When meeting the person face to face and the person is presenting aggressively, Financial institution staff should (cont.):

- Avoid raising your voice or talking too fast.*
- Be aware that the person may overreact to negative words; therefore, use positive words (such as 'stay calm') instead of negative words (such as 'don't fight').*
- Stay calm and avoid nervous behaviour (e.g. shuffling your feet, fidgeting, making abrupt movements).*
- Do not restrict the person's movement (e.g. if he or she wants to pace up and down the room).*
- Consider taking a break from the conversation to allow the person a chance to calm down.*

When there is serious concern about the person's safety, or in situations where the relevant actions do not seem to be working, the following people should contact their immediate manager.*

If the person is becoming aggressive, Financial institution staff should:

- Remain as calm as possible.*
- Speak to the person slowly and confidently with a gentle, caring tone of voice.*
- Not respond in a hostile, disciplinary or challenging manner.*
- Not argue with the person.*
- Not threaten them as this may increase fear or prompt aggressive behaviour.*
- Avoid raising their voice or talking too fast.*

*Endorsed in Round 1; ^Endorsed in Round 2

All Financial institution Staff: Communication (cont.)

If the person is becoming aggressive, Financial institution staff should:

- Be aware that the person may overreact to negative words; therefore, use positive words (such as 'Let's stay calm') instead of negative or patronising words (such as 'don't fight').*
- Be aware that the person's symptoms or fear causing their aggression might be made worse if you take certain steps (e.g. involve the police).*
- Consider taking a break from the conversation to allow the person a chance to calm down.*
- Take any threats or warnings seriously, particularly if the person believes they are being persecuted.*

Be aware that the person's symptoms or fear causing their aggression might be exacerbated if you take certain steps (e.g. involve the police).^

If the person is becoming aggressive, Financial institution staff should (cont):

- Terminate the interview and seek assistance from a supervisor or manager, if there is concern about their own or the person's safety.^
- Exercise good listening skills.^
- Try to ensure the safety of those around you.^
- Do not take the person's aggressive behaviour personally and understand that they may be feeling confused or frightened.^
- Take a break after the encounter and talk to a supervisor or manager.^
- Should explain to the person that although you would like to help them, it is not possible when they are (state the unacceptable behaviour, e.g. yelling).#

Financial institution staff should be aware that people with mental health problems may have memory or concentration problems that require simple and concise information about their situation and that this information may need to be repeated.*

Financial institution staff should be familiar with and listen for signs that may indicate that the person may be confused or out of touch with reality, e.g. bizarre beliefs, paranoia, illogical thinking, etc.*

Financial institution staff should tailor their approach and interaction to the way the person who is confused or out of touch with reality is behaving, e.g. if the person is suspicious, be sensitive to this and reassure them you are there to assist them.*

*Endorsed in Round 1; ^Endorsed in Round 2; #Endorsed in Round 3

All Financial institution Staff: Communication (cont.)

Financial institution staff should be aware that delusions and hallucinations are very real to the person. They should not:

- Dismiss, minimise or argue with the person about their confusion, delusions or hallucinations.*
- Act alarmed, horrified or embarrassed by the person's confusion, delusions or hallucinations.*
- Laugh at the person's confusion, delusions or hallucinations.*
- Encourage or inflame the person's paranoia, if the person exhibits paranoid behaviour.*

Assume the person cannot understand what is being said, even if their response is limited.*

When talking to the person who is confused or out of touch with reality, Financial institution staff should:

- Respond to the person's delusions without agreeing with them, by saying something like "that must be horrible for you" or "I can see that you are upset".*
- Respond to disorganised speech by communicating in an uncomplicated and succinct manner.*
- Repeat things if necessary.*
- Be patient and allow plenty of time for the person to process the information and respond to what has been said.*

When talking over the phone to the person who is confused or out of touch with reality, Financial institution staff should:

- Should not raise their voice as if the person is deaf.^
- If the person is threatening harm to themselves or others, explain that this cannot be kept confidential and try to ascertain the person's whereabouts.^

If the person is threatening harm to themselves or others, take any necessary protective action, e.g. contact the police or mental health crisis team.^

When there is serious concern about the person's safety, or in situations where the relevant actions do not seem to be working, Financial institution staff should contact the authorised third party, if there is one, and discuss their concern.#

*Endorsed in Round 1; ^Endorsed in Round 2; #Endorsed in Round 3

Hardship: Communication

Hardship staff should be aware that the person who is confused or out of touch with reality may be frightened by their thoughts and feelings.^

Hardship staff should be aware that the person's confusion and fear about what is happening to them may lead them to deny that anything is wrong with them.^

^Endorsed in Round 2

Financial Counsellors: Policy and Procedures

Financial counsellors should receive basic mental health training, e.g. mental health first aid. (Mental health first aid is the help offered a person developing to a mental health problem or experiencing a mental health crisis. The first aid is given until appropriate professional help is received or until the crisis resolves.)*

Financial counsellors should be required to have continuing professional development that includes mental health training.*

The financial counselling service should have a list of statements that their staff can paraphrase, using their own words, to help guide the conversation when talking to the person who appears to be in a negative emotional state, e.g. “It can be important to talk to someone confidentially who is not a friend or family member about what has been happening. Are you currently speaking to someone who can give you that support?” or “It is understandable that you are anxious at a time like this given everything that is happening for you at the moment....Lots of people say that going through financial difficulties can be very stressful…”*

Financial counsellors should receive training on any documents or forms used when working with people with mental health problems, e.g. debt and mental illness evidence form, advance directive.^

Financial counsellors should receive basic counselling skills training.^

Any mental health training of financial counsellors should include:

- Hearing about the lived experience of people who have had mental health problems and financial difficulties.^
- Learning about the impact of mental health problems and financial difficulties on the family of the person.^
- contemporary values-based, recovery-oriented, person-centred, trauma-informed thinking, and not a 'chemical imbalance'/defective brain disease model.^

Any website for people with financial difficulties should have a link to information which assists people in a mental health crisis, e.g. Lifeline, *beyondblue*.#

*Endorsed in Round 1; ^Endorsed in Round 2; #Endorsed in Round 3

Financial Institutions: Policies and Procedures

Financial institutions should have policies and procedures for dealing with advance directives.*

Financial institutions should have a standard form allowing people with a history of mental health problems and financial difficulties to nominate a third party point of contact who can negotiate on their behalf, should they become acutely unwell.*

*Endorsed in Round 1

Financial Institutions: Policies and Procedures (cont.)

The financial institution should follow the hardship provisions in the most recent voluntary Code of Banking Practice.*

The financial institution should have a list of statements that their staff can paraphrase, using their own words, to help guide the conversation when talking to the person who appears to be in a negative emotional state, e.g. "It can be important to talk to someone confidentially who is not a friend or family member about what has been happening. Are you currently speaking to someone who can give you that support?" or "It is understandable that you are anxious at a time like this given everything that is happening for you at the moment....Lots of people say that going through financial difficulties can be very stressful..."*

Financial institutions should have a policy for working with people with mental health problems and financial difficulties.*

Financial institutions should make their policy for working with people with mental health problems and financial difficulties available to financial counsellors.*

Financial institutions should have specific procedures for working with the person with mental health problems in complex or sensitive situations, e.g. suicidal thoughts and behaviours, threats to harm others, sale of a family home.*

These procedures should include:

- Having a process to redirect the person away from the usual collections process to an appropriately skilled team/staff member.*
- Having a process for handing on calls when the staff member requires support to manage the situation.*
- Having a review panel with a range of expertise to ensure all appropriate options are considered.*
- Having a process for working with crisis situations, e.g. threat of harm to self or others.*
- Having processes for referring the person to mental health services.*

Having processes for referring the person to financial counselling services.*

Financial institutions should be flexible in their arrangements with people experiencing mental health problems and financial difficulties, e.g. changing payment arrangements as required, accepting oral rather than written confirmation.*

The tailored procedure should include the following options:

- Encouraging third party representation (e.g. a professional, such as a community lawyer or financial counsellor) advocating on their behalf.*
- deferring action when the person is acutely unwell or in a mental health crisis.#

Financial institutions should allow for extra time to work with people with mental health problems, e.g. longer conversations, additional time to provide written confirmation of communication, more frequent calls.*

*Endorsed in Round 1; #Endorsed in Round 3

**Financial Institutions: Policies and Procedures (cont.)**
Financial institutions that use specialist teams to work with people with mental health problems should also consider how the expertise that members of these teams possess can be shared with other colleagues via informal/semi-formal, in-house training courses.*
Financial institutions should have a process for training frontline/branch staff to identify people with mental health problems and financial difficulties and refer them to a specialised team within the bank, e.g. hardship team.*
Financial institutions should provide their staff with mental health awareness training that is tailored to their role and the amount of contact they are likely to have with people with mental health problems.*
Financial institutions should provide tailored mental health training for collections staff.*
Financial institutions should provide tailored mental health training for hardship staff.*
Financial institutions should provide training for their staff on how to assist someone over the phone who is having a mental health crisis.*
Financial institutions should ensure that any mental health training received by financial institution staff is revisited periodically.*
In providing relevant information about financial hardship to their customers, financial institutions should include mental illness as a possible contributor to financial difficulties.*
Financial institutions should publicise and share examples of good practice from within their own organisation with colleagues across the industry.)*
Financial institutions should work with state and national mental health organisations in order to develop policies that better assist people with mental health problems and financial difficulties.*
Financial institutions should work with community services organisations in order to develop policies that better assist people with mental health problems and financial difficulties.*
The financial institution should develop, in collaboration with mental health professionals and financial counsellors, a policy about which types of health professionals can provide evidence of the impact of mental health problems on financial difficulties.^
The financial institution should have a policy on how long the information on the debt and mental illness evidence form is held by the financial institution and this policy should be available to the person.^

*Endorsed in Round 1; ^Endorsed in Round 2

Financial Institutions: Policies and Procedures (cont.)

Any mental health training of financial institution staff should include:

- Hearing about the lived experience of people who have had mental health problems and financial difficulties.^
- Learning about the impact of mental health problems and financial difficulties on the family of the person.^

For people with mental health problems, financial institutions should have a tailored procedure for reviewing and taking action at key points in the collection process, e.g. sale of assets or sale of debt.^

Financial institutions should update their information on external resources/community services regularly to ensure they are not out of date.^

Any website for people with financial difficulties should have a link to information which assists people in a mental health crisis, e.g. Lifeline, *beyondblue*.#

^Endorsed in Round 2; #Endorsed in Round 3

Mental Health Professional: Policy and procedures

Mental health professionals should be trained in the following basic skills to assist the person to build financial stability:

- Identifying triggers and consequences of financial successes and failures.*
- Educating the person on the impact that their symptoms may have on their financial stability/financial difficulties.*
- Assisting the person to identify how their symptoms impact on their financial difficulties.*

Mental health professionals should have procedures for prompt referral to financial counselling services of people with financial difficulties.*

Mental health professionals should be trained in the following basic skills:

- Assessing the pattern or history of the person's financial concerns.^
- Assisting the person to identify behaviours that have led to the problem debt, e.g. impulsive spending, unstable work history.^

*Endorsed in Round 1; ^Endorsed in Round 2

Mental Health and Financial Difficulties Impact Form

The following information should be included in a Debt and Mental Illness Evidence Form:

- Name, contact details and profession of the person requesting that the form be completed, e.g. financial institution staff, the financial counsellor.*
- Signature of the mental health professional.*
- A disclosure statement indicating what this form could be used for in the future.*
- Details of how the mental health problems affect the person's ability to resolve their current financial difficulties.*
- Any factors that may be relevant to communicating effectively with the person about their financial difficulties, e.g. preferred mode of communication, comprehension problems.*
- The person's capacity for and level of understanding.*
- The person's capacity for decision-making.*
- A statement about how long the information on the form is valid for.*
- The person's signature to indicate the person's informed consent to give this form to the financial institution and the financial counsellor.*
- Details of the support the person is receiving with regards to their financial difficulties.^
- Option for listing an authorised third party representative who the financial institution can contact if they are unable to contact the person.

If the mental health professional has provided advice that the person is unable to make specific decisions concerning their current financial situation, this advice should be reviewed on a regular basis or when the person's mental condition changes significantly.*

A copy of the completed form should be given to the person (and their support person, where appropriate).^

The mental health professional should review this form when the person's mental health problems change and resubmit it if needed, with the person's consent^

*Endorsed in Round 1; ^Endorsed in Round 2
