# Supplementary material for: Assisting Australians with mental health problems and financial difficulties: a Delphi study to develop guidelines for financial counsellors, financial institution staff, mental health professionals and carers
Source: BMC Health Serv Res. 2015 Jun 3;15:218. doi: 10.1186/s12913-015-0868-2 (PMC4453216; doi:10.1186/s12913-015-0868-2)
Supplement: Additional file 5: — Rejected Items: List of the survey items that were not endorsed by all five panels. [file 12913_2015_868_MOESM5_ESM.pdf]

Additional file 2: Rejected Items (items that did not reach at least 80% endorsement by each panel)

Category 1a: General awareness about mental health problems for financial counsellors

Item	FIS	FC	MHP	CON	CAR
The financial counsellor should be aware of the range of mental illness treatments and their effects.*	77.2	88.1	70.3	84.0	77.8
The financial counsellor should be familiar with the following mental health first aid guidelines:					
• Eating disorders*	75.9	88.1	78.9	64.0	88.9
• Non-suicidal self-injury*	81.0	90.5	86.8	68.0	88.9
• Traumatic events*	88.9	100.0	89.5	68.0	100.0
• How to help a person with problem drug use^	92.4	100.0	97.4	76.0	100.0
• How to help a person with problem drinking.^	89.9	100.0	100.0	76.0	100.0

*Rejected in Round 1; ^Rejected in Round 2
FIS-Financial Institution Staff; FC-Financial Counsellor; MHP-Mental Health Professional; CON-Consumer; CAR-Carer

Category 1b: General awareness about mental health problems for all financial institution staff

Item	FIS	FC	MHP	CON	CAR
Financial institution staff should be aware of the types of mental illnesses.*	75.9	88.4	76.3	69.6	63.0
Financial institution staff should be aware of the causes of and risk factors for mental illnesses.*	75.9	86.0	67.6	66.7	74.1
Financial institution staff should be aware of the range of mental illness treatments and their effects.*	51.3	65.1	56.8	64.0	70.4
Financial institution staff should be aware of the importance of early identification and intervention for preventing or limiting relapse in the person with mental health problems.*	44.3	58.1	65.8	68.0	63.0
Financial institution staff should be aware of how they can reduce stressors that can contribute to relapse, i.e. a reoccurrence of a mental health problem in a person who is currently well.*	56.4	79.1	71.1	72.0	85.2
Financial institution staff should be aware that the level of support needed by the person with mental health problems will fluctuate, as the symptoms of most mental health problems come and go over time.*	54.4	79.1	84.2	96.0	85.2

*Rejected in Round 1
FIS-Financial Institution Staff; FC-Financial Counsellor; MHP-Mental Health Professional; CON-Consumer; CAR-Carer

Category 1b: General awareness about mental health problems for all financial institution staff (cont.)

Item	FIS	FC	MHP	CON	CAR
The financial institution staff who are in customer facing roles should be familiar with the following mental health first aid guidelines:					
• Panic attack*	72.2	90.5	92.1	80.0	96.3
• Assisting the Person Who is Suicidal*	73.8	97.6	92.1	87.5	92.6
Financial institution staff should be aware of the warning signs of suicide.*	87.5	95.3	86.8	68.0	85.2

*Rejected in Round 1
FIS-Financial Institution Staff; FC-Financial Counsellor; MHP-Mental Health Professional; CON-Consumer; CAR-Carer

Category 1c: General awareness about mental health problems for hardship staff

Item	FIS	FC	MHP	CON	CAR
Hardship staff should be aware of the types of mental illnesses.^	73.3	100.0	90.3	85.0	84.0
Hardship staff should be aware of the range of mental illness treatments and their effects.^	56.7	85.3	71.0	90.0	72.0

^Rejected in Round 2
FIS-Financial Institution Staff; FC-Financial Counsellor; MHP-Mental Health Professional; CON-Consumer; CAR-Carer

Category 1c: General awareness about mental health problems for hardship staff (cont.)

Item	FIS	FC	MHP	CON	CAR
Hardship staff should be aware of how they can reduce stressors that can contribute to relapse, i.e. a reoccurrence of a mental health problem in a person who is currently well.#	69.8	100.0	90.3	100.0	95.8
The financial institution hardship staff should be aware of how they can reduce stressors that can contribute to relapse, i.e. a reoccurrence of a mental health problem in a person who is currently well. #	69.8	100.0	90.3	100.0	95.8
Hardship staff should be aware of the importance of early identification and intervention for preventing or limiting relapse in the person with mental health problems.^	61.7	85.3	83.9	80.0	84.0
Hardship staff should be aware that the level of support needed by the person with mental health problems will fluctuate, as the symptoms of most mental health problems come and go over time.^	73.3	97.1	96.8	90.0	96.0
Hardship staff should be aware that mental health problems may take time, usually weeks or months, to develop.^	71.7	91.2	96.8	80.0	84.0

^Rejected in Round 2; #Rejected in Round 3
FIS-Financial Institution Staff; FC-Financial Counsellor; MHP-Mental Health Professional; CON-Consumer; CAR-Carer

Category 1c: General awareness about mental health problems for hardship staff (cont.)

Item	FIS	FC	MHP	CON	CAR
Hardship staff should be familiar with the mental health first aid guidelines for psychosis.^	46.7	94.1	90.3	80.0	96.0
Hardship staff should be familiar with the mental health first aid guidelines for panic attack.^	58.3	94.1	93.5	90.0	100.0

^Rejected in Round 2
FIS-Financial Institution Staff; FC-Financial Counsellor; MHP-Mental Health Professional; CON-Consumer; CAR-carer

Category 1d: General awareness about mental health problems for collection staff

Item	FIS	FC	MHP	CON	CAR
Collection staff should be aware of the signs and symptoms of mental illnesses.^	68.3	100.0	96.8	95.0	96.0
Collection staff should be aware of the types of mental illnesses.^	56.7	97.1	90.3	90.0	80.0
Collection staff should be aware of the range of mental illness treatments and their effects.^	41.7	79.4	67.7	85.0	72.0

^Rejected in Round 2
FIS-Financial Institution Staff; FC-Financial Counsellor; MHP-Mental Health Professional; CON-Consumer; CAR-Carer

Category 1d: General awareness about mental health problems for collection staff (cont.)

Item	FIS	FC	MHP	CON	CAR
Collection staff should be aware of the things which might indicate that the person has mental health problems, e.g. not attending appointments, not returning phone calls, not meeting repayment schedules, displaying unusual behaviours, lack of clarity of thought.^	66.7	100.0	96.8	100.0	96.0
Collection staff should be aware of the importance of early identification and intervention for preventing or limiting relapse in the person with mental health problems.^	46.7	85.3	80.6	85.0	84.0
Collection staff should be aware that the level of support needed by the person with mental health problems will fluctuate, as the symptoms of most mental health problems come and go over time.^	53.3	91.2	93.5	90.0	92.0
The following people should be aware of how they can reduce stressors that can contribute to relapse, i.e. a reoccurrence of a mental health problem in a person who is currently well.^	58.3	97.1	90.3	95.0	96.0
Collection staff should know how they can reduce stressors that may exacerbate the person's mental health problems.^	66.7	97.1	93.5	90.0	100.0

^Rejected in Round 2
FIS-Financial Institution Staff; FC-Financial Counsellor; MHP-Mental Health Professional; CON-Consumer; CAR-Carer

Category 1d: General awareness about mental health problems for collection staff (cont.)

Item	FIS	FC	MHP	CON	CAR
Collection staff should be aware that mental health problems may take time, usually weeks or months, to develop.^	55.0	91.2	93.5	80.0	84.0
Collection staff should be familiar with the mental health first aid guidelines for psychosis.^	39.0	91.2	83.9	80.0	96.0
Collection staff should be familiar with the mental health first aid guidelines for panic attack.^	48.3	94.1	90.3	90.0	100.0

^Rejected in Round 2
FIS-Financial Institution Staff; FC-Financial Counsellor; MHP-Mental Health Professional; CON-Consumer; CAR-Carer

Category 1e: General awareness about mental health problems for branch staff

Item	FIS	FC	MHP	CON	CAR
Branch staff should be aware of the types of mental illnesses.^	62.7	88.2	87.1	85.0	76.0
The financial institution branch staff should be aware of the signs and symptoms of mental illnesses. #	77.4	97.1	93.5	94.7	100.0
The financial institution branch staff should be aware of the signs and symptoms of mental illnesses.#	77.4	97.1	93.5	94.7	100.0

^Rejected in Round 2; #Rejected in Round 3
FIS-Financial Institution Staff; FC-Financial Counsellor; MHP-Mental Health Professional; CON-Consumer; CAR-Carer

Category 1e: General awareness about mental health problems for branch staff (cont.)

Item	FIS	FC	MHP	CON	CAR
Branch staff should be aware of the range of mental illness treatments and their effects.^	43.3	70.6	61.3	75.0	64.0
Branch staff should be aware of the things which might indicate that the person has mental health problems, e.g. not attending appointments, not returning phone calls, not meeting repayment schedules, displaying unusual behaviours, lack of clarity of thought.^	64.4	91.2	93.5	95.0	92.0
Branch staff should be aware of the importance of early identification and intervention for preventing or limiting relapse in the person with mental health problems.^	46.7	82.4	61.3	75.0	76.0
Branch staff should be aware that the level of support needed by the person with mental health problems will fluctuate, as the symptoms of most mental health problems come and go over time.^	56.7	82.4	74.2	89.5	84.0
Branch staff should know how they can reduce stressors that may exacerbate the person's mental health problems.^	65.0	94.1	83.9	90.0	96.0
Branch staff should be aware of how they can reduce stressors that can contribute to relapse, i.e. a reoccurrence of a mental health problem in a person who is currently well.^	56.7	88.2	77.4	85.0	92.0

^Rejected in Round 2
FIS-Financial Institution Staff; FC-Financial Counsellor; MHP-Mental Health Professional; CON-Consumer; CAR-Carer

Category 1e: General awareness about mental health problems for branch staff (cont.)

Item	FIS	FC	MHP	CON	CAR
Branch staff should be aware that mental health problems may take time, usually weeks or months, to develop.^	52.5	70.6	64.5	80.0	80.0
Branch staff should be aware that mental health problems can develop or worsen rapidly.^	76.7	88.2	71.0	80.0	88.0
Branch staff should be familiar with the mental health first aid guidelines for:					
• Psychosis^	50.0	82.4	74.2	75.0	92.0
• Panic attack.^	61.0	100.0	83.9	80.0	100.0
• Eating disorders*	53.2	71.4	52.8	60.0	63.0
• Non-suicidal self-injury.*	59.7	76.2	71.1	64.0	70.4
• Traumatic events*	73.4	85.7	71.1	64.0	81.5
• Problem drug use*	72.2	85.7	81.6	68.0	85.2
• Problem drinking.*	69.6	85.7	86.8	72.0	85.2

*Rejected in Round 1; ^Rejected in Round 2
FIS-Financial Institution Staff; FC-Financial Counsellor; MHP-Mental Health Professional; CON-Consumer; CAR-Carer

Category 1f: General awareness about mental health problems for contact centre staff

Item	FIS	FC	MHP	CON	CAR
Contact centre staff should be aware of the signs and symptoms of mental illnesses.^	65.0	97.1	83.9	95.0	92.0
Contact centre staff should be aware of the types of mental illnesses.^	51.7	88.2	80.6	80.0	72.0
Contact centre staff should be aware of the range of mental illness treatments and their effects.^	36.7	73.5	58.1	70.0	62.5
Contact centre staff should be aware of the things which might indicate that the person has mental health problems, e.g. not attending appointments, not returning phone calls, not meeting repayment schedules, displaying unusual behaviours, lack of clarity of thought.^	55.0	91.2	87.1	80.0	92.0
Contact centre staff should be aware of the importance of early identification and intervention for preventing or limiting relapse in the person with mental health problems.^	35.0	76.5	58.1	70.0	72.0
Contact centre staff should be aware that the level of support needed by the person with mental health problems will fluctuate, as the symptoms of most mental health problems come and go over time.^	43.3	79.4	71.0	85.0	84.0

^Rejected in Round 2
FIS-Financial Institution Staff; FC-Financial Counsellor; MHP-Mental Health Professional; CON-Consumer; CAR-Carer

Category 1f: General awareness about mental health problems for contact centre staff (cont.)

Item	FIS	FC	MHP	CON	CAR
Contact centre staff should be aware of how they can reduce stressors that can contribute to relapse, i.e. a reoccurrence of a mental health problem in a person who is currently well.^	50.0	88.2	74.2	85.0	88.0
Contact centre staff should know how they can reduce stressors that may exacerbate the person's mental health problems.^	58.3	91.2	80.6	85.0	96.0
Contact centre staff should be aware that mental health problems may take time, usually weeks or months, to develop.^	45.0	82.4	60.0	75.0	75.0
Contact centre staff should be aware that mental health problems can develop or worsen rapidly.^	65.0	88.2	64.5	80.0	88.0
Contact centre staff should be familiar with the mental health first aid guidelines for:					
• Psychosis^	35.0	85.3	60.0	70.0	84.0
• Panic attack.^	41.7	88.2	71.0	80.0	88.0

^Rejected in Round 2
FIS-Financial Institution Staff; FC-Financial Counsellor; MHP-Mental Health Professional; CON-Consumer; CAR-Carer

Category 2a: Awareness about mental health problems and financial difficulties for all financial institution staff

Item	FIS	FC	MHP	CON	CAR
Financial institution staff should be aware that people with bipolar disorder are more vulnerable to problem gambling.*	60.3	81.4	67.6	56.0	77.8
Financial institution staff should be aware that the person with mental health problems may not overspend but may have other problems with managing money, e.g. withdrawing all their money from the bank to keep it 'safe' at home.*	70.1	90.7	81.1	88.0	88.9
Financial institution staff should know that the person with mental health problems may incur debt to support a drug or alcohol problem.*	73.4	81.0	73.7	64.0	81.5
Financial institution staff should know that financial stability may not be an important goal for some people in the early stages of recovery from mental illness.*	76.3	90.7	73.7	80.0	81.5

*Rejected in Round 1
FIS-Financial Institution Staff; FC-Financial Counsellor; MHP-Mental Health Professional; CON-Consumer; CAR-Carer

Category 2b: Awareness about mental health problems and financial difficulties for hardship staff

Item	FIS	FC	MHP	CON	CAR
The financial institution hardship staff should know that the person with mental health problems may incur debt to support a drug or alcohol problem.#	75.5	91.2	71.0	80.0	87.5
Hardship staff should be familiar with cultural considerations when working with a person with a mental health problem who is from an Aboriginal or Torres Strait Islander backgRound.^	73.3	97.1	96.8	90.0	96.0

^Rejected in Round 2; #Rejected in Round 3
FIS-Financial Institution Staff; FC-Financial Counsellor; MHP-Mental Health Professional; CON-Consumer; CAR-Carer

Category 2c: Awareness about mental health problems and financial difficulties for collection staff

Item	FIS	FC	MHP	CON	CAR
Collection staff should know that the person with financial difficulties may find it difficult to pay for treatment for their mental health problems, which may worsen their condition.^	68.3	100.0	87.1	95.0	100.0

^Rejected in Round 2
FIS-Financial Institution Staff; FC-Financial Counsellor; MHP-Mental Health Professional; CON-Consumer; CAR-Carer

Category 2c: Awareness about mental health problems and financial difficulties for collection staff (cont.)

Item	FIS	FC	MHP	CON	CAR
The financial institution collection staff should be aware of how an episode of mania may affect a person's financial circumstances, e.g. not paying bills, job instability, over-spending.#	67.9	100.0	96.8	89.5	95.8
The financial institution collection staff should be aware that dealing with financial matters (e.g. opening mail from creditors, visiting Centrelink) may be overwhelming for the person.#	67.9	100.0	100.0	100.0	95.8
Collection staff should know that the way the person perceives their financial situation is what impacts on their mental health, rather than their actual situation.^	68.3	100.0	90.3	95.0	100.0
Collection staff should know that the person with mental health problems may incur debt to support a drug or alcohol problem.^	68.3	91.2	77.4	75.0	88.0
Collection staff should be aware that the person with mental health problems may not overspend but may have other problems with managing money, e.g. withdrawing all their money from the bank to keep it 'safe' at home.^	68.3	91.2	80.6	90.0	96.0

^Rejected in Round 2; #Rejected in Round 3
FIS-Financial Institution Staff; FC-Financial Counsellor; MHP-Mental Health Professional; CON-Consumer; CAR-Carer

Category 2c: Awareness about mental health problems and financial difficulties for collection staff (cont.)

Item	FIS	FC	MHP	CON	CAR
Collection staff should know that the cycle of financial difficulties and mental health problems can be intensified for people from culturally and linguistically diverse communities, due to the difficulty of navigating unfamiliar financial and health systems in Australia.^	70.0	97.1	93.5	100.0	100.0
Collection staff should be familiar with cultural considerations when working with a person with a mental health problem who is from an Aboriginal or Torres Strait Islander background.^	65.0	100.0	96.8	90.0	92.0

^Rejected in Round 2
FIS-Financial Institution Staff; FC-Financial Counsellor; MHP-Mental Health Professional; CON-Consumer; CAR-Carer

Category 2d: Awareness about mental health problems and financial difficulties for branch staff

Item	FIS	FC	MHP	CON	CAR
Branch staff should be aware that dealing with financial matters (e.g. opening mail from creditors, visiting Centrelink) may be overwhelming for the person.^	68.3	94.1	90.3	95.0	96.0

^Rejected in Round 2
FIS-Financial Institution Staff; FC-Financial Counsellor; MHP-Mental Health Professional; CON-Consumer; CAR-Carer

Category 2d: Awareness about mental health problems and financial difficulties for branch staff (cont.)

Item	FIS	FC	MHP	CON	CAR
Branch staff should know that the person with financial difficulties may find it difficult to pay for treatment for their mental health problems, which may worsen their condition.^	61.7	88.2	80.6	95.0	96.0
Branch staff should know that the way the person perceives their financial situation is what impacts on their mental health, rather than their actual situation.^	61.7	91.2	77.4	95.0	69.0
Branch staff should know that the person with mental health problems may incur debt to support a drug or alcohol problem.^	63.3	85.3	67.7	70.0	88.0
Branch staff should be aware that the person with mental health problems may not overspend but may have other problems with managing money, e.g. withdrawing all their money from the bank to keep it 'safe' at home.^	68.3	82.4	80.6	85.0	100.0
Branch staff should know that financial stability may not be an important goal for some people in the early stages of recovery from mental illness.^	71.7	85.3	74.2	90.0	88.0
Branch staff should be aware of how an episode of mania may affect a person's financial circumstances, e.g. not paying bills, job instability, over-spending.^	55.9	88.2	87.1	95.0	96.0

^Rejected in Round 2
FIS-Financial Institution Staff; FC-Financial Counsellor; MHP-Mental Health Professional; CON-Consumer; CAR-Carer

Category 2d: Awareness about mental health problems and financial difficulties for branch staff (cont.)

Item	FIS	FC	MHP	CON	CAR
Branch staff should know that the cycle of financial difficulties and mental health problems can be intensified for people from culturally and linguistically diverse communities, due to the difficulty of navigating unfamiliar financial and health systems in Australia.^	68.3	97.1	87.1	95.0	100.0
Branch staff should be familiar with cultural considerations when working with a person with a mental health problem who is from an Aboriginal or Torres Strait Islander background.^	65.0	100.0	90.3	90.0	92.0

^Rejected in Round 2
FIS-Financial Institution Staff; FC-Financial Counsellor; MHP-Mental Health Professional; CON-Consumer; CAR-Carer

Category 2e: Awareness about mental health problems and financial difficulties for contact centre staff

Item	FIS	FC	MHP	CON	CAR
Contact centre staff should be aware that dealing with financial matters (e.g. opening mail from creditors, visiting Centrelink) may be overwhelming for the person.^	54.2	97.1	77.4	90.0	96.0

^Rejected in Round 2
FIS-Financial Institution Staff; FC-Financial Counsellor; MHP-Mental Health Professional; CON-Consumer; CAR-Carer

Category 2e: Awareness about mental health problems and financial difficulties for contact centre staff (cont.)

Item	FIS	FC	MHP	CON	CAR
Contact centre staff should know that the person with financial difficulties may find it difficult to pay for treatment for their mental health problems, which may worsen their condition.^	53.3	88.2	70.0	90.0	95.8
Contact centre staff should know that the way the person perceives their financial situation is what impacts on their mental health, rather than their actual situation.^	47.5	94.1	67.7	90.0	96.0
Contact centre staff should know that the person with mental health problems may incur debt to support a drug or alcohol problem.^	55.9	82.4	61.3	70.0	80.0
Contact centre staff should be aware that the person with mental health problems may not overspend but may have other problems with managing money, e.g. withdrawing all their money from the bank to keep it 'safe' at home.^	51.7	85.3	61.3	85.0	96.0
Contact centre staff should know that financial stability may not be an important goal for some people in the early stages of recovery from mental illness.^	61.7	82.4	61.3	90.0	84.0
Contact centre staff should be aware of how an episode of mania may affect a person's financial circumstances, e.g. not paying bills, job instability, over-spending.^	43.1	82.4	80.6	85.0	96.0

^Rejected in Round 2
FIS-Financial Institution Staff; FC-Financial Counsellor; MHP-Mental Health Professional; CON-Consumer; CAR-Carer

Category 2e: Awareness about mental health problems and financial difficulties for contact centre staff (cont.)

Item	FIS	FC	MHP	CON	CAR
Contact centre staff should know that the cycle of financial difficulties and mental health problems can be intensified for people from culturally and linguistically diverse communities, due to the difficulty of navigating unfamiliar financial and health systems in Australia.^	55.0	94.1	83.9	85.0	100.0
Contact centre staff should be familiar with cultural considerations when working with a person with a mental health problem who is from an Aboriginal or Torres Strait Islander background.^	58.6	97.1	83.9	90.0	92.0

^Rejected in Round 2
FIS-Financial Institution Staff; FC-Financial Counsellor; MHP-Mental Health Professional; CON-Consumer; CAR-Carer

Category 3a: Knowledge of all financial institution staff about stigma

Item	FIS	FC	MHP	CON	CAR
Financial institution staff should be aware of the myths surrounding mental health problems that lead to stigma and limit the potential earning capabilities of the person affected by mental health problems.*	68.4	93.0	94.7	92.0	81.5
Financial institution staff should be aware of the effects of the person's own stigma about their illness on their ability to deal with a financial difficulties.*	66.7	81.4	84.2	84.0	88.9

*Rejected in Round 1
FIS-Financial Institution Staff; FC-Financial Counsellor; MHP-Mental Health Professional; CON-Consumer; CAR-Carer

Category 3b: Knowledge of hardship staff about stigma

Item	FIS	FC	MHP	CON	CAR
The financial institution hardship staff should be aware of the myths surrounding mental health problems that lead to stigma and limit the potential earning capabilities of the person affected by mental health problems.#	75.5	100.0	93.5	100.0	100.0

#Rejected in Round 3
FIS-Financial Institution Staff; FC-Financial Counsellor; MHP-Mental Health Professional; CON-Consumer; CAR-Carer

Category 3c: Knowledge of collection staff about stigma

Item	FIS	FC	MHP	CON	CAR
Collection staff should be aware of how the person's own stigmatising attitudes about their illness may impact on their ability to deal with financial difficulties.^	73.3	97.1	96.8	90.0	100.0
The financial institution collection staff should be aware of the potential effects of any stigmatising attitudes the financial institution staff may have on the person with mental health problems. #	79.2	100.0	100.0	100.0	100.0
Collection staff should be aware of the myths surrounding mental health problems that lead to stigma and limit the potential earning capabilities of the person affected by mental health problems.^	68.3	94.1	100.0	100.0	100.0

^Rejected in Round 2; #Rejected in Round 3
FIS-Financial Institution Staff; FC-Financial Counsellor; MHP-Mental Health Professional; CON-Consumer; CAR-Carer

Category 3d: Knowledge of branch staff about stigma

Item	FIS	FC	MHP	CON	CAR
Branch staff should be aware of how the person's own stigmatising attitudes about their illness may impact on their ability to deal with financial difficulties.^	73.3	94.1	90.3	90.0	100.0
Branch staff should be aware of the myths surrounding mental health problems that lead to stigma and limit the potential earning capabilities of the person affected by mental health problems.^	68.3	94.1	100.0	100.0	96.0

^Rejected in Round 2
FIS-Financial Institution Staff; FC-Financial Counsellor; MHP-Mental Health Professional; CON-Consumer; CAR-Carer

Category 3e: Knowledge of contact centre staff about stigma

Item	FIS	FC	MHP	CON	CAR
Contact centre staff should be aware of how the stigmatising attitudes of others can have a negative impact on the person with mental health problems.^	71.7	100.0	96.8	95.0	96.0
Contact centre staff should be aware of the potential effects of any stigmatising attitudes the financial institution staff may have on the person with mental health problems.^	68.3	100.0	93.5	95.0	100.0

^Rejected in Round 2
FIS-Financial Institution Staff; FC-Financial Counsellor; MHP-Mental Health Professional; CON-Consumer; CAR-Carer

Category 3e: Knowledge of contact centre staff about stigma

Item	FIS	FC	MHP	CON	CAR
Contact centre staff should be aware of how the person's own stigmatising attitudes about their illness may impact on their ability to deal with financial difficulties.^	61.4	94.1	83.9	85.0	100.0
Contact centre staff should be aware of the myths surrounding mental health problems that lead to stigma and limit the potential earning capabilities of the person affected by mental health problems.^	60.0	94.1	90.3	90.0	96.0

^Rejected in Round 2
FIS-Financial Institution Staff; FC-Financial Counsellor; MHP-Mental Health Professional; CON-Consumer; CAR-Carer

Category 4a: Financial counsellors support of clients with mental health problems and financial difficulties

Item	FIS	FC	MHP	CON	CAR
The financial counsellor should routinely ask new clients if they have ever been diagnosed with a mental illness.*	65.0	39.5	55.3	52.0	48.1
The financial counsellor should regularly ask all clients about current symptoms of mental health problems.*	48.1	30.2	40.5	48.0	25.9
If the person appears to have symptoms of mental health problems, the financial counsellor should ask about current mental health treatment.*	68.4	72.1	60.5	40.0	44.4

*Rejected in Round 1
FIS-Financial Institution Staff; FC-Financial Counsellor; MHP-Mental Health Professional; CON-Consumer; CAR-Carer

Category 4a: Financial counsellors support of clients with mental health problems and financial difficulties (cont.)

Item	FIS	FC	MHP	CON	CAR
If the person appears to have mental health problems for which they are not receiving treatment, the financial counsellor should advise them to seek professional mental health care.*	85.0	76.7	89.5	64.0	55.6
The financial counsellor should routinely screen for mental health problems in new clients by using a standardised screening questionnaire, e.g. a simple screening tool that is used to measure psychological distress.*	55.1	39.5	48.6	56.0	51.9
If the person appears to have symptoms of mental health problems, the financial counsellor should administer a standardised screening questionnaire, e.g. a simple screening tool that is used to measure psychological distress.*	73.8	39.5	44.7	52.0	63.0
The financial counsellor should regularly screen for mental health problems in all clients by using a standardised screening questionnaire, e.g. a simple screening tool that is used to measure psychological distress.*	57.0	27.9	31.6	48.0	48.1
The financial counsellor should ask all new clients if they or anyone in their family suffers from any illnesses as a subtle way of asking about mental health problems.^	55.0	44.1	48.4	60.0	60.0

*Rejected in Round 1; ^Rejected in Round 2
FIS-Financial Institution Staff; FC-Financial Counsellor; MHP-Mental Health Professional; CON-Consumer; CAR-Carer

Category 4a: Financial counsellors support of clients with mental health problems and financial difficulties (cont.)

Item	FIS	FC	MHP	CON	CAR
When the financial counsellor is completing a 'client service plan' with the person, they should take note of any medications the person takes as this may indicate if they have mental health problems.^	58.3	50.0	4109	60.0	52.0
If the person appears to have symptoms of mental health problems, the financial counsellor should ask if they have a diagnosis of mental illness.*	74.7	76.7	73.0	48.0	59.3
The financial counsellor should routinely ask new clients about current symptoms of mental health problems.*	53.8	46.5	56.8	60.0	37.0
The financial counsellor should ask the person about mental health problems in any family members who have contributed to financial difficulties, e.g. overspending due to mania or gambling problems.*	70.0	69.8	73.7	60.0	59.3
The financial counsellor should encourage the person to seek the support of their close family or friends in managing financial difficulties.*	63.3	46.5	52.8	68.0	57.7
The financial counsellor should advise the person that their family and friends are under no obligation to provide financial support.*	52.6	53.5	68.4	60.0	77.8

*Rejected in Round 1; ^Rejected in Round 2
FIS-Financial Institution Staff; FC-Financial Counsellor; MHP-Mental Health Professional; CON-Consumer; CAR-Carer

Category 4a: Financial counsellors support of clients with mental health problems and financial difficulties (cont.)

Item	FIS	FC	MHP	CON	CAR
The financial counsellor should offer to work with the person and their support person to develop an advance directive about handling financial difficulties arising from future illness-related behaviour.*	72.2	54.8	65.8	76.0	85.2
When developing an advance directive, the following topics should be considered, and relevant items included, depending on the individual situation:					
• Allowing the support person to manage the finances.*	67.1	58.1	60.5	68.0	55.6
• Restricting internet access in order to prevent overspending, e.g. online shopping and gambling.*	62.0	65.1	63.2	52.0	66.7
• Restricting or denying access to various sources of funds, e.g. credit cards or ATM cards.*	64.6	72.1	73.7	60.0	74.1
• Restricting access to transport, e.g. taking away car keys so the person can't go shopping.*	24.1	26.2	24.3	20.0	22.2
• Cancelling credit cards altogether.*	61.5	52.4	42.1	40.0	65.4
• Agreeing on a regular allowance.*	73.4	72.1	84.2	84.0	85.2

*Rejected in Round 1
FIS-Financial Institution Staff; FC-Financial Counsellor; MHP-Mental Health Professional; CON-Consumer; CAR-Carer

Category 4a: Financial counsellors support of clients with mental health problems and financial difficulties (cont.)

Item	FIS	FC	MHP	CON	CAR
When developing an advance directive, the following topics should be considered, and relevant items included, depending on the individual situation:					
• Restricting access to phone use, e.g. paid phone services.*	29.5	41.9	37.8	40.0	37.0
• Monitoring mail to ensure offers of credit cards are not taken up.*	50.0	66.7	42.1	52.0	66.7
• Requests for actions to be taken by the financial institutions, e.g. suspension of credit card when patterns of spending are unusual.*	66.7	73.8	68.4	72.0	81.5
The financial counsellor should encourage the person to arrange for financial power of attorney.*	88.6	59.5	73.7	52.0	74.1
The financial counsellor should help the person to prevent future financial difficulties by working out how their mental health problems may lead to overspending and other behaviour that results in financial difficulties.*	78.5	75.6	71.1	68.0	77.8
The financial counsellor should help the person work out their own warning signs that indicate that they may be at risk of relapse of mental health problems.*	61.3	69.0	42.1	60.0	66.7

*Rejected in Round 1
FIS-Financial Institution Staff; FC-Financial Counsellor; MHP-Mental Health Professional; CON-Consumer; CAR-Carer

Category 4a: Financial counsellors support of clients with mental health problems and financial difficulties (cont.)

Item	FIS	FC	MHP	CON	CAR
If the person appears to have symptoms of mental illness, the financial counsellor should ask them if they are having thoughts of suicide.*	61.3	88.4	75.7	72.0	63.0
The financial counsellor should routinely ask new clients if they are having thoughts of suicide.*	30.0	18.6	42.1	48.0	25.9
The financial counsellor should regularly ask all clients if they are having thoughts of suicide.*	17.5	16.3	23.7	32.0	18.5
The financial counsellor should advise the person to use the following strategies to help cope with strong/unhelpful emotions related to their mental health problems and financial difficulties:					
• Write down their worries and concerns.*	56.4	59.5	66.7	72.0	66.7
• Recall a past difficult situation that they overcame and identify what worked then and apply it to the current situation.*	48.1	73.2	55.3	52.0	68.0
• If they have a pre-existing medical condition that may be aggravated by stress, encourage them to talk to their doctor.*	69.6	92.9	75.7	68.0	77.8
• Avoid drugs and alcohol.*	53.2	61.9	59.5	48.0	66.7

*Rejected in Round 1
FIS-Financial Institution Staff; FC-Financial Counsellor; MHP-Mental Health Professional; CON-Consumer; CAR-Carer

Category 4a: Financial counsellors support of clients with mental health problems and financial difficulties (cont.)

Item	FIS	FC	MHP	CON	CAR
The financial counsellor should advise the person to use the following strategies to help cope with strong/unhelpful emotions related to their mental health problems and financial difficulties:					
• Take a problem solving approach to address their worries and concerns.*	62.8	71.4	78.9	75.0	70.4
• Identify the specific feelings they are experiencing and the concern/worry that may be underlying each of these feelings, e.g. worried about not being able to pay the rent or mortgage, or ashamed about losing their job.*	54.4	76.2	57.9	64.0	77.8
• Maintain a balanced diet and get some regular exercise.*	49.4	59.5	57.9	52.0	63.0
• Be aware that recovery from a significant stress takes time.*	68.4	88.1	75.7	64.0	85.2
• Identify friends and family members who they can talk to and who will help them to remain positive.*	67.1	92.9	86.8	80.0	88.9

*Rejected in Round 1
FIS-Financial Institution Staff; FC-Financial Counsellor; MHP-Mental Health Professional; CON-Consumer; CAR-Carer

Category 4a: Financial counsellors support of clients with mental health problems and financial difficulties (cont.)

Item	FIS	FC	MHP	CON	CAR
The financial counsellor should avoid trying to come up with answers to their problems.*	80.5	79.1	84.2	72.0	77.8
The financial counsellor should encourage the person with bipolar disorder to consider setting up a bank account that requires a second signature for large purchases.*	76.3	60.5	63.2	68.0	77.8
The financial counsellor should advise the person with bipolar disorder to avoid gambling altogether.*	69.2	65.1	52.6	60.0	60.0
The financial counsellor should encourage the person with bipolar disorder to delay making large purchases until they have discussed them with their support person.^	81.7	82.4	77.4	85.0	88.0
Where a person's mental health problems may lead to overspending, the financial counsellor should advise the person that it is possible to ask the financial institution to take a predetermined action if there are any unusual transactions.*	74.4	92.9	68.4	88.0	85.2
The financial counsellor should consider applying for an administration order when they believe the person continues to make decisions causing financial harm, where possible in consultation with the person and other appropriate professionals.*	87.3	42.9	75.7	72.0	84.6

*Rejected in Round 1
FIS-Financial Institution Staff; FC-Financial Counsellor; MHP-Mental Health Professional; CON-Consumer; CAR-Carer

Category 4a: Financial counsellors support of clients with mental health problems and financial difficulties (cont.)

Item	FIS	FC	MHP	CON	CAR
The financial counsellor should help the person to understand the interaction of finances, financial distress and mental health problems.*	81.3	78.6	68.4	80.0	77.8
If the person does not wish to seek the help of a mental health professional, the financial counsellor should explore the pros and cons of seeing a mental health professional with them.*	56.3	60.5	55.3	40.0	25.9
The financial counsellor should encourage the person to seek professional mental health care for any family member whose illness is contributing to financial difficulties in the family.*	78.8	72.1	78.9	72.0	55.6
The financial counsellor should advise the person that if they are more likely to overspend or sign up for credit cards when unwell, some financial institutions will add a note to their file, if requested.^	85.0	79.4	90.3	80.0	92.0

*Rejected in Round one; ^Rejected in Round 2
FIS-Financial Institution Staff; FC-Financial Counsellor; MHP-Mental Health Professional; CON-Consumer; CAR-Carer

Category 4a: Financial counsellors' support of clients with mental health problems and financial difficulties (cont.)

Item	FIS	FC	MHP	CON	CAR
The financial counsellor should encourage the person to form a 'support ne2rk' of people who would be helpful in a variety of situations, e.g. when emotional support is needed, when there is an increased risk of overspending or when they are short of money.^	86.7	85.3	77.4	85.0	84.0
The financial counsellor should know about the local self-help and support groups for people with mental health problems and recommend them when appropriate.^	88.3	94.1	64.5	90.0	76.0
Financial counsellors should encourage the person to disclose any mental health problems to the financial institution in order for them to reach the most suitable solution to the financial difficulties.*	82.5	62.8	57.9	68.0	66.7
The financial counsellor should advise the person that it is sometimes helpful to disclose their mental problems to the financial institution, depending on that financial institution's approach to people with mental health problems.*	85.0	88.4	86.8	72.0	80.8
If the person decides to disclose their mental health problems to the financial institution, the financial counsellor should discuss with the person what to disclose.^	65.0	50.0	67.7	90.0	84.0

*Rejected in Round 1; ^Rejected in Round 2
FIS-Financial Institution Staff; FC-Financial Counsellor; MHP-Mental Health Professional; CON-Consumer; CAR-Carer

Category 4b: All financial institution staffs' support of clients with mental health problems and financial difficulties

Item	FIS	FC	MHP	CON	CAR
Financial institution staff should be aware of how they can support the person with mental health problems in ways that promote recovery.*	64.6	69.8	62.2	84.0	81.5
Financial institution staff should be aware of how they can reduce stressors that can contribute to relapse, i.e. a reoccurrence of a mental health problem in a person who is currently well.*	57.0	81.4	76.3	80.0	88.9
The financial institution staff should choose their preferred method of contact when communicating with the person.*	48.7	42.9	32.4	24.0	42.3
The financial institution staff should provide the following options for people with mental health problems to contact them about financial difficulties:					
• Face to face meeting*	66.3	90.7	92.1	100.0	92.3
• Interactive electronic communication, e.g. internet chat, 2-way SMS*	57.5	72.1	83.3	84.0	80.8
Financial institution staff should encourage the person to arrange for financial power of attorney.*	32.9	42.9	47.4	28.0	33.3
If the person appears to have symptoms of mental illness, financial institution staff should ask them if they are having thoughts of suicide.*	30.8	60.5	54.1	44.0	48.1

*Rejected in Round 1
FIS-Financial Institution Staff; FC-Financial Counsellor; MHP-Mental Health Professional; CON-Consumer; CAR-Carer

Category 4b: All financial institution staffs' support of clients with mental health problems and financial difficulties (cont.)

Item	FIS	FC	MHP	CON	CAR
Financial institution staff should encourage the person with bipolar disorder to consider setting up a bank account that requires a second signature for large purchases.*	76.3	60.5	63.2	68.0	77.8
The financial institution staff should ask the person how their mental health problems impact on their ability to repay their debt.*	72.2	63.4	55.3	50.0	40.7
If an authorised third party (e.g. a financial counsellor) is not acting for the person, the financial institution staff should work with the mental health professional to determine the person's capacity to understand their financial situation.*	55.0	71.4	60.5	47.8	63.0
Where appropriate, the financial institution staff should encourage the person to find a support person.*	66.7	81.0	76.3	75.0	77.8
If the person with disclosed mental health problems is not adhering to arrangements tailored to their situation, then the financial institution staff can implement normal debt recovery procedures.*	75.0	15.0	26.3	24.0	18.5
If the person with disclosed mental health problems is not adhering to arrangements tailored to their situation, the financial institution staff should:					
• Make a site visit to assess the situation before proceeding with any sale of home.*	40.5	59.5	50.0	64.0	73.1

*Rejected in Round 1
FIS-Financial Institution Staff; FC-Financial Counsellor; MHP-Mental Health Professional; CON-Consumer; CAR-Carer

Category 4b: All financial institution staffs' support of clients with mental health problems and financial difficulties (cont.)

Item	FIS	FC	MHP	CON	CAR
If the person with disclosed mental health problems is not adhering to arrangements tailored					
• Defer action when the person is acutely unwell or in a mental health crisis.*	72.5	97.6	100.0	92.0	100.0
• Not sell debt to third parties.*	28.2	78.6	76.3	88.0	92.6
• Suspend interest payments when the person is acutely unwell or in a mental health crisis.*	46.8	92.9	89.5	84.0	92.6
Financial institution staff should ask the person about mental health problems in any family members who have contributed to financial difficulties, e.g. overspending due to mania or gambling problems.*	29.1	38.1	36.8	33.3	25.9
Financial institution staff should be aware of the various Centrelink benefits that are available to the person with mental health problems and financial difficulties.*	69.6	83.3	76.3	76.0	81.5
Financial institution staff should advise the person with mental health problems and financial difficulties to discuss with Centrelink their eligibility for benefits.*	62.8	78.6	76.3	68.0	73.1

*Rejected in Round 1
FIS-Financial Institution Staff; FC-Financial Counsellor; MHP-Mental Health Professional; CON-Consumer; CAR-Carer

Category 4b: All financial institution staffs' support of clients with mental health problems and financial difficulties (cont.)

Item	FIS	FC	MHP	CON	CAR
When a person with financial difficulties has mental health problems recorded, any financial institution staff member who deals with their case should ensure that the recorded information is still current.*	81.0	86.0	73.0	92.0	84.6
Financial institutions should provide a single point of contact for the person with mental health problems for the duration of the financial difficulties.*	69.2	93.0	84.2	79.2	88.9
Financial institutions should not sell or refer the debt of people with disclosed mental health problems.*	38.8	81.4	78.4	84.0	88.9
The financial institution staff and the person with mental health problems should come to an agreement about what is sufficient time to consult a financial counsellor or mental health professional.^	71.7	61.8	77.4	75.0	80.0
When the financial institution staff are referring a person who is in arrears to a financial counsellor, they should find out about current waiting list times in order to give the client sufficient time to consult with the financial counsellor.^	48.3	94.1	96.8	85.0	92.0
If a person has disclosed mental health problems and gives consent, the financial institution staff should ensure that this information is passed on in the event of any sale or referral of debt.*	63.8	83.3	62.2	68.0	65.4

*Rejected in Round 1; ^Rejected in Round 2
FIS-Financial Institution Staff; FC-Financial Counsellor; MHP-Mental Health Professional; CON-Consumer; CAR-Carer

Category 4b: All financial institution staffs' support of clients with mental health problems and financial difficulties (cont.)

Item	FIS	FC	MHP	CON	CAR
Financial institution staff should make the person with financial difficulties aware that if they disclose mental health problems to the financial institution, this will assist the financial institution staff to reach the most suitable solution to their financial difficulties.*	81.3	72.1	73.0	80.0	74.1
When a person with financial difficulties discloses mental health problems, Financial institution staff should seek permission to record this information for use by other staff members dealing with their case.*	87.3	76.2	70.3	88.0	66.7
When a person with financial difficulties discloses mental health problems, the financial institution staff should inform the person that they may be asked to provide evidence of their mental health problems.*	82.5	93.0	75.7	84.0	70.4

*Rejected in Round 1
FIS-Financial Institution Staff; FC-Financial Counsellor; MHP-Mental Health Professional; CON-Consumer; CAR-Carer

Category 4c: Hardship staffs' support of clients with mental health problems and financial difficulties

Item	FIS	FC	MHP	CON	CAR
The financial institution hardship staff should know what an advance directive is and how to act upon it.#	60.4	100.0	96.8	94.7	95.8
If a person has an advance directive in place, the hardship team should be the person's first point of call.^	68.3	50.0	51.6	70.0	64.0
If the person is being managed by a hardship team, that team should have the discretion to remove the account from any automated collection processes.*	94.9	93.0	50.0	72.0	70.4
Where a mental health problem has been disclosed, the financial institution staff should review the person's situation prior to the end of any hardship payment arrangements.*	73.8	97.6	75.7	88.0	88.9
Hardship staff should have a thorough working knowledge of their state or territory laws regarding the appointment of a third party to manage the person's finances, e.g. guardianship, administration orders, financial management orders.^	67.2	88.2	90.3	90.0	100.0
If it is suspected that the person in financial difficulties may have mental health problems, hardship staff should refer them to financial counselling services, who can advocate on the person's behalf and make an appropriate referral.^	80.0	70.6	80.6	85.0	76.0

*Rejected in Round 1; ^Rejected in Round 2; #Rejected in Round 3
FIS-Financial Institution Staff; FC-Financial Counsellor; MHP-Mental Health Professional; CON-Consumer; CAR-Carer

Category 4c: Hardship staffs' support of clients with mental health problems and financial difficulties (cont.)

Item	FIS	FC	MHP	CON	CAR
If the person with disclosed mental health problems is not adhering to arrangements tailored to their situation and there is a risk that they may lose their family home, hardship staff should make a referral to an agency who can help them find alternative accommodation before selling the family home.^	51.7	76.5	96.8	90.0	96.0
If the person appears to be in a negative state (i.e. confused or out of touch with reality, aggressive, highly distressed or anxious, or depressed) hardship staff should avoid trying to come up with answers to the person's problems.^	66.1	88.2	74.2	85.0	68.0

^Rejected in Round 2
FIS-Financial Institution Staff; FC-Financial Counsellor; MHP-Mental Health Professional; CON-Consumer; CAR-Carer

Category 4d: Collection staffs' support of clients with mental health problems and financial difficulties

Item	FIS	FC	MHP	CON	CAR
Collection staff should have a thorough working knowledge of their state or territory laws regarding the appointment of a third party to manage the person's finances, e.g. guardianship, administration orders, financial management orders.^	57.6	82.4	77.4	85.0	96.0

^Rejected in Round 2
FIS-Financial Institution Staff; FC-Financial Counsellor; MHP-Mental Health Professional; CON-Consumer; CAR-Carer

Category 4d: Collection staffs' support of clients with mental health problems and financial difficulties (cont.)

Item	FIS	FC	MHP	CON	CAR
If it is suspected that the person in financial difficulties may have mental health problems, collection staff should refer them to financial counselling services, who can advocate on the person's behalf and make an appropriate referral.^	65.0	70.6	74.2	85.0	75.0
If it is suspected that the person in financial difficulties may have mental health problems, the financial institution collection staff should offer to refer them to financial counselling services, who can advocate on the person's behalf and make an appropriate referral.#	79.2	94.1	96.8	89.5	100.0
Collection staff should know what an advance directive is and how to act upon it.^	66.7	88.2	100.0	95.0	96.0
If the person with disclosed mental health problems is not adhering to arrangements tailored to their situation and there is a risk that they may lose their family home, collection staff should make a referral to an agency who can help them find alternative accommodation before selling the family home.^	45.8	75.8	77.4	90.0	91.7
If the person appears to be in a negative state (i.e. confused or out of touch with reality, aggressive, highly distressed or anxious, or depressed) collection staff should avoid trying to come up with answers to the person's problems.^	67.8	85.3	77.4	85.0	75.0

^Rejected in Round 2; #Rejected in Round 3
FIS-Financial Institution Staff; FC-Financial Counsellor; MHP-Mental Health Professional; CON-Consumer; CAR-Carer

Category 4e: Branch staffs' support of clients with mental health problems and financial difficulties

Item	FIS	FC	MHP	CON	CAR
Branch staff should have a thorough working knowledge of their state or territory laws regarding the appointment of a third party to manage the person's finances, e.g. guardianship, administration orders, financial management orders.^	56.7	73.5	67.7	70.0	88.0
If it is suspected that the person in financial difficulties may have mental health problems, branch staff should refer them to financial counselling services, who can advocate on the person's behalf and make an appropriate referral.^	63.3	67.6	51.6	80.0	75.0
If it is suspected that the person in financial difficulties may have mental health problems, branch staff should offer to refer them to financial counselling services, who can advocate on the person's behalf and make an appropriate referral.^	71.7	94.1	93.5	90.0	92.0
Branch staff should know what an advance directive is and how to act upon it.^	66.7	85.3	90.3	80.0	96.0
If the person with disclosed mental health problems is not adhering to arrangements tailored to their situation and there is a risk that they may lose their family home, branch staff should make a referral to an agency who can help them find alternative accommodation before selling the family home.^	25.4	69.7	67.7	78.9	87.5

^Rejected in Round 2
FIS-Financial Institution Staff; FC-Financial Counsellor; MHP-Mental Health Professional; CON-Consumer; CAR-Carer

Category 4e: Branch staffs' support of clients with mental health problems and financial difficulties (cont.)

Item	FIS	FC	MHP	CON	CAR
If branch staff are concerned that the person may be experiencing mental health problems, they should talk with the person in a private room rather than at the desk with other customers present.^	73.3	82.4	71.0	90.0	100.0
If the person appears to be in a negative state (i.e. confused or out of touch with reality, aggressive, highly distressed or anxious, or depressed) branch staff should avoid trying to come up with answers to the person's problems.^	63.3	85.3	80.0	85.0	70.8

^Rejected in Round 2
FIS-Financial Institution Staff; FC-Financial Counsellor; MHP-Mental Health Professional; CON-Consumer; CAR-Carer

Category 4f: Contact centre staffs' support of clients with mental health problems and financial difficulties

Item	FIS	FC	MHP	CON	CAR
Contact centre staff should have a thorough working knowledge of their state or territory laws regarding the appointment of a third party to manage the person's finances, e.g. guardianship, administration orders, financial management orders.^	46.7	67.6	64.5	70.0	80.0

^Rejected in Round 2
FIS-Financial Institution Staff; FC-Financial Counsellor; MHP-Mental Health Professional; CON-Consumer; CAR-Carer

Category 4f: Contact centre staffs' support of clients with mental health problems and financial difficulties (cont.)

Item	FIS	FC	MHP	CON	CAR
Contact centre staff should have a general understanding of their state or territory laws regarding the appointment of a third party to manage the person's finances, e.g. guardianship, administration orders, financial management orders.^	71.7	82.4	80.0	90.0	92.0
If it is suspected that the person in financial difficulties may have mental health problems, contact centre staff should refer them to financial counselling services, who can advocate on the person's behalf and make an appropriate referral.^	50.0	70.6	54.8	80.0	69.6
If it is suspected that the person in financial difficulties may have mental health problems, contact centre staff should offer to refer them to financial counselling services, who can advocate on the person's behalf and make an appropriate referral.^	53.3	94.1	87.1	90.0	88.0
Contact centre staff should know what an advance directive is and how to act upon it.^	59.3	82.4	80.6	80.0	92.0
If the person with disclosed mental health problems is not adhering to arrangements tailored to their situation and there is a risk that they may lose their family home, contact centre staff should make a referral to an agency who can help them find alternative accommodation before selling the family home.^	18.6	60.6	64.5	78.9	70.8

^Rejected in Round 2
FIS-Financial Institution Staff; FC-Financial Counsellor; MHP-Mental Health Professional; CON-Consumer; CAR-Carer

Category 4f: Contact centre staffs' support of clients with mental health problems and financial difficulties (cont.)

Item	FIS	FC	MHP	CON	CAR
If the person appears to be in a negative state (i.e. confused or out of touch with reality, aggressive, highly distressed or anxious, or depressed) contact centre staff should avoid trying to come up with answers to the person's problems.^	60.0	87.9	77.4	80.0	66.7

^Rejected in Round 2
FIS-Financial Institution Staff; FC-Financial Counsellor; MHP-Mental Health Professional; CON-Consumer; CAR-Carer

Category 4g: Mental health professionals' support of clients with mental health problems and financial difficulties

Items	FIS	FC	MHP	CON	CAR
The mental health professional should be aware that water providers cannot disconnect mains water supplies because it is considered a health risk.*	74.4	88.1	89.5	96.0	92.6
The mental health professional should help the person prepare for their first appointment with a financial counsellor by helping them gather relevant paperwork or bills.*	61.3	55.8	63.2	80.0	66.7
If the person is unwilling or unable to talk to a financial counsellor, the mental health professional should:					
• Offer to accompany them to appointments with a financial counsellor.*	49.4	79.1	63.2	76.0	85.2

*Rejected in Round 1
FIS-Financial Institution Staff; FC-Financial Counsellor; MHP-Mental Health Professional; CON-Consumer; CAR-Carer

Category 4g: Mental health professionals' support of clients with mental health problems and financial difficulties (cont.)

Items	FIS	FC	MHP	CON	CAR
If the person is unwilling or unable to talk to a financial counsellor, the mental health professional should:					
• Seek the advice of a financial counsellor themselves on behalf of the person.*	50.0	65.9	60.5	44.0	55.6
• Assist the person to identify priority debts (e.g. debts that can lead to loss of home, imprisonment or disconnection of utilities) so that the person or the mental health professional can contact those creditors immediately.*	68.4	81.0	81.6	88.0	70.4
The mental health professional should routinely ask the person with financial difficulties if they are having thoughts of suicide.*	65.0	62.8	73.7	88.0	77.8
The mental health professional should encourage the person with bipolar disorder to consider setting up a bank account that requires a second signature for large purchases.*	68.8	62.8	63.2	72.0	77.8
The mental health professional should advise the person with bipolar disorder to avoid gambling altogether.*	77.2	69.8	63.2	72.0	74.1
The mental health professional should offer to work with the person and their support person to develop an advance directive about handling financial difficulties arising from future illness-related behaviour.*	72.2	54.8	65.8	76.0	85.2

*Rejected in Round 1
FIS-Financial Institution Staff; FC-Financial Counsellor; MHP-Mental Health Professional; CON-Consumer; CAR-Carer

Category 4g: Mental health professionals' support of clients with mental health problems and financial difficulties (cont.)

Items	FIS	FC	MHP	CON	CAR
The mental health professional should encourage the person to arrange for financial power of attorney.*	80.8	69.0	68.4	52.0	80.8
The mental health professional should consider applying for an administration order when they believe the person continues to make decisions causing financial harm, where possible in consultation with the person and other appropriate professionals.*	77.2	76.2	73.7	80.0	81.5
Mental health professionals should encourage the person to disclose any mental health problems to the financial institution in order for them to reach the most suitable solution to the financial difficulties.*	78.5	60.5	65.8	68.0	73.1
The mental health professional should advise the person that it is sometimes helpful to disclose their mental problems to the financial institution, depending on that financial institution's approach to people with mental health problems.*	82.3	86.0	89.5	72.0	80.8
The mental health professional should offer to write a letter that the person can give to the financial institution stating how their mental health problems have an impact on their ability to manage their finances.*	87.5	90.7	64.9	84.0	77.8

*Rejected in Round 1
FIS-Financial Institution Staff; FC-Financial Counsellor; MHP-Mental Health Professional; CON-Consumer; CAR-Carer

Category 4g: Mental health professionals' support of clients with mental health problems and financial difficulties (cont.)

Items	FIS	FC	MHP	CON	CAR
The mental health professional and the person should agree on what information will be provided to the financial counsellor concerning how the person's mental health problems affect their ability to manage money or repay their debt.^	81.7	76.5	90.3	95.0	92.0
If the person decides to disclose their mental health problems to the financial institution, the mental health professional should discuss with the person what to disclose.#	69.8	82.4	64.5	89.5	83.3

^Rejected in Round 2; #Rejected in Round 3
FIS-Financial Institution Staff; FC-Financial Counsellor; MHP-Mental Health Professional; CON-Consumer; CAR-Carer

Category 4h: Support persons' support of clients with mental health problems and financial difficulties

Item	FIS	FC	MHP	CON	CAR
The support person should proactively provide the person with practical help with financial matters, e.g. assisting with budgeting, setting up automatic bill payments, attending appointments with banking staff and the financial counsellor.*	62.5	46.5	52.6	66.7	63.0

*Rejected in Round 1
FIS-Financial Institution Staff; FC-Financial Counsellor; MHP-Mental Health Professional; CON-Consumer; CAR-Carer

Category 4h: Support persons' support of clients with mental health problems and financial difficulties (cont.)

Item	FIS	FC	MHP	CON	CAR
The support person should reassure the person that there is no such thing as unsolvable financial difficulties.*	70.0	67.4	76.3	68.0	88.9
The support person should encourage the person to arrange for financial power of attorney.*	75.9	64.3	65.8	52.0	77.8
The support person should encourage the person with bipolar disorder to consider setting up a bank account that requires a second signature for large purchases.*	78.8	62.8	57.9	68.0	77.8
The support person should advise the person with bipolar disorder to avoid gambling altogether.*	78.5	69.8	65.8	68.0	74.1
The support person should offer the person practical help with financial matters, e.g. assisting with budgeting, setting up automatic bill payments and attending appointments with the financial institution and the financial counsellor.*	83.5	69.8	81.6	68.0	74.1
The support person should consider applying for an administration order when they believe the person continues to make decisions causing financial harm, where possible in consultation with the person and other appropriate professionals.*	79.7	80.5	78.9	72.0	88.9

*Rejected in Round 1
FIS-Financial Institution Staff; FC-Financial Counsellor; MHP-Mental Health Professional; CON-Consumer; CAR-Carer

Category 4h: Support persons' support of clients with mental health problems and financial difficulties (cont.)

Item	FIS	FC	MHP	CON	CAR
The support person should encourage the person to disclose any mental health problems to the financial institution in order for them to reach the most suitable solution to the financial difficulties.*	78.5	60.5	60.5	60.0	70.4
The support person should advise the person that it is sometimes helpful to disclose their mental problems to the financial institution, depending on that financial institution's approach to people with mental health problems.*	82.3	81.0	86.8	64.0	84.6

*Rejected in Round 1
FIS-Financial Institution Staff; FC-Financial Counsellor; MHP-Mental Health Professional; CON-Consumer; CAR-Carer

Category 5: Financial counsellors, financial institution staff, mental health professionals and the support person working together

Item	FIS	FC	MHP	CON	CAR
The financial counsellor and the mental health professional should work together with the person and their support person to develop an advance directive that would include handling financial difficulties arising from future illness-related behaviour.*	75.9	67.4	73.7	95.8	92.6
The financial counsellor should encourage the person with mental health problems to make their own appointment with a mental health service.*	76.9	69.8	73.7	72.0	63.0

*Rejected in Round 1
FIS-Financial Institution Staff; FC-Financial Counsellor; MHP-Mental Health Professional; CON-Consumer; CAR-Carer

Category 5: Financial counsellors, financial institution staff, mental health professionals and the support person working together (cont.)

Item	FIS	FC	MHP	CON	CAR
The financial counsellor should help the person with mental health problems make an appointment with a mental health service.*	77.2	61.9	47.4	52.0	61.5
The financial counsellor should make the appointment with the mental health service for the person with mental health problems.*	36.4	16.3	15.8	8.0	7.4
If the financial institution staff suspects the person has mental health problems, they should refer the person to the hardship team.*	58.8	72.1	50.0	68.0	55.6
If a hardship team member suspects the person has mental health problems, they should encourage them to seek help from a mental health professional.*	52.5	72.1	84.2	75.0	70.4
If the person appears to have financial difficulties, the mental health professional should make a referral to a financial counsellor.*	73.1	69.8	63.2	72.0	70.4
The financial counsellor and the mental health professional should work together with the person to discuss the option of an advance directive, with the person's consent.^	80.0	73.5	100.0	95.0	96.0
Where possible, the mental health professional should offer the opportunity for the financial counsellor to meet with the person at the mental health services premises.*	57.5	34.9	44.7	64.0	51.9

*Rejected in Round 1; ^Rejected in Round 2
FIS-Financial Institution Staff; FC-Financial Counsellor; MHP-Mental Health Professional; CON-Consumer; CAR-Carer

Category 5: Financial counsellors, financial institution staff, mental health professionals and the support person working together (cont.)

Item	FIS	FC	MHP	CON	CAR
Where possible, the financial counsellor should offer the opportunity for the mental health professional to meet with the person on the financial counselling premises.*	55.4	44.2	55.3	68.0	59.3
The financial counsellor should work with the person and their support person to develop an advance directive about handling financial difficulties arising from future illness-related behaviour.*	72.2	54.8	65.8	76.0	85.2
The mental health professional should work with the person and their support person to develop an advance directive about handling financial difficulties arising from future illness-related behaviour.*	62.3	67.4	64.9	76.0	81.5
Financial institution staff should be aware of the mental health and disability support services available in the community.*	81.8	72.1	86.8	68.0	70.4
The financial counsellor should provide clients with printed material about local mental health services.*	85.0	74.4	73.7	80.0	63.0
The financial counsellor should display posters or other information about mental health services in their offices.*	91.3	65.1	89.5	76.0	81.5

*Rejected in Round 1
FIS-Financial Institution Staff; FC-Financial Counsellor; MHP-Mental Health Professional; CON-Consumer; CAR-Carer

Category 5: Financial counsellors, financial institution staff, mental health professionals and the support person working together (cont.)

Item	FIS	FC	MHP	CON	CAR
If the financial institution staff suspect that the person in financial hardship may have mental health problems, they should refer them to financial counselling services, who can advocate on the person's behalf and make an appropriate referral.*	73.8	97.6	78.9	84.0	85.2
The mental health professional should refer the person with poor money management skills to a financial counsellor.*	87.2	82.9	70.3	100.0	96.0
The financial counsellor should work with the mental health professional to determine whether the person is able to make specific decisions concerning their current financial situation.*	83.5	72.1	86.5	76.0	77.8
The financial counsellor and the mental health professional should work together and deliver consistent messages to the person.^	83.3	61.8	58.1	95.0	80.0
The financial counsellor and the mental health professional should be aware of and discuss any conflicting priorities between financial and treatment goals.^	81.7	70.6	64.5	95.0	72.0
The financial counsellor and the mental health professional should communicate regularly about the person they are both working with, with the person's consent and with the person knowing the content of the conversation.^	78.3	67.6	80.6	95.0	96.0

*Rejected in Round 1; ^Rejected in Round 2
FIS-Financial Institution Staff; FC-Financial Counsellor; MHP-Mental Health Professional; CON-Consumer; CAR-Carer

Category 5: Financial counsellors, financial institution staff, mental health professionals and the support person working together (cont.)

Item	FIS	FC	MHP	CON	CAR
Collection staff should work with the financial counsellor to determine whether the person is able to make specific decisions concerning their current financial situation, with the person's consent.^	56.7	82.4	89.7	80.0	79.2
Branch staff should work with the financial counsellor to determine whether the person is able to make specific decisions concerning their current financial situation, with the person's consent.^	47.5	64.7	69.0	75.0	75.0
Contact centre staff should work with the financial counsellor to determine whether the person is able to make specific decisions concerning their current financial situation, with the person's consent.^	37.1	67.6	65.5	68.4	62.5
The financial counsellor should consider contacting the financial institution, with the person's consent, to explain how the mental health problems may affect the person's ability to manage their finances.^	85.0	94.1	74.2	70.0	88.0
The mental health professional should follow-up with the financial counsellor regarding the person's progress, with the person's informed consent.*	73.4	81.0	84.2	76.0	92.6

*Rejected in Round 1; ^Rejected in Round 2
FIS-Financial Institution Staff; FC-Financial Counsellor; MHP-Mental Health Professional; CON-Consumer; CAR-Carer

Category 5: Financial counsellors, financial institution staff, mental health professionals and the support person working together (cont.)

Item	FIS	FC	MHP	CON	CAR
The mental health professional should follow-up with the person regarding the progress they are making with the financial counsellor.*	73.8	86.0	92.1	92.0	96.3
The financial counsellor and the mental health professional should work together so that the financial plans complement the treatment plan.*	74.4	81.4	84.2	84.0	88.0
The financial counsellor and the mental health professional should communicate regularly about the person they are both working with, with that person's permission.*	72.5	76.7	73.7	76.0	84.6
The financial counsellor and the mental health professional should inform the person about the content of any discussion between them.#	81.1	73.5	90.3	94.7	83.3
The financial counsellor should consider working with the person's support person, with the person's permission.*	87.5	67.4	78.9	80.0	92.6
The financial counsellor should encourage the person to discuss their financial difficulties with their support person.*	73.1	69.8	86.5	76.0	81.5

*Rejected in Round 1; #Rejected in Round 3
FIS-Financial Institution Staff; FC-Financial Counsellor; MHP-Mental Health Professional; CON-Consumer; CAR-Carer

Category 5: Financial counsellors, financial institution staff, mental health professionals and the support person working together (cont.)

Item	FIS	FC	MHP	CON	CAR
The financial counsellor should ask the mental health professional to write a letter that the person can give to the financial institution stating how their mental health problems impact on their ability to manage their finances.*	80.0	83.7	57.9	80.0	70.4
Financial institution staff should work with the financial counsellor to determine the person's capacity to understand their financial situation.*	90.0	87.8	81.6	54.2	70.4
When financial institution staff are working with the mental health professional to determine the person's capacity, the following issues should be addressed:					
• Ability to implement repayment plans.*	86.1	73.8	81.6	91.3	96.3
• Long-term impact of the mental illness.*	87.5	88.1	71.1	70.8	81.5
The person should be present at any meeting between the financial counsellor, the mental health professional and the support person.^	53.3	41.2	77.4	60.0	68.0
Where possible, the financial counsellor, mental health professional and support person should work together with the person and other appropriate professionals when making a decision about power of attorney.^	91.7	73.5	77.4	90.0	92.0

*Rejected in Round 1; ^Rejected in Round 2
FIS-Financial Institution Staff; FC-Financial Counsellor; MHP-Mental Health Professional; CON-Consumer; CAR-Carer

RA1 15/12/2014 3:32 PM
Comment [1]: Note that this item was missing the scores and that they have been added in.

Category 5: Financial counsellors, financial institution staff, mental health professionals and the support person working together (cont.)

Item	FIS	FC	MHP	CON	CAR
The financial institution collection staff should know a few key mental health and disability support services available in their community.#	71.7	97.1	100.0	94.7	95.8
The financial institution branch staff should know a few key mental health and disability support services available in their community.#	75.5	97.1	87.1	94.7	95.8

#Rejected in Round 3
FIS-Financial Institution Staff; FC-Financial Counsellor; MHP-Mental Health Professional; CON-Consumer; CAR-Carer

Category 6a: Communication guidelines for financial counsellors

Item	FIS	FC	MHP	CON	CAR
When meeting the person face to face and the person is unable to communicate effectively (e.g. with mania), the financial counsellor should:					
• Encourage them to write down their responses to questions.*	68.8	42.9	36.8	48.0	60.0

*Rejected in Round 1
FIS-Financial Institution Staff; FC-Financial Counsellor; MHP-Mental Health Professional; CON-Consumer; CAR-Carer

Category 6a: Communication guidelines for financial counsellors (cont.)

Item	FIS	FC	MHP	CON	CAR
When meeting the person face to face and the person is unable to communicate effectively (e.g. with mania), the financial counsellor should:					
• Consider asking structured rather than open-ended questions.*	73.8	78.6	71.1	80.0	76.9
• Terminate the interview and reschedule the appointment.^	58.3	79.4	61.3	70.0	72.0
• Ask the person if they would like to terminate the appointment and reschedule.^	88.3	70.6	67.7	95.0	88.0
• Terminate the interview and reschedule the appointment to a time when a support person or mental health worker can attend with the person.^	83.3	79.4	71.0	80.0	80.0
When meeting the person face to face and the person is presenting aggressively, the financial counsellor should:					
• Consider inviting the person to sit down if they are standing.*	74.7	93.0	84.2	92.0	92.3
• Terminate the interview and reschedule the appointment.^	71.7	50.0	56.7	85.0	70.8
• Take a deep breath to calm and ground themselves.^	76.7	64.7	87.1	90.0	88.0

*Rejected in Round 1; ^Rejected in Round 2
FIS-Financial Institution Staff; FC-Financial Counsellor; MHP-Mental Health Professional; CON-Consumer; CAR-Carer

Category 6a: Communication guidelines for financial counsellors (cont.)

Item	FIS	FC	MHP	CON	CAR
When meeting the person face to face and the person is presenting aggressively, the financial counsellor should:					
• State that the aggression is unacceptable behaviour.^	55.0	41.2	38.7	75.0	56.0
• Validate the person's feeling of frustration and anger.^	68.3	82.4	87.1	90.0	76.0
• Encourage them to get professional help, if appropriate.^	85.0	87.9	64.5	55.0	75.0
If it is known that a person behaves aggressively, the financial counsellor should ask a support worker or family member to attend the meeting with them.^	90.0	70.6	77.4	80.0	88.0
If the financial counsellor has listened non-judgmentally and the person remains in their negative emotional state they should offer the following:					
• Relevant websites, e.g. beyondblue.*	83.3	86.0	68.4	72.0	81.5

*Rejected in Round 1; ^Rejected in Round 2
FIS-Financial Institution Staff; FC-Financial Counsellor; MHP-Mental Health Professional; CON-Consumer; CAR-Carer

Category 6a: Communication guidelines for financial counsellors (cont.)

Item	FIS	FC	MHP	CON	CAR
When there is serious concern about the person's safety, or in situations where the relevant actions do not seem to be working, the financial counsellor should:					
• Request the police to check on them.^	85.0	44.1	54.8	45.0	48.0
• Terminate the interview and seek assistance from a supervisor or manager.^	88.1	70.6	83.9	85.0	60.0
When talking over the phone with the person and they are talking about suicide, the financial counsellor should offer to call a suicide call-back service on behalf of the person.#	90.6	82.4	77.4	80.0	87.5
When talking over the phone to the person who is confused or out of touch with reality, the financial counsellor should paraphrase what the person has said. #	79.2	73.5	67.7	84.2	75.0

^Rejected in Round 2; #Rejected in Round 3
FIS-Financial Institution Staff; FC-Financial Counsellor; MHP-Mental Health Professional; CON-Consumer; CAR-Carer

Category 6b: Communication guidelines for all financial institution staff

Item	FIS	FC	MHP	CON	CAR
When meeting the person face to face and the person is unable to communicate effectively (e.g. with mania), the financial institution staff should:					
• Ask the person if they would like to terminate the interview and reschedule.^	90.0	76.5	67.7	95.0	92.0
• Consider asking structured rather than open-ended questions.*	70.0	82.5	73.0	82.6	73.1
• Encourage them to write down their responses to questions.*	62.0	47.5	42.9	45.8	50.0
• Terminate the interview and reschedule the appointment.^	61.7	72.7	61.3	60.0	80.0
• Terminate the interview and reschedule the appointment to a time when a support person or mental health worker can attend with the person.^	80.0	91.2	71.0	80.0	88.0

*Rejected in Round 1; ^Rejected in Round 2
FIS-Financial Institution Staff; FC-Financial Counsellor; MHP-Mental Health Professional; CON-Consumer; CAR-Carer

Category 6b: Communication guidelines for all financial institution staff (cont.)

Item	FIS	FC	MHP	CON	CAR
When meeting the person face to face and the person is presenting aggressively, the financial institution staff should:					
• Consider inviting the person to sit down if they are standing.^	73.3	67.6	83.9	90.0	76.0
• Terminate the interview and reschedule the appointment.^	65.0	57.6	56.7	70.0	56.0
• Take a deep breath to calm and ground themselves.^	76.7	64.7	87.1	95.0	80.0
• State that the aggression is unacceptable behaviour.^	60.0	38.2	41.9	75.0	44.0
• Validate the person's feeling of frustration and anger.^	68.3	79.4	96.8	90.0	68.0
• Encourage them to get professional help, if appropriate.^	83.3	73.5	67.7	50.0	60.0
• Offer to see the person privately.^	31.7	15.2	29.0	45.0	24.0

^Rejected in Round 2
FIS-Financial Institution Staff; FC-Financial Counsellor; MHP-Mental Health Professional; CON-Consumer; CAR-Carer

Category 6b: Communication guidelines for all financial institution staff (cont.)

Item	FIS	FC	MHP	CON	CAR
If the financial institution staff has listened non-judgmentally and the person remains in their negative emotional state they should offer the following:					
• Information about local mental health services (verbally or in an email, or via the post).*	64.9	69.8	70.3	68.0	66.7
• Referral to a telephone counselling service, e.g. Lifeline.*	85.9	88.4	75.7	72.0	66.7
• Relevant websites, e.g. *beyondblue*.*	68.4	69.8	59.5	62.5	63.0
The financial institution staff should do the following when talking over the phone with the person:					
• Offer relevant resources, e.g. contact details for Lifeline, local mental health service.*	86.3	90.7	83.8	80.0	72.0
When there is serious concern about the person's safety, or in situations where the relevant actions do not seem to be working, financial institution staff should:					
• Contact their local mental health service.*	58.4	69.8	83.3	72.0	80.8
• Request the police to check on them.^	86.7	44.1	58.1	40.0	48.0
• Terminate the interview and seek assistance from a supervisor or manager.^	91.5	85.3	87.1	85.0	68.0

*Rejected in Round 1; ^Rejected in Round 2
FIS-Financial Institution Staff; FC-Financial Counsellor; MHP-Mental Health Professional; CON-Consumer; CAR-Carer

Category 6b: Communication guidelines for all financial institution staff (cont.)

Item	FIS	FC	MHP	CON	CAR
If possible, financial institution staff should request that the person who may be confused or out of touch with reality finds a spot to talk that is quiet and free of distractions.*	68.8	81.0	76.3	84.0	88.5
If possible, financial institution staff should finds a spot to talk that is quiet and free of background noise when they are talking to the person that may be confused or out of touch with reality.*	72.4	88.4	78.9	88.0	92.6
The financial institution collection staff should be aware that the person's confusion and fear about what is happening to them may lead them to deny that anything is wrong with them.#	79.2	100.0	96.8	100.0	100.0
The financial institution collection staff should be aware that the person who is confused or out of touch with reality may be frightened by their thoughts and feelings.#	71.7	100.0	93.5	100.0	100.0
When the person who may be confused or out of touch with reality and is denying that anything is wrong, financial institution staff should encourage them to talk to someone they trust.*	69.2	93.0	100.0	80.0	88.9
The financial institution branch staff should be aware that the person who is confused or out of touch with reality may be frightened by their thoughts and feelings.#	75.5	100.0	96.8	100.0	100.0

*Rejected in Round 1; #Rejected in Round 3
FIS-Financial Institution Staff; FC-Financial Counsellor; MHP-Mental Health Professional; CON-Consumer; CAR-Carer

Category 6b: Communication guidelines for all financial institution staff (cont.)

Item	FIS	FC	MHP	CON	CAR
If the person is becoming aggressive over the phone, the financial institution staff should involve a more senior staff member who is trained in mental health issues. #	66.0	94.1	83.9	94.7	100.0

#Rejected in Round 3
FIS-Financial Institution Staff; FC-Financial Counsellor; MHP-Mental Health Professional; CON-Consumer; CAR-Carer

Category 7: Mental Health and Financial Difficulties Impact Form

Item	FIS	FC	MHP	CON	CAR
The following information should be included in a Debt and Mental Illness Evidence Form:					
• Details about the mental health problems of the person.*	87.5	72.5	59.5	44.0	65.4
• Details of the treatment/support the person is receiving with regards to their mental health problems.*	61.3	61.9	56.8	40.0	66.7
• Details of when the treatment started and whether it is ongoing.*	77.5	59.5	45.9	36.0	38.5
• Details about when the person's mental health problems started.*	67.1	50.0	33.3	28.0	30.8

*Rejected in Round 1
FIS-Financial Institution Staff; FC-Financial Counsellor; MHP-Mental Health Professional; CON-Consumer; CAR-Carer

Category 7: Mental Health and Financial Difficulties Impact Form (cont.)

Item	FIS	FC	MHP	CON	CAR
• Details about whether their mental health problems are ongoing.*	92.5	76.2	64.9	48.0	70.4
• Details of how long the condition is likely to last.*	84.8	69.0	51.4	41.7	59.3
• Option for authorising financial institutions to contact the mental health professional if they are unable to contact the person or their authorised party representative.*	68.4	69.0	80.6	80.0	74.1
• An option for the mental health professional to provide any additional relevant information.^	88.3	94.1	80.6	90.0	72.0
• Information about how any treatment (e.g. medication, ECT) may affect the person's comprehension.^	88.3	85.3	74.2	90.0	64.0
The financial counsellor should ask all clients with mental health problems to obtain this completed form from their mental health professional.*	65.0	40.5	28.9	36.0	30.8
The financial counsellor should ask the person with mental health problems to obtain this completed form from their mental health professional, when proof of condition is required to obtain a particular outcome from the financial institution.*	92.5	76.2	78.9	84.0	81.5

*Rejected in Round 1; ^Rejected in Round 2
FIS-Financial Institution Staff; FC-Financial Counsellor; MHP-Mental Health Professional; CON-Consumer; CAR-Carer

Category 7: Mental Health and Financial Difficulties Impact Form (cont.)

Item	FIS	FC	MHP	CON	CAR
The mental health professional should always complete this form when first consulting with a person with financial difficulties.*	59.5	35.7	23.7	32.0	46.2
The mental health professional should complete this form when the information is requested by a financial institution.*	79.5	60.0	39.5	64.0	46.2
The mental health professional should give this completed form to the person to pass on to the financial counsellor.*	3.0	3.0	37.8	60.0	32.0
The mental health professional should give this completed form to the person to pass on to the financial institution.*	55.1	42.9	27.0	36.0	19.2
The mental health professional should complete this form when the information is requested by a financial counsellor.*	81.0	80.0	50.0	75.0	56.0
The mental health professional should give this completed form directly to the financial counsellor, with the person's permission.*	83.8	71.8	63.2	84.0	84.0
The mental health professional should give this completed form directly to the financial institution, with the person’s permission.*	75.9	62.5	45.9	72.0	73.1

*Rejected in Round 1
FIS-Financial Institution Staff; FC-Financial Counsellor; MHP-Mental Health Professional; CON-Consumer; CAR-Carer

Category 7: Mental Health and Financial Difficulties Impact Form (cont.)

Item	FIS	FC	MHP	CON	CAR
The mental health professional should review this form annually and resubmit it if needed.*	60.5	44.4	44.7	56.0	57.7
The mental health professional should review this form every 6 months after initial completion and resubmit it if needed.*	65.4	42.9	36.8	56.0	53.8
The mental health professional should review this form when the person's mental health problems change and resubmit it if needed.*	87.3	75.6	73.0	84.0	77.8
The financial counsellor should ask the person with mental health problems to obtain this completed form from their mental health professional, when proof of condition is required to obtain a particular outcome from the financial institution.^	92.5	76.2	78.9	84.0	81.5
If there is a cost associated with the mental health professional completing the form, this should be paid by:					
• The financial counsellor*	10.8	2.4	15.8	25.0	32.0
• The financial institution*	10.7	28.6	42.1	41.7	45.8
• The mental health professional (i.e. waive cost)*	66.7	61.9	27.0	48.0	66.7
• The person*	28.6	7.1	5.3	12.5	20.0

*Rejected in Round 1; ^Rejected in Round 2
FIS-Financial Institution Staff; FC-Financial Counsellor; MHP-Mental Health Professional; CON-Consumer; CAR-Carer

Category 8a: Policies and procedures for financial institutions

Item	FIS	FC	MHP	CON	CAR
The tailored procedure (for people with mental health problems and financial difficulties) should include the following options:					
• Longer time lines for repayment or settlement of debt.*	70.5	97.7	91.9	96.0	100.0
• Longer time line to vacate property.*	67.5	88.4	89.2	96.0	100.0
• Not selling debt to third parties.*	38.5	78.6	89.2	88.0	96.3
• Suspending interest payments when the person is acutely unwell or in a mental health crisis.*	57.1	97.7	97.3	96.0	100.0
The financial institution should have specific policies around credit listing for people with mental health problems.*	61.8	83.7	78.4	83.3	77.8
Financial institutions should make their policy on working with people with mental health problems and financial difficulties publically available.*	72.4	90.5	86.8	96.0	92.3
Financial institutions should have a mental health advisory committee to review relevant information for customers (e.g. financial education, hardship literature) to ensure it is appropriate and non-stigmatising.*	69.2	97.6	86.8	96.0	100.0

*Rejected in Round 1
FIS-Financial Institution Staff; FC-Financial Counsellor; MHP-Mental Health Professional; CON-Consumer; CAR-Carer

Category 8a: Policies and procedures for financial institutions (cont.)

Item	FIS	FC	MHP	CON	CAR
The financial institution staff should be proactive in assisting people with mental health problems before they experience financial difficulties, e.g. providing tips and tools for financial management, and financial counselling contact information.^	70.0	88.2	83.9	75.0	96.0

^Rejected in Round 2
FIS-Financial Institution Staff; FC-Financial Counsellor; MHP-Mental Health Professional; CON-Consumer; CAR-Carer

Category 8b: Policies and procedures for mental health services

Item	FIS	FC	MHP	CON	CAR
Mental health professionals should be required to have financial literacy skills training as part of their continuing professional development.*	57.7	67.4	63.2	68.0	81.5
The mental health professional should be trained in the following basic skills to assist the person to build financial stability:					
• Assisting the person to create a basic budget.*	57.5	57.1	78.9	76.0	85.2
• Educating the person on typical banking procedures.*					

*Rejected in Round 1
FIS-Financial Institution Staff; FC-Financial Counsellor; MHP-Mental Health Professional; CON-Consumer; CAR-Carer

Category 8b: Policies and procedures for mental health services (cont.)

Item	FIS	FC	MHP	CON	CAR
The mental health professional should be trained in the following basic skills to assist the person to build financial stability:					
• Basic financial literacy skills.*	66.7	76.7	65.8	80.0	85.2
• Assisting the person to identify financial practices that benefit or add to financial stability, e.g. saving, budgeting, comparison shopping.*	70.9	82.9	89.5	88.0	96.3
• Meeting regularly to review person's financial plans/goals/status.*	57.0	52.4	65.8	84.0	85.2

*Rejected in Round 1
FIS-Financial Institution Staff; FC-Financial Counsellor; MHP-Mental Health Professional; CON-Consumer; CAR-Carer
