# Supplementary material for: Assisting Australians with mental health problems and financial difficulties: a Delphi study to develop guidelines for financial counsellors, financial institution staff, mental health professionals and carers
Source: BMC Health Serv Res. 2015 Jun 3;15:218. doi: 10.1186/s12913-015-0868-2 (PMC4453216; doi:10.1186/s12913-015-0868-2)
Supplement: Additional file 6: — Guidelines for professionals who work with people with mental health problems and financial difficulties: Final copy (prior to graphic design stage) of the guidelines for financial counsellors, financial institution staff, mental health professionals and carers. [file 12913_2015_868_MOESM6_ESM.pdf]

Guidelines for professionals who work with people with mental health problems and financial difficulties

Table of Contents

1 Introduction to the Guidelines for Professionals Who Work with People with Mental Health Problems and Financial Difficulties 4
Purpose of the guidelines 4
Development of the guidelines 4
How to use this document 4

2 Guidelines for Financial Counsellors 6
Knowledge of mental health problems 6
The links between mental health problems and financial difficulties 6
The impact of mental health problems on financial difficulties 7
The impact of financial difficulties on mental health 7
The impact of stigma 8
Support of clients with mental health problems 8
Resources 8
Relevant laws and professional codes of conduct 9
Working with mental health professionals 9
Disclosing mental health problems to the financial institution 9
Effective communication 10
General communication skills 10
Additional considerations for when the client has memory and concentration problems 12
Additional considerations when the client is distressed 12
Additional considerations when the client is confused or out of touch with reality 12
Additional considerations when the client is behaving aggressively 13
Additional considerations when there is a concern about safety 13
Policies and procedures for financial counselling services 14

3 Guidelines for Financial Institution Staff 15

Guidelines for Hardship Staff 15
Knowledge of mental health problems 15
The impact of stigma 15
The links between mental health problems and financial difficulties 16
The impact of mental health problems on financial difficulties 16
The impact of financial difficulties on mental health 16
Support for customers with mental health problems 17
Relevant Laws 17
Working with other professionals 17

Draft. Not for distribution.

Guidelines for Collection Staff 18
Knowledge of mental health problems 18
The impact of stigma 18
The links between mental health problems and financial difficulties 18
The impact of mental health on financial difficulties 19
The impact of financial difficulties on mental health 19
Support for customers with mental health problems 19
Relevant Laws 20
Working with other professionals 20

Guidelines for Branch Staff 20
Knowledge of mental health problems 20
The impact of stigma 21
The links between mental health problems and financial difficulties 21
The impact of mental health on financial difficulties 21
The impact of financial difficulties on mental health 22
Support for customers with mental health problems 22
Relevant laws 22
Cultural considerations 22
Working with other professionals 22

Guidelines for Contact Centre Staff 23
Knowledge of mental health problems 23
The links between mental health problems and financial difficulties 23
The impact of mental health on financial difficulties 23
The impact of financial difficulties on mental health 24
Support for customers with mental health problems 24
Relevant laws 24
Working with other professionals 25

Communication Guidelines for Financial Institution Staff 25
General communication skills 25
Additional considerations for when the customer has memory and concentration problems 27
Addition considerations when the customer is distressed 27
Additional considerations when the customer is confused or out of touch with reality 27
Additional considerations when the customer is behaving aggressively 28
Additional considerations when there is a concern about safety 28
Policies and Procedures for Financial Institutions 29
Mental health training for staff 29
Mental health information for customers 30

4 Guidelines for Mental Health Professionals 31
The links between mental health problems and financial difficulties 31
The impact of mental health problems on financial difficulties 31
The impact of financial difficulties on mental health 31
Supporting clients with financial difficulties 32
Resources 33
Relevant laws and professional codes of conduct 33
Working with financial counsellors 33
Training for mental health professionals 33

Draft. Not for distribution.

5 Guidelines for the Support Person 35
The links between mental health problems and financial difficulties 35
The impact of mental health on financial difficulties 35
The impact of financial difficulties on mental health 36
Supporting the person 36
6 Mental Health and Financial Difficulties Impact Form 38

Draft. Not for distribution.

1 Introduction to the Guidelines for Professionals Who Work with People with Mental Health Problems and Financial Difficulties

Purpose of the guidelines

Research shows a strong association between mental health problems and financial difficulties. These guidelines have been developed to guide the best way to work with and support a person who is experiencing mental health problems and financial difficulties. They are for the use of financial counsellors, financial institution staff, mental health professionals, and family and other support people. These guidelines are a general set of recommendations and may not be appropriate for every person who is experiencing mental health problems and financial difficulties. It is important that the professional or support person tailor their approach to the individual and their situation.

Development of the guidelines

These guidelines were produced using the Delphi method, which is a systematic way of assessing the consensus of a panel of experts. A wide range of potential actions were derived from a review of the research and best practice literature. The actions included in the guidelines have been rated as important or essential by expert panels of financial counsellors, financial institution staff, mental health professionals, mental health consumers and carers with experience of supporting a person with mental health problems and financial difficulties.

Details of the methodology can be found in Bond, K, et al., (in submission) *Assisting the person with mental health problems and financial difficulties: A Delphi expert consensus study to develop guidelines for financial counsellors, financial institution staff, mental health professionals and carers.*

Although these guidelines are copyright, they can be freely reproduced for non-profit purposes provided the source is acknowledged. Please cite these guidelines as follows:

Mental Health First Aid Australia. *Guidelines for professionals who work with people with mental health problems and financial difficulties*. Mental Health First Aid Australia: Melbourne; 2014.

This initiative was funded by the Australian Government Department of Health, and Ageing, and Australia and New Zealand Banking Group Ltd (ANZ), GE Capital Finance Australasia Pty Ltd, National Australia Bank Ltd (NAB), Westpac Banking Corporation and *beyondblue*.

How to use this document

The chapters in this document are set out to allow the specific roles (e.g. financial counsellor, hardship staff) to look up and read the guidelines specific to

Draft. Not for distribution.

them, without having to read all of the guidelines. It is set out in the following way:

- **Chapter two** outlines what the **financial counsellor** needs to know and do when working with a client with mental health problems. It also contains communication guidelines when speaking to a client with mental health problems, both in person and over the phone. It concludes with outlining the policies and procedures, which financial counselling services should develop regarding working with clients with mental health problems.
- **Chapter three** is divided into guidelines for the specific **financial institution staff**, specifically hardship, collection, branch and contact centre staff. It contains information about what the various financial institution staff need to know and do when working and communicating with a customer experiencing mental health problems and financial difficulties. It concludes with a section addressing financial institution policies and procedures related to working with people with mental health problems and financial difficulties.
- **Chapter four** presents what the **mental health professional** needs to know and do when working with clients with financial difficulties.
- **Chapter five** outlines what the **support person** needs to know and do when supporting someone with mental health problems and financial difficulties.
- **Chapter six** presents the guidelines for the development and use of the *Mental Health and Financial Difficulties Impact Form*.

Draft. Not for distribution.

2 Guidelines for Financial Counsellors

This section presents the guidelines for financial counsellors. The first six sections address what the financial counsellor needs to know and do when working with clients with mental health problems. The next section presents guidelines for communicating with clients with mental health problems and the final section presents guidelines for financial counselling services with regards to policies and procedures.

Knowledge of mental health problems

The financial counsellor should be aware of:

- What a mental illness is
- Types of mental illnesses
- How common mental illnesses are
- Signs and symptoms of mental illness, including behaviours that may indicate that a client has mental health problems, e.g. not attending appointments, not returning phone calls, not meeting repayment schedules, displaying unusual behaviours, lack of clarity of thought
- Causes of and risk factors for mental illnesses
- Warning signs and risk factors for suicide.

The financial counsellor should be aware that mental health problems may take weeks or months to develop, or can develop or worsen rapidly. They should also know that the impact of mental health problems will differ from one client to another, and may vary over time.

The financial counsellor should be aware of the importance of early identification of and intervention for mental health problems, because this can limit the impact of mental health problems. The financial counsellor should be familiar with the following mental health first aid guidelines (free download at www.mhfa.com):

- Depression
- Psychosis
- Panic attacks.

Box

Definitions of mental illness and mental health problems

'Mental illness' is a diagnosable illness that affects a person's thinking, emotional state and behaviour, and disrupts the person's ability to work or carry out other daily activities and engage in satisfying personal relationships.

‘Mental health problems’ is a broader term including mental illnesses, symptoms of mental illnesses that may not be severe enough to warrant the diagnosis of a mental illness, as well as mental health-related crises such as having thoughts of suicide.

The links between mental health problems and financial difficulties

The financial counsellor should be aware that mental health problems can contribute to financial difficulties, and vice versa, e.g. failing to pay bills because

Draft. Not for distribution.

of depression or experiencing suicidal thoughts due to financial difficulties. The financial counsellor should be aware that having mental health problems is not necessarily an indicator of an inability to manage money.

The financial counsellor should know that the cycle of financial difficulties and mental health problems can be intensified for people who have difficulty navigating unfamiliar financial and health systems. This may include people from Aboriginal, Torres Strait Islander, and culturally and linguistically diverse heritage. The financial counsellor should be familiar with the cultural considerations when working with these groups of people (go to www.mhfa.com.au for a copy the guidelines for providing mental health first aid to an Aboriginal and Torres Strait Islander person.)

The impact of mental health problems on financial difficulties
The financial counsellor should know how the symptoms of mental health problems may impact on financial difficulties. For instance, a client may not complete financial tasks because they forget, lack the motivation to pay bills, make poor financial decisions, or feel overwhelmed by financial tasks, such as communicating with creditors, opening mail, or visiting Centrelink. The financial counsellor should be aware that financial stability may not be an immediate goal for some clients in the early stages of recovery from mental illness. The financial counsellor should also know that overspending and financial difficulties can be a sign of relapse of the client's mental health problems. There are also some specific sources of financial difficulties faced by people with mental health problems (see box).

Box
Specific sources of financial difficulties for people with mental health problems may include:

- Debt incurred to support a drug or alcohol problem
- Increased medical expenses
- Problem gambling associated with bipolar disorder
- Overspending when in a manic or depressed state
- Job instability associated with episodic mental illness
- Poor money management due to paranoid thoughts, e.g. withdrawing money from the bank to keep it 'safe'.

The impact of financial difficulties on mental health
The financial counsellor should know that financial difficulties can have a negative impact on mental health and that addressing financial problems early can reduce this impact. It is also important to know that the way the client perceives their financial situation is what impacts on their mental health, rather than their actual situation. Even a client who is making their repayments may still be experiencing financial difficulties and distress.

Financial difficulties may also impact on mental health of the person in a number of less obvious ways, including:

- Isolation and lack of support due to the inability to pay for participation in social activities

Draft. Not for distribution.

- Worsening of mental health problems due to an inability to pay for treatment
- Stress caused by the processes that institutions use to recover debt.

The financial counsellor should be aware that when a client sees no way back from their financial difficulties they might become suicidal or attempt suicide. The financial counsellor should be familiar with the mental health first aid guidelines for helping a person who is experiencing suicidal thoughts and behaviours (free download at www.mhfa.com).

The impact of stigma

People with mental health problems can be intelligent, capable people going through a difficult time, like many other people in the community. Unfortunately the myths surrounding mental health problems can lead to stigma which can have a negative impact on people with mental health problems, including limiting their earning capacity. Even people with mental health problems can hold stigmatising attitudes about themselves that can have a negative impact, including on how they deal with financial difficulties. It is important that the financial counsellor be aware of any stigmatising attitudes that they themselves may hold and understand how these may affect their clients with mental health problems.

Support of clients with mental health problems

The financial counsellor should know how they can support their clients in a way that promotes recovery of mental health. They should also know how to reduce stressors that may exacerbate current mental health problems or that may contribute to relapse. The financial counsellor should be aware that their clients with mental health problems are likely to need fluctuating levels of support, as the symptoms of most mental health problems come and go over time.

The financial counsellor should adopt a flexible approach to appointments when working with the client with mental health problems, e.g. extra time for the appointment, shorter and more frequent appointments, increasing the number of sessions.

When working with the person to develop financial strategies, the financial counsellor should consider the following:

- The person's level of understanding
- The person's capacity for decision-making
- The person's ability to engage meaningfully with financial institutions
- The person's ability to implement realistic repayment plans
- The long-term impact of the mental illness.

The financial counsellor should also be aware that the client's financial decisions may be influenced by their support person or others.

Resources

The financial counsellor should be aware of the following resources and encourage their client to use them:

Draft. Not for distribution.

- Community mental health services
- Disability support services
- Centrelink benefits for people with mental health problems.

Relevant laws and professional codes of conduct
The financial counsellor should have a thorough working knowledge of relevant laws and professional codes of conduct related to:
- The appointment of a third party to manage a client's finances, e.g. financial administration order
- The interactions between a client with mental health problems and creditors
- Their responsibility in responding to a client who is experiencing a mental health crisis.

Working with mental health professionals
If the client discloses mental health problems and asks for help with these, the financial counsellor should provide the client with written material about options for professional help and refer them, as appropriate.

With the client's consent, the financial counsellor and the mental health professional should work together. They should:
- Deliver consistent messages to the client
- Ensure that the financial counsellor's client service plan complements the mental health treatment plan
- Be aware of and discuss any conflicting priorities between the financial and treatment goals
- Determine whether the person is able to make specific decisions concerning their current financial situation
- Work with the client and support person to identify the specific supports needed to help the client make financial decisions
- Use the *Mental Health and Financial Difficulties Impact Form* to communicate information about how the client's mental health problems impact on their financial difficulties (see the section entitled Mental Health and Financial Difficulties Impact Form on page?).

Disclosing mental health problems to the financial institution
The financial counsellor should be aware that people with mental health problems may be reluctant to tell financial institutions about their condition, e.g. because of shame, fear of being refused credit or because they have other worries about how the information will be used.

However, most financial institutions have policies about assistance for people who are experiencing financial difficulties. The financial counsellor should know about these policies and the positive role that financial institutions can play for people with mental health problems and financial difficulties.

If the client wishes to disclose mental health problems to a financial institution, the financial counsellor should:

Draft. Not for distribution.

- Encourage the client to decide what they hope to achieve by disclosing and check if this is realistic
- Advise the client to ask to speak to a specialist team member at the financial institution who is experienced in working with people with mental health problems and financial difficulties
- Inform the client that they may be asked to provide evidence of their mental health problems in order to reach the most suitable solution to the financial difficulties
- Encourage the use of the *Mental Health and Financial Difficulties Impact Form* to communicate information on the impact of the client's mental health problems on their financial difficulties
- Inform the client that, if they do disclose their mental health problems, the financial institution staff may not necessarily understand the impact of mental health problems on financial difficulties
- Encourage the client to keep records of who they have spoken to, what was said and when.

Effective communication

The guidelines in this section are about communicating with a client with mental health problems and financial difficulties, both over the phone and when meeting with the person face-to-face. In cases where it is only applicable to phone or face-to-face communication this will be indicated.

Personal attitudes, beliefs and judgements influence communication, therefore it is important for financial counsellors to be aware of their own attitudes about mental health problems and how these might affect their ability to respond to a client non-judgmentally. They should endeavour to set aside any negative beliefs and avoid expressing any negative reactions and judgements, in order to focus on the needs of the client they are assisting.

General communication skills

The financial counsellor should know how to communicate effectively with clients with mental health problems. The financial counsellor should take time to listen to their clients with mental health problems, allowing them enough time to think and talk, by:

- Letting the client set the pace and style of the conversation, as far as possible
- Listening non-judgmentally so that the client can feel like they have been heard and understood
- Showing empathy, by using statements such as, "this must be very difficult for you", and convey acceptance to the client
- Conveying genuineness to the client, i.e. the financial counsellor's body language and tone of voice match what they are saying
- Respecting the client's feelings and opinions, even if the financial counsellor does not agree with them.

The financial counsellor should use the following communication skills:

- Ask questions that indicate care, e.g. clarifying questions to show that they understand what the client is saying

Draft. Not for distribution.

- Check their understanding by re-stating what the client has said and summarise facts and feelings
- Listen not only to what the client says, but how they say it, i.e. their tone of voice
- Use prompts, such as “I see” and “Ah”, when necessary to show the client that they are listening
- Be patient, even when the client may not be communicating well, may be repetitive or may be speaking slowly and unclearly
- Allow pauses and silences to give the client time to think or gather their thoughts.

To facilitate communication, the financial counsellor should avoid:

- Attempting to say something positive which may be perceived as trivialising the client’s experiences, such as “put a smile on your face” or “lighten up”
- Attempting to say something supportive which may be perceived as belittling or dismissing the client's feelings, such as "you don't seem that bad to me", “get your act together”
- Speaking in a tone of voice which may be perceived as patronising
- Labelling the client’s feelings for them
- Pressuring the client to talk about their feelings
- Being critical or expressing frustration at the client for being in a negative emotional state
- Interrupting the client when they are speaking, especially to give their opinion
- Using a hostile or sarcastic tone when the client’s responses are not what they expect
- Confrontation, unless it is necessary to prevent harmful or dangerous acts.

If the client is unable to communicate effectively, the financial counsellor should discourage the client from making any financial decisions until they are able to communicate effectively. If speaking over the phone, the financial counsellor should offer to call them back at a better time.

They should be familiar with and listen for signs that may indicate that the client is:

- Having memory or concentration problems
- Distressed
- Confused or out of touch with reality
- Aggressive
- Behaving in a way that leads to concern for safety.

If the client is displaying one or more of the above signs, the financial counsellor should offer relevant information to the client, e.g. contact details for Lifeline or the local mental health service. If the financial counsellor has listened non-judgmentally and the client remains in a negative emotional state they should offer to contact a support person or the client’s mental health worker.

Draft. Not for distribution.

Additional considerations for when the client has memory and concentration problems
The financial counsellor should be aware that clients with mental health problems may have memory and concentration problems. Where this is the case, giving simple and concise information about their situation and repeating information can be helpful. If needed, the financial counsellor should provide the client with a written plain language summary of the information and options discussed during the appointment. If appropriate, they should also encourage the client to record important information in a diary.

Additional considerations when the client is distressed
The financial counselling services should have a list of statements that their staff can paraphrase, using their own words, to help guide telephone conversations when talking to the client who appears to be distressed, e.g. "It is understandable that you are anxious at a time like this given everything that is happening for you at the moment" or "Lots of people say that going through financial difficulties can be very stressful", or "It can be important to talk to someone about what is happening. Is there someone who can give you that support?"

Additional considerations when the client is confused or out of touch with reality
When the financial counsellor is talking over the phone with a client who is confused or out of touch with reality, they should ask the client to find a spot that is quiet and free from distractions. The financial counsellor should also try to find a spot that is quiet and free of background noise.

The financial counsellor should tailor their interaction to the way the client is behaving. If the client is confused, the financial counsellor should respond to the client by communicating in a simple and concise manner. It is important to be patient and allow time for the client to process the information and respond to what has been said, repeating things if necessary.

If the client is out of touch with reality, be sensitive to this and reassure them you are there to assist. The financial counsellor should be aware that delusions and hallucinations are very real to the client. While not agreeing with the client's delusions and hallucinations, the financial counsellor can acknowledge how the client may be feeling, e.g. "that must be difficult for you" or "I can see that you are upset".

So as not to worsen the situation, the financial counsellor should not:
- Assume the client cannot understand what is being said
- Raise their voice as if speaking to someone who is hard of hearing
- Dismiss, minimise or argue with the client about their confusion, delusions or hallucinations
- Act alarmed, horrified or embarrassed by the client's confusion, delusions or hallucinations
- Laugh at the client's confusion, delusions or hallucinations
- Encourage or inflame any paranoia.

Draft. Not for distribution.

When the financial counsellor is working with a client who is confused or out of touch with reality, they should be aware that the client may be frightened by their thoughts and feelings and this fear may lead them to deny that anything is wrong. If this happens, the financial counsellor should encourage the client to talk to someone they trust.

Additional considerations when the client is behaving aggressively

If a client is behaving aggressively it is important not to take the aggressive behaviour personally and understand that they may be feeling confused or frightened. Be aware that their symptoms or fear causing their aggression might be exacerbated if certain steps are taken, e.g. involving the police. The following strategies can be helpful when working with an aggressive client:

- Remain as calm as possible and avoid nervous behaviour, e.g. shuffling your feet, fidgeting, making abrupt movements
- Use positive words, such as 'stay calm', rather than negative words, such as 'stop shouting'
- Speak to the client slowly and confidently with a gentle, caring tone of voice
- Explain to the client that although you would like to help them, it is not possible when they are (state the unacceptable behaviour, e.g. yelling)
- Consider taking a break from the conversation to allow the client a chance to calm down.

So as not to increase fear or aggressive behaviour, the financial counsellor should avoid:

- Raising their voice or talking too fast
- Responding in a hostile, disciplinary or challenging manner
- Arguing with the client
- Threatening them
- Restricting the client's movement (e.g. if the meeting is face to face and he or she wants to pace up and down the room).

After an encounter with a client who is behaving aggressively the financial counsellor should take a break and discuss it with a supervisor.

Additional considerations when there is a concern about safety

If the financial counselor is concerned about the safety of the client, themselves or others, they should:

- Take any threats or warnings seriously, particularly if the client believes they are being persecuted
- Explain that this cannot be kept confidential
- Consider terminating the interview
- Seek assistance from a supervisor
- Contact the local mental health service and the authorised third party, if there is one, to discuss their concerns about the client
- Take any necessary protective action, e.g. contact the police or mental health crisis team.

Draft. Not for distribution.

If the contact is by phone, the financial counsellor should also try to ascertain the client's whereabouts.

Box
Self-care resources for the financial counsellor
Working with people with mental health problems and financial difficulties can be stressful. For this reason, if the financial counsellor is having difficulty coping, they should make use of any support services provided by their employer or available in the community.

Policies and procedures for financial counselling services

Financial counselling services should have policies and procedures that are adapted to support clients with mental health problems. These policies and procedures should cover the training of financial counsellors and information resources for clients.

Training of financial counsellors should include:

- Basic mental health and counselling skills, which are informed by contemporary thinking and best practice in the mental health sector
- Continuing professional development that includes mental health training
- Hearing about the lived experience of people who have had mental health problems and financial difficulties, as well as learning about the impact of mental health problems and financial difficulties on the family of the client
- Use of any documents or forms relevant to working with people with mental health problems, e.g. *Mental Health and Financial Difficulties Impact Form*, an advance directive.

Information for clients should include external resources and community services relevant to people with mental health problems. This should be regularly updated. Any website for people with financial difficulties should have a link to information which assists people in a mental health crisis, e.g. Lifeline, *beyondblue*.

Draft. Not for distribution.

3 Guidelines for Financial Institution Staff

This section presents the guidelines for financial institution staff. It first presents the guidelines for the individual financial institution roles (i.e. hardship, collection, branch and contact centre). The following section presents guidelines for communicating with clients with mental health problems and financial difficulties; this section applies to all of the financial institution staff roles. The final section presents guidelines for financial institutions with regards to policies and procedures.

Not for distribution.

Guidelines for Hardship Staff

Knowledge of mental health problems

Hardship staff should be aware of what a mental illness is, how common mental illnesses are, and the signs and symptoms of mental illnesses. They should also be aware of the risk factors for suicide. Hardship staff should be able to recognise the behaviours that may indicate that their customer has mental health problems, e.g. not returning phone calls, not meeting repayment schedules, displaying unusual behaviours, lack of clarity of thought.

Hardship staff should be aware that mental health problems may take weeks or months to develop, or can develop or worsen rapidly. They should also know that the impact of mental health problems will differ from one person to another, and may vary over time.

Box

Definitions of mental illness and mental health problems

'Mental illness' is a diagnosable illness that affects a person's thinking, emotional state and behaviour, and disrupts the person's ability to work or carry out other daily activities and engage in satisfying personal relationships.

'Mental health problems' is a broader term including mental illnesses, symptoms of mental illnesses that may not be severe enough to warrant the diagnosis of a mental illness, as well as mental health-related crises such as having thoughts of suicide.

The impact of stigma

People with mental health problems can be intelligent, capable people going through a difficult time, like many other people in the community. Stigmatising attitudes of others can have a negative impact on people with mental health problems, e.g. limiting their earning capacity. Even people with mental health problems can hold these attitudes about themselves, which can have a negative impact, including on how they deal with financial difficulties. It is important that hardship staff be aware that they themselves may hold stigmatising attitudes

and understand how these may affect their customer with mental health problems.

The links between mental health problems and financial difficulties

Hardship staff should be aware that mental health problems can contribute to financial difficulties, and vice versa, e.g. failing to pay bills because of depression or experiencing suicidal thoughts due to financial difficulties. Like many people, those with mental health problems may have limited financial literacy skills. However, hardship staff should be aware that having mental health problems is not necessarily an indicator of an inability to manage money.

Hardship staff should know that the cycle of financial difficulties and mental health problems can be intensified for people from culturally and linguistically diverse heritage, due to the difficulty of navigating unfamiliar financial and health systems in Australia.

The impact of mental health problems on financial difficulties

Hardship staff should know how the symptoms of mental health problems may impact on financial difficulty. For instance, a customer may not complete financial tasks because they forget, lack the motivation to pay bills, make poor financial decisions, or feel overwhelmed by financial tasks, such as communicating with creditors, opening mail, or visiting Centrelink. Hardship staff should be aware that financial stability may not be an immediate goal for some customers in the early stages of recovery from mental illness. Hardship staff should also know that overspending and financial difficulties can be a sign of relapse of the customer's mental health problems. There are also some specific sources of financial difficulties faced by people with mental health problems (see box).

Box

Specific sources of financial difficulties for people with mental health problems may include:

- Debt incurred to support a drug or alcohol problem
- Increased medical expenses
- Problem gambling associated with bipolar disorder
- Overspending when in a manic or depressed state
- Job instability associated with episodic mental illness
- Poor money management due to paranoid thoughts, e.g. withdrawing money from the bank to keep it 'safe'.

The impact of financial difficulties on mental health

Hardship staff should know that financial difficulties can have a negative impact on mental health and that addressing financial problems early can reduce this impact. It is also important to know that the way the customer perceives their financial situation is what impacts on their mental health, rather than their actual situation. Even a customer who is making their repayments may still be experiencing financial difficulties and distress, e.g. they may not be meeting their other financial obligations.

Draft. Not for distribution.

Financial difficulties may also impact on mental health in a number of less obvious ways, including:

- Worsening of mental health problems due to an inability to pay for treatment
- Stress caused by the processes that institutions use to recover debt.

Hardship staff should be aware that when a customer sees no way back from their financial difficulties they might become suicidal or attempt suicide.

Support for customers with mental health problems

Hardship staff should know how to reduce stressors that may exacerbate current mental health problems. They should adopt a respectful and positive approach and should consider implementing the following strategies:

- Allowing the customer to choose their preferred method of contact, including via letters, phone or Skype
- Offering to give the customer a plain language summary of information and options discussed during their phone conversation
- Suspending collection activity to give the customer sufficient time to consult a financial counsellor
- Moving from an automated to a tailored collection process.

If a tailored collection process has been put in place, but the customer is not adhering to these arrangements, hardship staff should:

- Encourage authorised third party representation, e.g. a professional, such as a community lawyer or financial counsellor, who can advocate on their behalf
- Ensure any authorised third party representatives are contacted.

Relevant Laws

Hardship staff should have a general understanding of their state and territory laws regarding the appointment of a third party to manage a customer's finances, e.g. guardianship, administration orders, financial management orders. They should be aware of what a financial administration order is and the implications for the customer and the financial institution.

Working with other professionals

Hardship staff should know a few key mental health and disability support services available in the community and update this information regularly. Hardship staff should know that financial counselling services play an important role for people with mental health problems and financial difficulties, including advocating on the behalf of the customer and making appropriate referrals to mental health services. Therefore, hardship staff should offer to refer any customers who have mental health problems, or whom they suspect may have mental health problems, to a financial counsellor.

If the customer is working with a financial counsellor, hardship staff should work with the financial counsellor to determine whether the customer is able to make specific decisions concerning their current financial situation.

Draft. Not for distribution.

If the customer is not working with a financial counsellor, but has a mental health professional, hardship staff should consider working with the professional to determine the customer's capacity. The following issues should be addressed:

- The customer's ability to engage meaningfully with the financial institution
- The customer's capacity for understanding
- The customer's capacity for decision-making.

Guidelines for Collection Staff

Knowledge of mental health problems

Collection staff should be aware of what a mental illness is and how common mental illnesses are in the community. They should also be aware of the risk factors for suicide.

Collection staff should be aware that mental health problems may take weeks or months to develop, or can develop or worsen rapidly. They should also know that the impact of mental health problems will differ from one person to another, and may vary over time.

Box

Definitions of mental illness and mental health problems

'Mental illness' is a diagnosable illness that affects a person's thinking, emotional state and behaviour, and disrupts the person's ability to work or carry out other daily activities and engage in satisfying personal relationships.

'Mental health problems' is a broader term including mental illnesses, symptoms of mental illnesses that may not be severe enough to warrant the diagnosis of a mental illness, as well as mental health-related crises such as having thoughts of suicide.

The impact of stigma

Just like many in the community, people with mental health problems can be intelligent, capable people going through a difficult time. Collection staff should be aware that stigmatising attitudes of others can have a negative impact on people with mental health problems, e.g. limiting their earning capacity.

The links between mental health problems and financial difficulties

Collection staff should be aware that mental health problems can contribute to financial difficulties, and vice versa, e.g. failing to pay bills because of depression or experiencing suicidal thoughts due to financial difficulties. Like many people, those with mental health problems may have limited financial literacy skills. However, collection staff should be aware that having mental health problems is not necessarily an indicator of an inability to manage money.

The impact of mental health on financial difficulties

Collection staff should know how the symptoms of mental health problems may impact on financial difficulties. For instance, a customer may not complete financial tasks because they forget, lack the motivation to pay bills, make poor financial decisions, or feel overwhelmed by financial tasks, such as communicating with creditors, opening mail, or visiting Centrelink. Collection staff should be aware that financial stability may not be an immediate goal for some customers in the early stages of recovery from mental illness. Collection staff should also know that overspending and financial difficulties can be a sign of relapse of the customer's mental health problems. There are also some specific sources of financial difficulties faced by people with mental health problems (see box).

Box

Specific sources of financial difficulties for people with mental health problems may include:

- Debt incurred to support a drug or alcohol problem
- Increased medical expenses
- Problem gambling associated with bipolar disorder
- Overspending when in a manic or depressed state
- Job instability associated with episodic mental illness
- Poor money management due to paranoid thoughts, e.g. withdrawing money from the bank to keep it 'safe'.

The impact of financial difficulties on mental health

Collection staff should know that financial difficulties can have a negative impact on mental health and that addressing financial problems early can reduce this impact. Even a customer who is making their repayments may still be experiencing financial difficulties and distress, e.g. they may not be meeting their other financial obligations.

Financial difficulties may also impact on mental health in a number of less obvious ways, including stress caused by the processes that institutions use to recover debt.

Collection staff should be aware that when a customer sees no way back from their financial difficulties they might become suicidal or attempt suicide.

Support for customers with mental health problems

Collection staff should adopt a respectful and positive approach when working with customers with mental health problems and should consider implementing the following strategies:

- Allowing the customer to choose their preferred method of contact, including via letters, phone or Skype
- Offering to give the customer a plain language summary of information and options discussed during their phone conversation
- Suspending collection activity to give the customer sufficient time to consult a financial counsellor

Draft. Not for distribution.

- Moving from an automated to a tailored collection process.

If a tailored collection process has been put in place, but the customer is not adhering to these arrangements, collection staff should:

- Encourage authorised third party representation, e.g. a professional, such as a community lawyer or financial counsellor, who can advocate on their behalf
- Ensure any authorised third party representatives are contacted.

Relevant Laws
Collection staff should have a general understanding of their state and territory laws regarding the appointment of a third party to manage a customer's finances, e.g. guardianship, administration orders, financial management orders. They should also be aware of what a financial administration order is and the implications for the customer and the financial institution.

Working with other professionals

Collection staff should know that financial counselling services play an important role for people with mental health problems and financial difficulties, including advocating on the behalf of the customer and making appropriate referrals to mental health services. Therefore, collection staff should offer to refer any customers who have disclosed mental health problems to a financial counsellor.

If the customer is not working with a financial counsellor, but has a mental health professional, collection staff should consider working with the professional to determine the customer's capacity. The following issues should be addressed:

- The customer's ability to engage meaningfully with the financial institution
- The customer's capacity for understanding
- The customer's capacity for decision-making.

Draft. Not for distribution.

Guidelines for Branch Staff

Knowledge of mental health problems

Branch staff should be aware of what a mental illness is and how common mental illnesses are in the community. They should also be aware of the risk factors for suicide.

Branch staff should also know that the impact of mental health problems will differ from one person to another, and may vary over time.

Branch staff should be familiar with the following mental health first aid guidelines (go to www.mhfa.com to download the guidelines):

- Depression
- Suicidal thoughts and behaviours

The impact of stigma

Just like many in the community, people with mental health problems can be intelligent, capable people going through a difficult time. Branch staff should be aware that stigmatising attitudes of others can have a negative impact on people with mental health problems, e.g. limiting their earning capacity. It is important that branch staff be aware that they themselves may hold stigmatising attitudes and understand how these may affect their customer with mental health problems.

Box

Definitions of mental illness and mental health problems

'Mental illness' is a diagnosable illness that affects a person's thinking, emotional state and behaviour, and disrupts the person's ability to work or carry out other daily activities and engage in satisfying personal relationships.

'Mental health problems' is a broader term including mental illnesses, symptoms of mental illnesses that may not be severe enough to warrant the diagnosis of a mental illness, as well as mental health-related crises such as having thoughts of suicide.

The links between mental health problems and financial difficulties

Branch staff should be aware that mental health problems can contribute to financial difficulties, and vice versa, e.g. failing to pay bills because of depression or experiencing suicidal thoughts due to financial difficulties. Like many people, those with mental health problems may have limited financial literacy skills. However, branch staff should be aware that having mental health problems is not necessarily an indicator of an inability to manage money.

The impact of mental health on financial difficulties

Branch staff should know how the symptoms of mental health problems may impact on financial difficulties. For instance, a customer may not complete financial tasks because they forget, lack the motivation to pay bills, make poor financial decisions, or feel overwhelmed by financial tasks, such as communicating with creditors, opening mail, or visiting Centrelink. Branch staff should also know that overspending and financial difficulties can be a sign of relapse of the customer's mental health problems. There are also some specific sources of financial difficulties faced by people with mental health problems (see box).

Draft. Not for distri

Box

Specific sources of financial difficulties for customers with mental health problems may include:

- Debt incurred to support a drug or alcohol problem
- Increased medical expenses
- Problem gambling associated with bipolar disorder
- Overspending when in a manic or depressed state
- Job instability associated with episodic mental illness
- Poor money management due to paranoid thoughts, e.g. withdrawing money from the bank to keep it 'safe'.

The impact of financial difficulties on mental health
Branch staff should know that financial difficulties can have a negative impact on mental health and that addressing financial problems early can reduce this impact. Even a customer who is making their repayments may still be experiencing financial difficulties and distress, e.g. they may not be meeting their other financial obligations.

Financial difficulties may also impact on mental health in a number of less obvious ways, including stress caused by the processes that institutions use to recover debt.

Branch staff should be aware that when a customer sees no way back from their financial difficulties they might become suicidal or attempt suicide.

Support for customers with mental health problems
Branch staff should adopt a respectful and positive approach when working with customers with mental health problems.

When working with people with mental health problems, branch staff should consider some of the following strategies:
- Allowing the customer to choose their preferred method of contact, including via letters, phone or Skype
- Offering to give the customer a plain language summary of information and options discussed during their conversation.

If a tailored collection process has been put in place, but the customer is not adhering to these arrangements, branch staff should:
- Encourage authorised third party representation, e.g. a professional, such as a community lawyer or financial counsellor, who can advocate on their behalf
- Ensure any authorised third party representatives are contacted.

Relevant laws
Branch staff should have a general understanding of their state and territory laws regarding the appointment of a third party to manage a customer's finances, e.g. guardianship, administration orders, financial management orders.

They should also be aware of what a financial administration order is and the implications for the customer and the financial institution.

Cultural considerations
Branch staff should be familiar with the cultural considerations of working with the customer from a culturally and linguistically diverse heritage.

Working with other professionals
Branch staff should know that financial counselling services play an important role for people with mental health problems and financial difficulties, including advocating on the behalf of the customer and making appropriate referrals to

Draft. Not for distribution.

mental health services. Therefore, branch staff should offer to refer any customers who have disclosed mental health problems to a financial counsellor.

If the customer is not working with a financial counsellor, but has a mental health professional, branch staff should consider working with the professional to determine the customer's capacity. The following issues should be addressed:

- The customer's ability to engage meaningfully with the financial institution
- The customer's capacity for understanding
- The customer's capacity for decision-making.

Guidelines for Contact Centre Staff

Knowledge of mental health problems

Contact centre staff should be aware of what a mental illness is and how common mental illnesses are in the community. They should also be aware of the risk factors for suicide.

Contact centre staff should also know that the impact of mental health problems will differ from one person to another, and may vary over time.

Box

Definitions of mental illness and mental health problems

'Mental illness' is a diagnosable illness that affects a person's thinking, emotional state and behaviour, and disrupts the person's ability to work or carry out other daily activities and engage in satisfying personal relationships.

'Mental health problems' is a broader term including mental illnesses, symptoms of mental illnesses that may not be severe enough to warrant the diagnosis of a mental illness, as well as mental health-related crises such as having thoughts of suicide.

The links between mental health problems and financial difficulties

Contact centre staff should be aware that mental health problems can contribute to financial difficulties, and vice versa, e.g. failing to pay bills because of depression or experiencing suicidal thoughts due to financial difficulties. Contact centre staff should be aware that having mental health problems is not necessarily an indicator of an inability to manage money.

The impact of mental health on financial difficulties

Contact centre staff should know how the symptoms of mental health problems may impact on financial difficulties. For instance, a customer may not complete financial tasks because they forget, lack the motivation to pay bills, make poor financial decisions, or feel overwhelmed by financial tasks, such as communicating with creditors, opening mail, or visiting Centrelink. Contact centre staff should also know that overspending and financial difficulties can be a sign of relapse of the customer's mental health problems. There are also some

Draft [illegible] distribution.

specific sources of financial difficulties faced by people with mental health problems (see box).

Box

Specific sources of financial difficulties for people with mental health problems may include:

- Debt incurred to support a drug or alcohol problem
- Increased medical expenses
- Problem gambling associated with bipolar disorder
- Overspending when in a manic or depressed state
- Job instability associated with episodic mental illness
- Poor money management due to paranoid thoughts, e.g. withdrawing money from the bank to keep it 'safe'.

The impact of financial difficulties on mental health

Contact centre staff should know that financial difficulties can have a negative impact on mental health and that addressing financial problems early can reduce this impact. Even a customer who is making their repayments may still be experiencing financial difficulties and distress, e.g. they may not be meeting their other financial obligations.

Financial difficulties may also impact on mental health in a number of less obvious ways, including stress caused by the processes that institutions use to recover debt.

Contact centre staff should be aware that when a customer sees no way back from their financial difficulties they might become suicidal or attempt suicide.

Support for customers with mental health problems

Contact centre staff should adopt a respectful and positive approach when working with customers with mental health problems and should consider implementing the following strategies:

- Allowing the customer to choose their preferred method of contact, including via letters, phone or Skype
- Offering to give the customer a plain language summary of information and options discussed during their phone conversation
- Requesting that a customer be moved from an automated to a tailored collection process.

If a tailored collection process has been put in place, but the customer is not adhering to these arrangements, contact centre staff should:

- Encourage authorised third party representation, e.g. a professional, such as a community lawyer or financial counsellor, who can advocate on their behalf
- Ensure any authorised third party representatives are contacted.

Relevant laws

Contact centre staff should be aware of what a financial administration order is and the implications for the customer and the financial institution.

Draft. Not for distribution.

Working with other professionals

Contact centre staff should know that financial counselling services play an important role for people with mental health problems and financial difficulties, including advocating on the behalf of the customer and making appropriate referrals to mental health services. Therefore, contact centre staff should offer to refer any customers who have disclosed mental health problems to a financial counsellor.

If the customer is not working with a financial counsellor, but has a mental health professional, contact centre staff should consider working with the professional to determine the customer's capacity. The following issues should be addressed:

- The customer's ability to engage meaningfully with the financial institution
- The customer's capacity for understanding
- The customer's capacity for decision-making.

Communication Guidelines for Financial Institution Staff

The guidelines in this section are about communicating with a customer with mental health problems and financial difficulties, both over the phone and when meeting with the customer face-to-face. In cases where it is only applicable to the phone or face-to-face communication this will be indicated. Most of these guidelines apply to the following financial institution staff: hardship, collections, branch and contact centre staff. The few statements that apply only to one role are identified as such.

Personal attitudes, beliefs and judgements influence communication, therefore it is important for staff to be aware of their own attitudes about mental health problems and how these might affect their ability to respond to the customer non-judgmentally. They should endeavour to set aside any negative beliefs and avoid expressing any negative reactions and judgements, in order to focus on the needs of the customer they are assisting.

General communication skills

Staff should know how to communicate effectively with customers with mental health problems.

Staff should take time to listen to their customers with mental health problems, allowing them enough time to think and talk, by:

- Letting the customer set the pace and style of the conversation, as far as possible
- Listening non-judgmentally so that the customer can feel like they have been heard and understood
- Showing empathy, by using statements such as, "this must be very difficult for you", and convey acceptance to the customer

Draft. Not for distribution.

- Conveying genuineness to the customer, i.e. the staff member's body language and tone of voice match what they are saying
- Respecting the customer's feelings and opinions, even if the staff member does not agree with them.

Staff should use the following communication skills:

- Ask questions that indicate care, e.g. clarifying questions to show that they understand what the customer is saying
- Check their understanding by re-stating what the customer has said and summarise facts and feelings
- Listen not only to what the customer says, but how they say it, i.e. their tone of voice
- Use prompts, such as "I see" and "Ah", when necessary to show the customer that they are listening
- Be patient, even when the customer may not be communicating well, may be repetitive or may be speaking slowly and unclearly
- Allow pauses and silences to give the customer time to think or gather their thoughts.

To facilitate communication, staff should avoid:

- Attempting to say something positive which may be perceived as trivialising the customer's experiences, such as "put a smile on your face" or "lighten up"
- Attempting to say something supportive which may be perceived as belittling or dismissing the customer's feelings, such as "you don't seem that bad to me", "get your act together"
- Speaking in a tone of voice which may be perceived as patronising
- Labelling the customer's feelings for them, e.g. "you're depressed"
- Pressuring the customer to talk about their feelings
- Being critical or expressing frustration at the customer for being in a negative emotional state
- Interrupting the customer when they are speaking, especially to give their opinion
- Using a hostile or sarcastic tone when the customer's responses are not what they expect
- Confrontation, unless it is necessary to prevent harmful or dangerous acts.

If the customer is unable to communicate effectively, staff should discourage the customer from making any financial decisions until they are able to communicate effectively. If speaking over the phone, the staff should offer to call them back at a better time.

They should be familiar with and listen for signs that may indicate that the customer is:

- Having memory or concentration problems
- Distressed
- Confused or out of touch with reality

Draft. Not for distribution.

- Aggressive
- Behaving in a way that leads to concern for safety.

Additional considerations for when the customer has memory and concentration problems
Staff should be aware that customers with mental health problems may have memory and concentration problems. Where this is the case, it can be helpful to give simple and concise information about their situation and repeat information.

Addition considerations when the customer is distressed
The financial institution should have a list of statements that their staff can paraphrase, using their own words, to help guide telephone conversations when talking to the customer who appears to be distressed, e.g. "It is understandable that you are anxious at a time like this given everything that is happening for you at the moment" or "Lots of people say that going through financial difficulties can be very stressful", or "It can be important to talk to someone about what is happening. Is there someone who can give you that support?"

Additional considerations when the customer is confused or out of touch with reality
Staff should tailor their interaction to the way the customer is behaving. If the customer is confused, staff should respond to the customer by communicating in a simple and concise manner. It is important to be patient and allow time for the customer to process the information and respond to what has been said, repeating things if necessary.

If the customer is out of touch with reality, staff should be sensitive to this and reassure them they are there to assist. Staff should be aware that delusions and hallucinations are very real to the customer. While not agreeing with the customer's delusions and hallucinations, staff can acknowledge how they may feel, e.g. "that must be difficult for you" or "I can see that you are upset".

So as not to worsen the situation, staff should not:
- Assume the customer cannot understand what is being said
- Raise their voice as if speaking to someone who is hard of hearing
- Dismiss, minimise or argue with the customer about their confusion, delusions or hallucinations
- Act alarmed, horrified or embarrassed by the customer's confusion, delusions or hallucinations
- Laugh at the customer's confusion, delusions or hallucinations
- Encourage or inflame any paranoia.

When hardship staff are working with a customer who is confused or out of touch with reality, they should be aware that the customer may be frightened by their thoughts and feelings and this fear may lead them to deny that anything is wrong.

Draft. Not for distribution.

Additional considerations when the customer is behaving aggressively
If a customer is behaving aggressively it is important not to take the aggressive behaviour personally and understand that they may be feeling confused or frightened. Be aware that their symptoms or fear causing their aggression might be exacerbated if certain steps are taken, e.g. involving the police. The following strategies can be helpful when working with an aggressive customer:

- Remain as calm as possible and avoid nervous behaviour, e.g. shuffling your feet, fidgeting, making abrupt movements
- Use positive words, such as 'stay calm', rather than negative words, such as 'stop shouting'
- Speak to the customer slowly and confidently with a gentle, caring tone of voice
- Explain to the customer that although you would like to help them, it is not possible when they are (state the unacceptable behaviour, e.g. yelling)
- Consider taking a break from the conversation to allow the customer a chance to calm down.

So as not to increase fear or aggressive behaviour, staff should avoid:

- Raising their voice or talking too fast
- Responding in a hostile, disciplinary or challenging manner
- Arguing with the customer
- Threatening them
- Restricting the customer's movement (e.g. if the meeting is face to face and he or she wants to pace up and down the room).

After an encounter with a customer who is behaving aggressively the staff member should take a break and discuss it with a supervisor.

Additional considerations when there is a concern about safety
If staff are concerned about the safety of the customer, themselves or others, they should:

- Take any threats or warnings seriously, particularly if the customer believes they are being persecuted
- Explain that this cannot be kept confidential
- Consider terminating the interview
- Seek assistance from a supervisor
- Contact the local mental health service and the authorised third party, if there is one, to discuss their concerns about the customer
- Take any necessary protective action, e.g. contact the police or mental health crisis team.

If the contact is by phone, staff should also try to ascertain the customer's whereabouts.

Draft. Not for distribution.

Policies and Procedures for Financial Institutions

Financial institutions should develop policies and procedures to better assist people with mental health problems and financial difficulties. In developing these policies and procedures, they should work with state and national financial counselling, mental health and community service organisations, for example using a review panel with a range of expertise to ensure all appropriate options are considered. The financial institution should follow the hardship provisions in the most recent Code of Banking Practice. The financial institution should make these policies available to financial counsellors.

As a guiding principle, financial institutions should be flexible in their arrangements with people experiencing mental health problems and financial difficulties. They should have specific procedures for:

- Handing on calls when the staff member requires support to manage the situation
- Redirecting the customer away from the usual collections process to an appropriately skilled team/staff member
- Allowing for extra time to work with people with mental health problems, e.g. longer conversations, additional time to provide written confirmation of communication, more frequent calls
- Encouraging third party representation, e.g. a professional, such as a community lawyer or financial counsellor
- Referring the customer to mental health or financial counselling services
- Reviewing and taking action at key points in the collection process, e.g. sale of assets or sale of debt
- Only initiating court action to pursue the customer's debt as a last resort
- Working with the customer with mental health problems in complex, crisis or sensitive situations, e.g. threats to harm self or others, sale of a family home
- Deferring action when the customer is acutely unwell or in a mental health crisis
- Dealing with advance directives.

Financial institutions should have policies and procedures for using the *Mental Health and Financial Difficulties Impact Form*. These policies and procedures should include:

- How the information will be used and who will have access to it
- Informing the customer how the information will be used
- The types of health professionals who can complete the form
- The length of time the information is held by the financial institution.

This policy should be available to the customer.

Mental health training for staff

Financial institutions should provide their staff with mental health awareness training that is tailored to the role and the amount of contact they are likely to have with people with mental health problems. This training should include:

Draft. Not for distribution.

- How branch staff can identify people with mental health problems and financial difficulties and refer them to a specialised team within the bank, e.g. hardship team
- How to assist someone over the phone who is having a mental health crisis
- Hearing about the lived experience of people who have had mental health problems and financial difficulties
- Learning about the impact of mental health problems and financial difficulties on the family of the customer
- Information about internal and external resources available to staff should they feel distressed by their work with people with mental health problems
- Periodic refresher training.

Financial institutions that use specialist teams to work with people with mental health problems should consider how the expertise of these teams can be shared with other colleagues.

Financial institutions should publicise and share examples of good practice from within their own organisation with colleagues across the industry.

Mental health information for customers
In providing relevant information about financial hardship to their customers, financial institutions should include mental illness as a possible contributor to financial difficulties.

Sections of financial institutions' websites that deal with financial difficulties should have links to information which assist people in a mental health crisis, e.g. Lifeline, *beyondblue*.

Draft. Not for distribution.

4 Guidelines for Mental Health Professionals

The links between mental health problems and financial difficulties

The mental health professional should be aware that mental health problems can contribute to financial difficulties, and vice versa, e.g. failing to pay bills because of depression or experiencing suicidal thoughts due to financial difficulties.

The mental health professional should know that the cycle of financial difficulties and mental health problems can be intensified for people from culturally and linguistically diverse heritage, due to the difficulty of navigating unfamiliar financial and health systems in Australia.

The impact of mental health problems on financial difficulties

The mental health professional should know how the symptoms of mental health problems may impact on financial difficulties. For instance, a client may not complete financial tasks because they forget, lack the motivation to pay bills, make poor financial decisions, or feel overwhelmed by financial tasks, such as communicating with creditors, opening mail, or visiting Centrelink. Mental health professionals should be aware that financial stability may not be an immediate goal for some clients in the early stages of recovery from mental illness. The mental health professional should also know that overspending and financial difficulties can be a sign of relapse of the client's mental health problems. There are also some specific sources of financial difficulties faced by people with mental health problems (see box).

Box

Specific sources of financial difficulties for clients with mental health problems may include:

- Debt incurred to support a drug or alcohol problem
- Increased medical expenses
- Problem gambling associated with bipolar disorder
- Overspending when in a manic or depressed state
- Job instability associated with episodic mental illness
- Poor money management due to paranoid thoughts, e.g. withdrawing money from the bank to keep it 'safe'.

The impact of financial difficulties on mental health

The mental health professional should know that financial difficulties can have a negative impact on mental health and that addressing financial problems early can reduce this impact. Like many people in the community, people with mental health problems may have limited financial literacy skills. It is also important to know that the way the client perceives their financial situation is what impacts on their mental health, rather than their actual situation. Even a client who is making their repayments may still be experiencing financial difficulties and distress.

Financial difficulties may also impact on mental health in a number of less obvious ways, including:

Draft. Not for distribution.

- Isolation and lack of support due to the inability to participate in social activities
- Worsening of mental health problems due to an inability to pay for treatment
- Stress caused by the processes that institutions use to recover debt.

The mental health professional should be aware that when a client sees no way back from their financial difficulties they might become suicidal or attempt suicide.

Supporting clients with financial difficulties

The mental health professional should know how they can support a client with financial difficulties. The mental health professional should ask direct questions about the client's financial situation, and whether they have any financial difficulties, particularly if overspending is a feature of the client's mental health problem.

If the client has financial difficulties, the mental health professional should consider the following strategies:

- Advise them to seek the help of a financial counsellor
- Discourage the person from ignoring their financial difficulties and tell them that dealing with these difficulties sooner rather than later may make it more manageable
- Encourage them to delay making large purchases until they have discussed them with their support person
- Encourage them to apply for the range of concessions and financial assistance available, e.g. reduced car registration, council rates and energy costs to health care card holders
- If there is difficulty in paying utility bills (gas, electricity, water), encourage them to contact the utility provider and request 'hardship' assistance, as utility providers have, by law, provisions for people in financial hardship.

Mental health professionals should be aware that every state and territory has an ombudsman to deal with difficulties with energy providers.

Most financial institutions have policies about assistance for people who are experiencing financial difficulties. Mental health professionals should know about these policies and the positive role that financial institutions can play for people with mental health problems and financial difficulties. In cases where the client is not seeing a financial counsellor, the mental health professional should encourage them to contact their financial institution as soon as they think they will experience financial difficulties.

The mental health professional should not make payment arrangements with creditors regarding the client's debt without consulting with a financial counsellor, as there are specific consumer protection laws that might mean the debt does not need to be paid.

Draft. Not for distribution.

If the mental health professional has provided information about their client's ability to make decisions concerning their financial situation they should review this on a regular basis or when the person's mental health condition changes significantly. See section on *Mental Health and Financial Difficulties Impact Form*.

Resources

The mental health professional should be aware of the following resources, how to access them and encourage their client to use them:

- Centrelink benefits for people with mental health problems
- Financial counselling services.

They should explain what financial counsellors do and the potential benefits to their clients with financial difficulties and should provide them with information about local financial counselling services. They should display signs about financial counselling services in their offices.

Relevant laws and professional codes of conduct

The mental health professional should have a thorough working knowledge of relevant laws and professional codes of conduct related to:

- The appointment of a third party to manage their client's finances, e.g. financial administration order
- The interactions between a client with mental health problems and creditors

Working with financial counsellors

With the client's consent, the mental health professional and the financial counsellor should work together. They should:

- Deliver consistent messages to the client
- Ensure that the financial counsellor's client service plan complements the mental health treatment plan
- Be aware of and discuss any conflicting priorities between the financial and treatment goals
- Determine whether the person is able to make specific decisions concerning their current financial situation
- Work with the client and support person to identify the specific supports needed to help the client make financial decisions
- Use the *Mental Health And Financial Difficulties Impact Form* to communicate information about how the client's mental health problems impact on their financial difficulties. See section on *Mental Health and Financial Difficulties Impact Form*.

Training for mental health professionals

The mental health professional should be trained in the following basic skills in order to prevent and manage financial difficulties in their clients:

- Assisting the client to identify how their symptoms impact on their financial difficulties
- Assessing the pattern or history of the client's financial concerns

Draft. Not for distribution.

- Assisting the client to identify behaviours that have led to financial difficulties, e.g. impulsive spending, unstable work history.

Draft. Not for distribution.

5 Guidelines for the Support Person

This section presents the guidelines for the support person. It includes guidelines about what the support person needs to know and do when supporting the person with mental health problems and financial difficulties. It may be that a person with mental health problems and financial difficulties has a support person, such as someone who is a significant source of emotional and/or practical support or assistance. A support person may be a partner, close friend or family member. However, it is not assumed that every person has a support person.

Box

Definitions of mental illness and mental health problems

'Mental illness' is a diagnosable illness that affects a person's thinking, emotional state and behaviour, and disrupts the person's ability to work or carry out other daily activities and engage in satisfying personal relationships.

'Mental health problems' is a broader term including mental illnesses, symptoms of mental illnesses that may not be severe enough to warrant the diagnosis of a mental illness, as well as mental health-related crises such as having thoughts of suicide.

Draft. Not for dist.

The links between mental health problems and financial difficulties

The support person should be aware that mental health problems can contribute to financial difficulties, and vice versa, e.g. failing to pay bills because of depression or experiencing suicidal thoughts due to financial difficulties.

The impact of mental health on financial difficulties

The support person should know how the symptoms of mental health problems may impact on financial difficulties. For instance, the person may not complete financial tasks because they forget, lack the motivation to pay bills, make poor financial decisions, or feel overwhelmed by financial tasks, such as communicating with creditors, opening mail, or visiting Centrelink. The support person should be aware that financial stability may not be an immediate goal for some people in the early stages of recovery from mental illness. The support person should also know that overspending and financial difficulties can be a sign of relapse of the person's mental health problems. There are also some specific sources of financial difficulties faced by people with mental health problems (see box).

Box

Specific sources of financial difficulties for people with mental health problems may include:

- Debt incurred to support a drug or alcohol problem
- Increased medical expenses
- Problem gambling associated with bipolar disorder
- Overspending when in a manic or depressed state
- Job instability associated with episodic mental illness

- Poor money management due to paranoid thoughts, e.g. withdrawing money from the bank to keep it 'safe'.

The impact of financial difficulties on mental health
The support person should know that financial difficulties can have a negative impact on mental health and that addressing financial problems early can reduce this impact. Even a person who is making their repayments may still be experiencing financial difficulties and distress. It is also important to know that the way the person perceives their financial situation is what impacts on their mental health, rather than their actual situation.

Financial difficulties may also impact on mental health in a number of less obvious ways, including:

- Isolation and lack of support due to the inability to participate in social activities
- Worsening of mental health problems due to an inability to pay for treatment
- Stress caused by the processes that institutions use to recover debt.

The support person should be aware that when a person sees no way back from their financial difficulties they might become suicidal or attempt suicide.

The support person should be aware of the warning signs and risk factors for suicide and ask about suicide if they think the person may be having thoughts of suicide. They should be aware of the *First Aid Guidelines for Assisting the Person Who is Suicidal* (download at www.mhfa.com.au).

Supporting the person

Financial counsellors and financial institutions can play an important role for people with mental health problems and financial difficulties, e.g. promoting financial inclusion. By asking direct questions about the person's financial situation, and whether they have any financial difficulties, the support person will be able to refer the person for appropriate advice if necessary. The support person should also know the Centrelink benefits for people with mental health problems and encourage the person to use them.

The support person should be aware of the following:

- The financial behaviours that may indicate the person is becoming unwell
- Most financial institutions will have policies about assistance for people who are experiencing financial difficulties
- What a financial administration order is and the implications for the person and their dealings with the financial institution.

The support person should consider the following strategies for helping the person with mental health problems and financial difficulties:

- Discourage the person from ignoring their financial difficulties
- Encourage the person to delay making large purchases until they have discussed them with their support person

Draft. Not for distribution.

- Work with the financial counsellor and mental health professional to determine what specific supports the person needs to enable them to make financial decisions
- If memory or concentration problems are present, use simple and concise information about their financial situation and repeat this information, if necessary.

Draft. Not for distribution.

6 Mental Health and Financial Difficulties Impact Form

This section presents the guidelines for the development and use of the *Mental Health and Financial Difficulties Impact Form*[1].

There should be a standardised form that a mental health professional can complete to communicate information about a person's mental health problems to financial counsellors and financial institutions. This form will assist in understanding the impact of the person's current mental health problems on their ability to understand and manage their current financial situation. Its purpose is to allow the financial counsellor and the financial institution staff to work collaboratively to find the most appropriate solution to the person's financial difficulties.

The following information should be included in the form:

- Name, contact details and profession of the person who is requesting that the form be completed, e.g. financial institution staff, the financial counsellor.
- A disclosure statement from the requestor, indicating what the information on this form could be used for both now and in the future.
- Details of how the person's mental health problems affect their ability to resolve their current financial difficulties.
- The person's capacity for and level of understanding.
- The person's capacity for decision-making.
- Any factors that may be relevant to communicating effectively with the person about their financial difficulties, e.g. preferred mode of communication, comprehension problems.
- A statement about how long the information on the form is valid for.
- Details of any support the person is receiving with regards to their financial difficulties.
- Option for listing an authorised third party representative who the financial institution can contact if they are unable to contact the person.
- The person's signature to indicate their informed consent to give this form to the financial institution and the financial counsellor.
- Signature and contact details of the mental health professional completing this form.

A copy of the completed form should be given to the person (and their support person, where appropriate).

If the person consents, the mental health professional who completes the form should review it and resubmit if needed, when the person's mental health problems change.

Draft. Not for distribution.

---

[1] This form is still under development, but will be made available when it is complete.
